# On the Behavior of the Ethylene Glycol Components of Polydisperse Polyethylene Glycol PEG200

**DOI:** 10.1021/acs.jpcb.2c06773

**Published:** 2023-01-26

**Authors:** Markus M. Hoffmann, Matthew D. Too, Nathaniel A. Paddock, Robin Horstmann, Sebastian Kloth, Michael Vogel, Gerd Buntkowsky

**Affiliations:** † Department of Chemistry and Biochemistry, State University of New York College at Brockport, Brockport, New York14420, United States; ‡ Institute of Condensed Matter Physics, Technical University Darmstadt, Hochschulstraße 6, 64289Darmstadt, Germany; § Institute of Physical Chemistry, Technical University Darmstadt, Alarich-Weiss-Straße 8, D-64287Darmstadt, Germany

## Abstract

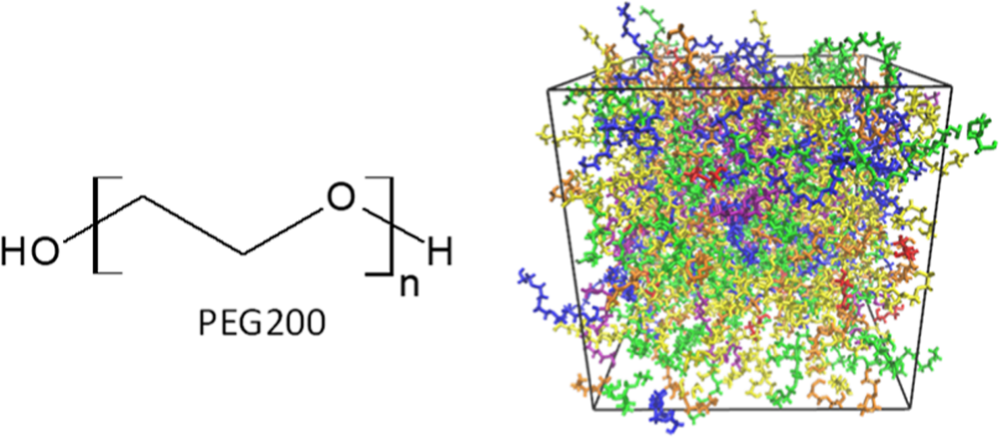

Molecular dynamics (MD) simulations are reported for [polyethylene glycol (PEG)200], a polydisperse mixture of ethylene glycol oligomers with an average molar weight of 200 g·mol^–1^. As a first step, available force fields for describing ethylene glycol oligomers were tested on how accurately they reproduced experimental properties. They were found to all fall short on either reproducing density, a static property, or the self-diffusion coefficient, a dynamic property. Discrepancies with the experimental data increased with the increasing size of the tested ethylene glycol oligomer. From the available force fields, the optimized potential for liquid simulation (OPLS) force field was used to further investigate which adjustments to the force field would improve the agreement of simulated physical properties with experimental ones. Two parameters were identified and adjusted, the (HO)–C–C–O proper dihedral potential and the polarity of the hydroxy group. The parameter adjustments depended on the size of the ethylene glycol oligomer. Next, PEG200 was simulated with the OPLS force field with and without modifications to inspect their effects on the simulation results. The modifications to the OPLS force field significantly decreased hydrogen bonding overall and increased the propensity of intramolecular hydrogen bond formation at the cost of intermolecular hydrogen bond formation. Moreover, some of the tri- and more so tetraethylene glycol formed intramolecular hydrogen bonds between the hydroxy end groups while still maintaining strong intramolecular interactions with the ether oxygen atoms. These observations allowed the interpretation of the obtained RDFs as well as structural properties such as the average end-to-end distances and the average radii of gyration. The MD simulations with and without the modifications showed no evidence of preferential association of like-oligomers to form clusters nor any evidence of long-range ordering such as a side-by-side stacking of ethylene glycol oligomers. Instead, the simulation results support the picture of PEG200 being a random mixture of its ethylene glycol oligomer components. Finally, additional MD simulations of a binary mixture of tri-and hexaethylene glycol with the same average molar weight as PEG200 revealed very similar structural and physical properties as for PEG200.

## 1 Introduction

**Figure 1:**
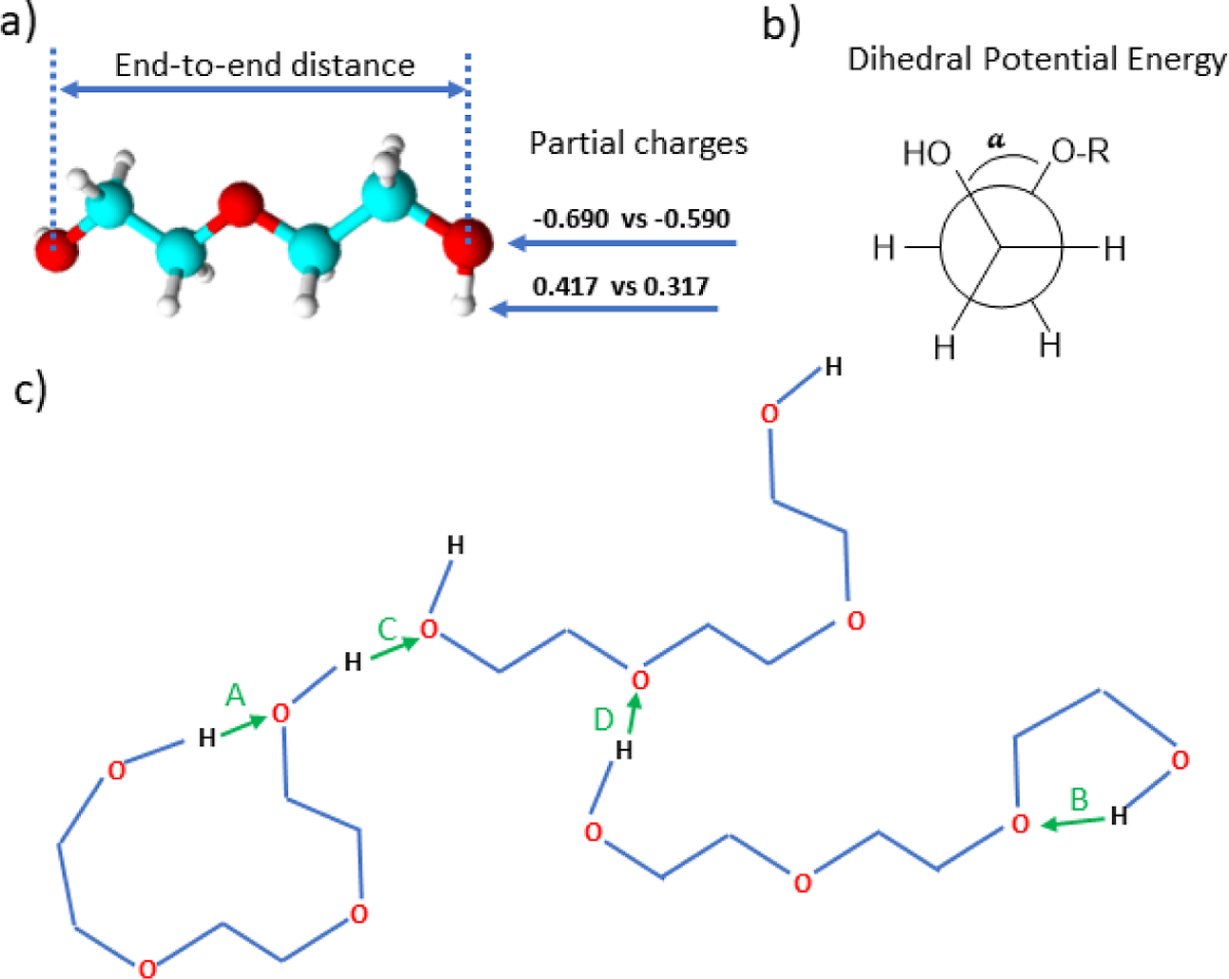
(a) Diethylene glycol as an example of ethylene glycol oligomers that make up PEG200 and H-[O-CH_2_-CH_2_]_*n*_-OH shown with the distance between the oxygen atoms of the terminal hydroxy end groups used in this report as a measure for the end-to-end distance and changes to the hydroxy partial charges explored in this report; (b) illustration of the proper dihedral angle referred to as (HO)–C–C–O in this report for which its potential energy was changed; (c) illustration of possible hydrogen bonding interactions: (A) intramolecular hydrogen bonding between hydroxy end groups (OH–OH), (B) intramolecular hydrogen bonding between hydroxy end group and an ether oxygen atom (OH–OE), and (C,D) is the same as (A,B) but between two different molecules, i.e., intermolecular hydrogen bonding.

Polyethylene glycol (PEG, H-[O-CH_2_-CH_2_]_*n*_-OH, see Figure 1 for diethylene glycol with *n* = 2) is an industrially important chemical with annual production on the order of 500,000 tons per year.^1^ In many applications, chiefly in the personal and health care industries,^2−4^ PEG is used as an additive. However, there are also applications where PEG is present as a major component of the system. These applications include the use of PEG as a heat transfer medium^5^ or as a chemical solvent.^6^ For the latter, there are several physical and chemical properties of PEG that are very favorable from an environmental and chemical safety point of view including being non-toxic, possessing a low vapor pressure, which reduces exposure through inhalation, and being biodegradable.^7^ Besides these favorable properties of PEG as a green chemical solvent, PEG is also attractive because of its relatively low cost, and its solvent capability may surpass that of ionic liquids for some solutes^8^ and even allow PEG to be used as a solvent for several mineral salts.^9^ Consequently, interest in PEG as an attractive alternative solvent for chemical synthesis and separations has grown as evidenced by several recent review articles on this subject.^10−12^

As recently pointed out,^6^ despite the increased interest in PEG as a chemical solvent, there is presently a dearth of studies devoted to improving a general understanding of how PEG behaves as a solvent. Important here is that PEG is industrially produced as a polydisperse mixture, where the average molar mass can be tuned to certain target values as indicated by the product name. For example, the average molar mass of PEG200 is approximately 200 g·mol^–1^. To the best of our knowledge, there have not been any investigations to discern the molecular behavior of these mixtures of ethylene glycol oligomers. The main motivation for the study reported here was to find an answer to the question of whether the ethylene glycol oligomers of various chain lengths mix homogeneously or if there is any indication of preferential interactions between ethylene glycols of similar chain lengths, potentially leading to clusters of ethylene glycol oligomer subgroups. Molecular dynamics (MD) simulations are well suited to address this question. However, force fields describing accurately the inter-atom interactions are required for MD simulations. A perusal of the literature revealed that while there are many more force fields available for the structurally related glymes, there are only a few force fields for PEGs, including AMBER,^13,14^ CHARMM,^15,16^ GROMOS,^17^ optimized potential for liquid simulations (OPLS),^18^ Martini,^19^ and a specific force field from the Müller-Plathe group.^20^ These force-fields were oftentimes developed and tested for aqueous solutions of PEG but not neat PEG. The GROMOS forcefield was recently optimized for PEG2000, which however is solid at ambient conditions.^21^ The GROMOS force field was also recently used for a study of a longer chain PEG polymer in aqueous and methanol solution.^22^ By the time of writing this report, we became aware of a study of PEG polymers intercalated between modified silicate hydrate^23^ that used the consistent valence force field,^24^ which we did not pursue in this report.

As a first step, we tested available force fields for accuracy against available experimental data, foremost density, self-diffusion coefficients, and dynamic viscosity (henceforth referred to as just “viscosity”).^25,26^ As we will show, none of the tested force fields produced a satisfactory agreement with literature data for all ethylene glycol oligomers. Therefore, some of the parameters of the OPLS force field, which produced the closest agreement with the literature data overall, were explored and adjusted to obtain simulated physical properties in better agreement with experimental data. The standard OPLS force field as well as the adjusted OPLS force field were then used to simulate mixtures representing commercial PEG200. The effects of the forcefield adjustments on the hydrogen bonding behavior are carefully inspected, which then aids in explaining and interpreting changes in the radial distribution functions and structural as well as physical properties. The obtained insights should be useful not only for the application of PEG as a solvent but in other research areas where PEG is a major component as well, such as, for example, PEG as a crowding agent in biochemical studies.^27^ Hence, the organization of this report is as follows: details on carrying out the MD simulations and analyzing the obtained trajectories are provided in the Methods section. The Results and Discussion section begins with a summary of the MD simulations conducted to test the accuracy of available force fields for reproducing experimental data, chiefly density and self-diffusion coefficients, beginning first with simulations on diethylene glycol and then tetraethylene glycol. Next, the outcomes from optimizing modifications to the OPLS force fields are summarized before finally turning to MD simulations on PEG200, where the effects of the modifications to the OPLS force field on the hydrogen bonding behavior as well as structural and physical properties are described and explained in depth. The main insights are summarized in the Conclusion section. Finally, it should be noted that extensive Supporting Information is provided with the goal of being as transparent as possible with respect to specific simulation settings and analysis procedures.

## 2 Methods

### 2.1 Force Fields

All simulations of either neat ethylene glycol oligomers or mixtures were carried out using GROMACS 2020.4^28−35^ and compiled with mixed precision. Compared to typical organic solvents, which oftentimes have a viscosity of less than 1 mPa·s at ambient conditions,^36^ PEG200 is much more viscous.^25^ To increase the rate of motion with a concurrent reduction in simulation time, simulations were carried out at a temperature of 328 K, which is in the middle of the temperature range 298–358 K of available literature data on density, viscosity, and self-diffusion coefficients.^25,26^ The composition of PEG200, a mixture of di through heptaethylene glycol, was taken from Hoffmann et al.^25^ The majority of the results were obtained with the all-atom OPLS (OPLS/AA, henceforth referred to as “OPLS”) force field developed by the Jorgensen group.^18^ For oligomers of PEG200, for which no improper torsions are applicable, the potential function is
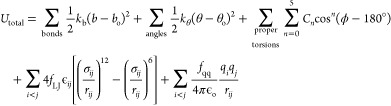
1where the Lennard–Jones (LJ) interaction parameters contact distance, σ, and well-depth, ε, between two atoms are calculated according to geometric mean combination rules  and . The fudge factors *f*_LJ_ and *f*_qq_ for Lennard Jones and Coulomb interactions, respectively, are zero for nonbonding interactions between 1–2 and 1–3 atom pairs, 0.5 for 1–4 pairs, and 1 for all other atom pairs, which means that nonbonding interactions are only calculated between atoms if they are located more than three covalent bonds apart or are present on different molecules. Bonds and angles are represented by simple harmonic oscillators with spring constants *k*_b_ and *k*_θ_ and equilibrium bond lengths/angles *b*_0_ and θ_0_, respectively, while proper dihedrals are represented by a Ryckaert–Bellemans potential with torsional energy barrier coefficients *C*_*n*_ for *n* = 0, 1, 2, 3, 4, 5 and torsional angle ϕ (see also Figure S1 in the Supporting Information).

Ethylene glycol oligomers using the OPLS force field were generated using the LigParGen web-server,^37−39^ which assigns LJ and bonding potential parameters according to the OPLS force field and calculates atomic charges using the 1.14*CM1A-LBCC charge model.^38^ We left the charges on the atoms as they were generated from the LigParGen web server even though they deviated slightly from being symmetric across each oligomer. However, we checked that a symmetrization of the partial charges of the oligomers had negligibly small effects on the studied properties. Other force fields were tested as well: CHARMM,^15,16^ AMBER,^13,40^ GROMOS,^17^ Martini,^19^ and a specific force field by the Müller-Plathe group.^20^ The Supporting Information displays all needed input files for running simulations with these force fields to ensure complete transparency and reproducibility of simulated results. While the simulation details provided next in Section 2.2 focuses on the OPLS force field, they largely apply also to the simulations with the other force fields.

### 2.2 Simulation Details

Systems of neat ethylene glycol oligomers or PEG200 were prepared by randomly inserting up to 1000 molecules total into a cubic box with a density approximately half that of the experimental density^25^ using the GROMACS insert-molecules module.^34,35^ For some simulations, a nonrandom distribution was desired and accomplished by using the freeware Packmol.^41,42^

Once prepared, systems were set through energy minimization using the steepest descent algorithm.^34^ An initial maximum displacement of 0.01 nm for each atom was used to remove high energy contacts generated during system preparation and was conducted until the system reached a local energy minimum or a maximum of 100,000 steps elapsed. Periodic boundary conditions in all 3 dimensions—*x*, *y*, and *z*—were used in conjunction with the atom-based Verlet cutoff scheme,^43^ which utilizes a buffer region with a buffer tolerance of 0.005 kJ mol^–1^ ps^–1^. All nonbonding interactions, including electrostatics and LJ interactions, were cut off at a distance of 1.4 nm. Smooth particle-mesh Ewald^44,45^ with cubic interpolation and a grid spacing of 0.168 nm was used for the treatment of long-range electrostatic interactions. No long-range analytic tail dispersion correction for energy and pressure^46^ nor any constraining of bonds was utilized during energy minimization.

After energy minimization, each system was simulated in the isothermal-isobaric *NPT* ensemble at 328 K and 1 bar using 2 fs time steps and initial velocities generated for each atom according to a Maxwell–Boltzmann distribution. The *NPT* simulation length was set long enough for the system density to converge within a tenth of the overall *NPT* simulation time. All bonds involving hydrogen in PEG oligomer molecules were constrained in every step using the LINCS algorithm with a fourth-order matrix expansion.^47^ Newton’s equations of motion were numerically integrated using the leap-frog algorithm,^48^ and the center of mass of the system was removed every 20 fs. Similar to that for energy minimization, the Verlet cutoff scheme^43^ (with the same buffer tolerance) with periodic boundary conditions was employed. Nonbonding interactions, including the 1.4 nm cutoff distance and the long-range treatment of electrostatic interactions, were identical to those for energy minimization except for the implementation of an analytic tail correction for energy and pressure for the long-range LJ potential.^46^ The system temperature was controlled using the Bussi–Donadio–Parrinello velocity-rescaling thermostat,^49^ a modified Berendsen thermostat^50^ containing an additional stochastic term that allows for the correct sampling of the kinetic energy distribution, with a time constant of 1.0 ps, while system pressure was controlled isotropically at 1 bar using the Parrinello–Rahman barostat^51,52^ set with an isothermal compressibility of 4.5 × 10^–5^ bar^–1^ and a time constant of 5.0 ps. A minimum of 1000 position frames were recorded for the trajectory, and a minimum of 10,000 frames were recorded for the energies.

After the *NPT* simulation, a position frame of average density from the converged region was taken from the trajectory and used as the starting configuration for a simulation in the canonical *NVT* ensemble set at 328 K. All parameters from the previous *NPT* simulation were used here except for pressure coupling, which was turned off. Although velocities were regenerated again to provide an average temperature of 328 K according to a Maxwell–Boltzmann distribution, we note that analysis of results would not be affected if an initial 1–2% of the trajectory was skipped, which is what was done here. The length of each simulation was set at least 100 times the structural relaxation times of the simulated PEGs^53^ to allow for the system to be ergodic. A minimum of 1000 position frames were recorded, and energy frames were recorded every 20 fs to accurately obtain shear viscosities via the Green–Kubo (GK) integral time decomposition^54^ method further detailed in Section 2.3.3.

### 2.3 Analysis

All analyses were conducted either using modules available in the GROMACS package^34,35^ or by self-generated scripts in either Bash or Python programming language. These scripts and other helpful scripts are included in the Supporting Information. Densities, isobaric thermal expansion coefficient, and isobaric molar heat capacity were analyzed from the *NPT* simulation trajectory beyond the time at which the density converged, while all other properties were analyzed from the *NVT* simulation trajectory. We note that additional *NVE* simulations were not conducted as the final stage of the simulation protocol to obtain dynamic properties because the temperature stability of long simulation times can be problematic. Furthermore, Basconi and Shirts have demonstrated that *NVT* simulations using proper temperature coupling schemes as used in this study lead to the same values as obtained with *NVE* simulations.^55^

#### 2.3.1 Density

The GROMACS energy module was used,^34,35^ which calculates the density from averaging instantaneous densities over the specified time frames of the trajectory. The standard deviation to the density value reported by the energy module of GROMACS is dependent on the size of the simulation box. Expressed in percent relative standard deviation (% RSD), 0.5–0.7 % RSD was obtained for simulations with 250 molecules and 0.2–0.4 % RSD for simulations with 1000 molecules. However, the % RSD obtained from repeated simulations was found to be even lower, below 0.2 and 0.05 % RSD for 250 and 1000 molecules, respectively.

#### 2.3.2 Self-Diffusion Coefficients

Following best practices,^56^ self-diffusion coefficients were found using the Einstein-relation^46^
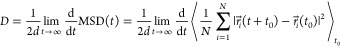
2where  refers to averaging over multiple time origins *t*_0_, which were increased in increments of 10 ps, *d* = 3 for a tridimensional system, and MSD(*t*) is the mean squared displacement at time *t* over all atoms *N*, which was obtained using the GROMACS *msd* module from the unwrapped trajectory. [We tested and confirmed that using *msd* module from wrapped vs unwrapped trajectory leads to the same MSD(*t*)]. A minimum of 1000 coordinate frames were used for that purpose. The MSD versus time plots were inspected to determine the diffusive regime to establish lower and upper time limits for linear regression analysis to obtain *D*(*L*) from MSD(*t*) = 6*D*(*L*) *t* + *c*, where the obtained values for the self-diffusion coefficients *D*(*L*) are dependent on the size of the simulation box, that is, the side length *L* of the cubic box. The analytic correction by Yeh and Hummer^57^
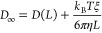
3was applied to obtain the self-diffusion coefficient at infinite box size, *D*_∞_, where *k*_B_ is the Boltzmann constant, *T* the temperature, ξ = 2.837298, and η the simulation viscosity. The uncertainty of the self-diffusion coefficient may be estimated by comparison of results when choosing the lower and the upper half of the chosen time range. With differences typically less than 3%, these variations were less than the 4–7 % RSD obtained from repeated simulations. Thus, the choice of the time range for the regression analysis was a contributing but not the main source for the uncertainty of the self-diffusion coefficient.

#### 2.3.3 Viscosity

Initially, the self-diffusion-based (D-based) method developed by Jamali et al. was followed.^58^ This method uses finite-size self-diffusivities from systems of at least two different system sizes to calculate the shear viscosity by the usage of eq 4, which is essentially eq 3 in rearranged form.
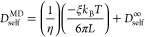
4where *D*_self_^MD^ is the finite-size self-diffusion coefficient for systems of different sizes, *D*_self_^∞^ is the self-diffusion coefficient in the thermodynamic/macroscopic limit, and the other symbols have the same meaning as for eq 3. To obtain η from eq 4 by linear regression, finite-size self-diffusion coefficients from multiple simulations (to improve statistics) of at least two different system sizes are required. Specifically, Jamali et al. determined the optimal setup for the D-based method to include two systems of different simulation box sizes, allocating 50–70% of total computational resources to the larger system, using at least 250 molecules for the smaller system, and having the larger system be at least 4× the size of the smaller system. Thus, we used 250 molecules and 1000 molecules. Although following this method worked reasonably well for obtaining viscosities from repeated simulations of diethylene glycol, it became evident that the box size difference would need to be higher when simulating higher ethylene glycol oligomers. The increased computational time for using a larger box size for higher ethylene glycol oligomers, which are also more viscous, became impractically long, so the D-based method of finding viscosities from MD simulations was abandoned, and only some viscosity results obtained from the D-based method are reported. Instead, the time decomposition method was followed, where the shear viscosity is obtained using the GK integral formalism, which relies on the integration of the pressure autocorrelation function (PACF)^46^
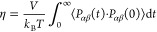
5where *V* is the volume of the box, *k*_B_ is the Boltzmann constant, *T* is the temperature (in K), *t* is the autocorrelation time, ⟨···⟩ represents an ensemble average, and *P*_αβ_ is an off-diagonal element of the pressure tensor. In eq 5, *P*_αβ_ is represented by
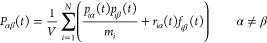
6where α,β = *x*, *y*, or *z* Cartesian coordinates, *N* is the total number of particles in the system, *p*_*i*α_ (or *p*_*i*β_) is the momentum of particle *i* in the α (or β) direction, *m*_*i*_ is the mass of particle *i*, *r*_*i*α_ is the position of particle *i* in the α direction, and *f*_*i*β_ is the total force on particle *i* in the β direction. Because the PACF decays fairly rapidly (i.e., 1–10 ps decay time for most systems), energies need to be recorded frequently during an MD simulation, which is why energies were recorded every 20 fs in all *NVT* simulations.^56^ As noted before,^54,59^ the PACF does not usually converge but instead shows large fluctuations at long autocorrelation times, even when averaging over the three off-diagonal elements of the pressure tensor—*P*_*xy*_, *P*_*xz*_, and *P*_*yz*_. These fluctuations make it difficult to determine the shear viscosity in a consistent manner from just single GK integrals. Rather than repeating MD simulations to improve statistics, which is the time decomposition method proposed by Zhang et al.,^54^ we split the full trajectory of a single *NVT* simulation into multiple time blocks, realizing that these time blocks of “short trajectories” should be relatively independent of one another. The number of time blocks, typically 25–100, was chosen as a compromise between having more time blocks, which would increase statistical accuracy and reduce fluctuation behavior, versus having larger-sized time blocks to ensure convergence. Examples of (a) the cancelation of fluctuations at long correlation time and (b) the effect of choosing time blocks of insufficient length can be seen in Figure S3 of the Supporting Information which also includes the Python and Bash scripts developed for our implementation of the time decomposition method. Analysis repetition from independent repeated simulation runs of di- to pentaethylene glycol resulted in about 15 % RSD. However, % RSD may potentially be higher for the most viscous systems simulated in this report where the PACF requires the most time to converge.

#### 2.3.4 Radial Distribution Functions

The radial distribution function (RDF) or pair correlation function between particles A and B with particle A as the central reference atom is calculated by
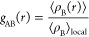
7where  is the average particle density of B at a distance *r* away from particle A, and  is the particle density of B averaged over all spheres centered upon particle A with a radius of half the box length. RDFs between two types of particles A and B were calculated using the GROMACS *rdf* module.^34,35^ We note that if particles A and B are entire molecules, the GROMACS *rdf* module iteratively goes through each atom of the reference molecule to sum up the distances to all atoms of the molecules of interest. Inter- and intramolecular RDFs were evaluated where we note that the intramolecular RDFs become zero and not unity at long *r* given the finite size of a molecule. The intramolecular RDFs were normalized to a total area of 1.

#### 2.3.5 Hydrogen Bonding

Hydrogen bond numbers at instantaneous points in time were determined using the GROMACS *hbond* module.^34,35^ To determine the number of hydrogen bonds between a donor atom (D) and an acceptor atom (A) at any point in time, a list of hydrogen bonding donor and acceptor groups was tabulated. The number of hydrogen bonds at an instantaneous point in time was then defined to be the number of donor–acceptor pairs that satisfied the criteria that the acceptor–donor-hydrogen triplet angle ≤30° and the acceptor–donor distance ≤0.35 nm, the distance for which the first local minima of the RDF between A and D occurs (see Section 3.5). We note that while these geometric criteria are the default criteria in the GROMACS *hbond* module, other hydrogen bond definitions exist based, for example, on energetics or topology.^60^ We did not explore the effect of using other criteria as we were more interested in trends of hydrogen bonding across the different ethylene glycol oligomers as a result of the modifications of the OPLS forcefield. The average number of hydrogen bonds was then calculated by averaging all the instantaneous hydrogen bond numbers over all trajectory frames using the GROMACS *analyze* module.^34,35^ Besides counting hydrogen bonds, additional comments are warranted on evaluating the possible number of hydrogen bonds. As can be seen in Figure 1, a H-[O-CH_2_-CH_2_]_*n*_-OH molecule possesses two hydrogen bonding donor protons, two hydroxy oxygen-hydrogen bonding acceptors, and *n* – 1 ether hydrogen bonding acceptors. A hydrogen already covalently bonded to an oxygen atom cannot also be hydrogen bonded to it. Thus, for *N* H-[O-CH_2_-CH_2_]_*n*_-OH molecules in a simulation there are the following hydrogen bonding donor–acceptor combinations (to which we will generally refer to in this report as *N*_possible_): 2*N* intramolecular hydroxy–hydroxy (OH–OH) hydrogen bonds and 4*N*(*N* – 1) intermolecular OH–OH hydrogen bonds adding up to 2*N*(2*N* – 1) total OH–OH hydrogen bonds as well as 2*N*(*n* – 1) intramolecular hydroxy–ether hydrogen (OH–OE) bonds and 2*N*(*N* – 1)(*n* – 1) intermolecular OH–OE hydrogen bonds adding up to 2*N*^2^(*n* – 1) total OH–OE hydrogen bonds. Reported are the intra- and intermolecular hydrogen bonds each oligomer experiences with its own kind, and the hydrogen bonds with any other oligomer but its own kind. The latter is, by default, all intermolecular in nature. It is important to note here that *N*_possible_ is proportional to *N* for intramolecular hydrogen bonds but is proportional to *N*(*N* – 1) for intermolecular hydrogen bonds, respectively. Thus, given that the count of detected hydrogen bonds, *N*_count_, is proportional to *N*, *N*_count_/*N*_possible_ is independent of *N* for intramolecular hydrogen bonds but not independent of intermolecular hydrogen bonding. This needs to be considered when comparing hydrogen bonding from simulations with differing numbers of *N* or comparing intra with intermolecular hydrogen bonding. Specifically, *N*_possible,inter_/*N*_possible,intra_ is equal to 2(*N* – 1) for OH–OH hydrogen bonding and equal to (*N* – 1) for OH–OE hydrogen bonding. From the analysis of repeated independent simulation runs of di- to pentaethylene glycol, the uncertainty of the number of hydrogen bonds was found to be less than 0.3 % RSD for intermolecular hydrogen bonds, less than 4 % RSD for intramolecular OH–OE hydrogen bonds, and less than 12% for intramolecular OH–OH hydrogen bonds.

#### 2.3.6 Average End-to-End Distances and Average Radii of Gyration

The GROMACS analysis modules^34,35^*distance* and *gyrate* were used to obtain average end-to-end distances and average radii of gyration. The hydroxy oxygen atoms of an H-[O-CH_2_-CH_2_]_*n*_-OH oligomer were chosen as a measure for the end-to-end distance, as shown in Figure 1. As will be discussed in Section 3.5.3, the end-to-end distances and the radius of gyration are dependent on the number of ethylene oxide repeat units *n*, ranging from 80–100% RSD to 1–3% RSD, respectively.

#### 2.3.7 Isobaric thermal Expansion Coefficient and Isobaric Molar Heat Capacity

Isobaric thermal expansivity coefficient α_p_ and isobaric molar heat capacity *C*_*p*,m_ were calculated with the help of the GROMACS *energy* module,^34,35^ which uses the fluctuations in energy quantities to evaluate α_p_ and *C*_*p*,m_ according to
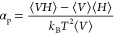
8and
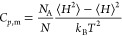
9Where *V* is the system volume, *H* is the enthalpy, *k*_B_ is the Boltzmann constant, *T* is the absolute temperature, *N*_A_ is Avogadro’s number, *N* is the number of molecules in the system, and ⟨···⟩ refers to a time average. Repeated analysis from independent simulation runs on the same system showed that random error in the analysis results are quite high, up to 20 % RSD for α_p_ and up to 10 % RSD for *C*_*p*,m_.

## 3 Results and Discussion

### 3.1 Initial Survey of Available Force Fields by Simulating Diethylene Glycol

Our initial set of force fields tested included OPLS, GROMOS, and CHARMM, and the results are summarized in Table 1. While the CHARMM force field reproduced experimental densities the closest, it produced the largest deviations from experimental data for the self-diffusion coefficients. The reverse is true for the GROMOS force field. The simulation results from the OPLS force field in Table 1 appear to represent the best compromise of achieving reasonable agreement with both experimental properties density and self-diffusion coefficient. Thus, the OPLS force field was chosen for further simulations. We changed the scaling of the Coulomb and van der Waals interactions for the 1–4 atom pairs of the OPLS force field by choosing different values for the parameters *f*_LJ_ and *f*_qq_ to achieve an improved agreement of the density temperature dependence with the experimental data. However, as can be seen from Table 1, the improved density agreement resulted in a worsened agreement with experimental data for the self-diffusion coefficient. Thus, the standard values of 0.5 each for *f*_LJ_ and *f*_qq_ were kept for all other simulations using the OPLS force field presented in this report.

**Table 1:** Comparison of Simulated Physical Properties of Diethylene Glycol with Experimental Values

*T*/K	Exp.^26^	OPLS^a^	OPLS^b^	GROMOS	CHARMM
Densities in kg·m^–3^					
298	1113.2	1108.3	1106.8	1065.6	1112.2
308	1106.0	1098.4	1099.2	1057.7	1103.1
318	1098.7	1087.4	1092.6	1050.6	1094.5
328	1091.4	1078.7	1083.9	1043.7	1086.6
338	1084.0	1067.0	1074.4	1035.2	1078.2
348	1076.5	1056.8	1065.9	1027.4	1068.3
358	1068.9	1045.9	1056.5	1019.2	1058.9
Self-Diffusion Coefficient in 10^–11^ m^2^·s^–1^^c^					
298	5.70	2.37	1.069	2.50	1.23
308	8.27	4.09	1.690	4.52	2.53
318	12.48	7.65	3.124	7.03	4.48
328	17.15	11.28	5.574	12.36	7.55
338	23.32	16.85	8.816	17.53	10.52
348	30.47	24.65	12.78	23.77	16.05
358	40.53	33.55	18.37	35.26	23.02

a *f*_LJ_ = 0.5 and *f*_qq_ = 0.5.

b *f*_LJ_ = 0.2 and *f*_qq_ = 0.75.

c Values are uncorrected for size effects, which would by estimation lead to a 10–15% increase.

### 3.2 Simulation of Ethylene Glycol Oligomers with the OPLS Force Field

As a next step, the OPLS force field was further tested by a series of simulations on ethylene glycol oligomers. Included in the analysis of these tests are comparisons of experimental viscosities with viscosities obtained from MD simulations using the finite-size effects of self-diffusivity introduced by Jamali et al.,^58^ as outlined in Section 2.3.3. The simulation results are summarized in Table 2. While deviations of OPLS simulated densities from experimental values slightly increase with increasing oligomer size, the simulated self-diffusion coefficients and viscosities become drastically different from the experimental values, more than an order of magnitude for pentaethylene glycol.

**Table 2:** Comparison with Experimental Values of Physical Properties at *T* = 328 K of Ethylene Glycol Oligomers, HO(CH_2_CH_2_O)_*n*_H, Simulated with the OPLS Force Field

*N*	*n* = 2	*n* = 3	*n* = 4	*n* = 5	*n* = 6	*n* = 7
Densities in kg·m^–3^						
exp.^18^	1091.4	1091.7	1096.0	1097.4	1099.3	1097.0
250	1077.5	1089.3	1103.4	1106.6	1113.5	1115.4
1000	1077.4	1089.1	1103.0	1106.1		
Self-Diffusion Coefficient, *D*, in 10^–11^ m^2^·s^–1^						
exp.^18^	16.8	12.5	10.0	8.6	6.6	5.7
250	12.7	4.48	1.56	0.86	0.47	0.29
1000	13.0	4.58	1.53	0.84		
D-based^a^	13.5	4.73	1.48	0.80		
Viscosities, η, in mPa·s						
exp.^18^	9.25	11.2	13.2	15.8	18.3	20.6
250	11.1	27.3	65.5	123.7	226	315
1000	11.2	26.5	65.1	149.7		
D-based^a^	7.6	20.4	100.1	142.5		
*D*η (10^–14^ N)						
exp.^18^	155	140	132	135	120.8	117.42
250	125	106	88	93	95	81.9
1000	133	111	92	120		
D-based^a^	103	97	148	115		

a Obtained from finite size effects on self-diffusion coefficient following Jamali et al.^58^

As detailed in Section 2.3.3, it became apparent that the finite size-based method to obtain viscosities becomes impractical for simulations of higher oligomers. Hence, other methods of extracting viscosities from the simulation data were attempted, where the time decomposition method as described in Section 2.3.3 proved to be the most efficient and reliable. Table 2 includes OPLS viscosities obtained by both analysis methods, time decomposition and the finite size method by Jamali et al.^58^ The viscosity values for di-, tri-, and pentaethylene glycol obtained by the finite size method differ by up to 35% from the viscosities obtained from the time decomposition method, with the largest discrepancy in the case of tetraethylene glycol. In this regard, it is helpful to also inspect in Table 2 the product of viscosity, η, and self-diffusion coefficient, *D*. According to the Stokes–Einstein relation, in the rearranged form presented in eq 10, where *k*_B_ is the Boltzmann constant, and *c* is a constant with values typically ranging between 4 for the slip boundary conditions and 6 for the stick boundary conditions,^61^*D*η should decrease slightly at a constant temperature, *T*, with increasing ethylene glycol oligomer reflecting the increase in hydrodynamic radius, *r*.
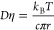
10

This is indeed the case for the experimental data listed in Table 2. It is interesting that the simulated *D*η-values are overall in reasonable agreement with the experimental ones and thus also show a slight overall decrease with increasing ethylene glycol oligomer size. In other words, the large overestimate of the simulated viscosities negates the large underestimate of the simulated diffusion coefficients. For tetraethylene glycol, one can see that the *D*η-value is underestimated using the viscosities obtained from the time decomposition method but overestimated using the viscosities from the finite size method indicating that per chance both methods resulted in inaccuracies of similar proportion but in the opposite direction.

### 3.3 Further Survey of Available Force Fields by Simulating Tetraethylene Glycol

Because of the increasingly poor performance of the MD simulations using the OPLS force field to reproduce experimental data for the dynamic properties of higher oligomers, we went back to testing the performance of other force fields for simulating tetraethylene glycol. We included two more force fields that we were able to find in the literature at that point, namely the Martini force field^19^ and a specific force field from the Müller–Plathe group (CZMP).^20^ These force field test results are summarized in Table 3. It can be seen in Table 3 that the GROMOS, CHARMM, and AMBER force fields also show simulated properties that differ from experimental values, as was the case for diethylene glycol in Table 1. The simulations with the GROMOS force fields produced dynamic properties that are somewhat more accurate than those obtained from the OPLS force field, but the reverse is true for the density. It can also be seen in Table 3 that there is a counterbalance between accurately simulating the density as a static property versus the dynamic properties. Simulations resulting in more accurate densities result in more inaccurate dynamic properties and vice versa. Specifically, as simulated densities decrease in Table 3, viscosities decrease, and self-diffusion coefficients increase. Simulations with the Martini and CZMP force fields produced simulated properties that were unacceptably far off from experimental values. In summary, the performance of the tested force-fields of simulating density, viscosity, and self-diffusion coefficients was not significantly better than the OPLS force field, and we decided to continue working with the OPLS force field, where, as described in the next subsection, we attempted various modifications of the OPLS force field to improve its performance in reproducing experimental properties.

**Table 3:** Comparing Experimental with Simulated Densities, Viscosities, and Self-Diffusion Coefficients at *T* = 328 K of Tetraethylene Glycol, HO(CH_2_CH_2_O)_4_H, Using Several Different Force Fields with 1000 Molecules

force field	densities (kg·m^–3^)	self-diffusion coefficient, *D* (10^–11^ m^2^·s^–1^)	viscosity, η (mPa·s)	*D*η (10^–14^N)
exp.^26^	1096.0	10.0	13.2	132
GROMOS	1034.2	5.9	23.9	130
CHARMM	1084.9	2.7	50.4	125
AMBER	1141.8	1.4	83.8	108
CZMP	883.7	35.1	3.1	94
Martini^a^	951.6	86.9	2.4	201

a No scaling factor was applied.

### 3.4 Testing Modifications to the OPLS Force Field

It is understood that parameter values of a force field may be interdependent, so changing a single parameter in isolation could be problematic.^18^ Nevertheless, this was attempted here to gain insights into which aspects of the force field may require particular attention in future force field developments in order to improve agreement between simulated and experimental properties. In this regard, it is interesting that the self-diffusion coefficients obtained from the simulations of diethylene glycol are reasonable but become increasingly smaller compared to experimental values with higher ethylene glycol oligomers. The number of ethers increases with increasing oligomer chain length, but the number of hydroxy end groups remains constant at two. Therefore, the effects of the hydroxy end groups on the dynamic properties should diminish with increasing oligomer chain length while that of the ether oxygen increases. Thus, we tested for tetraethylene glycol if perhaps the balance of negative charge between the hydroxy and ether oxygen atoms requires adjustment. To systematically inspect trends, the negative charge of the hydroxy oxygen atom, normally at −0.6888 for the OPLS force field, was varied between −0.3888 and −0.9888 in 0.2 increments with concurrent adjustment of the ether oxygen charge accordingly to maintain zero charges overall for the tetraethylene glycol molecule. These charge adjustments all resulted in a worse agreement with literature values as densities increased and self-diffusion coefficients decreased. This unfavorable outcome was confirmed also for the GROMOS force field, where simulations with similar adjusted force fields were also made for tetraethylene glycol. Apparently, the OPLS and GROMOS force fields generally do properly balance the negative charges between hydroxy and ether oxygen atoms.

Next, we tested the effect of the charge balance on the hydroxy group itself (see Figure 1a). We found that making the hydroxy group in tetraethylene glycol less polar by decreasing the magnitude of both, the positive hydroxy hydrogen charge and the negative hydroxy oxygen charge, lowers the simulated density, and increases the simulated self-diffusion coefficient. This trend is expected because a lower polarity of the hydroxy group will decrease the strength of intermolecular interactions. Consequently, adjusting the hydroxy group polarity can only be done to a limited extend before densities become too low compared to experimental densities. Therefore, other aspects of the force field may need adjustments as well. Perusal of the OPLS force field parameters revealed that the parameters for the potential function associated with the proper dihedral angles showed large value entries of about 9 kJ·mol^–1^ in magnitude for the (HO)–C–C–O proper dihedral (see Figure 1b for the definition of (HO)–C–C–O proper dihedral), which is 5–10 times higher than the other proper dihedral parameters in the OPLS force field of tetraethylene glycol. Such a large value would restrict the C–C bond rotation of the terminal (HO)–C–C–O dihedral. A comparison of the (HO)–C–C–O dihedral potential function of the various force field (see Figure S1 in the Supporting Information) shows that these differ substantially in both shape and magnitude, which illustrates that it is presently generally unclear, which parameters should be used to best describe the (HO)–C–C–O dihedral potential function. The relation between dihedral motions and segmental motions of polymers was studied in some detail in previous simulation studies.^62−64^ Some of these analyses also scaled the heights of the dihedral potentials. Generally, the dihedral motions show Arrhenius temperature dependence. Thus, upon cooling, they decouple from the segmental motion, which is associated with viscosity-related structural relaxation and shows super-Arrhenius behavior. Furthermore, the dihedral motions were found to be of a rather complex nature. For example, they involve extended correlated forward-backward jump sequences, which reflect the fact that the orientation of a given chain segment largely depends on the momentary configuration of the adjoining chain segments. The exact relation between dihedral motions and segmental motions is still elusive, but outside the scope of this manuscript. Nevertheless, we inspected the effect of reducing the (HO)–C–C–O proper dihedral parameter value of 9 kJ·mol^–1^ to 1/2 or 1/4 of its value. We observed that the simulated self-diffusion coefficients increased while lowering the density only very slightly. We also checked the effects of modifying the potential function parameters for the H–O–C–C and the O–C–C–O dihedrals. Simulated densities and self-diffusion coefficients were observed to be very insensitive to these adjustments and stayed at essentially the same values as for the unchanged OPLS force field, even when the parameters were set to zero.

Finally, we investigated combinations of adjustments with respect to (HO)–C–C–O dihedral potential parameters and the polarity of the hydroxy group. These simulation tests were done for each di- to heptaethylene glycol oligomer using 250 molecules. As can be seen in Table 4, where the resulting densities, self-diffusion coefficients as well as viscosities are summarized, different combinations of adjustments need to be made for different ethylene glycol oligomers to achieve as close agreement with experimental values as possible. It can also be seen in Table 4 that the adjustments to the force field are still insufficient to achieve good agreement with experimental data for the higher oligomers. We note that not just the OPLS force field but all the other general force fields tested here have essentially the same force field parameters regardless of ethylene glycol oligomer. It might be worth investigating with quantum mechanical methods if each ethylene glycol oligomer requires its own unique force field. As one specific example, the charges of the ether oxygen may differ in higher oligomers and not be identical. However, developing new force-fields, one for each ethylene glycol oligomer, goes beyond the scope of this report. Instead, we chose to investigate the qualitative question if ethylene glycol oligomers are distributed evenly in polydisperse PEG200 by dual sets of MD simulations, one set with the original OPLS force fields and one set with the modified OPLS force fields, where we took the parameters for the proper dihedral potential and the hydroxy charge distribution as shown for the columns in Table 4, where simulated results agreed best with experimental measurements as highlighted in green. These parameters were chosen as a compromise for improving the agreement of dynamic properties with experimental values while maintaining reasonable agreement of the densities with experimental values. The next subsection discusses the simulation results for PEG200.

**Table 4:**
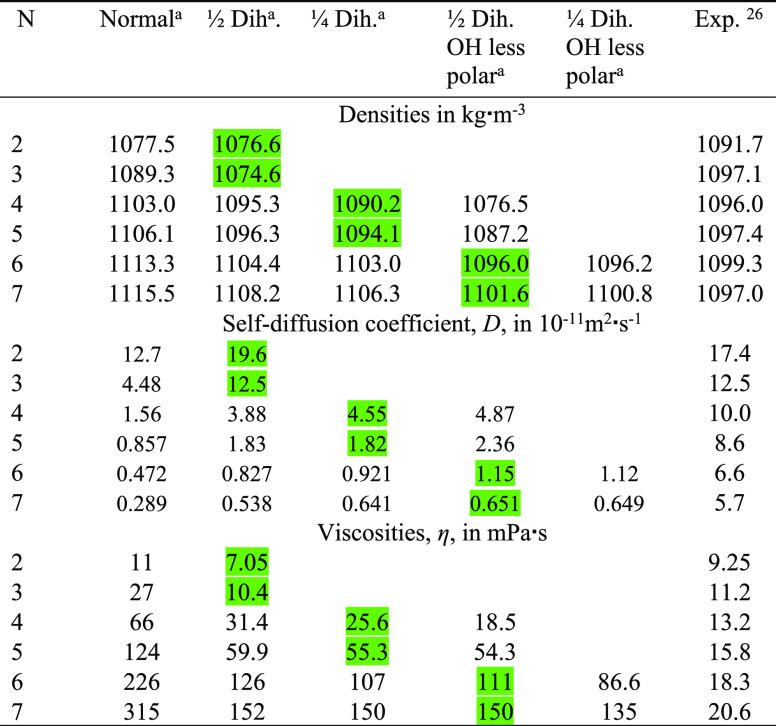
Comparing Experimental with Simulated Densities, Viscosities, and Self-Diffusion Coefficients at *T* = 328 K of Ethylene Glycol Oligomers, HO(CH_2_CH_2_O)_*n*_H from Simulations with the OPLS Force Field with Altered Dihedral Angle Potentials and Partial Charges on the Hydroxy Groups^a^

a Green labeled fields indicate the best compromise agreement with experimental data for all three properties and the choice of modified force field for simulating PEG200.

b OPLS force field parameters and their modifications (changing proper (HO)–C–C–O dihedral potential to 1/2 or 1/4 of the original value and reducing the magnitude of charges on proton and oxygen of the hydroxy end groups by 0.1 units) are listed in the Supporting Information.

### 3.5 Simulation of PEG200

**Figure 2:**
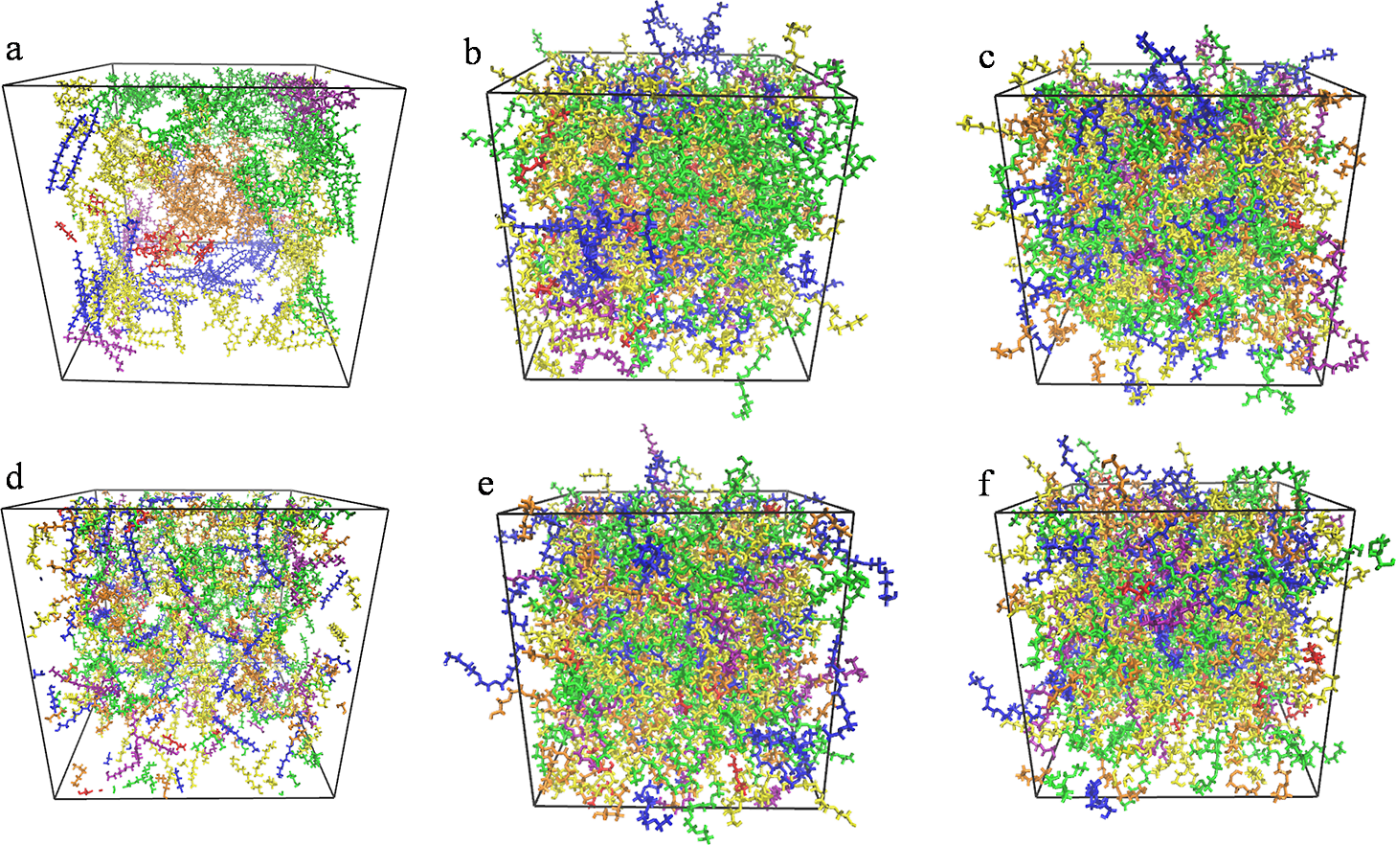
Snapshots from MD simulations of PEG200 using the OPLS force field with clustered starting position (a–c) and random starting position (d–f) obtained after energy minimization (a,d), *NPT* equilibration (b,e), and *NVT* production run (c,f).

Two sets of four different PEG200 simulations were conducted, one set using the OPLS force field without any modifications and one set using the modified force field (see Table 4). The oligomer mole fractions reported from GC analysis of commercial PEG200 were used.^25^ Each set of simulations consisted of (a) 1000, (b) 500 total molecules randomly placed into the simulation box as the starting configuration, (c) 500 molecules placed in clusters of the same oligomers into the simulation box, (d) 500 molecules of a binary mixture randomly placed in the simulation box consisting of 0.620 mol fraction tri- and 0.380 mol fraction hexaethylene glycol, which is a composition reported elsewhere^25^ to obtain 200 g·mol^–1^ as the average molar weight. The two different sets of four simulations allowed for testing the effects of the different force field parameters on the simulation results. Simulations with 1000 versus 500 molecules were conducted to verify the absence of significant box size effects on simulation results. The simulations with different starting configurations, randomly placed molecules versus placement of molecules in clusters, were conducted to confirm that the chosen simulation length was sufficient to reach equilibrium with the absence of any slow dynamic processes. The simulations of the binary tri- and hexaethylene glycol mixtures were inspired by the recent finding that such mixture displays the same densities, viscosities, and self-diffusion coefficient as polydisperse PEG200.^25^ With respect to the starting configurations, the six-panels in Figure 2 show a side-by-side comparison of random versus clustered starting configuration through the progression of simulation stages. The clustering appears to have vanished after the equilibration stage as will be further confirmed in the next subsection where it can be seen that simulation results are indistinguishable from those that started with random configuration. This stayed true for both simulations, using the unmodified and the modified OPLS force field. The absence of aggregation of oligomers of the same kind is further confirmed by the oligomer–oligomer RDFs shown in Section 6 of the Supporting Information. If there were clusters of each oligomer present, then the likelihood of an oligomer being surrounded by the same oligomer would have to be significantly higher than the likelihood to be surrounded by any of the other oligomers. This is not indicated in the oligomer–oligomer RDF. Thus, the simulations are supportive of thinking of PEG200 as a homogeneous mixture of ethylene glycol oligomers without the presence of any oligomer domains.

#### 3.5.1 Bulk Physical Properties

Table 5 compares the physical properties obtained from the various MD simulations of PEG200 with experimental results reported in the literature. Included in Table 5 are also the thermal expansion coefficient and the isobaric molar heat capacity. As noted in Section 3.4, the modifications to the OPLS force field lead to slightly smaller densities, increased self-diffusion coefficients, and decreased viscosities. However, self-diffusion coefficients and viscosities are still off from experimental values by a factor of about 2.5. These simulated properties are reproducible within uncertainty regardless of simulating with 500 or 1000 molecules or starting from random or clustered starting configuration of molecules. As also observed experimentally,^25^ the simulated binary mixture shows quite similar values for these bulk physical properties as for the simulated PEG200. As for the thermodynamic properties of thermal expansion coefficient and molar heat capacity, these do not seem to be affected significantly by the modifications to the OPLS force field. The simulated isobaric thermal expansion coefficients are in reasonable agreement with the experimental values, while the simulated isobaric molar heat capacities are approximately a factor of two larger than those reported in the literature.

**Table 5:** Comparison of Simulated and Experimental Physical Properties for PEG200 at 328 K

simulation details^a^	densities (kg·m^–3^)	self-diffusion coefficient, (10^–11^ m^2^·s^–1^)	viscosities, (mPa·s)	isobaric thermal expansion coefficient, (1000 K^–1^)	isobaric molar heat capacity, (J·mol^–1^·K^–1^)
Simulations with Random Starting Position					
OPLS (1000)	1103.5	1.28	84.4	0.932	917
OPLS	1104.7	1.25	76.5	0.864	889
Mod. OPLS (1000)	1090.3	3.32	32.2	0.909	901
Mod. OPLS	1090.1	3.35	27.3	0.971	934
Simulations with Clustered Starting Positions					
OPLS	1104.3	1.25	80.9	0.810	867
Mod. OPLS	1090.3	3.24	35.9	0.954	919
exp.^25,65,66^	1097.0	8.2	14.7	0.727	431^b^
Simulations of Binary Mixture of Tri and Hexaethylene Glycol, Random Starting Position					
OPLS	1102.1	0.48	84.1	0.675	792
Mod. OPLS	1085.2	3.93	31.0	0.833	824
exp.^25^	1097.0	8.4	14.7	0.729	

a The number 1000 in parenthesis indicates the number of molecules simulated. All other simulations listed here were carried out with 500 molecules.

b Extrapolated from a second-order polynomial fit to the literature data at lower temperatures.

#### 3.5.2 Hydrogen Bonding

**Figure 3:**
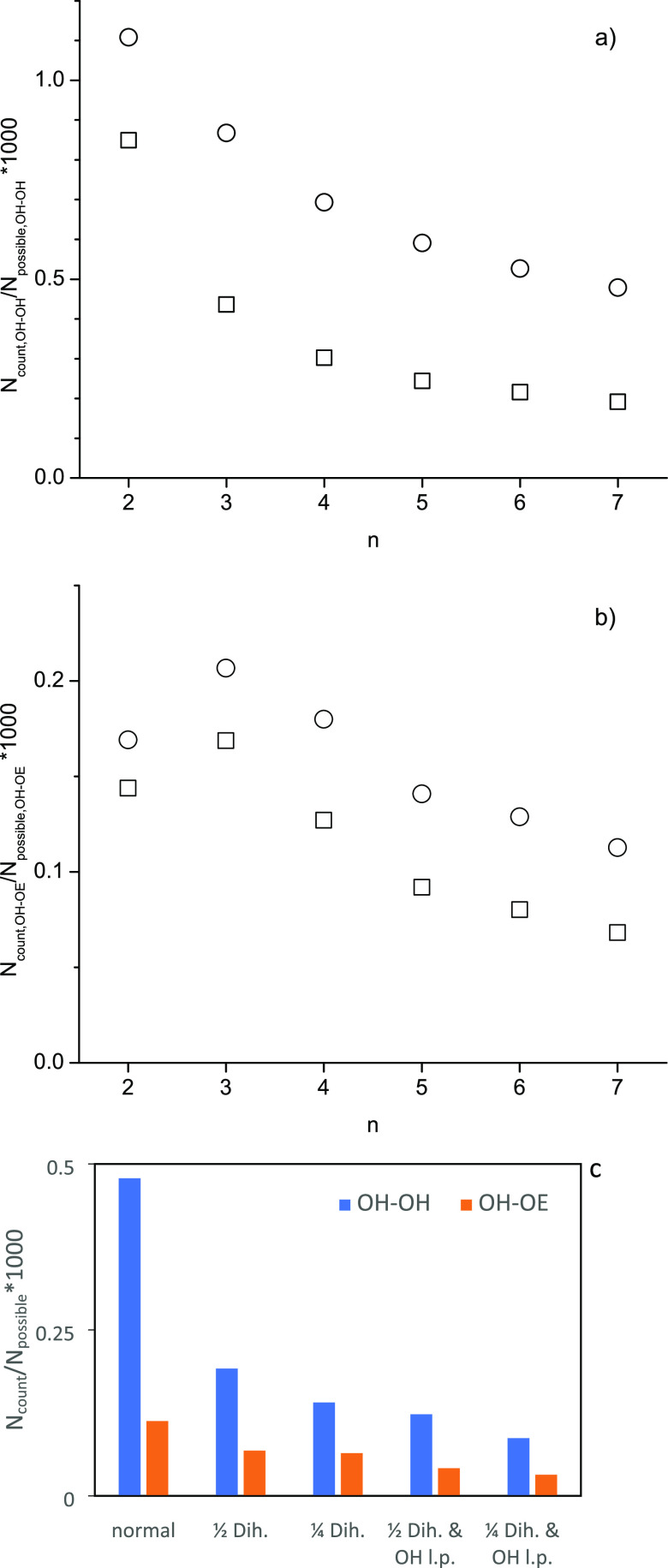
Effects on (a) OH–OH and (b) OH–OE hydrogen bonding from modifying the OPLS force field (unmodified: open circles, modified, and open squares) for MD simulations of 250 H-[O-CH_2_-CH_2_]_*n*_-OH oligomers by using 1/2 the value of the (HO)–C–C–O dihedral potential energy and (c) of all OPLS force field modifications tested (see Table 4) on the hydrogen bonding of heptaethylene glycol (*n* = 7): changing proper (HO)–C–C–O dihedral potential to 1/2 (“1/2 Dih.”) or 1/4 (“1/4 Dih.”) of the original value and lowering the polarity of the OH functional group by reducing the magnitude of charges on the hydrogen and oxygen atoms (“1/2 Dih. OH l.p.” and “1/4 Dih. OH l.p.”).

Hydrogen bonding interactions can be expected to be an essential component of the intermolecular interactions in PEGs. Therefore, it is necessary to inspect how the modifications of the OPLS force field discussed in Section 3.4 affect the hydrogen bonding in simulated PEG200 to be able to explain the resulting physical properties summarized in Table 5. For this purpose, it is helpful to first inspect the effects of the force field modification on the hydrogen bonding of each neat ethylene glycol oligomer. (The reader is referred to Section 2.3.5 for the details on comparing hydrogen bonding numbers adjusted by the number of possible hydrogen bonds). Figure 3a,b illustrates that reducing the (HO)–C–C–O dihedral potential energy to half of the original OPLS force field values universally lowers hydrogen bonding for all of the ethylene glycol oligomers and for both OH–OH and OH–OE hydrogen bonding. This observation may at first seem counterintuitive because a hydroxy group that can more freely rotate around the carbon–carbon bond of the (HO)–C–C–O dihedral should more easily be able to adjust to the angle needed to engage in intra- or intermolecular hydrogen bonding. However, once a hydrogen bond is formed, the lower (HO)–C–C–O potential energy also makes it easier for the hydroxy group to escape the hydrogen bonding interaction. Apparently, the increased ability to break a hydrogen bond outweighs the increased ability to form a hydrogen bond. Additional adjustments as listed in the column labels in Table 4 further reduce hydrogen bonding, as illustrated in Figure 3c for the case of heptaethylene glycol. As stated in the introduction, many force fields have been optimized for aqueous solutions. Given that water is a strong hydrogen bond former, this might have resulted in structural force field parameters that lead to an overestimation of hydrogen bonding in the neat ethylene glycol oligomers. Finally, it appears that hydrogen bonding interactions are diminished when increasing ethylene oxide homologue significantly more than captured by any of the investigated force fields, including the OPLS force field with the most extreme adjustments (see Table 4).

We now turn to the hydrogen bonding in PEG200 where, as stated in more detail in Section 2.3.5, more combinations of hydrogen bonding partners need to be considered. Section 7 in the Supporting Information summarizes the number of OH–OH and OH–OE hydrogen bonds of each oligomer to its own kind (intra and inter) and then to all other oligomers but itself. These tables also include values for each specific kind of hydrogen bond in relation to (a) the number of the respective oligomer present in the simulation box and (b) the number of possible hydrogen bonds of that specific kind (see Section 2.3.5 for further details on the number of possible hydrogen bonds). The latter is more meaningful for comparison of the hydrogen bonding behavior across the oligomers because the number of ether oxygen atoms increases with increasing oligomer providing more possible combinations of hydrogen bonds between hydroxy hydrogens and ether oxygens.

**Figure 4:**
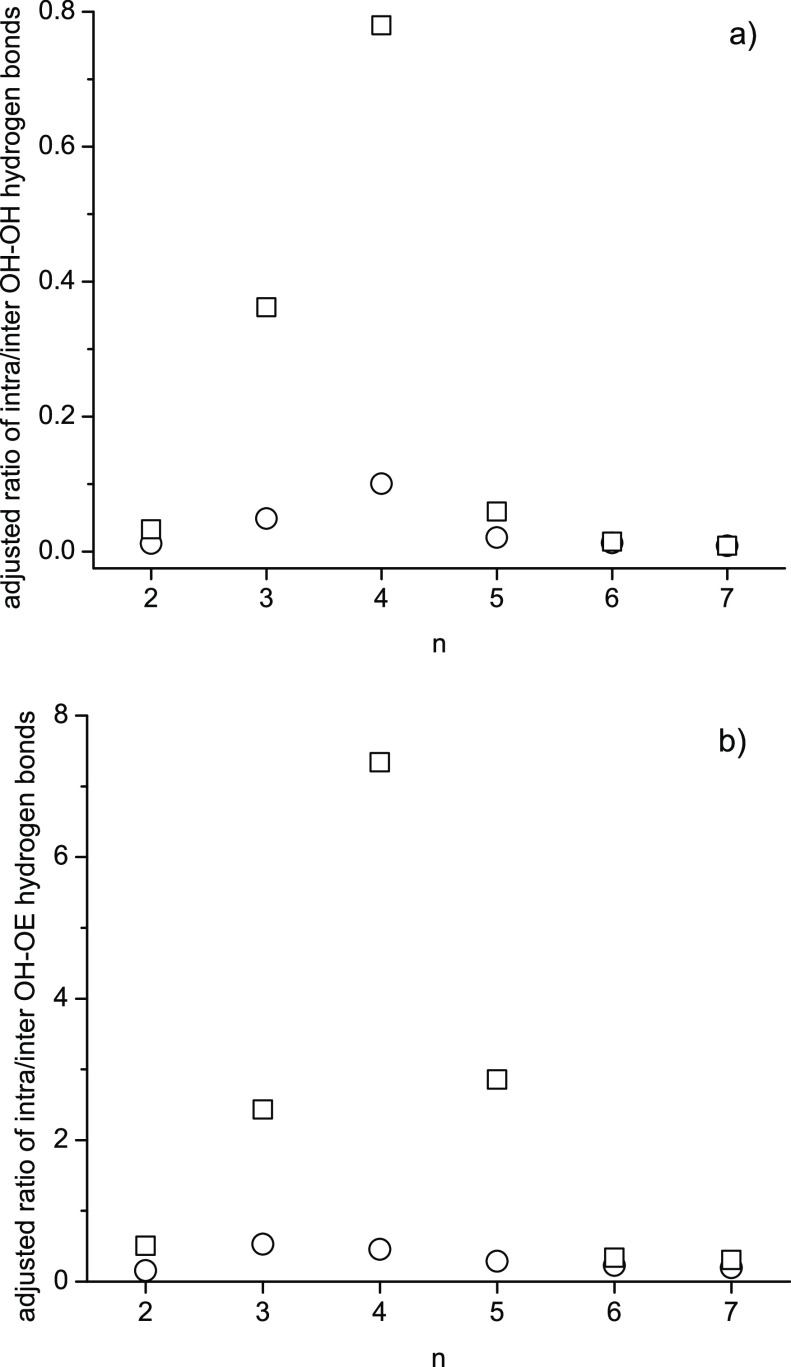
Adjusted ratio of intra- over intermolecular hydrogen bonds between hydroxy hydrogen and (a) hydroxy oxygen and (b) ether oxygen for each oligomer with itself in PEG200 obtained from MD simulations at 328 K using the OPLS force field (circles) and modified (see Table 4) OPLS force field (squares). The ratio numbers were adjusted by the number of possible intra- and intermolecular hydrogen bonds, the respective oligomer mole fraction, and a scaling factor depending on the number of oligomer components as discussed in the text.

Figure 4 compares the adjusted oligomer to the same oligomer hydrogen bond numbers between intra- and intermolecular hydrogen bonds [(*N*_count_,_intra_/*N*_possible,intra_)/(*N*_count_,_inter_/*N*_possible,inter_)*(*N*_total_/*N*_*n*_)/*f*)], where *N*_total_/*N*_*n*_ accounts for the different oligomer mole fractions in the PEG200 mixture, and *f* adjusts for the different dependence on the number of respective H-[O-CH_2_-CH_2_]_*n*_-OH oligomer molecules in the simulation box, as explained in Section 2.3.5 [*f* = 2(*N*_*n*_ – 1) for OH–OH hydrogen bonding and *f* = *N*_*n*_ – 1 for OH–OE hydrogen bonding]. Shown in Figure 4 are only the results from the simulations with 1000 molecules total to reduce clutter. The hydrogen bonding results from the other simulations (500 molecules total, clustered starting configuration) are essentially the same except for the diethylene glycol component, where values differ substantially, likely caused by the small statistics given that diethylene glycol is the least present oligomer component in PEG200. Similar results as displayed in Figure 4 were also obtained when doing the same hydrogen bonding analysis of the neat oligomer runs (not shown).

Keeping in mind that the modifications to the OPLS force field reduce hydrogen bonding overall (Figure 3), Figure 4 illustrates that the force field modifications generally shift the remaining hydrogen bonding toward intramolecular hydrogen bonding at the cost of intermolecular hydrogen bonding. The unmodified OPLS force field results in a preference for intermolecular OH–OH hydrogen bonding (Figure 4a) as the values are all <1. The force field modifications show hardly an effect in Figure 4a for hexa- and heptaethylene glycol and only a small shift toward intramolecular hydrogen bonding for di- and pentaethylene glycol. Much stronger shifts toward intramolecular hydrogen bonding are observed for tri- and tetraethylene glycol. The tendency toward intramolecular hydrogen bonding is also strongest for tetraethylene glycol in the case of OH–OE hydrogen bonding interactions, as can be seen in Figure 4b. While intermolecular OH–OE hydrogen bonding dominates here too for the unmodified OPLS force field, its dominance is less strong than observed for OH–OH hydrogen bonding in Figure 4a (note the 10 times larger *y*-scale in Figure 4b compared to Figure 4a), and the shift toward intramolecular hydrogen bonding for the modified OPLS force field is more pronounced as well. Intramolecular OH–OE hydrogen bonding becomes even the preferred mode for tri-, tetra-, and pentaethylene glycol since their values in Figure 4b are >1.

**Figure 5:**
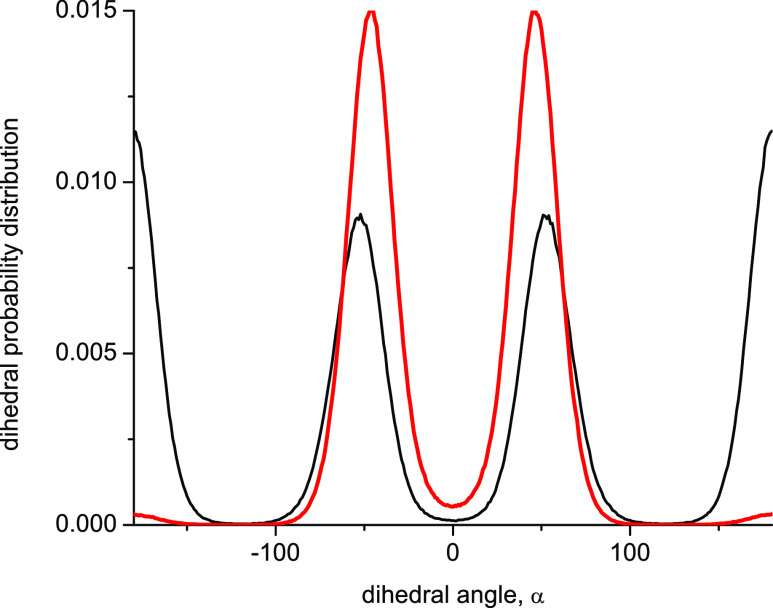
Probability distribution of dihedral (HO)–C–C–O angles (see scheme 1) in tetraethylene glycol obtained from MD simulation of PEG200 at 328 K using the OPLS force field (black thin line) and the modified OPLS force field according to Table 4 (red thick line).

Intramolecular hydrogen bonding leads to a spatial configuration of the hydroxy group that is gauche if not cis relative to the nearest ether oxygen. Thus, a shift from inter- to intramolecular hydrogen bonding should be reflected in corresponding changes in the angle distribution of the (HO)–C–C–O dihedral. Exemplary for the case of tetraethylene glycol, this is indeed confirmed in Figure 5, which shows that the likelihood of finding the (HO)–C–C–O dihedral in anti-configuration is greatly diminished under the modified OPLS force field.

**Figure 6:**
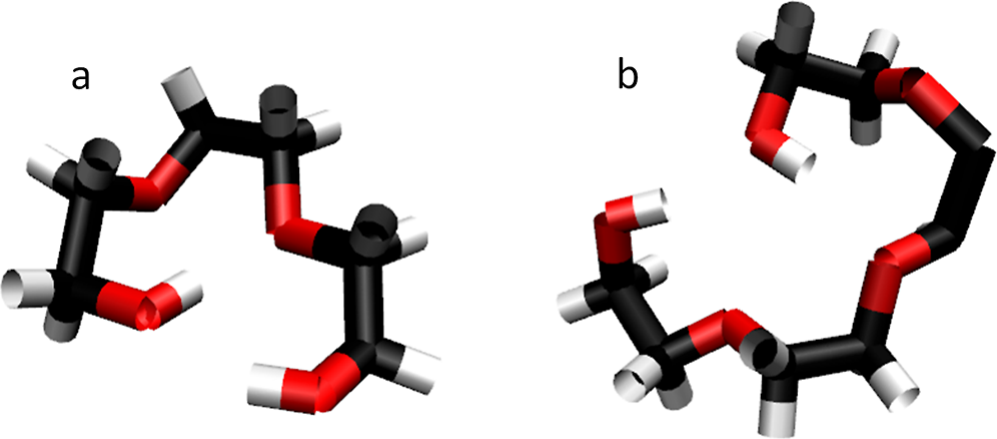
Snapshot showing a selected triethylene glycol (a) and tetraethylene glycol (b) oligomer obtained from an MD simulation of PEG200 at 328 K simulation with the unmodified OPLS force field. These typical structural configurations were more dominant in simulations with the modified OPLS force field.

From Figure 4 it seems that the tendency of tetraethylene glycol toward intramolecular hydrogen bonding is extraordinary. Even for the unmodified OPLS force field, a heightened intramolecular OH–OH hydrogen bonding compared to the other oligomers is indicated in Figure 4a. Inspection of simulation snapshots shows intermolecular interactions between the hydroxy hydrogen and the nearest intramolecular ether oxygen to form five-membered cyclic rings that are frequently present. Tetraethylene glycol and, to a lesser extent, triethylene glycol show these ring structures for both terminal hydroxy groups. Moreover, some of the tetraethylene glycol form ring structures where the terminal hydroxy groups’ hydrogen bond with each other while their hydroxy hydrogen atoms maintain close proximity to intramolecular ether oxygens as shown exemplarily in Figure 6. We note that while these intramolecular OH–OE interactions bring the hydroxy hydrogen atoms into proximity with the ether atoms, their angular orientation may not fulfill the ≤30° requirement to qualify as a hydrogen bond. From inspecting the simulation snapshots, such ring structures are somewhat present also in triethylene glycol but essentially absent for the higher oligomers. It appears that the length of the tetraethylene glycol molecule is optimal to form these ring structures, while still maintaining intramolecular interactions between the terminal hydroxy hydrogen and nearest ether oxygen atoms. This peculiarity might explain the large tendency toward intramolecular hydrogen bonding of tetraethylene glycol displayed in Figure 4. A similar propensity for forming 5-member cyclic rings through intramolecular interactions was also observed for diols in an experimental infrared spectroscopy study.^67^

Finally, Figure S4 in the Supporting Information inspects the hydrogen bonding interactions of each oligomer with all of the other oligomers but themselves. Figure S4 confirms the already described observation in Figure 3, that the force field modifications lead to a general reduction of hydrogen bonding for both types, OH–OH and OH–OE.

#### 3.5.3 Impacts of Changed Hydrogen Bonding Behavior

**Figure 7:**
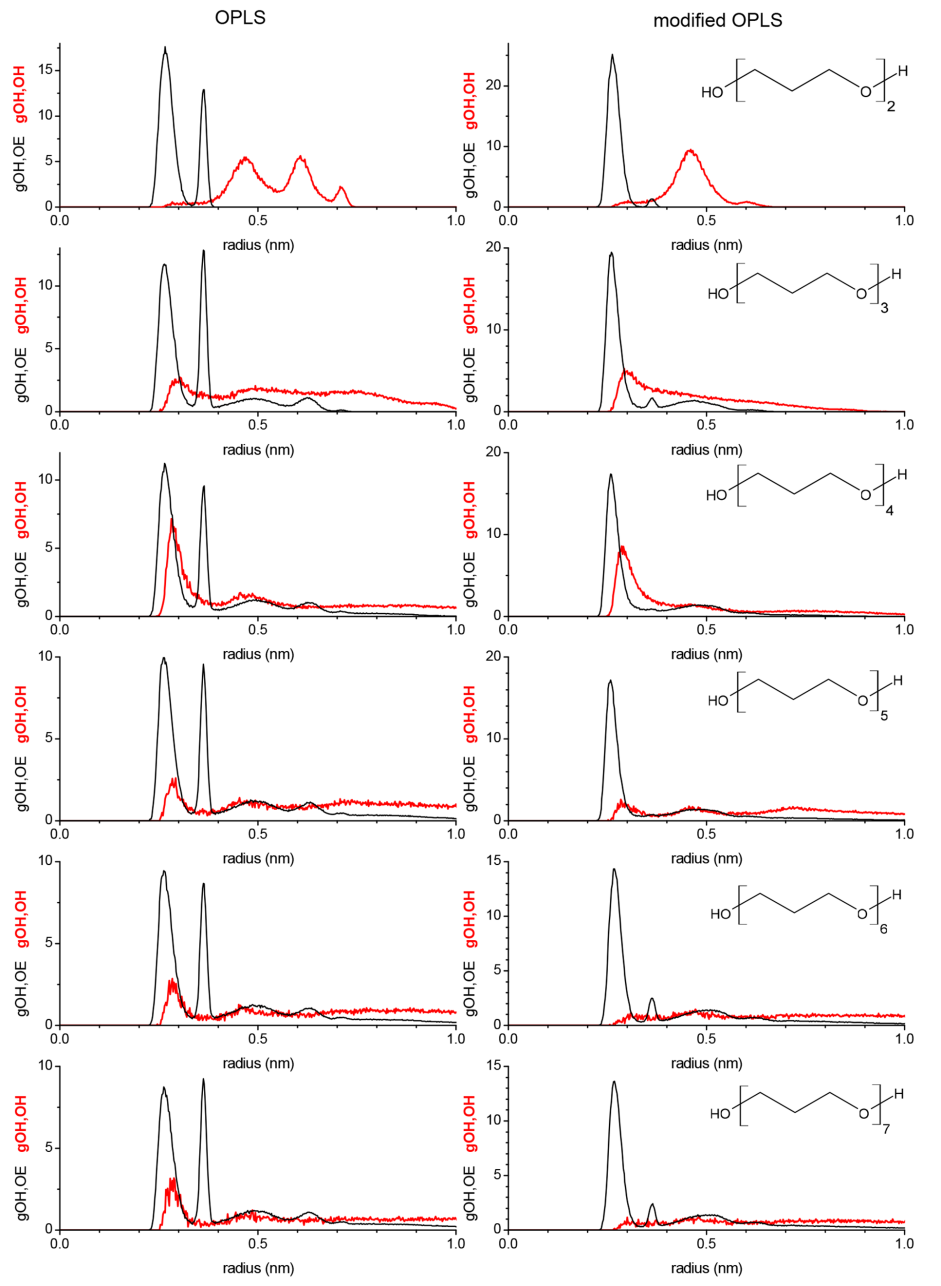
Normalized intramolecular RDFs obtained from simulating PEG200 at 328 K with the OPLS force field (left panel) and the modified (see Table 4) OPLS force field (right panel).

The different hydrogen bonding behavior caused by the modifications to the OPLS forcefield leads to significant changes in the structural configurations of the ethylene glycol oligomers in PEG200, which are readily discernible in the RDFs. We begin with the intramolecular RDFs shown in Figure 7 where the series obtained from the OPLS force field is shown on the left side and the series obtained from the modified OPLS force field is shown on the right side. Each graph takes the perspective of the hydroxy group in how likely it is to encounter another hydroxy oxygen (gOH,OH) and ether oxygen (gOH,OE). All of the gOH,OE graphs, shown in Figure 7, show a very strong peak near 0.25 nm, which corresponds to a gauche (nearly cis) position of the OH group relative to the next nearest ether, which the molecule assumes while undergoing intramolecular OH–OE interactions. In addition, the gOH, OE shows a feature near 0.35 nm that is much reduced for the gOH,OE graphs obtained from the modified OPLS force field where this feature is essentially unobservable for tetra- and pentaethylene glycol. This 0.35 nm peak is for the relative orientation of the hydroxy group with the next nearest ether oxygen when the hydroxy group is not engaged in intramolecular interactions. The observations made in Section 3.5.2 that the modifications to the OPLS force field increase intramolecular (hydrogen bonding-like) interactions explain the drastic reduction of the 0.35 nm peak with a concomitant increase of the 0.25 nm peak in the corresponding gOH,OE graphs in Figure 7 obtained from the modified OPLS force field. Since diethylene glycol only possesses one ether oxygen, the gOH,OE only display the 0.25 and 0.35 nm peaks. Starting with triethylene glycol, two more peaks in the gOH,OE from the unmodified OPLS appear near 0.5 and 0.6 nm, corresponding to the distance from the hydroxy hydrogen to the second ether oxygen away when it is intramolecularly interacting with the nearest ether or not, respectively. The 0.6 nm peak is essentially absent in the gOH,OE graphs obtained from the modified OPLS force field (except perhaps just barely for hexa- and heptaethylene glycol) again because of the increased intramolecular (hydrogen bonding-like) interactions.

As for the intramolecular gOH–OH in Figure 7, there is a peculiar progression from oligomer to higher oligomer in both panels, left and right. The gOH,OH of diethylene glycol obtained from the modified OPLS force field shows just one pronounced peak near 0.45 nm. From inspecting simulation snapshots, this OH–OH distance corresponds to the scenario when both hydroxy groups bend toward the ether oxygen to engage in intramolecular interactions with the ether oxygen. The gOH,OH of diethylene glycol from the unmodified force field shows two additional peaks near 0.6 and albeit weaker near 0.7 nm. These OH–OH distances correspond respectively to the scenarios when only one or neither of the hydroxy groups interacts with the ether oxygen. The absence of the 0.6 and 0.7 nm peaks in the gOH–OH from the modified OPLS is explained by the observation that the modifications significantly increase intramolecular OH–OE (hydrogen bonding-like) interactions. For the particular case of diethylene glycol, there appears to be no significant intramolecular OH–OH hydrogen bonding because this would lead to a peak near 0.3 nm in the gOH,OH, which is absent for both the gOH,OH from unmodified and modified OPLS. Such peak, however, is observed in the gOH,OH for all other oligomers, the strongest for tetraethylene glycol, which is in line with its particular propensity toward intramolecular OH–OH hydrogen bonding noted in Section 3.5.2. Interestingly, the 0.3 nm peak is stronger observed in the gOH,OH of the higher oligomers when obtained from the unmodified OPLS force field compared to the modified OPLS force field where this feature nearly diminishes completely for heptaethylene glycol. This observation suggests that the modifications to the OPLS force field shift the preference of intramolecular interactions from OH–OH to OH–OE for the higher oligomers. The broad feature near 0.5 nm observed in the gOH–OH of tri- to heptaethylene glycol corresponds to the scenario when one hydroxy group interacts with the ether oxygen nearest to the other hydroxy group of the molecule. This feature, however, is washed out in the gOH–OH of tri- and to a lesser extent tetraethylene glycol obtained from the modified OPLS such that there is (almost) no minimum between the 0.3 and 0.45 nm feature. Apparently, the hydroxy groups of tri- and tetraethylene glycol are often at intermittent OH–OH distances as the hydroxy end groups transition between OH–OH and OH–OE intramolecular interactions.

**Figure 8:**
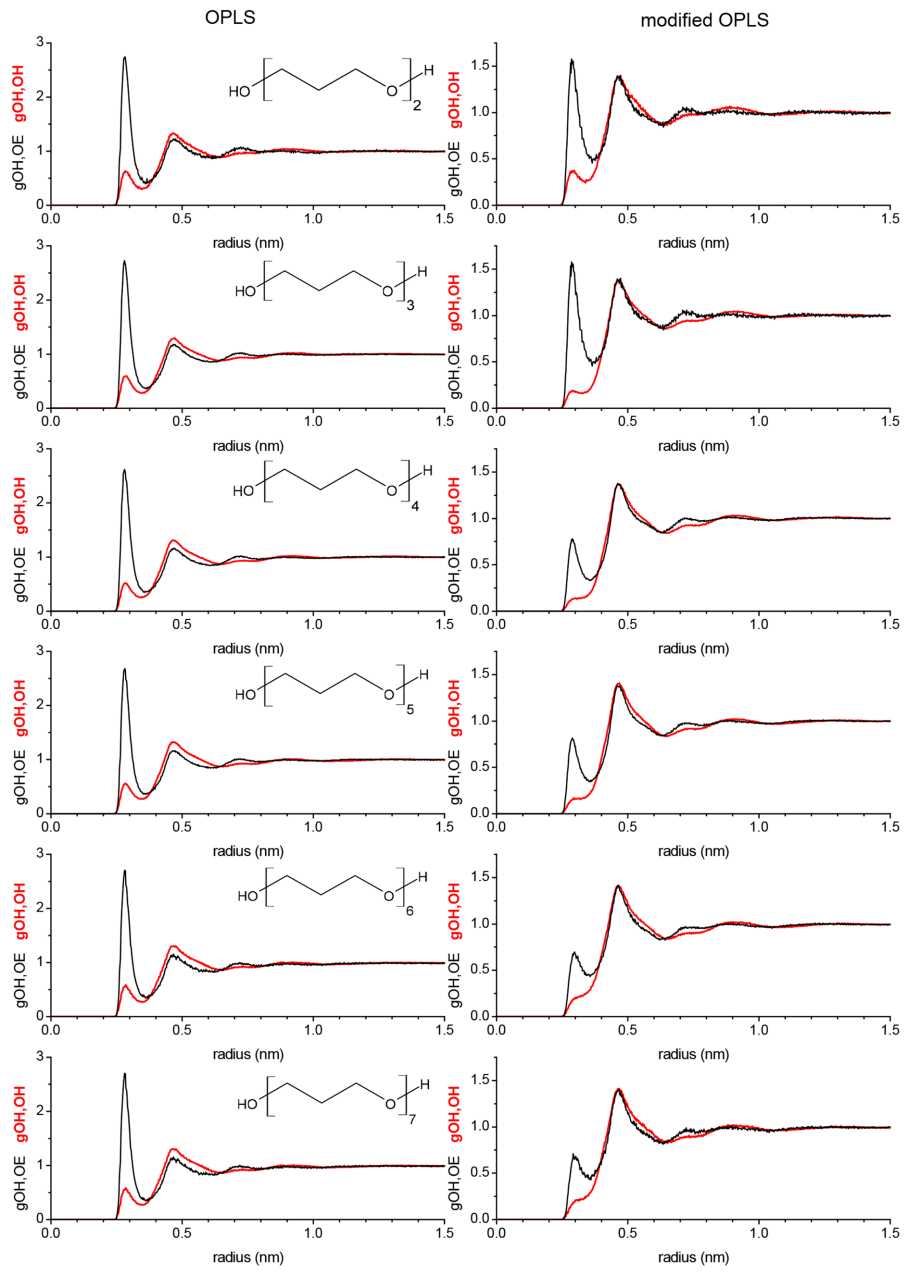
Intermolecular RDFs obtained from simulating PEG200 at 328 K with the OPLS force field (left panels) and the modified (see Table 4) OPLS force field (right panel).

We now turn to the intermolecular RDFs. As can be seen in Figure 8, the intermolecular gOH,OH and gOH,OE of each oligomer are showing the same peaks at the same distances, where only the first peak near 0.25 nm is varying substantially from oligomer to oligomer as well as the choice of force field. The essentially identical peak positions come about from the fact that each oligomer sees on average the same surrounding as other oligomers. If a hydroxy hydrogen of one oligomer is hydrogen bonded to a hydroxy oxygen atom of another oligomer, it does not really matter which oligomer it is, the hydrogen bond distance will essentially be the same. The same is true for an intermolecular hydrogen bond between hydroxy hydrogen and an ether. In fact, the bond distance of an intermolecular OH–OH hydrogen bond is very similar to that of an intermolecular OH–OE hydrogen bond. Both peaks in Figure 8 are near 0.25 nm. Consequently, the distances to the next nearest hydroxy oxygen or ether oxygen are also similar. The peak at 0.25 nm in the intermolecular gOH–OH from the unmodified OPLS force field is approximately similarly strong across all oligomers. The same peak is much reduced in amplitude for the gOH–OH from the modified force field due to the overall decrease in hydrogen bonding and the stronger preference toward intramolecular hydrogen bonding. The loss in intermolecular OH–OH hydrogen bonding appears to be stronger with increasing size of oligomers. Similar can be said for the OH–OE intermolecular hydrogen bonds where the 0.25 nm peak is also much diminished. The shapes of the gOH,OH and gOH,OE at higher than 0.4 nm do not show significant differentiation with respect to ethylene glycol oligomer or force field. In fact, there is even much overlap between gOH,OH and gOH,OE. These portions of the gOH,OH and gOH,OE represent the solvation structure with the peak near 0.5 nm representing the average distance between two oxygens, regardless of hydroxy or ether oxygen, coming in closest contact without engaging into any specific hydrogen bonding. Therefore, the very shallow oscillations in the gOH,OH and gOH,OE indicate that solvation shells beyond the first solvation shell are not very structured, which would not be the case if for example layered assemblies of ethylene glycol oligomers were present.

**Figure 9:**
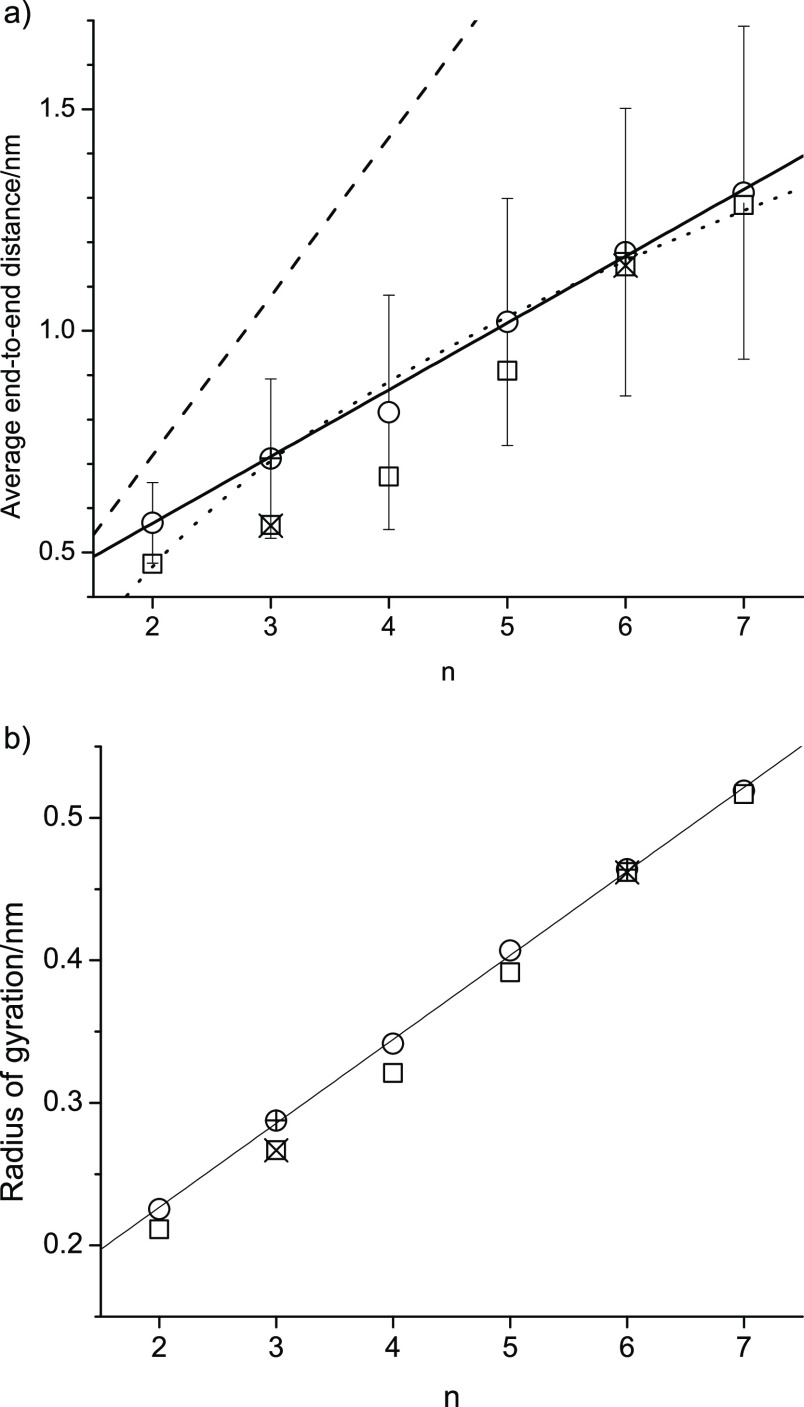
(a) Average end-to-end distance of ethylene glycol oligomers of hydroxy oxygen atoms in and (b) average radii of gyration of ethylene glycol oligomers, HO(CH_2_CH_2_O)_*n*_H in PEG200 simulated by OPLS force field (circles) and modified OPLS force field (squares). Data points for a binary mixture of tri- and hexaethylene glycol simulated with an OPLS force field (plus) and a modified OPLS force field (cross) are included. Solid lines are linear fits where the tetraethylene glycol data point was excluded. Standard deviations are included in (a) and a dashed line for comparison in the case where oligomers are in a completely stretched linear configuration. Error bars in (a) are similar in magnitude to square symbols but are omitted to reduce clutter. Error bars are omitted in (b) as they would be smaller than the size of the symbols. Also shown as dotted line in (a) is a fit to the data with the worm-like-chain model with a persistence length of 0.3791 nm.

The increased tendency to form intramolecular hydrogen bonding with concomitant ring-like structural configurations, especially for tetraethylene glycol, reduces the effective size of the molecule. This is reflected in the average end-to-end distance and the radius of gyration of each of the ethylene glycol oligomers in PEG200 shown in Figure 9. The dependence of the average end-to-end distance on the number of repeat units, *n*, is generally known to be *n*^1/2^ for large polymers.^68^ However, for the relatively short ethylene glycol oligomers in PEG200, it can be seen in Figure 9a that the average end-to-end distance increases approximately linearly with *n*, as evidenced by the included linear regression line. A fit to the worm-like chain model^69^
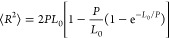
11where we equate the simulated end-to-end distance as the square root of the mean square end-to-end distance, ⟨*R*^2^⟩, is included in Figure 9. In eq 11, *L*_0_ is the end-to-end distance of each oligomer in a linear structural configuration such that the longest possible end-to-end distance is achieved (shown in Figure 9 as a dashed line), and *P* is the persistence parameter, which was obtained from fitting eq 11 to the data. Although eq 11 results in a nonlinear fit function, the fit values are within the shown error brackets, which represent the standard deviations of the average end-to-end distances, and we obtained a fit value for *P* of 0.3971 nm that is consistent with reported data for high molecular weight PEG, also referred to as polyethylene oxide.^19,70,71^ We note that the standard deviation increases with increasing n, which is readily understood since the structural configuration space increases with the increasing length of the ethylene glycol oligomer. The increased tendency to form intramolecular hydrogen bonds caused by the modifications to the OPLS force field explains the smaller average end-to-end distances, especially noticeable for the lower oligomers. The special tendency of tetraethylene glycol to form intramolecular hydrogen bonds is also noticeable from the unmodified OPLS force field by a relatively slightly smaller end-to-end distance. For this reason, the linear trendlines (solid line) in Figure 9 were obtained with the exclusion of the *n* = 4 data points. The significant difference between the dashed line and the solid line in Figure 9 illustrates a general deviation from the linear configuration for the oligomers in PEG200. Interestingly, the tri- and hexaethylene glycol average end-to-end distances obtained from simulating the binary mixture of these two are indistinguishable from the corresponding average end-to-end distances obtained from the PEG simulations. This suggests that although hydrogen bonding number counts for tri- and hexaethylene glycol differ between the binary and the PEG mixture due to the different compositions, their structural configurations may not be affected significantly by the different compositions. This is further confirmed when inspecting the average radii of gyration shown in Figure 9b, where the results from the binary mixture overlap with those from PEG200, and overall similar trends are observed as just described for the average end-to-end distances. The average radius of gyration obtained from the unmodified OPLS forcefield also increases linearly with n, except again for tetraethylene glycol, and the modifications to the OPLS forcefield also lead to smaller average radii of gyration for the oligomers di- to pentaethylene glycol while essentially no difference in average radius of gyration is observed for the hexa- and heptaethylene glycol.

The different hydrogen bonding behavior caused by the modifications to the OPLS forcefield, and the resulting structural changes explain the simulated physical properties of PEG200 in Table 5 as follows. The overall reduction in hydrogen bonding allows the ethylene glycol oligomers to move more freely, which increases their rates of self-diffusion. A second effect leading to faster self-diffusion is the reduced hydrodynamic radius caused by the increased tendency to form intramolecular hydrogen bonds. Less cohesive forces between molecules lead to less momentum transfer between molecules and, thus, a reduction in viscosity overall. Less cohesive forces also explain the observed decrease in density, which may not be as pronounced as one might expect because some volume is also freed from the removal of the orientational constraints that are required by hydrogen bonds.

## 4 Conclusions

The following conclusions can be drawn from the investigations presented in this report. None of the available force fields for describing ethylene glycol oligomers reproduce accurately the experimental properties. They either fall short of reproducing density, a static property, or they fall short of reproducing the self-diffusion coefficient, a dynamic property. Discrepancies with experimental data grew worse with the increasing size of the ethylene glycol oligomer. Agreement of simulated physical properties with experimental ones improved upon adjustment of the (HO)–C–C–O proper dihedral potential and the polarity of the hydroxy group for the OPLS force field. These adjustments need to be made to varying degrees depending on the size of the ethylene glycol oligomer, which suggests that future force field development should be less universal and more specific for each oligomer. PEG200, a polydisperse mixture of ethylene glycol oligomers ranging from di- to heptaethylene glycol, was simulated with the OPLS force field with and without modifications. These simulations showed that the modifications to the OPLS force field significantly decreased hydrogen bonding overall and shifted the preference of the remaining hydrogen bonding interactions toward intramolecular hydrogen bond formation at the cost of intermolecular hydrogen bond formation. Tri- and especially tetraethylene glycol showed a propensity to form intramolecular OH–OH hydrogen bonds while still maintaining OH–OE intermolecular interactions. The impacts of the different hydrogen bonding behaviors on the structural properties were significant in the RDFs as well as the average end-to-end distances and the average radii of gyration. Overall, the simulation results showed no evidence of preferential association of like-oligomers to form clusters. There was also no evidence of long-range ordering such as a side-by-side stacking of ethylene glycol oligomers. Instead, PEG200 may be viewed as a random mixture of its ethylene glycol oligomer components. Interestingly, MD simulations of a binary mixture of tri-and hexaethylene glycol with the same average molar weight as PEG200 showed very similar structural and physical properties as for PEG200. This observation is in qualitative agreement with an experimental study.^25^ Building on the findings of this report, more force field development is needed to improve the agreement of simulated properties with experimentally obtained values and further solidify the intricate details of the intra- and intermolecular hydrogen bonding behavior of PEGs. For example, the use of polarizable force fields^72,73^ might lead to more accurate dynamic properties.

## Data Availability

Parts of the supporting information have also been made available in the Zenodo depository.

## Supporting Information Available

Content of input files used for the reported simulations, comparison of dihedral potentials used in different force fields, python and bash script files used for running and analyzing MD simulations, tables detailing hydrogen bonding count results, and graphs representing intermolecular hydrogen bonding in PEG200 (PDF)
