# Supplementary material for: On the Behavior of the Ethylene Glycol Components of Polydisperse Polyethylene Glycol PEG200
Source: J Phys Chem B. 2023 Jan 26;127(5):1178–96. doi: 10.1021/acs.jpcb.2c06773 (PMC9923754; doi:10.1021/acs.jpcb.2c06773)
Supplement: Supplementary file 1 — jp2c06773_si_001.pdf [file jp2c06773_si_001.pdf]

Supplementary Materials

On the Behavior of the Ethylene Glycol Components of Polydisperse Polyethylene Glycol PEG200

Markus M. Hoffmann,*[1] Matthew D. Too,[1] Nathaniel A. Paddock,[1] Robin Horstmann,[2] Sebastian Kloth,[2] Michael Vogel,[2] and Gerd Buntkowsky*[3]

[1] *Department of Chemistry and Biochemistry, State University of New York College at Brockport, Brockport, NY, 14420, USA*

[2] *Institute of Condensed Matter Physics, Technical University Darmstadt, Hochschulstraße 6, 64289 Darmstadt, Germany*

[3] *Institute of Physical Chemistry, Technical University Darmstadt, Alarich-Weiss-Straße 8, D-64287 Darmstadt, Germany*

Corresponding authors: mhoffman@brockport.edu
gerd.buntkowsky@chemie.tu-darmstadt.de

1 Contents

2 Supporting information for MD simulations of neat ethylene glycol oligomers 3

2.1 OPLS forcefield 3

2.1.1 Topology files 3

2.1.2 Structure Coordinate files 38

2.1.3 Molecular Dynamics Parameter files 42

2.2 GROMOS forcefield 46

2.2.1 Topology files 46

2.2.2 Structure Coordinate file 49

2.2.3 MD Parameter files, typical example for tetraethylene glycol 50

2.3 CHARMM forcefield 54

2.3.1 Topology files 54

2.3.2 Structure Coordinate files 62

2.3.3 Molecular Dynamics Parameter files 63

2.4 AMBER forcefield 66

2.4.1 Topology file 66

2.4.2 Structure Coordinate file 76

2.4.3 Molecular Dynamics Parameter file 77

2.5 Martini forcefield 81

2.5.1 Topology file 81

2.5.2 Structure Coordinate file.....82
2.5.3 Molecular Dynamics Parameter file.....82
2.6 CZMP forcefield.....86
2.6.1 Topology file.....86
2.6.2 Structure Coordinate file.....90
2.6.3 Molecular Dynamics Parameter file.....91
3 Comparison of dihedral potential functions used by different forcefields.....96
4 Supporting Information for MD simulations of PEG200.....97
4.1 Using OPLS forcefield.....97
4.1.1 Topology files.....97
4.1.2 Structure Coordinate files.....124
4.1.3 Molecular Dynamics files.....128
4.2 Using modified OPLS forcefield.....132
4.2.1 Topology files.....132
4.2.2 Structure Coordinate files.....163
4.2.3 Molecular Dynamics files.....163
5 Script files.....164
5.1 Script files for finding slice having density equal to average density as starting configuration for NPT production run: "boxcut.sh" and "boxsize.py".....164
5.2 Viscosity analysis scripts "viscosity.sh" and "viscosity.py".....169
5.3 Bash script "repeated_simulations.sh" for running multiple MD simulations of same system.....177
5.4 Script for running MD simulation of PEG200.....180
5.5 Scripts for analyzing completed MD simulation of PEG200.....191
5.5.1 Python script file for handling analysis of radial distribution functions.....191
5.5.2 Bash script file for analysis from NPT run.....195
5.5.3 Bash script for analysis from NVT production run.....198
6 Oligomer-Oligomer Radial Distribution Functions.....215
6.1 RDFs obtained from unmodified OPLS Forcefield.....215
6.2 Modified OPLS forcefield.....218
7 Hydrogen bonding analysis results of various PEG200 runs.....221
8 Plots on intermolecular hydrogen bonding in PEG200.....231

2 Supporting information for MD simulations of neat ethylene glycol oligomers

2.1 OPLS forcefield

2.1.1 Topology files

The main topology file is of same format for each oligomer shown below for example for heptaethylene glycol (HepEG). For other oligomers, “HepEG” and comment text is replaced accordingly to include the appropriate itp files listed specifically below. Also the number of molecules is adjusted as desired.

Main topology file “top_HepEG_bulk.top”

```
; include force field
#include "oplsaa.ff/forcefield.itp"

; include heptaethylene glycol topology
#include "HepEG.itp"

; system level topology
[ system ]
Heptaethylene glycol

[ molecules ]
HepEG 250
```

Diethylene Glycol: Include topology file “DEG.itp”

```
;
; GENERATED BY LigParGen Server
; Jorgensen Lab @ Yale University
;
[ atomtypes ]
  opls_810  H810     1.0080     0.000    A    2.50000E-01   1.25520E-01
  opls_802  C802    12.0110     0.000    A    3.50000E-01   2.76144E-01
  opls_815  H815     1.0080     0.000    A    2.50000E-01   1.25520E-01
  opls_801  C801    12.0110     0.000    A    3.50000E-01   2.76144E-01
  opls_809  H809     1.0080     0.000    A    2.50000E-01   1.25520E-01
  opls_803  O803    15.9990     0.000    A    2.90000E-01   5.85760E-01
  opls_807  H807     1.0080     0.000    A    0.00000E+00   0.00000E+00
  opls_814  H814     1.0080     0.000    A    2.50000E-01   1.25520E-01
  opls_812  H812     1.0080     0.000    A    2.50000E-01   1.25520E-01
  opls_800  O800    15.9990     0.000    A    3.12000E-01   7.11280E-01
  opls_816  H816     1.0080     0.000    A    0.00000E+00   0.00000E+00
  opls_804  C804    12.0110     0.000    A    3.50000E-01   2.76144E-01
```

```
  opls_808  H808     1.0080     0.000    A    2.50000E-01   1.25520E-01
  opls_813  H813     1.0080     0.000    A    2.50000E-01   1.25520E-01
  opls_811  H811     1.0080     0.000    A    2.50000E-01   1.25520E-01
  opls_805  C805    12.0110     0.000    A    3.50000E-01   2.76144E-01
  opls_806  O806    15.9990     0.000    A    3.12000E-01   7.11280E-01
[ moleculetype ]
; Name               nrexcl
DEG                   3
[ atoms ]
;   nr       type  resnr residue  atom   cgnr     charge       mass
     1   opls_800      1    DEG   O00      1    -0.6888    15.9990
     2   opls_801      1    DEG   C01      1     0.1083    12.0110
     3   opls_802      1    DEG   C02      1     0.0070    12.0110
     4   opls_803      1    DEG   O03      1    -0.4006    15.9990
     5   opls_804      1    DEG   C04      1    -0.0412    12.0110
     6   opls_805      1    DEG   C05      1     0.1079    12.0110
     7   opls_806      1    DEG   O06      1    -0.6863    15.9990
     8   opls_807      1    DEG   H07      1     0.4182     1.0080
     9   opls_808      1    DEG   H08      1      0.082     1.0080
    10   opls_809      1    DEG   H09      1      0.082     1.0080
    11   opls_810      1    DEG   H0A      1     0.0928     1.0080
    12   opls_811      1    DEG   H0B      1     0.0928     1.0080
    13   opls_812      1    DEG   H0C      1     0.0946     1.0080
    14   opls_813      1    DEG   H0D      1     0.0946     1.0080
    15   opls_814      1    DEG   H0E      1     0.1067     1.0080
    16   opls_815      1    DEG   H0F      1     0.1067     1.0080
    17   opls_816      1    DEG   H0G      1     0.4233     1.0080
[ bonds ]
    2     1     1      0.1410 267776.000
    3     2     1      0.1529 224262.400
    4     3     1      0.1410 267776.000
    5     4     1      0.1410 267776.000
    6     5     1      0.1529 224262.400
    7     6     1      0.1410 267776.000
    8     1     1      0.0945 462750.400
    9     2     1      0.1090 284512.000
   10     2     1      0.1090 284512.000
   11     3     1      0.1090 284512.000
   12     3     1      0.1090 284512.000
   13     5     1      0.1090 284512.000
   14     5     1      0.1090 284512.000
   15     6     1      0.1090 284512.000
   16     6     1      0.1090 284512.000
   17     7     1      0.0945 462750.400
[ angles ]
;  ai    aj    ak funct            c0            c1            c2            c3
    1     2     3     1    109.500    418.400
    2     3     4     1    109.500    418.400
    3     4     5     1    109.500    502.080
    4     5     6     1    109.500    418.400
    5     6     7     1    109.500    418.400
    2     1     8     1    108.500    460.240
    1     2     9     1    109.500    292.880
    1     2    10     1    109.500    292.880
    2     3    11     1    110.700    313.800
    2     3    12     1    110.700    313.800
    4     5    13     1    109.500    292.880
```

```
    4     5    14     1    109.500    292.880
    5     6    15     1    110.700    313.800
    5     6    16     1    110.700    313.800
    6     7    17     1    108.500    460.240
    4     3    11     1    109.500    292.880
    9     2    10     1    107.800    276.144
   15     6    16     1    107.800    276.144
   13     5    14     1    107.800    276.144
    6     5    14     1    110.700    313.800
   11     3    12     1    107.800    276.144
    3     2     9     1    110.700    313.800
    6     5    13     1    110.700    313.800
    7     6    15     1    109.500    292.880
    7     6    16     1    109.500    292.880
    4     3    12     1    109.500    292.880
    3     2    10     1    110.700    313.800
[ dihedrals ]
; IMPROPER DIHEDRAL ANGLES
;  ai    aj    ak    al funct            c0            c1            c2            c3
c4            c5
[ dihedrals ]
; PROPER DIHEDRAL ANGLES
;  ai    aj    ak    al funct            c0            c1            c2            c3
c4            c5
    6    5    4    3        3       1.715   2.845   1.046  -5.607  -0.000   0.000
    5    4    3    2        3       1.715   2.845   1.046  -5.607  -0.000   0.000
   12    3    2    9        3       0.628   1.883   0.000  -2.510  -0.000   0.000
   16    6    5   13        3       0.628   1.883   0.000  -2.510  -0.000   0.000
   12    3    2   10        3       0.628   1.883   0.000  -2.510  -0.000   0.000
   16    6    5   14        3       0.628   1.883   0.000  -2.510  -0.000   0.000
   11    3    2   10        3       0.628   1.883   0.000  -2.510  -0.000   0.000
   15    6    5   13        3       0.628   1.883   0.000  -2.510  -0.000   0.000
   11    3    2    9        3       0.628   1.883   0.000  -2.510  -0.000   0.000
   15    6    5   14        3       0.628   1.883   0.000  -2.510  -0.000   0.000
   14    5    6    7        3       0.979   2.937   0.000  -3.916  -0.000   0.000
   12    3    2    1        3       0.979   2.937   0.000  -3.916  -0.000   0.000
   13    5    6    7        3       0.979   2.937   0.000  -3.916  -0.000   0.000
   11    3    2    1        3       0.979   2.937   0.000  -3.916  -0.000   0.000
   15    6    5    4        3       0.979   2.937   0.000  -3.916  -0.000   0.000
   16    6    5    4        3       0.979   2.937   0.000  -3.916  -0.000   0.000
   10    2    3    4        3       0.979   2.937   0.000  -3.916  -0.000   0.000
    9    2    3    4        3       0.979   2.937   0.000  -3.916  -0.000   0.000
    9    2    1    8        3       0.736   2.209   0.000  -2.946  -0.000   0.000
   10    2    1    8        3       0.736   2.209   0.000  -2.946  -0.000   0.000
   13    5    4    3        3       1.590   4.770   0.000  -6.360  -0.000   0.000
   11    3    4    5        3       1.590   4.770   0.000  -6.360  -0.000   0.000
   14    5    4    3        3       1.590   4.770   0.000  -6.360  -0.000   0.000
   12    3    4    5        3       1.590   4.770   0.000  -6.360  -0.000   0.000
   17    7    6    5        3      -0.444   3.833   0.728  -4.117  -0.000   0.000
    8    1    2    3        3      -0.444   3.833   0.728  -4.117  -0.000   0.000
   17    7    6   15        3       0.736   2.209   0.000  -2.946  -0.000   0.000
   17    7    6   16        3       0.736   2.209   0.000  -2.946  -0.000   0.000
    7    6    5    4        3       9.035  -9.035   0.000  -0.000  -0.000   0.000
    4    3    2    1        3       9.035  -9.035   0.000  -0.000  -0.000   0.000
[ pairs ]
     1     4    1
     2     5    1
```

```
     3      6    1
     4      7    1
     3      8    1
     1     11    1
     4      9    1
     1     12    1
     4     10    1
     5     11    1
     3     13    1
     8      9    1
     5     12    1
     3     14    1
     8     10    1
     4     15    1
     9     11    1
     7     13    1
     4     16    1
    10     11    1
     9     12    1
     7     14    1
    10     12    1
     5     17    1
    13     15    1
    14     15    1
    13     16    1
    14     16    1
    15     17    1
    16     17    1
```

Triethylene Glycol: Include topology file "TEG.itp"

```
;
; GENERATED BY LigParGen Server
; Jorgensen Lab @ Yale University
;
[ atomtypes ]
  opls_818  H818     1.0080     0.000    A    2.50000E-01   1.25520E-01
  opls_803  O803    15.9990     0.000    A    2.90000E-01   5.85760E-01
  opls_805  C805    12.0110     0.000    A    3.50000E-01   2.76144E-01
  opls_823  H823     1.0080     0.000    A    0.00000E+00   0.00000E+00
  opls_800  O800    15.9990     0.000    A    3.12000E-01   7.11280E-01
  opls_813  H813     1.0080     0.000    A    2.50000E-01   1.25520E-01
  opls_806  O806    15.9990     0.000    A    2.90000E-01   5.85760E-01
  opls_802  C802    12.0110     0.000    A    3.50000E-01   2.76144E-01
  opls_815  H815     1.0080     0.000    A    2.50000E-01   1.25520E-01
  opls_801  C801    12.0110     0.000    A    3.50000E-01   2.76144E-01
  opls_822  H822     1.0080     0.000    A    2.50000E-01   1.25520E-01
  opls_807  C807    12.0110     0.000    A    3.50000E-01   2.76144E-01
  opls_814  H814     1.0080     0.000    A    2.50000E-01   1.25520E-01
  opls_821  H821     1.0080     0.000    A    2.50000E-01   1.25520E-01
  opls_820  H820     1.0080     0.000    A    2.50000E-01   1.25520E-01
  opls_811  H811     1.0080     0.000    A    2.50000E-01   1.25520E-01
  opls_816  H816     1.0080     0.000    A    2.50000E-01   1.25520E-01
  opls_809  O809    15.9990     0.000    A    3.12000E-01   7.11280E-01
  opls_819  H819     1.0080     0.000    A    2.50000E-01   1.25520E-01
  opls_804  C804    12.0110     0.000    A    3.50000E-01   2.76144E-01
  opls_808  C808    12.0110     0.000    A    3.50000E-01   2.76144E-01
```

```
  opls_817  H817     1.0080     0.000    A    2.50000E-01   1.25520E-01
  opls_812  H812     1.0080     0.000    A    2.50000E-01   1.25520E-01
  opls_810  H810     1.0080     0.000    A    0.00000E+00   0.00000E+00
[ moleculetype ]
; Name               nrexcl
TEG                   3
[ atoms ]
;   nr       type  resnr residue  atom   cgnr     charge       mass
     1   opls_800      1    TEG   O00      1    -0.6904    15.9990
     2   opls_801      1    TEG   C01      1     0.1087    12.0110
     3   opls_802      1    TEG   C02      1     0.0049    12.0110
     4   opls_803      1    TEG   O03      1     -0.378    15.9990
     5   opls_804      1    TEG   C04      1     0.0098    12.0110
     6   opls_805      1    TEG   C05      1     0.0105    12.0110
     7   opls_806      1    TEG   O06      1    -0.3919    15.9990
     8   opls_807      1    TEG   C07      1    -0.0444    12.0110
     9   opls_808      1    TEG   C08      1     0.1087    12.0110
    10   opls_809      1    TEG   O09      1     -0.687    15.9990
    11   opls_810      1    TEG   H0A      1     0.4169     1.0080
    12   opls_811      1    TEG   H0B      1     0.0829     1.0080
    13   opls_812      1    TEG   H0C      1     0.0829     1.0080
    14   opls_813      1    TEG   H0D      1     0.0892     1.0080
    15   opls_814      1    TEG   H0E      1     0.0892     1.0080
    16   opls_815      1    TEG   H0F      1     0.0927     1.0080
    17   opls_816      1    TEG   H0G      1     0.0927     1.0080
    18   opls_817      1    TEG   H0H      1     0.0925     1.0080
    19   opls_818      1    TEG   H0I      1     0.0925     1.0080
    20   opls_819      1    TEG   H0J      1     0.0905     1.0080
    21   opls_820      1    TEG   H0K      1     0.0905     1.0080
    22   opls_821      1    TEG   H0M      1     0.1057     1.0080
    23   opls_822      1    TEG   H0N      1     0.1057     1.0080
    24   opls_823      1    TEG   H0O      1     0.4252     1.0080
[ bonds ]
    2     1     1      0.1410 267776.000
    3     2     1      0.1529 224262.400
    4     3     1      0.1410 267776.000
    5     4     1      0.1410 267776.000
    6     5     1      0.1529 224262.400
    7     6     1      0.1410 267776.000
    8     7     1      0.1410 267776.000
    9     8     1      0.1529 224262.400
   10     9     1      0.1410 267776.000
   11     1     1      0.0945 462750.400
   12     2     1      0.1090 284512.000
   13     2     1      0.1090 284512.000
   14     3     1      0.1090 284512.000
   15     3     1      0.1090 284512.000
   16     5     1      0.1090 284512.000
   17     5     1      0.1090 284512.000
   18     6     1      0.1090 284512.000
   19     6     1      0.1090 284512.000
   20     8     1      0.1090 284512.000
   21     8     1      0.1090 284512.000
   22     9     1      0.1090 284512.000
   23     9     1      0.1090 284512.000
   24    10     1      0.0945 462750.400
```

```
[ angles ]
;  ai    aj    ak funct            c0            c1            c2            c3
    1     2     3     1    109.500    418.400
    2     3     4     1    109.500    418.400
    3     4     5     1    109.500    502.080
    4     5     6     1    109.500    418.400
    5     6     7     1    109.500    418.400
    6     7     8     1    109.500    502.080
    7     8     9     1    109.500    418.400
    8     9    10     1    109.500    418.400
    2     1    11     1    108.500    460.240
    1     2    12     1    109.500    292.880
    1     2    13     1    109.500    292.880
    2     3    14     1    110.700    313.800
    2     3    15     1    110.700    313.800
    4     5    16     1    109.500    292.880
    4     5    17     1    109.500    292.880
    5     6    18     1    110.700    313.800
    5     6    19     1    110.700    313.800
    7     8    20     1    109.500    292.880
    7     8    21     1    109.500    292.880
    8     9    22     1    110.700    313.800
    8     9    23     1    110.700    313.800
    9    10    24     1    108.500    460.240
    7     6    18     1    109.500    292.880
   12     2    13     1    107.800    276.144
   18     6    19     1    107.800    276.144
    9     8    20     1    110.700    313.800
   16     5    17     1    107.800    276.144
    6     5    17     1    110.700    313.800
   10     9    23     1    109.500    292.880
   22     9    23     1    107.800    276.144
    7     6    19     1    109.500    292.880
   14     3    15     1    107.800    276.144
   10     9    22     1    109.500    292.880
    3     2    12     1    110.700    313.800
   20     8    21     1    107.800    276.144
    6     5    16     1    110.700    313.800
    3     2    13     1    110.700    313.800
    4     3    14     1    109.500    292.880
    9     8    21     1    110.700    313.800
    4     3    15     1    109.500    292.880

[ dihedrals ]
; IMPROPER DIHEDRAL ANGLES
;  ai    aj    ak    al funct            c0            c1            c2            c3
c4            c5

[ dihedrals ]
; PROPER DIHEDRAL ANGLES
;  ai    aj    ak    al funct            c0            c1            c2            c3
c4            c5
    6    5    4    3         3       1.715   2.845   1.046  -5.607  -0.000   0.000
    9    8    7    6         3       1.715   2.845   1.046  -5.607  -0.000   0.000
    5    4    3    2         3       1.715   2.845   1.046  -5.607  -0.000   0.000
    8    7    6    5         3       1.715   2.845   1.046  -5.607  -0.000   0.000
   14    3    2   12         3       0.628   1.883   0.000  -2.510  -0.000   0.000
```

```
   23    9    8   20         3       0.628   1.883   0.000  -2.510  -0.000   0.000
   22    9    8   21         3       0.628   1.883   0.000  -2.510  -0.000   0.000
   18    6    5   16         3       0.628   1.883   0.000  -2.510  -0.000   0.000
   15    3    2   12         3       0.628   1.883   0.000  -2.510  -0.000   0.000
   14    3    2   13         3       0.628   1.883   0.000  -2.510  -0.000   0.000
   23    9    8   21         3       0.628   1.883   0.000  -2.510  -0.000   0.000
   22    9    8   20         3       0.628   1.883   0.000  -2.510  -0.000   0.000
   19    6    5   17         3       0.628   1.883   0.000  -2.510  -0.000   0.000
   18    6    5   17         3       0.628   1.883   0.000  -2.510  -0.000   0.000
   15    3    2   13         3       0.628   1.883   0.000  -2.510  -0.000   0.000
   19    6    5   16         3       0.628   1.883   0.000  -2.510  -0.000   0.000
   21    8    9   10         3       0.979   2.937   0.000  -3.916  -0.000   0.000
   14    3    2    1         3       0.979   2.937   0.000  -3.916  -0.000   0.000
   20    8    9   10         3       0.979   2.937   0.000  -3.916  -0.000   0.000
   15    3    2    1         3       0.979   2.937   0.000  -3.916  -0.000   0.000
   23    9    8    7         3       0.979   2.937   0.000  -3.916  -0.000   0.000
   18    6    5    4         3       0.979   2.937   0.000  -3.916  -0.000   0.000
   13    2    3    4         3       0.979   2.937   0.000  -3.916  -0.000   0.000
   19    6    5    4         3       0.979   2.937   0.000  -3.916  -0.000   0.000
   16    5    6    7         3       0.979   2.937   0.000  -3.916  -0.000   0.000
   17    5    6    7         3       0.979   2.937   0.000  -3.916  -0.000   0.000
   22    9    8    7         3       0.979   2.937   0.000  -3.916  -0.000   0.000
   12    2    3    4         3       0.979   2.937   0.000  -3.916  -0.000   0.000
   12    2    1   11         3       0.736   2.209   0.000  -2.946  -0.000   0.000
   13    2    1   11         3       0.736   2.209   0.000  -2.946  -0.000   0.000
   19    6    7    8         3       1.590   4.770   0.000  -6.360  -0.000   0.000
   20    8    7    6         3       1.590   4.770   0.000  -6.360  -0.000   0.000
   18    6    7    8         3       1.590   4.770   0.000  -6.360  -0.000   0.000
   16    5    4    3         3       1.590   4.770   0.000  -6.360  -0.000   0.000
   17    5    4    3         3       1.590   4.770   0.000  -6.360  -0.000   0.000
   14    3    4    5         3       1.590   4.770   0.000  -6.360  -0.000   0.000
   15    3    4    5         3       1.590   4.770   0.000  -6.360  -0.000   0.000
   21    8    7    6         3       1.590   4.770   0.000  -6.360  -0.000   0.000
   24   10    9    8         3      -0.444   3.833   0.728  -4.117  -0.000   0.000
   11    1    2    3         3      -0.444   3.833   0.728  -4.117  -0.000   0.000
   24   10    9   22         3       0.736   2.209   0.000  -2.946  -0.000   0.000
   24   10    9   23         3       0.736   2.209   0.000  -2.946  -0.000   0.000
   10    9    8    7         3       9.035  -9.035   0.000  -0.000  -0.000   0.000
    4    3    2    1         3       9.035  -9.035   0.000  -0.000  -0.000   0.000
    7    6    5    4         3      -1.151   1.151   0.000  -0.000  -0.000   0.000

[ pairs ]
     1     4    1
     2     5    1
     3     6    1
     4     7    1
     5     8    1
     3    11    1
     6     9    1
     1    14    1
     4    12    1
     1    15    1
     7    10    1
     4    13    1
     5    14    1
     3    16    1
     5    15    1
```

```
    3    17    1
    4    18    1
   11    12    1
    7    16    1
    4    19    1
   11    13    1
    7    17    1
   12    14    1
    8    18    1
    6    20    1
   13    14    1
   12    15    1
    8    19    1
    6    21    1
   13    15    1
    7    22    1
   10    20    1
    7    23    1
   10    21    1
    8    24    1
   16    18    1
   17    18    1
   16    19    1
   17    19    1
   20    22    1
   21    22    1
   20    23    1
   21    23    1
   22    24    1
   23    24    1
```

Tetraethylene Glycol: Include topology file "TeEG.itp"

```
;
; GENERATED BY LigParGen Server
; Jorgensen Lab @ Yale University
;
[ atomtypes ]
  opls_818  H818     1.0080     0.000    A    2.50000E-01   1.25520E-01
  opls_803  O803    15.9990     0.000    A    2.90000E-01   5.85760E-01
  opls_824  H824     1.0080     0.000    A    2.50000E-01   1.25520E-01
  opls_809  O809    15.9990     0.000    A    2.90000E-01   5.85760E-01
  opls_800  O800    15.9990     0.000    A    3.12000E-01   7.11280E-01
  opls_828  H828     1.0080     0.000    A    2.50000E-01   1.25520E-01
  opls_806  O806    15.9990     0.000    A    2.90000E-01   5.85760E-01
  opls_802  C802    12.0110     0.000    A    3.50000E-01   2.76144E-01
  opls_815  H815     1.0080     0.000    A    2.50000E-01   1.25520E-01
  opls_801  C801    12.0110     0.000    A    3.50000E-01   2.76144E-01
  opls_822  H822     1.0080     0.000    A    2.50000E-01   1.25520E-01
  opls_807  C807    12.0110     0.000    A    3.50000E-01   2.76144E-01
  opls_825  H825     1.0080     0.000    A    2.50000E-01   1.25520E-01
  opls_814  H814     1.0080     0.000    A    2.50000E-01   1.25520E-01
  opls_810  C810    12.0110     0.000    A    3.50000E-01   2.76144E-01
  opls_820  H820     1.0080     0.000    A    2.50000E-01   1.25520E-01
  opls_821  H821     1.0080     0.000    A    2.50000E-01   1.25520E-01
  opls_816  H816     1.0080     0.000    A    2.50000E-01   1.25520E-01
  opls_812  O812    15.9990     0.000    A    3.12000E-01   7.11280E-01
```

```
  opls_811  C811    12.0110     0.000    A    3.50000E-01   2.76144E-01
  opls_823  H823     1.0080     0.000    A    2.50000E-01   1.25520E-01
  opls_813  H813     1.0080     0.000    A    0.00000E+00   0.00000E+00
  opls_805  C805    12.0110     0.000    A    3.50000E-01   2.76144E-01
  opls_826  H826     1.0080     0.000    A    2.50000E-01   1.25520E-01
  opls_819  H819     1.0080     0.000    A    2.50000E-01   1.25520E-01
  opls_804  C804    12.0110     0.000    A    3.50000E-01   2.76144E-01
  opls_827  H827     1.0080     0.000    A    2.50000E-01   1.25520E-01
  opls_808  C808    12.0110     0.000    A    3.50000E-01   2.76144E-01
  opls_817  H817     1.0080     0.000    A    2.50000E-01   1.25520E-01
  opls_829  H829     1.0080     0.000    A    2.50000E-01   1.25520E-01
  opls_830  H830     1.0080     0.000    A    0.00000E+00   0.00000E+00
[ moleculetype ]
; Name                 nrexcl
TeEG                     3
[ atoms ]
;   nr       type  resnr residue  atom   cgnr     charge       mass
     1   opls_800      1    TeEG   O00      1    -0.6888    15.9990
     2   opls_801      1    TeEG   C01      1     0.1085    12.0110
     3   opls_802      1    TeEG   C02      1     0.0084    12.0110
     4   opls_803      1    TeEG   O03      1    -0.3854    15.9990
     5   opls_804      1    TeEG   C04      1     0.0086    12.0110
     6   opls_805      1    TeEG   C05      1     0.0089    12.0110
     7   opls_806      1    TeEG   O06      1    -0.3849    15.9990
     8   opls_807      1    TeEG   C07      1     0.0089    12.0110
     9   opls_808      1    TeEG   C08      1     0.0067    12.0110
    10   opls_809      1    TeEG   O09      1    -0.3989    15.9990
    11   opls_810      1    TeEG   C0A      1    -0.0409    12.0110
    12   opls_811      1    TeEG   C0B      1     0.1079    12.0110
    13   opls_812      1    TeEG   O0C      1    -0.6858    15.9990
    14   opls_813      1    TeEG   H0D      1     0.4178     1.0080
    15   opls_814      1    TeEG   H0E      1     0.0821     1.0080
    16   opls_815      1    TeEG   H0F      1     0.0821     1.0080
    17   opls_816      1    TeEG   H0G      1     0.0926     1.0080
    18   opls_817      1    TeEG   H0H      1     0.0926     1.0080
    19   opls_818      1    TeEG   H0I      1     0.0916     1.0080
    20   opls_819      1    TeEG   H0J      1     0.0916     1.0080
    21   opls_820      1    TeEG   H0K      1     0.0917     1.0080
    22   opls_821      1    TeEG   H0M      1     0.0917     1.0080
    23   opls_822      1    TeEG   H0N      1     0.0913     1.0080
    24   opls_823      1    TeEG   H0O      1     0.0913     1.0080
    25   opls_824      1    TeEG   H0P      1      0.092     1.0080
    26   opls_825      1    TeEG   H0Q      1      0.092     1.0080
    27   opls_826      1    TeEG   H0R      1     0.0946     1.0080
    28   opls_827      1    TeEG   H0S      1     0.0946     1.0080
    29   opls_828      1    TeEG   H0T      1     0.1069     1.0080
    30   opls_829      1    TeEG   H0U      1     0.1069     1.0080
    31   opls_830      1    TeEG   H0V      1     0.4234     1.0080
[ bonds ]
    2     1     1      0.1410 267776.000
    3     2     1      0.1529 224262.400
    4     3     1      0.1410 267776.000
    5     4     1      0.1410 267776.000
    6     5     1      0.1529 224262.400
    7     6     1      0.1410 267776.000
    8     7     1      0.1410 267776.000
    9     8     1      0.1529 224262.400
```

```
   10     9     1      0.1410 267776.000
   11    10     1      0.1410 267776.000
   12    11     1      0.1529 224262.400
   13    12     1      0.1410 267776.000
   14     1     1      0.0945 462750.400
   15     2     1      0.1090 284512.000
   16     2     1      0.1090 284512.000
   17     3     1      0.1090 284512.000
   18     3     1      0.1090 284512.000
   19     5     1      0.1090 284512.000
   20     5     1      0.1090 284512.000
   21     6     1      0.1090 284512.000
   22     6     1      0.1090 284512.000
   23     8     1      0.1090 284512.000
   24     8     1      0.1090 284512.000
   25     9     1      0.1090 284512.000
   26     9     1      0.1090 284512.000
   27    11     1      0.1090 284512.000
   28    11     1      0.1090 284512.000
   29    12     1      0.1090 284512.000
   30    12     1      0.1090 284512.000
   31    13     1      0.0945 462750.400
[ angles ]
;  ai    aj    ak funct            c0            c1            c2            c3
    1     2     3     1    109.500    418.400
    2     3     4     1    109.500    418.400
    3     4     5     1    109.500    502.080
    4     5     6     1    109.500    418.400
    5     6     7     1    109.500    418.400
    6     7     8     1    109.500    502.080
    7     8     9     1    109.500    418.400
    8     9    10     1    109.500    418.400
    9    10    11     1    109.500    502.080
   10    11    12     1    109.500    418.400
   11    12    13     1    109.500    418.400
    2     1    14     1    108.500    460.240
    1     2    15     1    109.500    292.880
    1     2    16     1    109.500    292.880
    2     3    17     1    110.700    313.800
    2     3    18     1    110.700    313.800
    4     5    19     1    109.500    292.880
    4     5    20     1    109.500    292.880
    5     6    21     1    110.700    313.800
    5     6    22     1    110.700    313.800
    7     8    23     1    109.500    292.880
    7     8    24     1    109.500    292.880
    8     9    25     1    110.700    313.800
    8     9    26     1    110.700    313.800
   10    11    27     1    109.500    292.880
   10    11    28     1    109.500    292.880
   11    12    29     1    110.700    313.800
   11    12    30     1    110.700    313.800
   12    13    31     1    108.500    460.240
   25     9    26     1    107.800    276.144
   23     8    24     1    107.800    276.144
    3     2    15     1    110.700    313.800
   29    12    30     1    107.800    276.144
```

```
   12    11    27     1    110.700    313.800
   15     2    16     1    107.800    276.144
    7     6    22     1    109.500    292.880
   21     6    22     1    107.800    276.144
   27    11    28     1    107.800    276.144
    6     5    19     1    110.700    313.800
    9     8    23     1    110.700    313.800
   13    12    29     1    109.500    292.880
    3     2    16     1    110.700    313.800
    4     3    18     1    109.500    292.880
    9     8    24     1    110.700    313.800
   19     5    20     1    107.800    276.144
   13    12    30     1    109.500    292.880
    6     5    20     1    110.700    313.800
   10     9    25     1    109.500    292.880
   10     9    26     1    109.500    292.880
    4     3    17     1    109.500    292.880
   17     3    18     1    107.800    276.144
   12    11    28     1    110.700    313.800
    7     6    21     1    109.500    292.880
[ dihedrals ]
; IMPROPER DIHEDRAL ANGLES
;  ai    aj    ak    al funct            c0            c1            c2            c3
c4            c5
[ dihedrals ]
; PROPER DIHEDRAL ANGLES
;  ai    aj    ak    al funct            c0            c1            c2            c3
c4            c5
    6    5    4    3        3       1.715   2.845   1.046  -5.607  -0.000   0.000
    9    8    7    6        3       1.715   2.845   1.046  -5.607  -0.000   0.000
   12   11   10    9        3       1.715   2.845   1.046  -5.607  -0.000   0.000
    5    4    3    2        3       1.715   2.845   1.046  -5.607  -0.000   0.000
    8    7    6    5        3       1.715   2.845   1.046  -5.607  -0.000   0.000
   11   10    9    8        3       1.715   2.845   1.046  -5.607  -0.000   0.000
   18    3    2   15        3       0.628   1.883   0.000  -2.510  -0.000   0.000
   18    3    2   16        3       0.628   1.883   0.000  -2.510  -0.000   0.000
   21    6    5   20        3       0.628   1.883   0.000  -2.510  -0.000   0.000
   21    6    5   19        3       0.628   1.883   0.000  -2.510  -0.000   0.000
   22    6    5   19        3       0.628   1.883   0.000  -2.510  -0.000   0.000
   29   12   11   28        3       0.628   1.883   0.000  -2.510  -0.000   0.000
   30   12   11   28        3       0.628   1.883   0.000  -2.510  -0.000   0.000
   17    3    2   16        3       0.628   1.883   0.000  -2.510  -0.000   0.000
   22    6    5   20        3       0.628   1.883   0.000  -2.510  -0.000   0.000
   30   12   11   27        3       0.628   1.883   0.000  -2.510  -0.000   0.000
   17    3    2   15        3       0.628   1.883   0.000  -2.510  -0.000   0.000
   26    9    8   24        3       0.628   1.883   0.000  -2.510  -0.000   0.000
   26    9    8   23        3       0.628   1.883   0.000  -2.510  -0.000   0.000
   29   12   11   27        3       0.628   1.883   0.000  -2.510  -0.000   0.000
   25    9    8   23        3       0.628   1.883   0.000  -2.510  -0.000   0.000
   25    9    8   24        3       0.628   1.883   0.000  -2.510  -0.000   0.000
   28   11   12   13        3       0.979   2.937   0.000  -3.916  -0.000   0.000
   18    3    2    1        3       0.979   2.937   0.000  -3.916  -0.000   0.000
   27   11   12   13        3       0.979   2.937   0.000  -3.916  -0.000   0.000
   17    3    2    1        3       0.979   2.937   0.000  -3.916  -0.000   0.000
   19    5    6    7        3       0.979   2.937   0.000  -3.916  -0.000   0.000
   20    5    6    7        3       0.979   2.937   0.000  -3.916  -0.000   0.000
   30   12   11   10        3       0.979   2.937   0.000  -3.916  -0.000   0.000
```

```
   26    9    8    7        3       0.979   2.937   0.000  -3.916  -0.000   0.000
   24    8    9   10        3       0.979   2.937   0.000  -3.916  -0.000   0.000
   22    6    5    4        3       0.979   2.937   0.000  -3.916  -0.000   0.000
   23    8    9   10        3       0.979   2.937   0.000  -3.916  -0.000   0.000
   29   12   11   10        3       0.979   2.937   0.000  -3.916  -0.000   0.000
   21    6    5    4        3       0.979   2.937   0.000  -3.916  -0.000   0.000
   25    9    8    7        3       0.979   2.937   0.000  -3.916  -0.000   0.000
   15    2    3    4        3       0.979   2.937   0.000  -3.916  -0.000   0.000
   16    2    3    4        3       0.979   2.937   0.000  -3.916  -0.000   0.000
   16    2    1   14        3       0.736   2.209   0.000  -2.946  -0.000   0.000
   15    2    1   14        3       0.736   2.209   0.000  -2.946  -0.000   0.000
   21    6    7    8        3       1.590   4.770   0.000  -6.360  -0.000   0.000
   24    8    7    6        3       1.590   4.770   0.000  -6.360  -0.000   0.000
   22    6    7    8        3       1.590   4.770   0.000  -6.360  -0.000   0.000
   28   11   10    9        3       1.590   4.770   0.000  -6.360  -0.000   0.000
   26    9   10   11        3       1.590   4.770   0.000  -6.360  -0.000   0.000
   19    5    4    3        3       1.590   4.770   0.000  -6.360  -0.000   0.000
   25    9   10   11        3       1.590   4.770   0.000  -6.360  -0.000   0.000
   17    3    4    5        3       1.590   4.770   0.000  -6.360  -0.000   0.000
   20    5    4    3        3       1.590   4.770   0.000  -6.360  -0.000   0.000
   23    8    7    6        3       1.590   4.770   0.000  -6.360  -0.000   0.000
   18    3    4    5        3       1.590   4.770   0.000  -6.360  -0.000   0.000
   27   11   10    9        3       1.590   4.770   0.000  -6.360  -0.000   0.000
   31   13   12   11        3      -0.444   3.833   0.728  -4.117  -0.000   0.000
   14    1    2    3        3      -0.444   3.833   0.728  -4.117  -0.000   0.000
   31   13   12   30        3       0.736   2.209   0.000  -2.946  -0.000   0.000
   31   13   12   29        3       0.736   2.209   0.000  -2.946  -0.000   0.000
   13   12   11   10        3       9.035  -9.035   0.000  -0.000  -0.000   0.000
    4    3    2    1        3       9.035  -9.035   0.000  -0.000  -0.000   0.000
    7    6    5    4        3      -1.151   1.151   0.000  -0.000  -0.000   0.000
   10    9    8    7        3      -1.151   1.151   0.000  -0.000  -0.000   0.000
[ pairs ]
     1     4    1
     2     5    1
     3     6    1
     4     7    1
     5     8    1
     6     9    1
     7    10    1
     3    14    1
     1    17    1
     8    11    1
     4    15    1
     1    18    1
     4    16    1
     9    12    1
     5    17    1
     3    19    1
    10    13    1
     5    18    1
     3    20    1
     4    21    1
     7    19    1
     4    22    1
     7    20    1
    14    15    1
     8    21    1
```

```
     6    23    1
    14    16    1
     8    22    1
     6    24    1
    15    17    1
     7    25    1
    16    17    1
    15    18    1
    10    23    1
     7    26    1
    16    18    1
    10    24    1
    11    25    1
     9    27    1
    11    26    1
     9    28    1
    10    29    1
    19    21    1
    13    27    1
    10    30    1
    20    21    1
    19    22    1
    13    28    1
    20    22    1
    11    31    1
    23    25    1
    24    25    1
    23    26    1
    24    26    1
    27    29    1
    28    29    1
    27    30    1
    28    30    1
    29    31    1
    30    31    1
```

Pentaethylene Glycol: Include topology file "PeEG.itp"

```
;
; GENERATED BY LigParGen Server
; Jorgensen Lab @ Yale University
;
[ atomtypes ]
  opls_818  H818     1.0080     0.000    A    2.50000E-01   1.25520E-01
  opls_814  C814    12.0110     0.000    A    3.50000E-01   2.76144E-01
  opls_803  O803    15.9990     0.000    A    2.90000E-01   5.85760E-01
  opls_824  H824     1.0080     0.000    A    2.50000E-01   1.25520E-01
  opls_809  O809    15.9990     0.000    A    2.90000E-01   5.85760E-01
  opls_800  O800    15.9990     0.000    A    3.12000E-01   7.11280E-01
  opls_836  H836     1.0080     0.000    A    2.50000E-01   1.25520E-01
  opls_834  H834     1.0080     0.000    A    2.50000E-01   1.25520E-01
  opls_810  C810    12.0110     0.000    A    3.50000E-01   2.76144E-01
  opls_828  H828     1.0080     0.000    A    2.50000E-01   1.25520E-01
  opls_812  O812    15.9990     0.000    A    2.90000E-01   5.85760E-01
  opls_837  H837     1.0080     0.000    A    0.00000E+00   0.00000E+00
  opls_832  H832     1.0080     0.000    A    2.50000E-01   1.25520E-01
  opls_806  O806    15.9990     0.000    A    2.90000E-01   5.85760E-01
```

```
  opls_802  C802    12.0110     0.000    A    3.50000E-01   2.76144E-01
  opls_827  H827     1.0080     0.000    A    2.50000E-01   1.25520E-01
  opls_801  C801    12.0110     0.000    A    3.50000E-01   2.76144E-01
  opls_822  H822     1.0080     0.000    A    2.50000E-01   1.25520E-01
  opls_807  C807    12.0110     0.000    A    3.50000E-01   2.76144E-01
  opls_825  H825     1.0080     0.000    A    2.50000E-01   1.25520E-01
  opls_830  H830     1.0080     0.000    A    2.50000E-01   1.25520E-01
  opls_821  H821     1.0080     0.000    A    2.50000E-01   1.25520E-01
  opls_820  H820     1.0080     0.000    A    2.50000E-01   1.25520E-01
  opls_815  O815    15.9990     0.000    A    3.12000E-01   7.11280E-01
  opls_831  H831     1.0080     0.000    A    2.50000E-01   1.25520E-01
  opls_816  H816     1.0080     0.000    A    0.00000E+00   0.00000E+00
  opls_805  C805    12.0110     0.000    A    3.50000E-01   2.76144E-01
  opls_811  C811    12.0110     0.000    A    3.50000E-01   2.76144E-01
  opls_823  H823     1.0080     0.000    A    2.50000E-01   1.25520E-01
  opls_813  C813    12.0110     0.000    A    3.50000E-01   2.76144E-01
  opls_826  H826     1.0080     0.000    A    2.50000E-01   1.25520E-01
  opls_835  H835     1.0080     0.000    A    2.50000E-01   1.25520E-01
  opls_819  H819     1.0080     0.000    A    2.50000E-01   1.25520E-01
  opls_804  C804    12.0110     0.000    A    3.50000E-01   2.76144E-01
  opls_833  H833     1.0080     0.000    A    2.50000E-01   1.25520E-01
  opls_808  C808    12.0110     0.000    A    3.50000E-01   2.76144E-01
  opls_817  H817     1.0080     0.000    A    2.50000E-01   1.25520E-01
  opls_829  H829     1.0080     0.000    A    2.50000E-01   1.25520E-01
[ moleculetype ]
; Name              nrexcl
PeEG                3
[ atoms ]
;   nr       type  resnr residue  atom   cgnr     charge       mass
     1   opls_800      1    PeEG O00      1    -0.6886    15.9990
     2   opls_801      1    PeEG C01      1      0.108    12.0110
     3   opls_802      1    PeEG C02      1     0.0082    12.0110
     4   opls_803      1    PeEG O03      1    -0.3867    15.9990
     5   opls_804      1    PeEG C04      1     0.0082    12.0110
     6   opls_805      1    PeEG C05      1     0.0055    12.0110
     7   opls_806      1    PeEG O06      1    -0.3768    15.9990
     8   opls_807      1    PeEG C07      1     0.0113    12.0110
     9   opls_808      1    PeEG C08      1     0.0113    12.0110
    10   opls_809      1    PeEG O09      1    -0.3768    15.9990
    11   opls_810      1    PeEG C0A      1     0.0053    12.0110
    12   opls_811      1    PeEG C0B      1      0.007    12.0110
    13   opls_812      1    PeEG O0C      1    -0.4004    15.9990
    14   opls_813      1    PeEG C0D      1     -0.041    12.0110
    15   opls_814      1    PeEG C0E      1     0.1082    12.0110
    16   opls_815      1    PeEG O0F      1    -0.6862    15.9990
    17   opls_816      1    PeEG H0G      1     0.4171     1.0080
    18   opls_817      1    PeEG H0H      1     0.0817     1.0080
    19   opls_818      1    PeEG H0I      1     0.0817     1.0080
    20   opls_819      1    PeEG H0J      1     0.0925     1.0080
    21   opls_820      1    PeEG H0K      1     0.0925     1.0080
    22   opls_821      1    PeEG H0M      1     0.0926     1.0080
    23   opls_822      1    PeEG H0N      1     0.0926     1.0080
    24   opls_823      1    PeEG H0O      1     0.0877     1.0080
    25   opls_824      1    PeEG H0P      1     0.0877     1.0080
    26   opls_825      1    PeEG H0Q      1     0.0927     1.0080
    27   opls_826      1    PeEG H0R      1     0.0927     1.0080
    28   opls_827      1    PeEG H0S      1     0.0929     1.0080
```

```
    29   opls_828      1    PeEG H0T      1     0.0929     1.0080
    30   opls_829      1    PeEG H0U      1     0.0876     1.0080
    31   opls_830      1    PeEG H0V      1     0.0876     1.0080
    32   opls_831      1    PeEG H0W      1     0.0931     1.0080
    33   opls_832      1    PeEG H0X      2     0.0931     1.0080
    34   opls_833      1    PeEG H0Y      2     0.0945     1.0080
    35   opls_834      1    PeEG H0Z      2     0.0945     1.0080
    36   opls_835      1    PeEG H10      2     0.1061     1.0080
    37   opls_836      1    PeEG H11      2     0.1061     1.0080
    38   opls_837      1    PeEG H12      2     0.4236     1.0080
[ bonds ]
    2     1     1      0.1410 267776.000
    3     2     1      0.1529 224262.400
    4     3     1      0.1410 267776.000
    5     4     1      0.1410 267776.000
    6     5     1      0.1529 224262.400
    7     6     1      0.1410 267776.000
    8     7     1      0.1410 267776.000
    9     8     1      0.1529 224262.400
   10     9     1      0.1410 267776.000
   11    10     1      0.1410 267776.000
   12    11     1      0.1529 224262.400
   13    12     1      0.1410 267776.000
   14    13     1      0.1410 267776.000
   15    14     1      0.1529 224262.400
   16    15     1      0.1410 267776.000
   17     1     1      0.0945 462750.400
   18     2     1      0.1090 284512.000
   19     2     1      0.1090 284512.000
   20     3     1      0.1090 284512.000
   21     3     1      0.1090 284512.000
   22     5     1      0.1090 284512.000
   23     5     1      0.1090 284512.000
   24     6     1      0.1090 284512.000
   25     6     1      0.1090 284512.000
   26     8     1      0.1090 284512.000
   27     8     1      0.1090 284512.000
   28     9     1      0.1090 284512.000
   29     9     1      0.1090 284512.000
   30    11     1      0.1090 284512.000
   31    11     1      0.1090 284512.000
   32    12     1      0.1090 284512.000
   33    12     1      0.1090 284512.000
   34    14     1      0.1090 284512.000
   35    14     1      0.1090 284512.000
   36    15     1      0.1090 284512.000
   37    15     1      0.1090 284512.000
   38    16     1      0.0945 462750.400
[ angles ]
;  ai    aj    ak funct            c0            c1            c2            c3
    1     2     3     1    109.500    418.400
    2     3     4     1    109.500    418.400
    3     4     5     1    109.500    502.080
    4     5     6     1    109.500    418.400
    5     6     7     1    109.500    418.400
    6     7     8     1    109.500    502.080
    7     8     9     1    109.500    418.400
```

8	9	10	1	109.500	418.400
9	10	11	1	109.500	502.080
10	11	12	1	109.500	418.400
11	12	13	1	109.500	418.400
12	13	14	1	109.500	502.080
13	14	15	1	109.500	418.400
14	15	16	1	109.500	418.400
2	1	17	1	108.500	460.240
1	2	18	1	109.500	292.880
1	2	19	1	109.500	292.880
2	3	20	1	110.700	313.800
2	3	21	1	110.700	313.800
4	5	22	1	109.500	292.880
4	5	23	1	109.500	292.880
5	6	24	1	110.700	313.800
5	6	25	1	110.700	313.800
7	8	26	1	109.500	292.880
7	8	27	1	109.500	292.880
8	9	28	1	110.700	313.800
8	9	29	1	110.700	313.800
10	11	30	1	109.500	292.880
10	11	31	1	109.500	292.880
11	12	32	1	110.700	313.800
11	12	33	1	110.700	313.800
13	14	34	1	109.500	292.880
13	14	35	1	109.500	292.880
14	15	36	1	110.700	313.800
14	15	37	1	110.700	313.800
15	16	38	1	108.500	460.240
15	14	34	1	110.700	313.800
12	11	31	1	110.700	313.800
15	14	35	1	110.700	313.800
7	6	24	1	109.500	292.880
13	12	33	1	109.500	292.880
22	5	23	1	107.800	276.144
3	2	18	1	110.700	313.800
10	9	29	1	109.500	292.880
6	5	22	1	110.700	313.800
16	15	36	1	109.500	292.880
7	6	25	1	109.500	292.880
32	12	33	1	107.800	276.144
18	2	19	1	107.800	276.144
34	14	35	1	107.800	276.144
12	11	30	1	110.700	313.800
30	11	31	1	107.800	276.144
9	8	27	1	110.700	313.800
36	15	37	1	107.800	276.144
3	2	19	1	110.700	313.800
4	3	21	1	109.500	292.880
16	15	37	1	109.500	292.880
28	9	29	1	107.800	276.144
24	6	25	1	107.800	276.144
6	5	23	1	110.700	313.800
9	8	26	1	110.700	313.800
10	9	28	1	109.500	292.880
13	12	32	1	109.500	292.880
4	3	20	1	109.500	292.880

```
   26     8    27     1    107.800    276.144
   20     3    21     1    107.800    276.144
[ dihedrals ]
; IMPROPER DIHEDRAL ANGLES
;  ai    aj    ak    al funct            c0            c1            c2            c3
c4            c5
[ dihedrals ]
; PROPER DIHEDRAL ANGLES
;  ai    aj    ak    al funct            c0            c1            c2            c3
c4            c5
   12   11   10    9        3       1.715   2.845   1.046  -5.607  -0.000   0.000
    6    5    4    3        3       1.715   2.845   1.046  -5.607  -0.000   0.000
    9    8    7    6        3       1.715   2.845   1.046  -5.607  -0.000   0.000
   15   14   13   12        3       1.715   2.845   1.046  -5.607  -0.000   0.000
    5    4    3    2        3       1.715   2.845   1.046  -5.607  -0.000   0.000
    8    7    6    5        3       1.715   2.845   1.046  -5.607  -0.000   0.000
   11   10    9    8        3       1.715   2.845   1.046  -5.607  -0.000   0.000
   14   13   12   11        3       1.715   2.845   1.046  -5.607  -0.000   0.000
   25    6    5   22        3       0.628   1.883   0.000  -2.510  -0.000   0.000
   28    9    8   27        3       0.628   1.883   0.000  -2.510  -0.000   0.000
   29    9    8   27        3       0.628   1.883   0.000  -2.510  -0.000   0.000
   33   12   11   31        3       0.628   1.883   0.000  -2.510  -0.000   0.000
   29    9    8   26        3       0.628   1.883   0.000  -2.510  -0.000   0.000
   20    3    2   18        3       0.628   1.883   0.000  -2.510  -0.000   0.000
   24    6    5   22        3       0.628   1.883   0.000  -2.510  -0.000   0.000
   36   15   14   34        3       0.628   1.883   0.000  -2.510  -0.000   0.000
   36   15   14   35        3       0.628   1.883   0.000  -2.510  -0.000   0.000
   33   12   11   30        3       0.628   1.883   0.000  -2.510  -0.000   0.000
   21    3    2   19        3       0.628   1.883   0.000  -2.510  -0.000   0.000
   20    3    2   19        3       0.628   1.883   0.000  -2.510  -0.000   0.000
   37   15   14   34        3       0.628   1.883   0.000  -2.510  -0.000   0.000
   37   15   14   35        3       0.628   1.883   0.000  -2.510  -0.000   0.000
   21    3    2   18        3       0.628   1.883   0.000  -2.510  -0.000   0.000
   32   12   11   30        3       0.628   1.883   0.000  -2.510  -0.000   0.000
   24    6    5   23        3       0.628   1.883   0.000  -2.510  -0.000   0.000
   32   12   11   31        3       0.628   1.883   0.000  -2.510  -0.000   0.000
   25    6    5   23        3       0.628   1.883   0.000  -2.510  -0.000   0.000
   28    9    8   26        3       0.628   1.883   0.000  -2.510  -0.000   0.000
   34   14   15   16        3       0.979   2.937   0.000  -3.916  -0.000   0.000
   35   14   15   16        3       0.979   2.937   0.000  -3.916  -0.000   0.000
   20    3    2    1        3       0.979   2.937   0.000  -3.916  -0.000   0.000
   21    3    2    1        3       0.979   2.937   0.000  -3.916  -0.000   0.000
   19    2    3    4        3       0.979   2.937   0.000  -3.916  -0.000   0.000
   28    9    8    7        3       0.979   2.937   0.000  -3.916  -0.000   0.000
   30   11   12   13        3       0.979   2.937   0.000  -3.916  -0.000   0.000
   22    5    6    7        3       0.979   2.937   0.000  -3.916  -0.000   0.000
   33   12   11   10        3       0.979   2.937   0.000  -3.916  -0.000   0.000
   31   11   12   13        3       0.979   2.937   0.000  -3.916  -0.000   0.000
   24    6    5    4        3       0.979   2.937   0.000  -3.916  -0.000   0.000
   37   15   14   13        3       0.979   2.937   0.000  -3.916  -0.000   0.000
   36   15   14   13        3       0.979   2.937   0.000  -3.916  -0.000   0.000
   32   12   11   10        3       0.979   2.937   0.000  -3.916  -0.000   0.000
   23    5    6    7        3       0.979   2.937   0.000  -3.916  -0.000   0.000
   26    8    9   10        3       0.979   2.937   0.000  -3.916  -0.000   0.000
   18    2    3    4        3       0.979   2.937   0.000  -3.916  -0.000   0.000
   27    8    9   10        3       0.979   2.937   0.000  -3.916  -0.000   0.000
   29    9    8    7        3       0.979   2.937   0.000  -3.916  -0.000   0.000
```

25	6	5	4	3	0.979	2.937	0.000	-3.916	-0.000	0.000
18	2	1	17	3	0.736	2.209	0.000	-2.946	-0.000	0.000
19	2	1	17	3	0.736	2.209	0.000	-2.946	-0.000	0.000
25	6	7	8	3	1.590	4.770	0.000	-6.360	-0.000	0.000
35	14	13	12	3	1.590	4.770	0.000	-6.360	-0.000	0.000
33	12	13	14	3	1.590	4.770	0.000	-6.360	-0.000	0.000
31	11	10	9	3	1.590	4.770	0.000	-6.360	-0.000	0.000
22	5	4	3	3	1.590	4.770	0.000	-6.360	-0.000	0.000
28	9	10	11	3	1.590	4.770	0.000	-6.360	-0.000	0.000
27	8	7	6	3	1.590	4.770	0.000	-6.360	-0.000	0.000
24	6	7	8	3	1.590	4.770	0.000	-6.360	-0.000	0.000
34	14	13	12	3	1.590	4.770	0.000	-6.360	-0.000	0.000
23	5	4	3	3	1.590	4.770	0.000	-6.360	-0.000	0.000
26	8	7	6	3	1.590	4.770	0.000	-6.360	-0.000	0.000
32	12	13	14	3	1.590	4.770	0.000	-6.360	-0.000	0.000
21	3	4	5	3	1.590	4.770	0.000	-6.360	-0.000	0.000
29	9	10	11	3	1.590	4.770	0.000	-6.360	-0.000	0.000
20	3	4	5	3	1.590	4.770	0.000	-6.360	-0.000	0.000
30	11	10	9	3	1.590	4.770	0.000	-6.360	-0.000	0.000
38	16	15	14	3	-0.444	3.833	0.728	-4.117	-0.000	0.000
17	1	2	3	3	-0.444	3.833	0.728	-4.117	-0.000	0.000
38	16	15	37	3	0.736	2.209	0.000	-2.946	-0.000	0.000
38	16	15	36	3	0.736	2.209	0.000	-2.946	-0.000	0.000
16	15	14	13	3	9.035	-9.035	0.000	-0.000	-0.000	0.000
4	3	2	1	3	9.035	-9.035	0.000	-0.000	-0.000	0.000
13	12	11	10	3	-1.151	1.151	0.000	-0.000	-0.000	0.000
10	9	8	7	3	-1.151	1.151	0.000	-0.000	-0.000	0.000
7	6	5	4	3	-1.151	1.151	0.000	-0.000	-0.000	0.000

[pairs]

1	4	1
2	5	1
3	6	1
4	7	1
5	8	1
6	9	1
7	10	1
8	11	1
3	17	1
9	12	1
1	20	1
4	18	1
1	21	1
10	13	1
4	19	1
11	14	1
5	20	1
3	22	1
5	21	1
3	23	1
12	15	1
4	24	1
13	16	1
7	22	1
4	25	1
7	23	1
8	24	1
6	26	1

```
     8    25    1
     6    27    1
    17    18    1
     7    28    1
    17    19    1
    10    26    1
     7    29    1
    10    27    1
    18    20    1
    19    20    1
    18    21    1
    11    28    1
     9    30    1
    19    21    1
    11    29    1
     9    31    1
    10    32    1
    13    30    1
    10    33    1
    13    31    1
    22    24    1
    14    32    1
    12    34    1
    23    24    1
    22    25    1
    14    33    1
    12    35    1
    23    25    1
    13    36    1
    16    34    1
    13    37    1
    16    35    1
    14    38    1
    26    28    1
    27    28    1
    26    29    1
    27    29    1
    30    32    1
    31    32    1
    30    33    1
    31    33    1
    34    36    1
    35    36    1
    34    37    1
    35    37    1
    36    38    1
    37    38    1
```

Hexaethylene Glycol: Include topology file "HexEG.itp"

```
; GENERATED BY LigParGen Server
; Jorgensen Lab @ Yale University
;
[ atomtypes ]
  opls_814  C814    12.0110     0.000    A    3.50000E-01   2.76144E-01
  opls_831  H831     1.0080     0.000    A    2.50000E-01   1.25520E-01
```

```
  opls_803  O803    15.9990     0.000    A    2.90000E-01   5.85760E-01
  opls_824  H824     1.0080     0.000    A    2.50000E-01   1.25520E-01
  opls_809  O809    15.9990     0.000    A    2.90000E-01   5.85760E-01
  opls_800  O800    15.9990     0.000    A    3.12000E-01   7.11280E-01
  opls_836  H836     1.0080     0.000    A    2.50000E-01   1.25520E-01
  opls_834  H834     1.0080     0.000    A    2.50000E-01   1.25520E-01
  opls_828  H828     1.0080     0.000    A    2.50000E-01   1.25520E-01
  opls_812  O812    15.9990     0.000    A    2.90000E-01   5.85760E-01
  opls_806  O806    15.9990     0.000    A    2.90000E-01   5.85760E-01
  opls_802  C802    12.0110     0.000    A    3.50000E-01   2.76144E-01
  opls_827  H827     1.0080     0.000    A    2.50000E-01   1.25520E-01
  opls_801  C801    12.0110     0.000    A    3.50000E-01   2.76144E-01
  opls_838  H838     1.0080     0.000    A    2.50000E-01   1.25520E-01
  opls_818  O818    15.9990     0.000    A    3.12000E-01   7.11280E-01
  opls_807  C807    12.0110     0.000    A    3.50000E-01   2.76144E-01
  opls_830  H830     1.0080     0.000    A    2.50000E-01   1.25520E-01
  opls_810  C810    12.0110     0.000    A    3.50000E-01   2.76144E-01
  opls_842  H842     1.0080     0.000    A    2.50000E-01   1.25520E-01
  opls_820  H820     1.0080     0.000    A    2.50000E-01   1.25520E-01
  opls_819  H819     1.0080     0.000    A    0.00000E+00   0.00000E+00
  opls_821  H821     1.0080     0.000    A    2.50000E-01   1.25520E-01
  opls_815  O815    15.9990     0.000    A    2.90000E-01   5.85760E-01
  opls_832  H832     1.0080     0.000    A    2.50000E-01   1.25520E-01
  opls_844  H844     1.0080     0.000    A    0.00000E+00   0.00000E+00
  opls_805  C805    12.0110     0.000    A    3.50000E-01   2.76144E-01
  opls_816  C816    12.0110     0.000    A    3.50000E-01   2.76144E-01
  opls_811  C811    12.0110     0.000    A    3.50000E-01   2.76144E-01
  opls_823  H823     1.0080     0.000    A    2.50000E-01   1.25520E-01
  opls_813  C813    12.0110     0.000    A    3.50000E-01   2.76144E-01
  opls_826  H826     1.0080     0.000    A    2.50000E-01   1.25520E-01
  opls_835  H835     1.0080     0.000    A    2.50000E-01   1.25520E-01
  opls_841  H841     1.0080     0.000    A    2.50000E-01   1.25520E-01
  opls_825  H825     1.0080     0.000    A    2.50000E-01   1.25520E-01
  opls_804  C804    12.0110     0.000    A    3.50000E-01   2.76144E-01
  opls_833  H833     1.0080     0.000    A    2.50000E-01   1.25520E-01
  opls_839  H839     1.0080     0.000    A    2.50000E-01   1.25520E-01
  opls_817  C817    12.0110     0.000    A    3.50000E-01   2.76144E-01
  opls_808  C808    12.0110     0.000    A    3.50000E-01   2.76144E-01
  opls_843  H843     1.0080     0.000    A    2.50000E-01   1.25520E-01
  opls_840  H840     1.0080     0.000    A    2.50000E-01   1.25520E-01
  opls_837  H837     1.0080     0.000    A    2.50000E-01   1.25520E-01
  opls_829  H829     1.0080     0.000    A    2.50000E-01   1.25520E-01
  opls_822  H822     1.0080     0.000    A    2.50000E-01   1.25520E-01
[ moleculetype ]
; Name               nrexcl
HexEG                  3
[ atoms ]
;   nr       type  resnr residue  atom   cgnr     charge       mass
     1   opls_800      1    HexEG  O00      1    -0.6887    15.9990
     2   opls_801      1    HexEG  C01      1     0.1085    12.0110
     3   opls_802      1    HexEG  C02      1     0.0078    12.0110
     4   opls_803      1    HexEG  O03      1    -0.3847    15.9990
     5   opls_804      1    HexEG  C04      1     0.0075    12.0110
     6   opls_805      1    HexEG  C05      1     0.0085    12.0110
```

```
     7   opls_806      1    HexEG  O06      1     -0.384    15.9990
     8   opls_807      1    HexEG  C07      1     0.0086    12.0110
     9   opls_808      1    HexEG  C08      1     0.0085    12.0110
    10   opls_809      1    HexEG  O09      1     -0.384    15.9990
    11   opls_810      1    HexEG  C0A      1     0.0088    12.0110
    12   opls_811      1    HexEG  C0B      1     0.0084    12.0110
    13   opls_812      1    HexEG  O0C      1    -0.3844    15.9990
    14   opls_813      1    HexEG  C0D      1     0.0085    12.0110
    15   opls_814      1    HexEG  C0E      1     0.0069    12.0110
    16   opls_815      1    HexEG  O0F      1     -0.399    15.9990
    17   opls_816      1    HexEG  C0G      1     -0.041    12.0110
    18   opls_817      1    HexEG  C0H      1     0.1082    12.0110
    19   opls_818      1    HexEG  O0I      1    -0.6858    15.9990
    20   opls_819      1    HexEG  H0J      1     0.4176     1.0080
    21   opls_820      1    HexEG  H0K      1     0.0822     1.0080
    22   opls_821      1    HexEG  H0M      1     0.0822     1.0080
    23   opls_822      1    HexEG  H0N      1     0.0926     1.0080
    24   opls_823      1    HexEG  H0O      1     0.0926     1.0080
    25   opls_824      1    HexEG  H0P      1     0.0917     1.0080
    26   opls_825      1    HexEG  H0Q      1     0.0917     1.0080
    27   opls_826      1    HexEG  H0R      1     0.0917     1.0080
    28   opls_827      1    HexEG  H0S      1     0.0917     1.0080
    29   opls_828      1    HexEG  H0T      1     0.0916     1.0080
    30   opls_829      1    HexEG  H0U      1     0.0916     1.0080
    31   opls_830      1    HexEG  H0V      1     0.0918     1.0080
    32   opls_831      1    HexEG  H0W      1     0.0918     1.0080
    33   opls_832      1    HexEG  H0X      2     0.0917     1.0080
    34   opls_833      1    HexEG  H0Y      2     0.0917     1.0080
    35   opls_834      1    HexEG  H0Z      2     0.0919     1.0080
    36   opls_835      1    HexEG  H10      2     0.0919     1.0080
    37   opls_836      1    HexEG  H11      2     0.0915     1.0080
    38   opls_837      1    HexEG  H12      2     0.0915     1.0080
    39   opls_838      1    HexEG  H13      2      0.092     1.0080
    40   opls_839      1    HexEG  H14      2      0.092     1.0080
    41   opls_840      1    HexEG  H15      2     0.0946     1.0080
    42   opls_841      1    HexEG  H16      2     0.0946     1.0080
    43   opls_842      1    HexEG  H17      2     0.1068     1.0080
    44   opls_843      1    HexEG  H18      2     0.1068     1.0080
    45   opls_844      1    HexEG  H19      2     0.4236     1.0080
[ bonds ]
    2     1     1      0.1410 267776.000
    3     2     1      0.1529 224262.400
    4     3     1      0.1410 267776.000
    5     4     1      0.1410 267776.000
    6     5     1      0.1529 224262.400
    7     6     1      0.1410 267776.000
    8     7     1      0.1410 267776.000
    9     8     1      0.1529 224262.400
   10     9     1      0.1410 267776.000
   11    10     1      0.1410 267776.000
   12    11     1      0.1529 224262.400
   13    12     1      0.1410 267776.000
   14    13     1      0.1410 267776.000
   15    14     1      0.1529 224262.400
```

```
   16    15     1      0.1410 267776.000
   17    16     1      0.1410 267776.000
   18    17     1      0.1529 224262.400
   19    18     1      0.1410 267776.000
   20     1     1      0.0945 462750.400
   21     2     1      0.1090 284512.000
   22     2     1      0.1090 284512.000
   23     3     1      0.1090 284512.000
   24     3     1      0.1090 284512.000
   25     5     1      0.1090 284512.000
   26     5     1      0.1090 284512.000
   27     6     1      0.1090 284512.000
   28     6     1      0.1090 284512.000
   29     8     1      0.1090 284512.000
   30     8     1      0.1090 284512.000
   31     9     1      0.1090 284512.000
   32     9     1      0.1090 284512.000
   33    11     1      0.1090 284512.000
   34    11     1      0.1090 284512.000
   35    12     1      0.1090 284512.000
   36    12     1      0.1090 284512.000
   37    14     1      0.1090 284512.000
   38    14     1      0.1090 284512.000
   39    15     1      0.1090 284512.000
   40    15     1      0.1090 284512.000
   41    17     1      0.1090 284512.000
   42    17     1      0.1090 284512.000
   43    18     1      0.1090 284512.000
   44    18     1      0.1090 284512.000
   45    19     1      0.0945 462750.400

[ angles ]
;  ai    aj    ak funct            c0            c1            c2            c3
    1     2     3     1    109.500    418.400
    2     3     4     1    109.500    418.400
    3     4     5     1    109.500    502.080
    4     5     6     1    109.500    418.400
    5     6     7     1    109.500    418.400
    6     7     8     1    109.500    502.080
    7     8     9     1    109.500    418.400
    8     9    10     1    109.500    418.400
    9    10    11     1    109.500    502.080
   10    11    12     1    109.500    418.400
   11    12    13     1    109.500    418.400
   12    13    14     1    109.500    502.080
   13    14    15     1    109.500    418.400
   14    15    16     1    109.500    418.400
   15    16    17     1    109.500    502.080
   16    17    18     1    109.500    418.400
   17    18    19     1    109.500    418.400
    2     1    20     1    108.500    460.240
    1     2    21     1    109.500    292.880
    1     2    22     1    109.500    292.880
    2     3    23     1    110.700    313.800
```

2	3	24	1	110.700	313.800
4	5	25	1	109.500	292.880
4	5	26	1	109.500	292.880
5	6	27	1	110.700	313.800
5	6	28	1	110.700	313.800
7	8	29	1	109.500	292.880
7	8	30	1	109.500	292.880
8	9	31	1	110.700	313.800
8	9	32	1	110.700	313.800
10	11	33	1	109.500	292.880
10	11	34	1	109.500	292.880
11	12	35	1	110.700	313.800
11	12	36	1	110.700	313.800
13	14	37	1	109.500	292.880
13	14	38	1	109.500	292.880
14	15	39	1	110.700	313.800
14	15	40	1	110.700	313.800
16	17	41	1	109.500	292.880
16	17	42	1	109.500	292.880
17	18	43	1	110.700	313.800
17	18	44	1	110.700	313.800
18	19	45	1	108.500	460.240
9	8	30	1	110.700	313.800
18	17	41	1	110.700	313.800
10	9	32	1	109.500	292.880
7	6	28	1	109.500	292.880
3	2	21	1	110.700	313.800
27	6	28	1	107.800	276.144
9	8	29	1	110.700	313.800
4	3	23	1	109.500	292.880
37	14	38	1	107.800	276.144
12	11	33	1	110.700	313.800
13	12	36	1	109.500	292.880
23	3	24	1	107.800	276.144
43	18	44	1	107.800	276.144
3	2	22	1	110.700	313.800
13	12	35	1	109.500	292.880
16	15	39	1	109.500	292.880
35	12	36	1	107.800	276.144
15	14	37	1	110.700	313.800
15	14	38	1	110.700	313.800
39	15	40	1	107.800	276.144
16	15	40	1	109.500	292.880
19	18	43	1	109.500	292.880
33	11	34	1	107.800	276.144
7	6	27	1	109.500	292.880
25	5	26	1	107.800	276.144
19	18	44	1	109.500	292.880
4	3	24	1	109.500	292.880
10	9	31	1	109.500	292.880
12	11	34	1	110.700	313.800
41	17	42	1	107.800	276.144
6	5	26	1	110.700	313.800
31	9	32	1	107.800	276.144

```
     6      5    25      1    110.700    313.800
    18     17    42      1    110.700    313.800
    29      8    30      1    107.800    276.144
    21      2    22      1    107.800    276.144

[ dihedrals ]
; IMPROPER DIHEDRAL ANGLES
;  ai    aj    ak    al funct            c0            c1            c2
c3            c4            c5

[ dihedrals ]
; PROPER DIHEDRAL ANGLES
;  ai    aj    ak    al funct            c0            c1            c2
c3            c4            c5
    12    11    10     9        3        1.715    2.845    1.046   -5.607   -0.000    0.000
     6     5     4     3        3        1.715    2.845    1.046   -5.607   -0.000    0.000
    18    17    16    15        3        1.715    2.845    1.046   -5.607   -0.000    0.000
     9     8     7     6        3        1.715    2.845    1.046   -5.607   -0.000    0.000
    15    14    13    12        3        1.715    2.845    1.046   -5.607   -0.000    0.000
     5     4     3     2        3        1.715    2.845    1.046   -5.607   -0.000    0.000
     8     7     6     5        3        1.715    2.845    1.046   -5.607   -0.000    0.000
    17    16    15    14        3        1.715    2.845    1.046   -5.607   -0.000    0.000
    11    10     9     8        3        1.715    2.845    1.046   -5.607   -0.000    0.000
    14    13    12    11        3        1.715    2.845    1.046   -5.607   -0.000    0.000
    32     9     8    30        3        0.628    1.883    0.000   -2.510   -0.000    0.000
    35    12    11    34        3        0.628    1.883    0.000   -2.510   -0.000    0.000
    43    18    17    42        3        0.628    1.883    0.000   -2.510   -0.000    0.000
    31     9     8    29        3        0.628    1.883    0.000   -2.510   -0.000    0.000
    40    15    14    38        3        0.628    1.883    0.000   -2.510   -0.000    0.000
    44    18    17    42        3        0.628    1.883    0.000   -2.510   -0.000    0.000
    27     6     5    25        3        0.628    1.883    0.000   -2.510   -0.000    0.000
    24     3     2    21        3        0.628    1.883    0.000   -2.510   -0.000    0.000
    23     3     2    22        3        0.628    1.883    0.000   -2.510   -0.000    0.000
    40    15    14    37        3        0.628    1.883    0.000   -2.510   -0.000    0.000
    44    18    17    41        3        0.628    1.883    0.000   -2.510   -0.000    0.000
    39    15    14    37        3        0.628    1.883    0.000   -2.510   -0.000    0.000
    35    12    11    33        3        0.628    1.883    0.000   -2.510   -0.000    0.000
    43    18    17    41        3        0.628    1.883    0.000   -2.510   -0.000    0.000
    39    15    14    38        3        0.628    1.883    0.000   -2.510   -0.000    0.000
    24     3     2    22        3        0.628    1.883    0.000   -2.510   -0.000    0.000
    23     3     2    21        3        0.628    1.883    0.000   -2.510   -0.000    0.000
    32     9     8    29        3        0.628    1.883    0.000   -2.510   -0.000    0.000
    28     6     5    26        3        0.628    1.883    0.000   -2.510   -0.000    0.000
    31     9     8    30        3        0.628    1.883    0.000   -2.510   -0.000    0.000
    36    12    11    34        3        0.628    1.883    0.000   -2.510   -0.000    0.000
    36    12    11    33        3        0.628    1.883    0.000   -2.510   -0.000    0.000
    27     6     5    26        3        0.628    1.883    0.000   -2.510   -0.000    0.000
    28     6     5    25        3        0.628    1.883    0.000   -2.510   -0.000    0.000
    23     3     2     1        3        0.979    2.937    0.000   -3.916   -0.000    0.000
    41    17    18    19        3        0.979    2.937    0.000   -3.916   -0.000    0.000
    24     3     2     1        3        0.979    2.937    0.000   -3.916   -0.000    0.000
    42    17    18    19        3        0.979    2.937    0.000   -3.916   -0.000    0.000
    27     6     5     4        3        0.979    2.937    0.000   -3.916   -0.000    0.000
    29     8     9    10        3        0.979    2.937    0.000   -3.916   -0.000    0.000
```

38	14	15	16	3	0.979	2.937	0.000	-3.916	-0.000	0.000
32	9	8	7	3	0.979	2.937	0.000	-3.916	-0.000	0.000
31	9	8	7	3	0.979	2.937	0.000	-3.916	-0.000	0.000
22	2	3	4	3	0.979	2.937	0.000	-3.916	-0.000	0.000
36	12	11	10	3	0.979	2.937	0.000	-3.916	-0.000	0.000
33	11	12	13	3	0.979	2.937	0.000	-3.916	-0.000	0.000
26	5	6	7	3	0.979	2.937	0.000	-3.916	-0.000	0.000
34	11	12	13	3	0.979	2.937	0.000	-3.916	-0.000	0.000
21	2	3	4	3	0.979	2.937	0.000	-3.916	-0.000	0.000
43	18	17	16	3	0.979	2.937	0.000	-3.916	-0.000	0.000
44	18	17	16	3	0.979	2.937	0.000	-3.916	-0.000	0.000
37	14	15	16	3	0.979	2.937	0.000	-3.916	-0.000	0.000
39	15	14	13	3	0.979	2.937	0.000	-3.916	-0.000	0.000
35	12	11	10	3	0.979	2.937	0.000	-3.916	-0.000	0.000
28	6	5	4	3	0.979	2.937	0.000	-3.916	-0.000	0.000
25	5	6	7	3	0.979	2.937	0.000	-3.916	-0.000	0.000
30	8	9	10	3	0.979	2.937	0.000	-3.916	-0.000	0.000
40	15	14	13	3	0.979	2.937	0.000	-3.916	-0.000	0.000
22	2	1	20	3	0.736	2.209	0.000	-2.946	-0.000	0.000
21	2	1	20	3	0.736	2.209	0.000	-2.946	-0.000	0.000
33	11	10	9	3	1.590	4.770	0.000	-6.360	-0.000	0.000
23	3	4	5	3	1.590	4.770	0.000	-6.360	-0.000	0.000
38	14	13	12	3	1.590	4.770	0.000	-6.360	-0.000	0.000
29	8	7	6	3	1.590	4.770	0.000	-6.360	-0.000	0.000
39	15	16	17	3	1.590	4.770	0.000	-6.360	-0.000	0.000
36	12	13	14	3	1.590	4.770	0.000	-6.360	-0.000	0.000
34	11	10	9	3	1.590	4.770	0.000	-6.360	-0.000	0.000
30	8	7	6	3	1.590	4.770	0.000	-6.360	-0.000	0.000
32	9	10	11	3	1.590	4.770	0.000	-6.360	-0.000	0.000
25	5	4	3	3	1.590	4.770	0.000	-6.360	-0.000	0.000
28	6	7	8	3	1.590	4.770	0.000	-6.360	-0.000	0.000
27	6	7	8	3	1.590	4.770	0.000	-6.360	-0.000	0.000
42	17	16	15	3	1.590	4.770	0.000	-6.360	-0.000	0.000
26	5	4	3	3	1.590	4.770	0.000	-6.360	-0.000	0.000
24	3	4	5	3	1.590	4.770	0.000	-6.360	-0.000	0.000
41	17	16	15	3	1.590	4.770	0.000	-6.360	-0.000	0.000
31	9	10	11	3	1.590	4.770	0.000	-6.360	-0.000	0.000
35	12	13	14	3	1.590	4.770	0.000	-6.360	-0.000	0.000
40	15	16	17	3	1.590	4.770	0.000	-6.360	-0.000	0.000
37	14	13	12	3	1.590	4.770	0.000	-6.360	-0.000	0.000
20	1	2	3	3	-0.444	3.833	0.728	-4.117	-0.000	0.000
45	19	18	17	3	-0.444	3.833	0.728	-4.117	-0.000	0.000
45	19	18	44	3	0.736	2.209	0.000	-2.946	-0.000	0.000
45	19	18	43	3	0.736	2.209	0.000	-2.946	-0.000	0.000
19	18	17	16	3	9.035	-9.035	0.000	-0.000	-0.000	0.000
4	3	2	1	3	9.035	-9.035	0.000	-0.000	-0.000	0.000
13	12	11	10	3	-1.151	1.151	0.000	-0.000	-0.000	0.000
10	9	8	7	3	-1.151	1.151	0.000	-0.000	-0.000	0.000
7	6	5	4	3	-1.151	1.151	0.000	-0.000	-0.000	0.000
16	15	14	13	3	-1.151	1.151	0.000	-0.000	-0.000	0.000

```
[ pairs ]
     1     4    1
     2     5    1
```

3	6	1
4	7	1
5	8	1
6	9	1
7	10	1
8	11	1
9	12	1
10	13	1
3	20	1
1	23	1
11	14	1
4	21	1
1	24	1
4	22	1
12	15	1
5	23	1
3	25	1
13	16	1
5	24	1
3	26	1
14	17	1
4	27	1
7	25	1
4	28	1
15	18	1
7	26	1
16	19	1
8	27	1
6	29	1
8	28	1
6	30	1
7	31	1
10	29	1
7	32	1
10	30	1
20	21	1
20	22	1
11	31	1
9	33	1
11	32	1
9	34	1
21	23	1
22	23	1
21	24	1
10	35	1
22	24	1
13	33	1
10	36	1
13	34	1
14	35	1
12	37	1
14	36	1
12	38	1
25	27	1

```
    13    39    1
    26    27    1
    25    28    1
    16    37    1
    13    40    1
    26    28    1
    16    38    1
    17    39    1
    15    41    1
    17    40    1
    15    42    1
    16    43    1
    29    31    1
    19    41    1
    16    44    1
    30    31    1
    29    32    1
    19    42    1
    30    32    1
    17    45    1
    33    35    1
    34    35    1
    33    36    1
    34    36    1
    37    39    1
    38    39    1
    37    40    1
    38    40    1
    41    43    1
    42    43    1
    41    44    1
    42    44    1
    43    45    1
    44    45    1
```

Heptaethylene Glycol: Include topology file "HepEG.itp"

```
;
; GENERATED BY LigParGen Server
; Jorgensen Lab @ Yale University
;
[ atomtypes ]
  opls_814  C814    12.0110     0.000    A    3.50000E-01   2.76144E-01
  opls_831  H831     1.0080     0.000    A    2.50000E-01   1.25520E-01
  opls_803  O803    15.9990     0.000    A    2.90000E-01   5.85760E-01
  opls_824  H824     1.0080     0.000    A    2.50000E-01   1.25520E-01
  opls_809  O809    15.9990     0.000    A    2.90000E-01   5.85760E-01
  opls_851  H851     1.0080     0.000    A    0.00000E+00   0.00000E+00
  opls_848  H848     1.0080     0.000    A    2.50000E-01   1.25520E-01
  opls_800  O800    15.9990     0.000    A    3.12000E-01   7.11280E-01
  opls_836  H836     1.0080     0.000    A    2.50000E-01   1.25520E-01
  opls_834  H834     1.0080     0.000    A    2.50000E-01   1.25520E-01
```

```
  opls_828  H828     1.0080     0.000    A    2.50000E-01   1.25520E-01
  opls_812  O812    15.9990     0.000    A    2.90000E-01   5.85760E-01
  opls_806  O806    15.9990     0.000    A    2.90000E-01   5.85760E-01
  opls_802  C802    12.0110     0.000    A    3.50000E-01   2.76144E-01
  opls_827  H827     1.0080     0.000    A    2.50000E-01   1.25520E-01
  opls_801  C801    12.0110     0.000    A    3.50000E-01   2.76144E-01
  opls_838  H838     1.0080     0.000    A    2.50000E-01   1.25520E-01
  opls_844  H844     1.0080     0.000    A    2.50000E-01   1.25520E-01
  opls_807  C807    12.0110     0.000    A    3.50000E-01   2.76144E-01
  opls_821  O821    15.9990     0.000    A    3.12000E-01   7.11280E-01
  opls_830  H830     1.0080     0.000    A    2.50000E-01   1.25520E-01
  opls_810  C810    12.0110     0.000    A    3.50000E-01   2.76144E-01
  opls_842  H842     1.0080     0.000    A    2.50000E-01   1.25520E-01
  opls_826  H826     1.0080     0.000    A    2.50000E-01   1.25520E-01
  opls_837  H837     1.0080     0.000    A    2.50000E-01   1.25520E-01
  opls_829  H829     1.0080     0.000    A    2.50000E-01   1.25520E-01
  opls_815  O815    15.9990     0.000    A    2.90000E-01   5.85760E-01
  opls_832  H832     1.0080     0.000    A    2.50000E-01   1.25520E-01
  opls_839  H839     1.0080     0.000    A    2.50000E-01   1.25520E-01
  opls_805  C805    12.0110     0.000    A    3.50000E-01   2.76144E-01
  opls_846  H846     1.0080     0.000    A    2.50000E-01   1.25520E-01
  opls_816  C816    12.0110     0.000    A    3.50000E-01   2.76144E-01
  opls_811  C811    12.0110     0.000    A    3.50000E-01   2.76144E-01
  opls_823  H823     1.0080     0.000    A    2.50000E-01   1.25520E-01
  opls_819  C819    12.0110     0.000    A    3.50000E-01   2.76144E-01
  opls_820  C820    12.0110     0.000    A    3.50000E-01   2.76144E-01
  opls_835  H835     1.0080     0.000    A    2.50000E-01   1.25520E-01
  opls_841  H841     1.0080     0.000    A    2.50000E-01   1.25520E-01
  opls_825  H825     1.0080     0.000    A    2.50000E-01   1.25520E-01
  opls_804  C804    12.0110     0.000    A    3.50000E-01   2.76144E-01
  opls_833  H833     1.0080     0.000    A    2.50000E-01   1.25520E-01
  opls_822  H822     1.0080     0.000    A    0.00000E+00   0.00000E+00
  opls_817  C817    12.0110     0.000    A    3.50000E-01   2.76144E-01
  opls_808  C808    12.0110     0.000    A    3.50000E-01   2.76144E-01
  opls_843  H843     1.0080     0.000    A    2.50000E-01   1.25520E-01
  opls_840  H840     1.0080     0.000    A    2.50000E-01   1.25520E-01
  opls_849  H849     1.0080     0.000    A    2.50000E-01   1.25520E-01
  opls_813  C813    12.0110     0.000    A    3.50000E-01   2.76144E-01
  opls_847  H847     1.0080     0.000    A    2.50000E-01   1.25520E-01
  opls_850  H850     1.0080     0.000    A    2.50000E-01   1.25520E-01
  opls_845  H845     1.0080     0.000    A    2.50000E-01   1.25520E-01
  opls_818  O818    15.9990     0.000    A    2.90000E-01   5.85760E-01
[ moleculetype ]
; Name               nrexcl
HepEG                  3
[ atoms ]
;   nr       type  resnr residue  atom   cgnr     charge       mass
     1   opls_800      1    HepEG  O00      1    -0.6886    15.9990
     2   opls_801      1    HepEG  C01      1     0.1082    12.0110
     3   opls_802      1    HepEG  C02      1     0.0078    12.0110
     4   opls_803      1    HepEG  O03      1    -0.3844    15.9990
     5   opls_804      1    HepEG  C04      1     0.0073    12.0110
     6   opls_805      1    HepEG  C05      1     0.0085    12.0110
     7   opls_806      1    HepEG  O06      1    -0.3857    15.9990
     8   opls_807      1    HepEG  C07      1     0.0089    12.0110
     9   opls_808      1    HepEG  C08      1      0.005    12.0110
    10   opls_809      1    HepEG  O09      1     -0.376    15.9990
```

```
    11   opls_810      1    HepEG  C0A      1     0.0105    12.0110
    12   opls_811      1    HepEG  C0B      1     0.0107    12.0110
    13   opls_812      1    HepEG  O0C      1    -0.3758    15.9990
    14   opls_813      1    HepEG  C0D      1     0.0054    12.0110
    15   opls_814      1    HepEG  C0E      1     0.0086    12.0110
    16   opls_815      1    HepEG  O0F      1     -0.386    15.9990
    17   opls_816      1    HepEG  C0G      1     0.0087    12.0110
    18   opls_817      1    HepEG  C0H      1     0.0064    12.0110
    19   opls_818      1    HepEG  O0I      1    -0.3984    15.9990
    20   opls_819      1    HepEG  C0J      1     -0.041    12.0110
    21   opls_820      1    HepEG  C0K      1     0.1082    12.0110
    22   opls_821      1    HepEG  O0M      1    -0.6861    15.9990
    23   opls_822      1    HepEG  H0N      1     0.4172     1.0080
    24   opls_823      1    HepEG  H0O      1     0.0822     1.0080
    25   opls_824      1    HepEG  H0P      1     0.0822     1.0080
    26   opls_825      1    HepEG  H0Q      1     0.0924     1.0080
    27   opls_826      1    HepEG  H0R      1     0.0924     1.0080
    28   opls_827      1    HepEG  H0S      1     0.0912     1.0080
    29   opls_828      1    HepEG  H0T      1     0.0912     1.0080
    30   opls_829      1    HepEG  H0U      1     0.0913     1.0080
    31   opls_830      1    HepEG  H0V      1     0.0913     1.0080
    32   opls_831      1    HepEG  H0W      1     0.0924     1.0080
    33   opls_832      1    HepEG  H0X      2     0.0924     1.0080
    34   opls_833      1    HepEG  H0Y      2     0.0881     1.0080
    35   opls_834      1    HepEG  H0Z      2     0.0881     1.0080
    36   opls_835      1    HepEG  H10      2     0.0931     1.0080
    37   opls_836      1    HepEG  H11      2     0.0931     1.0080
    38   opls_837      1    HepEG  H12      2     0.0929     1.0080
    39   opls_838      1    HepEG  H13      2     0.0929     1.0080
    40   opls_839      1    HepEG  H14      2     0.0878     1.0080
    41   opls_840      1    HepEG  H15      2     0.0878     1.0080
    42   opls_841      1    HepEG  H16      2      0.093     1.0080
    43   opls_842      1    HepEG  H17      2      0.093     1.0080
    44   opls_843      1    HepEG  H18      2     0.0915     1.0080
    45   opls_844      1    HepEG  H19      2     0.0915     1.0080
    46   opls_845      1    HepEG  H1A      2     0.0916     1.0080
    47   opls_846      1    HepEG  H1B      2     0.0916     1.0080
    48   opls_847      1    HepEG  H1C      2     0.0943     1.0080
    49   opls_848      1    HepEG  H1D      2     0.0943     1.0080
    50   opls_849      1    HepEG  H1E      2     0.1067     1.0080
    51   opls_850      1    HepEG  H1F      2     0.1067     1.0080
    52   opls_851      1    HepEG  H1G      2     0.4236     1.0080
[ bonds ]
    2     1     1      0.1410 267776.000
    3     2     1      0.1529 224262.400
    4     3     1      0.1410 267776.000
    5     4     1      0.1410 267776.000
    6     5     1      0.1529 224262.400
    7     6     1      0.1410 267776.000
    8     7     1      0.1410 267776.000
    9     8     1      0.1529 224262.400
   10     9     1      0.1410 267776.000
   11    10     1      0.1410 267776.000
   12    11     1      0.1529 224262.400
   13    12     1      0.1410 267776.000
   14    13     1      0.1410 267776.000
   15    14     1      0.1529 224262.400
```

```
   16    15     1      0.1410 267776.000
   17    16     1      0.1410 267776.000
   18    17     1      0.1529 224262.400
   19    18     1      0.1410 267776.000
   20    19     1      0.1410 267776.000
   21    20     1      0.1529 224262.400
   22    21     1      0.1410 267776.000
   23     1     1      0.0945 462750.400
   24     2     1      0.1090 284512.000
   25     2     1      0.1090 284512.000
   26     3     1      0.1090 284512.000
   27     3     1      0.1090 284512.000
   28     5     1      0.1090 284512.000
   29     5     1      0.1090 284512.000
   30     6     1      0.1090 284512.000
   31     6     1      0.1090 284512.000
   32     8     1      0.1090 284512.000
   33     8     1      0.1090 284512.000
   34     9     1      0.1090 284512.000
   35     9     1      0.1090 284512.000
   36    11     1      0.1090 284512.000
   37    11     1      0.1090 284512.000
   38    12     1      0.1090 284512.000
   39    12     1      0.1090 284512.000
   40    14     1      0.1090 284512.000
   41    14     1      0.1090 284512.000
   42    15     1      0.1090 284512.000
   43    15     1      0.1090 284512.000
   44    17     1      0.1090 284512.000
   45    17     1      0.1090 284512.000
   46    18     1      0.1090 284512.000
   47    18     1      0.1090 284512.000
   48    20     1      0.1090 284512.000
   49    20     1      0.1090 284512.000
   50    21     1      0.1090 284512.000
   51    21     1      0.1090 284512.000
   52    22     1      0.0945 462750.400

[ angles ]
;  ai    aj    ak funct            c0            c1            c2            c3
    1     2     3     1    109.500    418.400
    2     3     4     1    109.500    418.400
    3     4     5     1    109.500    502.080
    4     5     6     1    109.500    418.400
    5     6     7     1    109.500    418.400
    6     7     8     1    109.500    502.080
    7     8     9     1    109.500    418.400
    8     9    10     1    109.500    418.400
    9    10    11     1    109.500    502.080
   10    11    12     1    109.500    418.400
   11    12    13     1    109.500    418.400
   12    13    14     1    109.500    502.080
   13    14    15     1    109.500    418.400
   14    15    16     1    109.500    418.400
   15    16    17     1    109.500    502.080
   16    17    18     1    109.500    418.400
   17    18    19     1    109.500    418.400
```

18	19	20	1	109.500	502.080
19	20	21	1	109.500	418.400
20	21	22	1	109.500	418.400
2	1	23	1	108.500	460.240
1	2	24	1	109.500	292.880
1	2	25	1	109.500	292.880
2	3	26	1	110.700	313.800
2	3	27	1	110.700	313.800
4	5	28	1	109.500	292.880
4	5	29	1	109.500	292.880
5	6	30	1	110.700	313.800
5	6	31	1	110.700	313.800
7	8	32	1	109.500	292.880
7	8	33	1	109.500	292.880
8	9	34	1	110.700	313.800
8	9	35	1	110.700	313.800
10	11	36	1	109.500	292.880
10	11	37	1	109.500	292.880
11	12	38	1	110.700	313.800
11	12	39	1	110.700	313.800
13	14	40	1	109.500	292.880
13	14	41	1	109.500	292.880
14	15	42	1	110.700	313.800
14	15	43	1	110.700	313.800
16	17	44	1	109.500	292.880
16	17	45	1	109.500	292.880
17	18	46	1	110.700	313.800
17	18	47	1	110.700	313.800
19	20	48	1	109.500	292.880
19	20	49	1	109.500	292.880
20	21	50	1	110.700	313.800
20	21	51	1	110.700	313.800
21	22	52	1	108.500	460.240
12	11	37	1	110.700	313.800
19	18	47	1	109.500	292.880
13	12	38	1	109.500	292.880
15	14	40	1	110.700	313.800
44	17	45	1	107.800	276.144
16	15	42	1	109.500	292.880
7	6	31	1	109.500	292.880
40	14	41	1	107.800	276.144
48	20	49	1	107.800	276.144
3	2	25	1	110.700	313.800
28	5	29	1	107.800	276.144
16	15	43	1	109.500	292.880
15	14	41	1	110.700	313.800
36	11	37	1	107.800	276.144
21	20	49	1	110.700	313.800
6	5	28	1	110.700	313.800
21	20	48	1	110.700	313.800
32	8	33	1	107.800	276.144
13	12	39	1	109.500	292.880
12	11	36	1	110.700	313.800
42	15	43	1	107.800	276.144
4	3	27	1	109.500	292.880
24	2	25	1	107.800	276.144
10	9	35	1	109.500	292.880

```
   22    21    51     1    109.500    292.880
   22    21    50     1    109.500    292.880
   18    17    45     1    110.700    313.800
    4     3    26     1    109.500    292.880
    9     8    32     1    110.700    313.800
   46    18    47     1    107.800    276.144
   34     9    35     1    107.800    276.144
    9     8    33     1    110.700    313.800
    7     6    30     1    109.500    292.880
   30     6    31     1    107.800    276.144
   26     3    27     1    107.800    276.144
   18    17    44     1    110.700    313.800
   10     9    34     1    109.500    292.880
   38    12    39     1    107.800    276.144
   19    18    46     1    109.500    292.880
    3     2    24     1    110.700    313.800
    6     5    29     1    110.700    313.800
   50    21    51     1    107.800    276.144

[ dihedrals ]
; IMPROPER DIHEDRAL ANGLES
;  ai    aj    ak    al funct             c0             c1             c2             c3
c4             c5

[ dihedrals ]
; PROPER DIHEDRAL ANGLES
;  ai    aj    ak    al funct             c0             c1             c2             c3
c4             c5
   12   11   10    9         3       1.715   2.845   1.046  -5.607  -0.000   0.000
   18   17   16   15         3       1.715   2.845   1.046  -5.607  -0.000   0.000
   15   14   13   12         3       1.715   2.845   1.046  -5.607  -0.000   0.000
    9    8    7    6         3       1.715   2.845   1.046  -5.607  -0.000   0.000
   21   20   19   18         3       1.715   2.845   1.046  -5.607  -0.000   0.000
    6    5    4    3         3       1.715   2.845   1.046  -5.607  -0.000   0.000
   11   10    9    8         3       1.715   2.845   1.046  -5.607  -0.000   0.000
   20   19   18   17         3       1.715   2.845   1.046  -5.607  -0.000   0.000
   14   13   12   11         3       1.715   2.845   1.046  -5.607  -0.000   0.000
   17   16   15   14         3       1.715   2.845   1.046  -5.607  -0.000   0.000
    5    4    3    2         3       1.715   2.845   1.046  -5.607  -0.000   0.000
    8    7    6    5         3       1.715   2.845   1.046  -5.607  -0.000   0.000
   43   15   14   41         3       0.628   1.883   0.000  -2.510  -0.000   0.000
   35    9    8   32         3       0.628   1.883   0.000  -2.510  -0.000   0.000
   34    9    8   33         3       0.628   1.883   0.000  -2.510  -0.000   0.000
   27    3    2   24         3       0.628   1.883   0.000  -2.510  -0.000   0.000
   26    3    2   25         3       0.628   1.883   0.000  -2.510  -0.000   0.000
   34    9    8   32         3       0.628   1.883   0.000  -2.510  -0.000   0.000
   46   18   17   45         3       0.628   1.883   0.000  -2.510  -0.000   0.000
   39   12   11   37         3       0.628   1.883   0.000  -2.510  -0.000   0.000
   50   21   20   48         3       0.628   1.883   0.000  -2.510  -0.000   0.000
   30    6    5   29         3       0.628   1.883   0.000  -2.510  -0.000   0.000
   51   21   20   48         3       0.628   1.883   0.000  -2.510  -0.000   0.000
   38   12   11   36         3       0.628   1.883   0.000  -2.510  -0.000   0.000
   42   15   14   40         3       0.628   1.883   0.000  -2.510  -0.000   0.000
   47   18   17   44         3       0.628   1.883   0.000  -2.510  -0.000   0.000
   43   15   14   40         3       0.628   1.883   0.000  -2.510  -0.000   0.000
   39   12   11   36         3       0.628   1.883   0.000  -2.510  -0.000   0.000
   38   12   11   37         3       0.628   1.883   0.000  -2.510  -0.000   0.000
```

35	9	8	33	3	0.628	1.883	0.000	-2.510	-0.000	0.000
50	21	20	49	3	0.628	1.883	0.000	-2.510	-0.000	0.000
30	6	5	28	3	0.628	1.883	0.000	-2.510	-0.000	0.000
26	3	2	24	3	0.628	1.883	0.000	-2.510	-0.000	0.000
31	6	5	29	3	0.628	1.883	0.000	-2.510	-0.000	0.000
46	18	17	44	3	0.628	1.883	0.000	-2.510	-0.000	0.000
51	21	20	49	3	0.628	1.883	0.000	-2.510	-0.000	0.000
27	3	2	25	3	0.628	1.883	0.000	-2.510	-0.000	0.000
42	15	14	41	3	0.628	1.883	0.000	-2.510	-0.000	0.000
47	18	17	45	3	0.628	1.883	0.000	-2.510	-0.000	0.000
31	6	5	28	3	0.628	1.883	0.000	-2.510	-0.000	0.000
48	20	21	22	3	0.979	2.937	0.000	-3.916	-0.000	0.000
27	3	2	1	3	0.979	2.937	0.000	-3.916	-0.000	0.000
49	20	21	22	3	0.979	2.937	0.000	-3.916	-0.000	0.000
26	3	2	1	3	0.979	2.937	0.000	-3.916	-0.000	0.000
29	5	6	7	3	0.979	2.937	0.000	-3.916	-0.000	0.000
43	15	14	13	3	0.979	2.937	0.000	-3.916	-0.000	0.000
42	15	14	13	3	0.979	2.937	0.000	-3.916	-0.000	0.000
34	9	8	7	3	0.979	2.937	0.000	-3.916	-0.000	0.000
35	9	8	7	3	0.979	2.937	0.000	-3.916	-0.000	0.000
24	2	3	4	3	0.979	2.937	0.000	-3.916	-0.000	0.000
51	21	20	19	3	0.979	2.937	0.000	-3.916	-0.000	0.000
37	11	12	13	3	0.979	2.937	0.000	-3.916	-0.000	0.000
28	5	6	7	3	0.979	2.937	0.000	-3.916	-0.000	0.000
32	8	9	10	3	0.979	2.937	0.000	-3.916	-0.000	0.000
47	18	17	16	3	0.979	2.937	0.000	-3.916	-0.000	0.000
46	18	17	16	3	0.979	2.937	0.000	-3.916	-0.000	0.000
45	17	18	19	3	0.979	2.937	0.000	-3.916	-0.000	0.000
33	8	9	10	3	0.979	2.937	0.000	-3.916	-0.000	0.000
31	6	5	4	3	0.979	2.937	0.000	-3.916	-0.000	0.000
38	12	11	10	3	0.979	2.937	0.000	-3.916	-0.000	0.000
44	17	18	19	3	0.979	2.937	0.000	-3.916	-0.000	0.000
40	14	15	16	3	0.979	2.937	0.000	-3.916	-0.000	0.000
25	2	3	4	3	0.979	2.937	0.000	-3.916	-0.000	0.000
36	11	12	13	3	0.979	2.937	0.000	-3.916	-0.000	0.000
39	12	11	10	3	0.979	2.937	0.000	-3.916	-0.000	0.000
41	14	15	16	3	0.979	2.937	0.000	-3.916	-0.000	0.000
50	21	20	19	3	0.979	2.937	0.000	-3.916	-0.000	0.000
30	6	5	4	3	0.979	2.937	0.000	-3.916	-0.000	0.000
25	2	1	23	3	0.736	2.209	0.000	-2.946	-0.000	0.000
24	2	1	23	3	0.736	2.209	0.000	-2.946	-0.000	0.000
31	6	7	8	3	1.590	4.770	0.000	-6.360	-0.000	0.000
45	17	16	15	3	1.590	4.770	0.000	-6.360	-0.000	0.000
40	14	13	12	3	1.590	4.770	0.000	-6.360	-0.000	0.000
29	5	4	3	3	1.590	4.770	0.000	-6.360	-0.000	0.000
47	18	19	20	3	1.590	4.770	0.000	-6.360	-0.000	0.000
36	11	10	9	3	1.590	4.770	0.000	-6.360	-0.000	0.000
41	14	13	12	3	1.590	4.770	0.000	-6.360	-0.000	0.000
32	8	7	6	3	1.590	4.770	0.000	-6.360	-0.000	0.000
26	3	4	5	3	1.590	4.770	0.000	-6.360	-0.000	0.000
27	3	4	5	3	1.590	4.770	0.000	-6.360	-0.000	0.000
35	9	10	11	3	1.590	4.770	0.000	-6.360	-0.000	0.000
46	18	19	20	3	1.590	4.770	0.000	-6.360	-0.000	0.000
48	20	19	18	3	1.590	4.770	0.000	-6.360	-0.000	0.000
39	12	13	14	3	1.590	4.770	0.000	-6.360	-0.000	0.000
49	20	19	18	3	1.590	4.770	0.000	-6.360	-0.000	0.000
37	11	10	9	3	1.590	4.770	0.000	-6.360	-0.000	0.000

33	8	7	6	3	1.590	4.770	0.000	-6.360	-0.000	0.000
28	5	4	3	3	1.590	4.770	0.000	-6.360	-0.000	0.000
42	15	16	17	3	1.590	4.770	0.000	-6.360	-0.000	0.000
30	6	7	8	3	1.590	4.770	0.000	-6.360	-0.000	0.000
43	15	16	17	3	1.590	4.770	0.000	-6.360	-0.000	0.000
44	17	16	15	3	1.590	4.770	0.000	-6.360	-0.000	0.000
38	12	13	14	3	1.590	4.770	0.000	-6.360	-0.000	0.000
34	9	10	11	3	1.590	4.770	0.000	-6.360	-0.000	0.000
52	22	21	20	3	-0.444	3.833	0.728	-4.117	-0.000	0.000
23	1	2	3	3	-0.444	3.833	0.728	-4.117	-0.000	0.000
52	22	21	51	3	0.736	2.209	0.000	-2.946	-0.000	0.000
52	22	21	50	3	0.736	2.209	0.000	-2.946	-0.000	0.000
22	21	20	19	3	9.035	-9.035	0.000	-0.000	-0.000	0.000
4	3	2	1	3	9.035	-9.035	0.000	-0.000	-0.000	0.000
10	9	8	7	3	-1.151	1.151	0.000	-0.000	-0.000	0.000
13	12	11	10	3	-1.151	1.151	0.000	-0.000	-0.000	0.000
16	15	14	13	3	-1.151	1.151	0.000	-0.000	-0.000	0.000
7	6	5	4	3	-1.151	1.151	0.000	-0.000	-0.000	0.000
19	18	17	16	3	-1.151	1.151	0.000	-0.000	-0.000	0.000

[pairs]

1	4	1
2	5	1
3	6	1
4	7	1
5	8	1
6	9	1
7	10	1
8	11	1
9	12	1
10	13	1
11	14	1
3	23	1
12	15	1
1	26	1
4	24	1
1	27	1
13	16	1
4	25	1
14	17	1
5	26	1
3	28	1
5	27	1
3	29	1
15	18	1
4	30	1
16	19	1
7	28	1
4	31	1
7	29	1
17	20	1
8	30	1
6	32	1
18	21	1
8	31	1
6	33	1
19	22	1

7	34	1
10	32	1
7	35	1
10	33	1
11	34	1
9	36	1
11	35	1
9	37	1
23	24	1
23	25	1
10	38	1
13	36	1
10	39	1
24	26	1
13	37	1
25	26	1
24	27	1
25	27	1
14	38	1
12	40	1
14	39	1
12	41	1
13	42	1
16	40	1
13	43	1
16	41	1
28	30	1
29	30	1
28	31	1
17	42	1
15	44	1
29	31	1
17	43	1
15	45	1
16	46	1
19	44	1
16	47	1
19	45	1
32	34	1
20	46	1
18	48	1
33	34	1
32	35	1
20	47	1
18	49	1
33	35	1
19	50	1
22	48	1
19	51	1
22	49	1
20	52	1
36	38	1
37	38	1
36	39	1
37	39	1
40	42	1
41	42	1

```
    40    43    1
    41    43    1
    44    46    1
    45    46    1
    44    47    1
    45    47    1
    48    50    1
    49    50    1
    48    51    1
    49    51    1
    50    52    1
    51    52    1
```

2.1.2 Structure Coordinate files

Structure/coordinate file "single_DEG.gro"

```
Diethylene glycol
   17
    1DEG    O00    1   0.100   0.100   0.000
    1DEG    C01    2  -0.042   0.100   0.000
    1DEG    C02    3  -0.094   0.100   0.143
    1DEG    O03    4  -0.237   0.100   0.143
    1DEG    C04    5  -0.289   0.100   0.276
    1DEG    C05    6  -0.441   0.100   0.269
    1DEG    O06    7  -0.487  -0.017   0.202
    1DEG    H07    8   0.128   0.100  -0.093
    1DEG    H08    9  -0.078   0.012  -0.054
    1DEG    H09   10  -0.078   0.189  -0.054
    1DEG    H0A   11  -0.057   0.189   0.196
    1DEG    H0B   12  -0.057   0.011   0.196
    1DEG    H0C   13  -0.254   0.189   0.330
    1DEG    H0D   14  -0.254   0.010   0.327
    1DEG    H0E   15  -0.478   0.187   0.213
    1DEG    H0F   16  -0.485   0.102   0.369
    1DEG    H0G   17  -0.434  -0.021   0.120
   1.00000   1.00000   1.00000
```

Structure/coordinate file "single_TEG.gro"

```
Triethylene glycol
   24
    1TEG    O00    1   0.100   0.100   0.000
    1TEG    C01    2  -0.042   0.100   0.000
    1TEG    C02    3  -0.095   0.100   0.143
    1TEG    O03    4  -0.237   0.100   0.140
    1TEG    C04    5  -0.292   0.088   0.272
    1TEG    C05    6  -0.445   0.082   0.263
    1TEG    O06    7  -0.485  -0.041   0.202
    1TEG    C07    8  -0.628  -0.050   0.192
    1TEG    C08    9  -0.662  -0.177   0.114
    1TEG    O09   10  -0.608  -0.291   0.181
    1TEG    H0A   11   0.128   0.100  -0.093
```

```
    1TEG    H0B   12  -0.078   0.011  -0.054
    1TEG    H0C   13  -0.078   0.188  -0.054
    1TEG    H0D   14  -0.058   0.189   0.196
    1TEG    H0E   15  -0.058   0.011   0.196
    1TEG    H0F   16  -0.262   0.175   0.331
    1TEG    H0G   17  -0.254  -0.003   0.319
    1TEG    H0H   18  -0.482   0.166   0.203
    1TEG    H0I   19  -0.488   0.087   0.364
    1TEG    H0J   20  -0.667   0.038   0.139
    1TEG    H0K   21  -0.669  -0.055   0.293
    1TEG    H0M   22  -0.618  -0.174   0.014
    1TEG    H0N   23  -0.770  -0.190   0.106
    1TEG    H0O   24  -0.514  -0.268   0.198
   1.00000   1.00000   1.00000
```

Structure/coordinate file "single_TeEG.gro"

```
Tetraethylene glycol
   31
    1TeEG   O00    1   0.100   0.100   0.000
    1TeEG   C01    2  -0.042   0.100   0.000
    1TeEG   C02    3  -0.095   0.100   0.143
    1TeEG   O03    4  -0.238   0.100   0.141
    1TeEG   C04    5  -0.291   0.100   0.273
    1TeEG   C05    6  -0.444   0.100   0.266
    1TeEG   O06    7  -0.497   0.100   0.399
    1TeEG   C07    8  -0.640   0.100   0.398
    1TeEG   C08    9  -0.691   0.100   0.541
    1TeEG   O09   10  -0.833   0.100   0.542
    1TeEG   C0A   11  -0.884   0.100   0.677
    1TeEG   C0B   12  -1.036   0.100   0.671
    1TeEG   O0C   13  -1.082  -0.017   0.604
    1TeEG   H0D   14   0.128   0.100  -0.093
    1TeEG   H0E   15  -0.078   0.012  -0.054
    1TeEG   H0F   16  -0.078   0.189  -0.054
    1TeEG   H0G   17  -0.058   0.189   0.196
    1TeEG   H0H   18  -0.058   0.011   0.196
    1TeEG   H0I   19  -0.257   0.189   0.327
    1TeEG   H0J   20  -0.256   0.012   0.328
    1TeEG   H0K   21  -0.478   0.011   0.212
    1TeEG   H0M   22  -0.478   0.188   0.211
    1TeEG   H0N   23  -0.677   0.011   0.345
    1TeEG   H0O   24  -0.677   0.188   0.345
    1TeEG   H0P   25  -0.653   0.189   0.594
    1TeEG   H0Q   26  -0.653   0.012   0.594
    1TeEG   H0R   27  -0.848   0.189   0.730
    1TeEG   H0S   28  -0.848   0.010   0.728
    1TeEG   H0T   29  -1.073   0.187   0.616
    1TeEG   H0U   30  -1.079   0.101   0.772
    1TeEG   H0V   31  -1.031  -0.020   0.521
   1.00000   1.00000   1.00000
```

Structure/coordinate file "single_PeEG.gro"

```
Pentaethylene glycol
   38
```

```
    1PeEG   O00    1   0.100   0.100   0.000
    1PeEG   C01    2  -0.042   0.100   0.000
    1PeEG   C02    3  -0.095   0.100   0.143
    1PeEG   O03    4  -0.237   0.100   0.141
    1PeEG   C04    5  -0.291   0.100   0.273
    1PeEG   C05    6  -0.443   0.099   0.266
    1PeEG   O06    7  -0.496   0.093   0.399
    1PeEG   C07    8  -0.638   0.103   0.400
    1PeEG   C08    9  -0.688   0.102   0.545
    1PeEG   O09   10  -0.647   0.223   0.609
    1PeEG   C0A   11  -0.685   0.225   0.747
    1PeEG   C0B   12  -0.630   0.352   0.812
    1PeEG   O0C   13  -0.672   0.358   0.948
    1PeEG   C0D   14  -0.621   0.476   1.012
    1PeEG   C0E   15  -0.670   0.478   1.156
    1PeEG   O0F   16  -0.622   0.363   1.225
    1PeEG   H0G   17   0.128   0.100  -0.093
    1PeEG   H0H   18  -0.078   0.012  -0.054
    1PeEG   H0I   19  -0.078   0.189  -0.054
    1PeEG   H0J   20  -0.058   0.189   0.196
    1PeEG   H0K   21  -0.058   0.011   0.196
    1PeEG   H0M   22  -0.257   0.189   0.328
    1PeEG   H0N   23  -0.256   0.011   0.328
    1PeEG   H0O   24  -0.478   0.013   0.209
    1PeEG   H0P   25  -0.478   0.190   0.216
    1PeEG   H0Q   26  -0.680   0.017   0.346
    1PeEG   H0R   27  -0.669   0.196   0.351
    1PeEG   H0S   28  -0.645   0.017   0.598
    1PeEG   H0T   29  -0.797   0.096   0.546
    1PeEG   H0U   30  -0.647   0.137   0.799
    1PeEG   H0V   31  -0.795   0.224   0.755
    1PeEG   H0W   32  -0.665   0.440   0.757
    1PeEG   H0X   33  -0.520   0.350   0.807
    1PeEG   H0Y   34  -0.657   0.565   0.959
    1PeEG   H0Z   35  -0.512   0.473   1.009
    1PeEG   H10   36  -0.779   0.476   1.160
    1PeEG   H11   37  -0.633   0.567   1.208
    1PeEG   H12   38  -0.644   0.287   1.167
   1.0    00000   1.00000
```

Hexaethylene glycol

```
   45
    1HexEG  O00    1   0.100   0.100   0.000
    1HexEG  C01    2  -0.042   0.100   0.000
    1HexEG  C02    3  -0.094   0.100   0.143
    1HexEG  O03    4  -0.237   0.100   0.141
    1HexEG  C04    5  -0.290   0.100   0.274
    1HexEG  C05    6  -0.443   0.100   0.267
    1HexEG  O06    7  -0.495   0.101   0.400
    1HexEG  C07    8  -0.637   0.100   0.400
    1HexEG  C08    9  -0.687   0.101   0.544
    1HexEG  O09   10  -0.830   0.101   0.544
    1HexEG  C0A   11  -0.882   0.101   0.676
    1HexEG  C0B   12  -1.034   0.101   0.670
    1HexEG  O0C   13  -1.087   0.101   0.803
    1HexEG  C0D   14  -1.230   0.101   0.803
```

```
    1HexEG  C0E   15  -1.280   0.101   0.947
    1HexEG  O0F   16  -1.423   0.101   0.948
    1HexEG  C0G   17  -1.473   0.101   1.082
    1HexEG  C0H   18  -1.625   0.101   1.077
    1HexEG  O0I   19  -1.671  -0.016   1.009
    1HexEG  H0J   20   0.128   0.100  -0.093
    1HexEG  H0K   21  -0.078   0.012  -0.054
    1HexEG  H0M   22  -0.078   0.189  -0.054
    1HexEG  H0N   23  -0.057   0.189   0.196
    1HexEG  H0O   24  -0.058   0.011   0.196
    1HexEG  H0P   25  -0.256   0.189   0.328
    1HexEG  H0Q   26  -0.256   0.012   0.328
    1HexEG  H0R   27  -0.477   0.012   0.213
    1HexEG  H0S   28  -0.477   0.189   0.213
    1HexEG  H0T   29  -0.675   0.012   0.348
    1HexEG  H0U   30  -0.675   0.189   0.347
    1HexEG  H0V   31  -0.650   0.190   0.596
    1HexEG  H0W   32  -0.650   0.013   0.597
    1HexEG  H0X   33  -0.847   0.190   0.730
    1HexEG  H0Y   34  -0.847   0.013   0.731
    1HexEG  H0Z   35  -1.069   0.012   0.617
    1HexEG  H10   36  -1.070   0.189   0.616
    1HexEG  H11   37  -1.267   0.012   0.750
    1HexEG  H12   38  -1.267   0.189   0.750
    1HexEG  H13   39  -1.242   0.190   0.998
    1HexEG  H14   40  -1.242   0.013   0.999
    1HexEG  H15   41  -1.437   0.190   1.135
    1HexEG  H16   42  -1.437   0.011   1.133
    1HexEG  H17   43  -1.662   0.188   1.021
    1HexEG  H18   44  -1.668   0.102   1.177
    1HexEG  H19   45  -1.620  -0.019   0.926
   1.00000   1.00000   1.00000
```

Heptaethylene glycol

```
   52
    1HepEG  O00    1   0.100   0.100   0.000
    1HepEG  C01    2  -0.042   0.100   0.000
    1HepEG  C02    3  -0.094   0.100   0.143
    1HepEG  O03    4  -0.237   0.100   0.141
    1HepEG  C04    5  -0.290   0.100   0.274
    1HepEG  C05    6  -0.442   0.100   0.267
    1HepEG  O06    7  -0.494   0.100   0.400
    1HepEG  C07    8  -0.637   0.100   0.400
    1HepEG  C08    9  -0.687   0.100   0.544
    1HepEG  O09   10  -0.829   0.106   0.544
    1HepEG  C0A   11  -0.882   0.096   0.677
    1HepEG  C0B   12  -1.034   0.095   0.669
    1HepEG  O0C   13  -1.077  -0.028   0.608
    1HepEG  C0D   14  -1.218  -0.032   0.591
    1HepEG  C0E   15  -1.256  -0.159   0.516
    1HepEG  O0F   16  -1.398  -0.166   0.503
    1HepEG  C0G   17  -1.438  -0.284   0.433
    1HepEG  C0H   18  -1.590  -0.285   0.424
    1HepEG  O0I   19  -1.633  -0.403   0.354
    1HepEG  C0J   20  -1.776  -0.407   0.344
    1HepEG  C0K   21  -1.815  -0.533   0.269
    1HepEG  O0M   22  -1.769  -0.648   0.340
```

```
    1HepEG  H0N   23   0.128   0.100  -0.093
    1HepEG  H0O   24  -0.078   0.012  -0.054
    1HepEG  H0P   25  -0.078   0.189  -0.054
    1HepEG  H0Q   26  -0.057   0.189   0.196
    1HepEG  H0R   27  -0.057   0.011   0.196
    1HepEG  H0S   28  -0.255   0.188   0.328
    1HepEG  H0T   29  -0.255   0.011   0.328
    1HepEG  H0U   30  -0.477   0.011   0.213
    1HepEG  H0V   31  -0.477   0.188   0.213
    1HepEG  H0W   32  -0.675   0.011   0.348
    1HepEG  H0X   33  -0.675   0.188   0.347
    1HepEG  H0Y   34  -0.646   0.186   0.598
    1HepEG  H0Z   35  -0.652   0.009   0.595
    1HepEG  H10   36  -0.849   0.183   0.735
    1HepEG  H11   37  -0.846   0.004   0.723
    1HepEG  H12   38  -1.070   0.179   0.610
    1HepEG  H13   39  -1.077   0.100   0.770
    1HepEG  H14   40  -1.252   0.056   0.534
    1HepEG  H15   41  -1.268  -0.030   0.689
    1HepEG  H16   42  -1.218  -0.246   0.569
    1HepEG  H17   43  -1.209  -0.158   0.416
    1HepEG  H18   44  -1.403  -0.373   0.487
    1HepEG  H19   45  -1.394  -0.285   0.333
    1HepEG  H1A   46  -1.625  -0.196   0.371
    1HepEG  H1B   47  -1.633  -0.284   0.525
    1HepEG  H1C   48  -1.812  -0.318   0.290
    1HepEG  H1D   49  -1.818  -0.409   0.445
    1HepEG  H1E   50  -1.769  -0.535   0.169
    1HepEG  H1F   51  -1.924  -0.540   0.259
    1HepEG  H1G   52  -1.675  -0.631   0.359
   1.00000   1.00000   1.00000
```

2.1.3 Molecular Dynamics Parameter files

Molecular dynamics parameter file for equilibrating positions: "em.mdp"

```
; very basics of the simulation
define                   = -DFLEXIBLE      ; flexible water so simulation does not
crash
integrator               = steep           ; steepest decent
nsteps                   = 100000          ; number of steepest decent steps
emstep                   = 0.01            ; 0.01 nm step
; next neighbor search and periodic boundary conditions
nstlist                  = 1               ; freq. to update neighbor list & long
range forces (>= 20 w/ GPUs)
rlist                    = 1.4             ; short-range neighbor list cutoff (nm)
cutoff-scheme            = Verlet          ; atom-based neighbor search with an
implicit buffer region
pbc                      = xyz             ; periodicity in x, y and z
; coulomb interaction
coulombtype              = PME             ; particle-mesh ewald summation for long
range (>rcoulomb)
rcoulomb                 = 1.4             ; short-range electrostatic cutoff (in nm)
(with PME,rcoulomb >= rvdw)
```

```
coulomb-modifier         = Potential-shift-Verlet ; shifts potential by constant so
potential is 0 at cut-off
fourierspacing           = 0.168          ; spacing of FFT in reciprocal space in
PME long range treatment (in nm)
pme-order                = 4              ; cubic PME interpolation order
; lennard-jones potential handling
vdwtype                  = cutoff         ; simple truncation cutoff
vdw-modifier             = Potential-shift-Verlet ; shifts potential by constant so
potential is 0 at cut-off
rvdw                     = 1.4            ; short-range vdw cutoff (in nm)
```

Molecular dynamics parameter file equilibrating under constant NPT conditions "eq_NPT_328K.mdp"

```
; very basics of the simulation
integrator               = md             ; solve newtown's equation of motion
dt                       = 0.002          ; integration time step / ps
nsteps                   = varies         ; number of steps (500000 * 0.002 = 1000
ps)
; remove drifts of the center of mass
comm-mode                = linear    ; remove COM translation
nstcomm                  = 10             ; number of steps for COM removal
comm-grps                = System         ; COM translation removed from system
; control frequency of output
nstvout                 = 0              ; write velocities to trajectory file every
number of steps
nstfout                 = 0              ; write forces to trajectory file every
number of steps
nstlog                   = varies            ; update log file every number of steps
nstcalcenergy            = 10             ; calculate energies/pressures every
nstenergy steps
nstenergy                = varies             ; write energies to energy file every
number of steps
nstxout-compressed       = varies            ; write positions using compression
(saves memory, worse quality)
compressed-x-precision   = 1000           ; precision to write compressed trajectory
; next neighbor search and periodic boundary conditions
nstlist                  = 20             ; freq. to update neighbor list & long
range forces (>= 20 w/ GPUs)
rlist                    = 1.4            ; short-range neighbor list cutoff (nm)
cutoff-scheme            = Verlet         ; atom-based neighbor search with an
implicit buffer region
pbc                      = xyz            ; periodicity in x, y and z
; coulomb interaction
coulombtype              = PME            ; particle-mesh ewald summation for long
range (>rcoulomb)
rcoulomb                 = 1.4            ; short-range electrostatic cutoff (in nm)
(with PME,rcoulomb >= rvdw)
coulomb-modifier         = Potential-shift-Verlet ; shifts potential by constant so
potential is 0 at cut-off
fourierspacing           = 0.168          ; spacing of FFT in reciprocal space in
PME long range treatment (in nm)
pme-order                = 4              ; cubic PME interpolation order
; lennard-jones potential handling
vdwtype                  = cutoff         ; simple truncation cutoff
vdw-modifier             = Potential-shift-Verlet ; shifts potential by constant so
potential is 0 at cut-off
rvdw                     = 1.4            ; short-range vdw cutoff (in nm)
```

```
DispCorr                  = EnerPres        ; long range correction for energy and
pressure from using vdw cutoff
; temperature coupling
tcoupl                    = v-rescale       ; the algorithm to use, v-rescale
generates correct canonical ensemble
tc-grps                   = System          ; groups to couple to temperature bath
tau-t                     = 1.0             ; time constant (in ps), meaning varies by
algorithm
ref-t                     = 328             ; temperature for coupling (K)
nsttcouple                = 1               ; frequency to couple temperature
; velocity generation
gen-vel                   = yes             ; generate velocities according to Maxwell
distr. (no for initial vel 0)
gen-temp                  = 328             ; temperature for Maxwell distribution (K)
gen-seed                  = -1              ; generate a random seed
; pressure coupling
pcoupl                    = Parrinello-Rahman  ; the algorithm to use (no for NVT)
pcoupltype                = isotropic       ; all ordinates scaled equally (preserves
the cubic box)
compressibility           = 4.5e-5          ; experimental value for water at 298 K
(in 1/bar)
tau-p                     = 5.0             ; time constant (in ps), should be about
4-5 times larger than tau_t
ref-p                     = 1               ; reference pressure (in bar)
nstpcouple                = 1               ; frequency to couple pressure
; constraints
constraints               = h-bonds         ; constrains bonds only involving hydrogen
constraint_algorithm      = lincs           ; algorithm to use, lincs should NOT be
used for angle constraining
lincs-order               = 4               ; highest order in constraint coupling
matrix expansion
lincs-iter                = 1               ; accuracy of lincs algorithm
continuation              = no              ; no for applying constraints at start of
run
```

Molecular dynamics parameter file for production run und constant NVT condidtions “md_NVT_328K.mdp”

```
; very basics of the simulation
integrator                = md              ; solve newtown's equation of motion
dt                        = 0.002           ; integration time step / ps
nsteps                    = varies         ; number of steps (5000000 * 0.002 = 10 ns)
; remove drifts of the center of mass
comm-mode                 = linear    ; remove COM translation
nstcomm                   = 10              ; number of steps for COM removal
comm-grps                 = System          ; COM translation removed from system
; control frequency of output
nstvout                 = 0                ; write velocities to trajectory file every
number of steps
nstfout                 = 0                ; write forces to trajectory file every
number of steps
nstlog                    = varies             ; update log file every number of steps
nstcalcenergy             = 10              ; calculate energies/pressures every
nstenergy steps
nstenergy                 = 10              ; write energies to energy file every
number of steps
```

```
nstxout-compressed       = varies            ; write positions using compression
(saves memory, worse quality)
compressed-x-precision   = 1000            ; precision to write compressed trajectory
; next neighbor search and periodic boundary conditions
nstlist                  = 20              ; freq. to update neighbor list & long
range forces (>= 20 w/ GPUs)
rlist                    = 1.4             ; short-range neighbor list cutoff (nm)
cutoff-scheme            = Verlet          ; atom-based neighbor search with an
implicit buffer region
pbc                      = xyz             ; periodicity in x, y and z
; coulomb interaction
coulombtype              = PME             ; particle-mesh ewald summation for long
range (>rcoulomb)
rcoulomb                 = 1.4             ; short-range electrostatic cutoff (in nm)
(with PME,rcoulomb >= rvdw)
coulomb-modifier         = Potential-shift-Verlet ; shifts potential by constant so
potential is 0 at cut-off
fourierspacing           = 0.168           ; spacing of FFT in reciprocal space in
PME long range treatment (in nm)
pme-order                = 4               ; cubic PME interpolation order
; lennard-jones potential handling
vdwtype                  = cutoff          ; simple truncation cutoff
vdw-modifier             = Potential-shift-Verlet ; shifts potential by constant so
potential is 0 at cut-off
rvdw                     = 1.4             ; short-range vdw cutoff (in nm)
DispCorr                 = EnerPres        ; long range correction for energy and
pressure from using vdw cutoff
; temperature coupling
tcoupl                   = v-rescale       ; the algorithm to use, v-rescale
generates correct canonical ensemble
tc-grps                  = System          ; groups to couple to temperature bath
tau-t                    = 1.0             ; time constant (in ps), meaning varies by
algorithm
ref-t                    = 328             ; temperature for coupling (K)
nsttcouple               = 1               ; frequency to couple temperature
; velocity generation
gen-vel                  = yes             ; generate velocities according to Maxwell
distr. (no for initial vel 0)
gen-temp                 = 328             ; temperature for Maxwell distribution (K)
gen-seed                 = -1              ; generate a random seed
; pressure coupling
pcoupl                   = no              ; the algorithm to use (no for NVT)
; constraints
constraints              = h-bonds         ; constrains bonds only involving hydrogen
constraint_algorithm     = lincs           ; algorithm to use, lincs should NOT be
used for angle constraining
lincs-order              = 4               ; highest order in constraint coupling
matrix expansion
lincs-iter               = 1               ; accuracy of lincs algorithm
continuation             = no              ; no for applying constraints at start of
run
```

2.2 GROMOS forcefield

2.2.1 Topology files

Example topology file for tetraethylene glycol

```
; parameter level topology
#include "/home/mmh/BrockportMDFiles/2016H66_orga_GROMACS/forcefield.itp"

; TeEG molecular level topology
#include "TeEG.itp"

; System level topology
[ system ]
Tetraethylene glycol

[ molecules ]
TeEG   1000
```

"DEG.itp"

```
; Include topology for diethylene glycol
; Contains only molecular level lists
; Exclude nonbonded interactions within 3 bonds, but generate nonbonded
; interactions for 1-4 pairs via a pairs list
; Charge groups are irrelevant because of use of the Verlet cutoff scheme

[ moleculetype ]
; name   nrexcl
DEG      3


[ atoms ]
; nr  type  resnr residu  atom  cgnr  charge   mass
1     H      1     TEG    H1     1    0.4100   1.00800
2     OA     1     TEG    OA1    1   -0.7000   15.99940
3     CH2    1     TEG    CH21   1    0.2900   14.02700
4     CH2    1     TEG    CH22   1    0.2900   14.02700
5     OE     1     TEG    OE1    1   -0.5800   15.99940
6     CH2    1     TEG    CH23   1    0.2900   14.02700
7     CH2    1     TEG    CH24   1    0.2900   14.02700
8     OA     1     TEG    OA2    1   -0.7000   15.99940
9     H      1     TEG    H2     1    0.4100   1.00800


[ pairs ]
; ai al func
  1  4   1
  2  5   1
  3  6   1
  4  7   1
  5  8   1
  6  9   1
```

```
[ bonds ]
; i  j  func  c0  c1
  1  2   2    gb_1
  2  3   2    gb_18
  3  4   2    gb_27
  4  5   2    gb_18
  5  6   2    gb_18
  6  7   2    gb_27
  7  8   2    gb_18
  8  9   2    gb_1


[ angles ]
; i  j  k  func  angle  fc
  1  2  3   2    ga_12
  2  3  4   2    ga_15
  3  4  5   2    ga_15
  4  5  6   2    ga_15
  5  6  7   2    ga_15
  6  7  8   2    ga_15
  7  8  9   2    ga_12


[ dihedrals ]
; ai aj ak al func ph0  cp  mult
  1  2  3  4   1   gd_23
  2  3  4  5   9   gd_45
  2  3  4  5   9   gd_46
  2  3  4  5   9   gd_47
  3  4  5  6   9   gd_42
  3  4  5  6   9   gd_43
  3  4  5  6   9   gd_44
  4  5  6  7   9   gd_42
  4  5  6  7   9   gd_43
  4  5  6  7   9   gd_44
  5  6  7  8   9   gd_45
  5  6  7  8   9   gd_46
  5  6  7  8   9   gd_47
  6  7  8  9   9   gd_23
```

"TeEG.itp"

```
Include topology for triethylene glycol
; Contains only molecular level lists
; Exclude nonbonded interactions within 3 bonds, but generate nonbonded
; interactions for 1-4 pairs via a pairs list
; Charge groups are irrelevant because of use of the Verlet cutoff scheme
[ moleculetype ]
; name    nrexcl
TeEG      3

[ atoms ]
; nr  type  resnr residu  atom  cgnr  charge   mass
1     H       1   TeEG    H1     1    0.4100   1.00800
2     OA      1   TeEG    OA1    1   -0.7000   15.99940
3     CH2     1   TeEG    CH21   1    0.2900   14.02700
4     CH2     1   TeEG    CH22   1    0.2900   14.02700
5     OE      1   TeEG    OE1    1   -0.5800   15.99940
```

```
6     CH2     1   TeEG    CH23   1    0.2900   14.02700
7     CH2     1   TeEG    CH24   1    0.2900   14.02700
8     OE      1   TeEG    OE2    1   -0.5800   15.99940
9     CH2     1   TeEG    CH25   1    0.2900   14.02700
10    CH2     1   TeEG    CH26   1    0.2900   14.02700
11    OE      1   TeEG    OE3    1   -0.5800   15.99940
12    CH2     1   TeEG    CH27   1    0.2900   14.02700
13    CH2     1   TeEG    CH28   1    0.2900   14.02700
14    OA      1   TeEG    OA2    1   -0.7000   15.99940
15    H       1   TeEG    H2     1    0.4100   1.00800


[ pairs ]
; ai al  func
  1  4   1
  2  5   1
  3  6   1
  4  7   1
  5  8   1
  6  9   1
  7  10  1
  8  11  1
  9  12  1
  10 13  1
  11 14  1
  12 15  1

[ bonds ]
; OA - H gb_1
; OA - CH2 gb_18
; CH2 - CH2 gb_27
; CH2 - OE gb_18
; ai aj  func
  1  2    2   gb_1
  2  3    2   gb_18
  3  4    2   gb_27
  4  5    2   gb_18
  5  6    2   gb_18
  6  7    2   gb_27
  7  8    2   gb_18
  8  9    2   gb_18
  9  10   2   gb_27
  10 11   2   gb_18
  11 12   2   gb_18
  12 13   2   gb_27
  13 14   2   gb_18
  14 15   2   gb_1

[ angles ]
; H - OA - CH2  ga_12
; OA - CH2 - CH2 ga_15
; CH2 - CH2 - OE ga_15
; CH2 - OE - CH2 ga_15
; ai aj ak  func
  1  2  3    2   ga_12
  2  3  4    2   ga_15
  3  4  5    2   ga_15
```

```
  4  5  6    2   ga_15
  5  6  7    2   ga_15
  6  7  8    2   ga_15
  7  8  9    2   ga_15
  8  9  10   2   ga_15
  9  10 11   2   ga_15
  10 11 12   2   ga_15
  11 12 13   2   ga_15
  12 13 14   2   ga_15
  13 14 15   2   ga_12

[ dihedrals ]
; H - OA - CH2 - CH2   gd_23
; OA - CH2 - CH2 - OE   gd_45, gd_46, gd_47
; CH2 - CH2 - OE - CH2  gd_42, gd_43, gd_44
; OE - CH2 - CH2 - OE  gd_45, gd_46, gd_47
; ai aj ak al  func
  1  2  3  4   1   gd_23
  2  3  4  5   9   gd_45
  2  3  4  5   9   gd_46
  2  3  4  5   9   gd_47
  3  4  5  6   9   gd_42
  3  4  5  6   9   gd_43
  3  4  5  6   9   gd_44
  4  5  6  7   9   gd_42
  4  5  6  7   9   gd_43
  4  5  6  7   9   gd_44
  5  6  7  8   9   gd_45
  5  6  7  8   9   gd_46
  5  6  7  8   9   gd_47
  6  7  8  9   9   gd_42
  6  7  8  9   9   gd_43
  6  7  8  9   9   gd_44
  7  8  9  10  9   gd_42
  7  8  9  10  9   gd_43
  7  8  9  10  9   gd_44
  8  9  10 11  9   gd_45
  8  9  10 11  9   gd_46
  8  9  10 11  9   gd_47
  9  10 11 12  9   gd_42
  9  10 11 12  9   gd_43
  9  10 11 12  9   gd_44
  10 11 12 13  9   gd_42
  10 11 12 13  9   gd_43
  10 11 12 13  9   gd_44
  11 12 13 14  9   gd_45
  11 12 13 14  9   gd_46
  11 12 13 14  9   gd_47
  12 13 14 15  1   gd_23

run
```

2.2.2 Structure Coordinate file

```
Diethylene glycol
   9
    1DEG     H1    1   0.043   0.420  -0.038
    1DEG    OA1    2  -0.010   0.351  -0.000
    1DEG   CH21    3   0.050   0.230  -0.039
    1DEG   CH22    4  -0.037   0.117   0.019
    1DEG    OE1    5   0.025  -0.004  -0.019
    1DEG   CH23    6  -0.045  -0.119   0.027
    1DEG   CH24    7   0.032  -0.241  -0.027
    1DEG    OA2    8  -0.027  -0.361   0.014
    1DEG     H2    9  -0.021  -0.366   0.108

Tetraethylene glycol
   15
    1TeEG    H1    1   0.140   0.774   0.012
    1TeEG   OA1    2   0.070   0.762  -0.055
    1TeEG  CH21    3   0.060   0.623  -0.080
    1TeEG  CH22    4  -0.045   0.596  -0.188
    1TeEG   OE1    5  -0.054   0.458  -0.222
    1TeEG  CH23    6  -0.134   0.384  -0.130
    1TeEG  CH24    7  -0.145   0.239  -0.178
    1TeEG   OE2    8  -0.222   0.164  -0.084
    1TeEG  CH25    9  -0.238   0.028  -0.126
    1TeEG  CH26   10  -0.323  -0.047  -0.024
    1TeEG   OE3   11  -0.459  -0.002  -0.021
    1TeEG  CH27   12  -0.540  -0.057  -0.124
    1TeEG  CH28   13  -0.680   0.002  -0.115
    1TeEG   OA2   14  -0.766  -0.058  -0.211
    1TeEG    H2   15  -0.785  -0.148  -0.181
   0.00000   0.00000   0.00000
```

2.2.3 MD Parameter files, typical example for tetraethylene glycol

Molecular dynamics parameter file for molecular structure/position equilibrating “em.mdp”

```
; very basics of the simulation
; define                   = -DFLEXIBLE      ; flexible water so simulation does not 
crash
integrator               = steep           ; steepest decent
nsteps                   = 100000          ; number of steepest decent steps
emstep                   = 0.01            ; 0.01 nm step

; next neighbor search and periodic boundary conditions
nstlist                  = 1               ; freq. to update neighbor list & long 
range forces (>= 20 w/ GPUs)
rlist                    = 1.4             ; short-range neighbor list cutoff (nm)
cutoff-scheme            = Verlet          ; atom-based neighbor search with an 
implicit buffer region
pbc                      = xyz             ; periodicity in x, y and z

; coulomb interaction
coulombtype              = PME             ; particle-mesh ewald summation for long 
range (>rcoulomb)
```

```
rcoulomb                 = 1.4              ; short-range electrostatic cutoff (in nm)
(with PME,rcoulomb >= rvdw)
coulomb-modifier         = Potential-shift-Verlet ; shifts potential by constant so
potential is 0 at cut-off
fourierspacing           = 0.168            ; spacing of FFT in reciprocal space in
PME long range treatment (in nm)
pme-order                = 4                ; cubic PME interpolation order

; lennard-jones potential handling
vdwtype                  = cutoff           ; simple truncation cutoff
vdw-modifier             = Potential-shift-Verlet ; shifts potential by constant so
potential is 0 at cut-off
rvdw                     = 1.4              ; short-range vdw cutoff (in nm)
```

Molecular dynamics parameter file equilibrating under constant NPT conditions "eq_NPT_328K.mdp"

```
; very basics of the simulation
integrator               = md               ; solve newtown's equation of motion
dt                       = 0.002            ; integration time step / ps
nsteps                   = 5000000          ; number of steps (5000000 * 0.002 = 10
ns)

; remove drifts of the center of mass
comm-mode                = linear    ; remove COM translation
nstcomm                  = 10               ; number of steps for COM removal
comm-grps                = System           ; COM translation removed from system

; control frequency of output
nstvout                 = 0                ; write velocities to trajectory file every
number of steps
nstfout                 = 0                ; write forces to trajectory file every
number of steps
nstlog                   = 5000             ; update log file every number of steps
nstcalcenergy            = 10               ; calculate energies/pressures every
nstenergy steps
nstenergy                = 500              ; write energies to energy file every
number of steps
nstxout-compressed       = 5000             ; write positions using compression (saves
memory, worse quality)
compressed-x-precision   = 1000             ; precision to write compressed trajectory

; next neighbor search and periodic boundary conditions
nstlist                  = 20               ; freq. to update neighbor list & long
range forces (>= 20 w/ GPUs)
rlist                    = 1.4              ; short-range neighbor list cutoff (nm)
cutoff-scheme            = Verlet           ; atom-based neighbor search with an
implicit buffer region
pbc                      = xyz              ; periodicity in x, y and z

; coulomb interaction
coulombtype              = PME              ; particle-mesh ewald summation for long
range (>rcoulomb)
rcoulomb                 = 1.4              ; short-range electrostatic cutoff (in nm)
(with PME,rcoulomb >= rvdw)
coulomb-modifier         = Potential-shift-Verlet ; shifts potential by constant so
potential is 0 at cut-off
```

```
fourierspacing           = 0.168          ; spacing of FFT in reciprocal space in
PME long range treatment (in nm)
pme-order                = 4              ; cubic PME interpolation order

; lennard-jones potential handling
vdwtype                  = cutoff         ; simple truncation cutoff
vdw-modifier             = Potential-shift-Verlet ; shifts potential by constant so
potential is 0 at cut-off
rvdw                     = 1.4            ; short-range vdw cutoff (in nm)
DispCorr                 = no             ; long range correction for energy and
pressure from using vdw cutoff

; temperature coupling
tcoupl                   = v-rescale      ; the algorithm to use, v-rescale
generates correct canonical ensemble
tc-grps                  = System         ; groups to couple to temperature bath
tau-t                    = 1.0            ; time constant (in ps), meaning varies by
algorithm
ref-t                    = 328            ; temperature for coupling (K)
nsttcouple               = 1              ; frequency to couple temperature

; velocity generation
gen-vel                  = yes            ; generate velocities according to Maxwell
distr. (no for initial vel 0)
gen-temp                 = 328            ; temperature for Maxwell distribution (K)
gen-seed                 = -1             ; generate a random seed

; pressure coupling
pcoupl                   = Parrinello-Rahman  ; the algorithm to use (no for NVT)
pcoupltype               = isotropic      ; all ordinates scaled equally (preserves
the cubic box)
compressibility          = 4.5e-5         ; experimental value for water at 298 K
(in 1/bar)
tau-p                    = 5.0            ; time constant (in ps), should be about
4-5 times larger than tau_t
ref-p                    = 1              ; reference pressure (in bar)
nstpcouple               = 1              ; frequency to couple pressure

; constraints
constraints              = h-bonds        ; constrains bonds only involving hydrogen
constraint_algorithm     = lincs          ; algorithm to use, lincs should NOT be
used for angle constraining
lincs-order              = 4              ; highest order in constraint coupling
matrix expansion
lincs-iter               = 1              ; accuracy of lincs algorithm
continuation             = no             ; no for applying constraints at start of
run
```

Molecular dynamics parameter file for production run und constant NVT conditions “md_NVT_328K.mdp”

```
; very basics of the simulation
integrator               = md             ; solve newtown's equation of motion
dt                       = 0.002          ; integration time step / ps
```

```
nsteps                    = 200000000       ; number of steps (200000000 * 0.002 = 400
ns)

; remove drifts of the center of mass
comm-mode                 = linear    ; remove COM translation
nstcomm                   = 10              ; number of steps for COM removal
comm-grps                 = System          ; COM translation removed from system

; control frequency of output
nstvout                  = 0               ; write velocities to trajectory file every
number of steps
nstfout                  = 0               ; write forces to trajectory file every
number of steps
nstlog                    = 40000           ; update log file every number of steps
nstcalcenergy             = 10              ; calculate energies/pressures every
nstenergy steps
nstenergy                 = 10              ; write energies to energy file every
number of steps
nstxout-compressed        = 40000           ; write positions using compression (saves
memory, worse quality)
compressed-x-precision    = 1000            ; precision to write compressed trajectory

; next neighbor search and periodic boundary conditions
nstlist                   = 20              ; freq. to update neighbor list & long
range forces (>= 20 w/ GPUs)
rlist                     = 1.4             ; short-range neighbor list cutoff (nm)
cutoff-scheme             = Verlet          ; atom-based neighbor search with an
implicit buffer region
pbc                       = xyz             ; periodicity in x, y and z

; coulomb interaction
coulombtype               = PME             ; particle-mesh ewald summation for long
range (>rcoulomb)
rcoulomb                  = 1.4             ; short-range electrostatic cutoff (in nm)
(with PME,rcoulomb >= rvdw)
coulomb-modifier          = Potential-shift-Verlet ; shifts potential by constant so
potential is 0 at cut-off
fourierspacing            = 0.168           ; spacing of FFT in reciprocal space in
PME long range treatment (in nm)
pme-order                 = 4               ; cubic PME interpolation order

; lennard-jones potential handling
vdwtype                   = cutoff          ; simple truncation cutoff
vdw-modifier              = Potential-shift-Verlet ; shifts potential by constant so
potential is 0 at cut-off
rvdw                      = 1.4             ; short-range vdw cutoff (in nm)
DispCorr                  = no              ; long range correction for energy and
pressure from using vdw cutoff

; temperature coupling
tcoupl                    = v-rescale       ; the algorithm to use, v-rescale
generates correct canonical ensemble
tc-grps                   = System          ; groups to couple to temperature bath
tau-t                     = 1.0             ; time constant (in ps), meaning varies by
algorithm
ref-t                     = 328             ; temperature for coupling (K)
nsttcouple                = 1               ; frequency to couple temperature
```

```
; velocity generation
gen-vel                  = yes            ; generate velocities according to Maxwell
distr. (no for initial vel 0)
gen-temp                 = 328            ; temperature for Maxwell distribution (K)
gen-seed                 = -1             ; generate a random seed

; pressure coupling
pcoupl                   = no             ; the algorithm to use (no for NVT)

; constraints
constraints              = h-bonds        ; constrains bonds only involving hydrogen
constraint_algorithm     = lincs          ; algorithm to use, lincs should NOT be
used for angle constraining
lincs-order              = 4              ; highest order in constraint coupling
matrix expansion
lincs-iter               = 1              ; accuracy of lincs algorithm
continuation             = no             ; no for applying constraints at start of
run
```

2.3 CHARMM forcefield

2.3.1 Topology files

Example topology file for tetraethylene glycol

```
“top_TeEG_bulk.top”
#include "/home/mmh/BrockportMDFiles/charmm36-feb2021.ff/forcefield.itp"

; additional params for the molecule
#include "TeEG.prm"

#include "TeEG.itp"

[ system ]
; Name
Tetraethylene glycol

[ molecules ]
; Compound        #mols
TeEG 1000


“DEG.itp”
; Created by cgenff_charmm2gmx.py

[ moleculetype ]
; Name                   nrexcl
DEG                     3

[ atoms ]
```

```
;      nr           type      resnr residue  atom   cgnr      charge        mass  typeB
chargeB          massB
; residue   1 DEG rtp DEG q qsum
     1       HGP1      1    DEG     H1      1      0.420      1.008   ;
     2      OG311      1    DEG     O2      2     -0.649     15.999   ;
     3      CG321      1    DEG     C3      3      0.049     12.011   ;
     4       HGA2      1    DEG     H4      4      0.090      1.008   ;
     5       HGA2      1    DEG     H5      5      0.090      1.008   ;
     6      CG321      1    DEG     C6      6     -0.011     12.011   ;
     7       HGA2      1    DEG     H7      7      0.090      1.008   ;
     8       HGA2      1    DEG     H8      8      0.090      1.008   ;
     9      OG301      1    DEG     O9      9     -0.338     15.999   ;
    10      CG321      1    DEG    C10     10     -0.011     12.011   ;
    11       HGA2      1    DEG    H11     11      0.090      1.008   ;
    12       HGA2      1    DEG    H12     12      0.090      1.008   ;
    13      CG321      1    DEG    C13     13      0.049     12.011   ;
    14       HGA2      1    DEG    H14     14      0.090      1.008   ;
    15       HGA2      1    DEG    H15     15      0.090      1.008   ;
    16      OG311      1    DEG    O16     16     -0.649     15.999   ;
    17       HGP1      1    DEG    H17     17      0.420      1.008   ;

[ bonds ]
;     ai      aj funct              c0            c1               c2
     c3
    1     2  1 ;        HGP1      OG311
    2     3  1 ;       OG311      CG321
    3     4  1 ;       CG321       HGA2
    3     5  1 ;       CG321       HGA2
    3     6  1 ;       CG321      CG321
    6     7  1 ;       CG321       HGA2
    6     8  1 ;       CG321       HGA2
    6     9  1 ;       CG321      OG301
    9    10  1 ;       OG301      CG321
   10    11  1 ;       CG321       HGA2
   10    12  1 ;       CG321       HGA2
   10    13  1 ;       CG321      CG321
   13    14  1 ;       CG321       HGA2
   13    15  1 ;       CG321       HGA2
   13    16  1 ;       CG321      OG311
   16    17  1 ;       OG311       HGP1

[ pairs ]
;     ai      aj funct              c0            c1               c2
     c3
    1     4  1
    1     5  1
    1     6  1
    2     7  1
    2     8  1
    2     9  1
    3    10  1
    4     7  1
    4     8  1
    4     9  1
    5     7  1
    5     8  1
    5     9  1
```

```
    6    11  1
    6    12  1
    6    13  1
    7    10  1
    8    10  1
    9    14  1
    9    15  1
    9    16  1
   10    17  1
   11    14  1
   11    15  1
   11    16  1
   12    14  1
   12    15  1
   12    16  1
   14    17  1
   15    17  1

[ angles ]
;     ai      aj  ak funct               c0                  c1              c2
        c3
    1     2     35 ;       HGP1      OG311      CG321
    2     3     45 ;      OG311      CG321       HGA2
    2     3     55 ;      OG311      CG321       HGA2
    2     3     65 ;      OG311      CG321      CG321
    4     3     55 ;       HGA2      CG321       HGA2
    4     3     65 ;       HGA2      CG321      CG321
    5     3     65 ;       HGA2      CG321      CG321
    3     6     75 ;      CG321      CG321       HGA2
    3     6     85 ;      CG321      CG321       HGA2
    3     6     95 ;      CG321      CG321      OG301
    7     6     85 ;       HGA2      CG321       HGA2
    7     6     95 ;       HGA2      CG321      OG301
    8     6     95 ;       HGA2      CG321      OG301
    6     9    105 ;      CG321      OG301      CG321
    9    10    115 ;      OG301      CG321       HGA2
    9    10    125 ;      OG301      CG321       HGA2
    9    10    135 ;      OG301      CG321      CG321
   11    10    125 ;       HGA2      CG321       HGA2
   11    10    135 ;       HGA2      CG321      CG321
   12    10    135 ;       HGA2      CG321      CG321
   10    13    145 ;      CG321      CG321       HGA2
   10    13    155 ;      CG321      CG321       HGA2
   10    13    165 ;      CG321      CG321      OG311
   14    13    155 ;       HGA2      CG321       HGA2
   14    13    165 ;       HGA2      CG321      OG311
   15    13    165 ;       HGA2      CG321      OG311
   13    16    175 ;      CG321      OG311       HGP1

[ dihedrals ]
;     ai      aj  ak      al  funct              c0              c1                  c2
            c3                    c4                c5
    1     2     3     4       9 ;        HGP1       OG311      CG321       HGA2
    1     2     3     5       9 ;        HGP1       OG311      CG321       HGA2
    1     2     3     6       9 ;        HGP1       OG311      CG321      CG321
    2     3     6     7       9 ;       OG311       CG321      CG321       HGA2
    2     3     6     8       9 ;       OG311       CG321      CG321       HGA2
```

```
    2     3     6     9       9 ;     OG311      CG321      CG321      OG301
    4     3     6     7       9 ;      HGA2      CG321      CG321       HGA2
    4     3     6     8       9 ;      HGA2      CG321      CG321       HGA2
    4     3     6     9       9 ;      HGA2      CG321      CG321      OG301
    5     3     6     7       9 ;      HGA2      CG321      CG321       HGA2
    5     3     6     8       9 ;      HGA2      CG321      CG321       HGA2
    5     3     6     9       9 ;      HGA2      CG321      CG321      OG301
    3     6     9    10       9 ;     CG321      CG321      OG301      CG321
    7     6     9    10       9 ;      HGA2      CG321      OG301      CG321
    8     6     9    10       9 ;      HGA2      CG321      OG301      CG321
    6     9    10    11       9 ;     CG321      OG301      CG321       HGA2
    6     9    10    12       9 ;     CG321      OG301      CG321       HGA2
    6     9    10    13       9 ;     CG321      OG301      CG321      CG321
    9    10    13    14       9 ;     OG301      CG321      CG321       HGA2
    9    10    13    15       9 ;     OG301      CG321      CG321       HGA2
    9    10    13    16       9 ;     OG301      CG321      CG321      OG311
   11    10    13    14       9 ;      HGA2      CG321      CG321       HGA2
   11    10    13    15       9 ;      HGA2      CG321      CG321       HGA2
   11    10    13    16       9 ;      HGA2      CG321      CG321      OG311
   12    10    13    14       9 ;      HGA2      CG321      CG321       HGA2
   12    10    13    15       9 ;      HGA2      CG321      CG321       HGA2
   12    10    13    16       9 ;      HGA2      CG321      CG321      OG311
   10    13    16    17       9 ;     CG321      CG321      OG311       HGP1
   14    13    16    17       9 ;      HGA2      CG321      OG311       HGP1
   15    13    16    17       9 ;      HGA2      CG321      OG311       HGP1
```

"TeEG.itp"

```
; Created by cgenff_charmm2gmx.py

[ moleculetype ]
; Name                    nrexcl
TeEG                   3

[ atoms ]
;      nr          type     resnr residue  atom   cgnr      charge        mass  typeB
chargeB          massB
; residue   1 TeEG rtp TeEG q     qsum
     1       HGP1      1   TeEG     H1      1      0.419      1.008   ;
     2      OG311      1   TeEG     O2      2     -0.651     15.999   ;
     3      CG321      1   TeEG     C3      3      0.049     12.011   ;
     4       HGA2      1   TeEG     H4      4      0.090      1.008   ;
     5       HGA2      1   TeEG     H5      5      0.090      1.008   ;
     6      CG321      1   TeEG     C6      6     -0.007     12.011   ;
     7       HGA2      1   TeEG     H7      7      0.090      1.008   ;
     8       HGA2      1   TeEG     H8      8      0.090      1.008   ;
     9      OG301      1   TeEG     O9      9     -0.340     15.999   ;
    10      CG321      1   TeEG    C10     10     -0.010     12.011   ;
    11       HGA2      1   TeEG    H11     11      0.090      1.008   ;
    12       HGA2      1   TeEG    H12     12      0.090      1.008   ;
    13      CG321      1   TeEG    C13     13     -0.010     12.011   ;
    14       HGA2      1   TeEG    H14     14      0.090      1.008   ;
    15       HGA2      1   TeEG    H15     15      0.090      1.008   ;
    16      OG301      1   TeEG    O16     16     -0.340     15.999   ;
    17      CG321      1   TeEG    C17     17     -0.010     12.011   ;
    18       HGA2      1   TeEG    H18     18      0.090      1.008   ;
    19       HGA2      1   TeEG    H19     19      0.090      1.008   ;
    20      CG321      1   TeEG    C20     20     -0.010     12.011   ;
```

```
    21       HGA2      1   TeEG    H21     21      0.090      1.008   ;
    22       HGA2      1   TeEG    H22     22      0.090      1.008   ;
    23      OG301      1   TeEG    O23     23     -0.340     15.999   ;
    24      CG321      1   TeEG    C24     24     -0.007     12.011   ;
    25       HGA2      1   TeEG    H25     25      0.090      1.008   ;
    26       HGA2      1   TeEG    H26     26      0.090      1.008   ;
    27      CG321      1   TeEG    C27     27      0.049     12.011   ;
    28       HGA2      1   TeEG    H28     28      0.090      1.008   ;
    29       HGA2      1   TeEG    H29     29      0.090      1.008   ;
    30      OG311      1   TeEG    O30     30     -0.651     15.999   ;
    31       HGP1      1   TeEG    H31     31      0.419      1.008   ;

[ bonds ]
;     ai      aj  funct              c0             c1                c2
      c3
    1     2  1 ;        HGP1      OG311
    2     3  1 ;       OG311      CG321
    3     4  1 ;       CG321       HGA2
    3     5  1 ;       CG321       HGA2
    3     6  1 ;       CG321      CG321
    6     7  1 ;       CG321       HGA2
    6     8  1 ;       CG321       HGA2
    6     9  1 ;       CG321      OG301
    9    10  1 ;       OG301      CG321
   10    11  1 ;       CG321       HGA2
   10    12  1 ;       CG321       HGA2
   10    13  1 ;       CG321      CG321
   13    14  1 ;       CG321       HGA2
   13    15  1 ;       CG321       HGA2
   13    16  1 ;       CG321      OG301
   16    17  1 ;       OG301      CG321
   17    18  1 ;       CG321       HGA2
   17    19  1 ;       CG321       HGA2
   17    20  1 ;       CG321      CG321
   20    21  1 ;       CG321       HGA2
   20    22  1 ;       CG321       HGA2
   20    23  1 ;       CG321      OG301
   23    24  1 ;       OG301      CG321
   24    25  1 ;       CG321       HGA2
   24    26  1 ;       CG321       HGA2
   24    27  1 ;       CG321      CG321
   27    28  1 ;       CG321       HGA2
   27    29  1 ;       CG321       HGA2
   27    30  1 ;       CG321      OG311
   30    31  1 ;       OG311       HGP1

[ pairs ]
;     ai      aj  funct              c0             c1                c2
      c3
    1     4  1
    1     5  1
    1     6  1
    2     7  1
    2     8  1
    2     9  1
    3    10  1
    4     7  1
```

```
     4      8  1
     4      9  1
     5      7  1
     5      8  1
     5      9  1
     6     11  1
     6     12  1
     6     13  1
     7     10  1
     8     10  1
     9     14  1
     9     15  1
     9     16  1
    10     17  1
    11     14  1
    11     15  1
    11     16  1
    12     14  1
    12     15  1
    12     16  1
    13     18  1
    13     19  1
    13     20  1
    14     17  1
    15     17  1
    16     21  1
    16     22  1
    16     23  1
    17     24  1
    18     21  1
    18     22  1
    18     23  1
    19     21  1
    19     22  1
    19     23  1
    20     25  1
    20     26  1
    20     27  1
    21     24  1
    22     24  1
    23     28  1
    23     29  1
    23     30  1
    24     31  1
    25     28  1
    25     29  1
    25     30  1
    26     28  1
    26     29  1
    26     30  1
    28     31  1
    29     31  1

[ angles ]
;     ai      aj  ak funct                 c0                  c1              c2
         c3
     1      2     35 ;        HGP1      OG311      CG321
```

```
    2     3     45 ;      OG311      CG321       HGA2
    2     3     55 ;      OG311      CG321       HGA2
    2     3     65 ;      OG311      CG321      CG321
    4     3     55 ;       HGA2      CG321       HGA2
    4     3     65 ;       HGA2      CG321      CG321
    5     3     65 ;       HGA2      CG321      CG321
    3     6     75 ;      CG321      CG321       HGA2
    3     6     85 ;      CG321      CG321       HGA2
    3     6     95 ;      CG321      CG321      OG301
    7     6     85 ;       HGA2      CG321       HGA2
    7     6     95 ;       HGA2      CG321      OG301
    8     6     95 ;       HGA2      CG321      OG301
    6     9    105 ;      CG321      OG301      CG321
    9    10    115 ;      OG301      CG321       HGA2
    9    10    125 ;      OG301      CG321       HGA2
    9    10    135 ;      OG301      CG321      CG321
   11    10    125 ;       HGA2      CG321       HGA2
   11    10    135 ;       HGA2      CG321      CG321
   12    10    135 ;       HGA2      CG321      CG321
   10    13    145 ;      CG321      CG321       HGA2
   10    13    155 ;      CG321      CG321       HGA2
   10    13    165 ;      CG321      CG321      OG301
   14    13    155 ;       HGA2      CG321       HGA2
   14    13    165 ;       HGA2      CG321      OG301
   15    13    165 ;       HGA2      CG321      OG301
   13    16    175 ;      CG321      OG301      CG321
   16    17    185 ;      OG301      CG321       HGA2
   16    17    195 ;      OG301      CG321       HGA2
   16    17    205 ;      OG301      CG321      CG321
   18    17    195 ;       HGA2      CG321       HGA2
   18    17    205 ;       HGA2      CG321      CG321
   19    17    205 ;       HGA2      CG321      CG321
   17    20    215 ;      CG321      CG321       HGA2
   17    20    225 ;      CG321      CG321       HGA2
   17    20    235 ;      CG321      CG321      OG301
   21    20    225 ;       HGA2      CG321       HGA2
   21    20    235 ;       HGA2      CG321      OG301
   22    20    235 ;       HGA2      CG321      OG301
   20    23    245 ;      CG321      OG301      CG321
   23    24    255 ;      OG301      CG321       HGA2
   23    24    265 ;      OG301      CG321       HGA2
   23    24    275 ;      OG301      CG321      CG321
   25    24    265 ;       HGA2      CG321       HGA2
   25    24    275 ;       HGA2      CG321      CG321
   26    24    275 ;       HGA2      CG321      CG321
   24    27    285 ;      CG321      CG321       HGA2
   24    27    295 ;      CG321      CG321       HGA2
   24    27    305 ;      CG321      CG321      OG311
   28    27    295 ;       HGA2      CG321       HGA2
   28    27    305 ;       HGA2      CG321      OG311
   29    27    305 ;       HGA2      CG321      OG311
   27    30    315 ;      CG321      OG311       HGP1

[ dihedrals ]
;     ai      aj  ak      al  funct                c0              c1                  c2
           c3                    c4                c5
    1     2     3     4       9 ;       HGP1       OG311      CG321       HGA2
```

1	2	3	5	9	;	HGP1	OG311	CG321	HGA2
1	2	3	6	9	;	HGP1	OG311	CG321	CG321
2	3	6	7	9	;	OG311	CG321	CG321	HGA2
2	3	6	8	9	;	OG311	CG321	CG321	HGA2
2	3	6	9	9	;	OG311	CG321	CG321	OG301
4	3	6	7	9	;	HGA2	CG321	CG321	HGA2
4	3	6	8	9	;	HGA2	CG321	CG321	HGA2
4	3	6	9	9	;	HGA2	CG321	CG321	OG301
5	3	6	7	9	;	HGA2	CG321	CG321	HGA2
5	3	6	8	9	;	HGA2	CG321	CG321	HGA2
5	3	6	9	9	;	HGA2	CG321	CG321	OG301
3	6	9	10	9	;	CG321	CG321	OG301	CG321
7	6	9	10	9	;	HGA2	CG321	OG301	CG321
8	6	9	10	9	;	HGA2	CG321	OG301	CG321
6	9	10	11	9	;	CG321	OG301	CG321	HGA2
6	9	10	12	9	;	CG321	OG301	CG321	HGA2
6	9	10	13	9	;	CG321	OG301	CG321	CG321
9	10	13	14	9	;	OG301	CG321	CG321	HGA2
9	10	13	15	9	;	OG301	CG321	CG321	HGA2
9	10	13	16	9	;	OG301	CG321	CG321	OG301
11	10	13	14	9	;	HGA2	CG321	CG321	HGA2
11	10	13	15	9	;	HGA2	CG321	CG321	HGA2
11	10	13	16	9	;	HGA2	CG321	CG321	OG301
12	10	13	14	9	;	HGA2	CG321	CG321	HGA2
12	10	13	15	9	;	HGA2	CG321	CG321	HGA2
12	10	13	16	9	;	HGA2	CG321	CG321	OG301
10	13	16	17	9	;	CG321	CG321	OG301	CG321
14	13	16	17	9	;	HGA2	CG321	OG301	CG321
15	13	16	17	9	;	HGA2	CG321	OG301	CG321
13	16	17	18	9	;	CG321	OG301	CG321	HGA2
13	16	17	19	9	;	CG321	OG301	CG321	HGA2
13	16	17	20	9	;	CG321	OG301	CG321	CG321
16	17	20	21	9	;	OG301	CG321	CG321	HGA2
16	17	20	22	9	;	OG301	CG321	CG321	HGA2
16	17	20	23	9	;	OG301	CG321	CG321	OG301
18	17	20	21	9	;	HGA2	CG321	CG321	HGA2
18	17	20	22	9	;	HGA2	CG321	CG321	HGA2
18	17	20	23	9	;	HGA2	CG321	CG321	OG301
19	17	20	21	9	;	HGA2	CG321	CG321	HGA2
19	17	20	22	9	;	HGA2	CG321	CG321	HGA2
19	17	20	23	9	;	HGA2	CG321	CG321	OG301
17	20	23	24	9	;	CG321	CG321	OG301	CG321
21	20	23	24	9	;	HGA2	CG321	OG301	CG321
22	20	23	24	9	;	HGA2	CG321	OG301	CG321
20	23	24	25	9	;	CG321	OG301	CG321	HGA2
20	23	24	26	9	;	CG321	OG301	CG321	HGA2
20	23	24	27	9	;	CG321	OG301	CG321	CG321
23	24	27	28	9	;	OG301	CG321	CG321	HGA2
23	24	27	29	9	;	OG301	CG321	CG321	HGA2
23	24	27	30	9	;	OG301	CG321	CG321	OG311
25	24	27	28	9	;	HGA2	CG321	CG321	HGA2
25	24	27	29	9	;	HGA2	CG321	CG321	HGA2
25	24	27	30	9	;	HGA2	CG321	CG321	OG311
26	24	27	28	9	;	HGA2	CG321	CG321	HGA2
26	24	27	29	9	;	HGA2	CG321	CG321	HGA2
26	24	27	30	9	;	HGA2	CG321	CG321	OG311
24	27	30	31	9	;	CG321	CG321	OG311	HGP1

```
   28    27    30    31       9 ;        HGA2       CG321      OG311       HGP1
   29    27    30    31       9 ;        HGA2       CG321      OG311       HGP1
```

2.3.2 Structure Coordinate files

```
Diethylene glycol
   17
    1DEG     H1    1   0.046   0.529  -0.146
    1DEG     O2    2   0.000   0.526  -0.230
    1DEG     C3    3   0.000   0.390  -0.266
    1DEG     H4    4   0.103   0.355  -0.276
    1DEG     H5    5  -0.052   0.331  -0.189
    1DEG     C6    6  -0.072   0.373  -0.400
    1DEG     H7    7  -0.176   0.408  -0.391
    1DEG     H8    8  -0.021   0.431  -0.477
    1DEG     O9    9  -0.072   0.237  -0.437
    1DEG    C10   10  -0.146   0.231  -0.557
    1DEG    H11   11  -0.248   0.268  -0.539
    1DEG    H12   12  -0.098   0.293  -0.633
    1DEG    C13   13  -0.153   0.086  -0.606
    1DEG    H14   14  -0.201   0.024  -0.530
    1DEG    H15   15  -0.051   0.049  -0.624
    1DEG    O16   16  -0.227   0.081  -0.726
    1DEG    H17   17  -0.227  -0.012  -0.750
   0.00000   0.00000   0.00000
Tetraethylene glycol

Tetraethylene glycol
   31
    1TeEG   HA1    1  -0.125   0.212  -0.714
    1TeEG   OA1    2  -0.190   0.146  -0.690
    1TeEG    C1    3  -0.147   0.096  -0.565
    1TeEG   HC1    4  -0.217   0.019  -0.530
    1TeEG   HC2    5  -0.143   0.178  -0.492
    1TeEG    C2    6  -0.008   0.034  -0.580
    1TeEG   HC3    7  -0.012  -0.048  -0.653
    1TeEG   HC4    8   0.062   0.111  -0.615
    1TeEG   OE1    9   0.035  -0.016  -0.456
    1TeEG    C3   10   0.037   0.096  -0.370
    1TeEG   HC5   11   0.106   0.171  -0.410
    1TeEG   HC6   12  -0.064   0.139  -0.364
    1TeEG    C4   13   0.083   0.053  -0.230
    1TeEG   HC7   14   0.084   0.140  -0.164
    1TeEG   HC8   15   0.014  -0.022  -0.191
    1TeEG   OE2   16   0.212  -0.002  -0.238
    1TeEG    C5   17   0.271   0.024  -0.113
    1TeEG   HC9   18   0.327   0.118  -0.118
    1TeEG   HCA   19   0.193   0.032  -0.037
    1TeEG    C6   20   0.367  -0.090  -0.076
    1TeEG   HCB   21   0.445  -0.098  -0.153
    1TeEG   HCC   22   0.311  -0.184  -0.072
    1TeEG   OE3   23   0.425  -0.063   0.049
    1TeEG    C7   24   0.494   0.059   0.032
    1TeEG   HCD   25   0.570   0.048  -0.046
    1TeEG   HCE   26   0.423   0.137   0.004
```

```
    1TeEG     C8   27   0.562   0.097   0.164
    1TeEG    HCF   28   0.633   0.019   0.192
    1TeEG    HCG   29   0.615   0.192   0.151
    1TeEG    OA2   30   0.465   0.112   0.265
    1TeEG    HA2   31   0.514   0.136   0.343
   0.00000   0.00000   0.00000
```

2.3.3 Molecular Dynamics Parameter files

Molecular dynamics parameter file for molecular structure/position equilibrating "em.mdp"

```
; very basics of the simulation
integrator               = steep             ; steepest decent
nsteps                   = 100000            ; number of steepest decent steps
emstep                   = 0.01              ; 0.01 nm step

; next neighbor search and periodic boundary conditions
nstlist                  = 1                 ; freq. to update neighbor list & long
range forces (>= 20 w/ GPUs)
rlist                    = 1.2               ; short-range neighbor list cutoff (nm)
cutoff-scheme            = Verlet            ; atom-based neighbor search with an
implicit buffer region
pbc                      = xyz               ; periodicity in x, y and z

; coulomb interaction
coulombtype              = PME               ; particle-mesh ewald summation for long
range (>rcoulomb)
rcoulomb                 = 1.2               ; short-range electrostatic cutoff (in nm)
(with PME,rcoulomb >= rvdw)
coulomb-modifier         = Potential-shift-Verlet ; shifts potential by constant so
potential is 0 at cut-off
fourierspacing           = 0.144             ; spacing of FFT in reciprocal space in
PME long range treatment (in nm)
pme-order                = 4                 ; cubic PME interpolation order

; lennard-jones potential handling
vdwtype                  = cutoff            ; simple truncation cutoff
rvdw                     = 1.2               ; short-range vdw cutoff (in nm)
vdw-modifier             = force-switch      ; smoothly switch force to 0 between rvdw-
switch and rvdw
rvdw-switch              = 1.0               ; distance to start switching force to 0
```

Molecular dynamics parameter file equilibrating under constant NPT conditions "eq_NPT_328K.mdp"

```
; very basics of the simulation
integrator               = md                ; solve newtown's equation of motion
dt                       = 0.002             ; integration time step / ps
nsteps                   = 5000000           ; number of steps (5000000 * 0.002 = 10
ns)

; remove drifts of the center of mass
comm-mode                = linear    ; remove COM translation
nstcomm                  = 10                ; number of steps for COM removal
comm-grps                = System            ; COM translation removed from system
```

```
; control frequency of output
nstvout                 = 0               ; write velocities to trajectory file every
number of steps
nstfout                 = 0               ; write forces to trajectory file every
number of steps
nstlog                   = 5000            ; update log file every number of steps
nstcalcenergy            = 10              ; calculate energies/pressures every
nstenergy steps
nstenergy                = 50              ; write energies to energy file every
number of steps
nstxout-compressed       = 5000            ; write positions using compression (saves
memory, worse quality)
compressed-x-precision   = 1000            ; precision to write compressed trajectory

; next neighbor search and periodic boundary conditions
nstlist                  = 20              ; freq. to update neighbor list & long
range forces (>= 20 w/ GPUs)
rlist                    = 1.2             ; short-range neighbor list cutoff (nm)
cutoff-scheme            = Verlet          ; atom-based neighbor search with an
implicit buffer region
pbc                      = xyz             ; periodicity in x, y and z

; coulomb interaction
coulombtype              = PME             ; particle-mesh ewald summation for long
range (>rcoulomb)
rcoulomb                 = 1.2             ; short-range electrostatic cutoff (in nm)
(with PME,rcoulomb >= rvdw)
coulomb-modifier         = Potential-shift-Verlet ; shifts potential by constant so
potential is 0 at cut-off
fourierspacing           = 0.144           ; spacing of FFT in reciprocal space in
PME long range treatment (in nm)
pme-order                = 4               ; cubic PME interpolation order

; lennard-jones potential handling
vdwtype                  = cutoff          ; simple truncation cutoff
rvdw                     = 1.2             ; short-range vdw cutoff (in nm)
vdw-modifier             = force-switch    ; smoothly switch force to 0 between rvdw-
switch and rvdw
rvdw-switch              = 1.0             ; distance to start switching force to 0
DispCorr                 = no              ; long range correction for energy and
pressure from using vdw cutoff

; temperature coupling
tcoupl                   = v-rescale       ; the algorithm to use, v-rescale
generates correct canonical ensemble
tc-grps                  = System          ; groups to couple to temperature bath
tau-t                    = 1.0             ; time constant (in ps), meaning varies by
algorithm
ref-t                    = 328             ; temperature for coupling (K)
nsttcouple               = 1               ; frequency to couple temperature

; velocity generation
gen-vel                  = yes             ; generate velocities according to Maxwell
distr. (no for initial vel 0)
gen-temp                 = 328             ; temperature for Maxwell distribution (K)
gen-seed                 = -1              ; generate a random seed
```

```
; pressure coupling
pcoupl                   = Parrinello-Rahman  ; the algorithm to use (no for NVT)
pcoupltype               = isotropic       ; all ordinates scaled equally (preserves
the cubic box)
compressibility          = 4.5e-5          ; experimental value for water at 298 K
(in 1/bar)
tau-p                    = 5.0             ; time constant (in ps), should be about
4-5 times larger than tau_t
ref-p                    = 1               ; reference pressure (in bar)
nstpcouple               = 1               ; frequency to couple pressure

; constraints
constraints              = h-bonds         ; constrains bonds only involving hydrogen
constraint_algorithm     = lincs           ; algorithm to use, lincs should NOT be
used for angle constraining
lincs-order              = 4               ; highest order in constraint coupling
matrix expansion
lincs-iter               = 1               ; accuracy of lincs algorithm
continuation             = no              ; no for applying constraints at start of
run
```

Molecular dynamics parameter file for production run und constant NVT conditions "md_NVT_328K.mdp"

```
; very basics of the simulation
integrator               = md              ; solve newtown's equation of motion
dt                       = 0.002           ; integration time step / ps
nsteps                   = 200000000       ; number of steps (200000000 * 0.002 = 400
ns)

; remove drifts of the center of mass
comm-mode                = linear    ; remove COM translation
nstcomm                  = 10              ; number of steps for COM removal
comm-grps                = System          ; COM translation removed from system

; control frequency of output
nstvout                 = 0               ; write velocities to trajectory file every
number of steps
nstfout                 = 0               ; write forces to trajectory file every
number of steps
nstlog                   = 40000           ; update log file every number of steps
nstcalcenergy            = 10              ; calculate energies/pressures every
nstenergy steps
nstenergy                = 10              ; write energies to energy file every
number of steps
nstxout-compressed       = 40000           ; write positions using compression (saves
memory, worse quality)
compressed-x-precision   = 1000            ; precision to write compressed trajectory

; next neighbor search and periodic boundary conditions
nstlist                  = 20              ; freq. to update neighbor list & long
range forces (>= 20 w/ GPUs)
rlist                    = 1.2             ; short-range neighbor list cutoff (nm)
cutoff-scheme            = Verlet          ; atom-based neighbor search with an
implicit buffer region
pbc                      = xyz             ; periodicity in x, y and z
```

```
; coulomb interaction
coulombtype              = PME              ; particle-mesh ewald summation for long
range (>rcoulomb)
rcoulomb                 = 1.2              ; short-range electrostatic cutoff (in nm)
(with PME,rcoulomb >= rvdw)
coulomb-modifier         = Potential-shift-Verlet ; shifts potential by constant so
potential is 0 at cut-off
fourierspacing           = 0.144            ; spacing of FFT in reciprocal space in
PME long range treatment (in nm)
pme-order                = 4                ; cubic PME interpolation order

; lennard-jones potential handling
vdwtype                  = cutoff           ; simple truncation cutoff
rvdw                     = 1.2              ; short-range vdw cutoff (in nm)
vdw-modifier             = force-switch     ; smoothly switch force to 0 between rvdw-
switch and rvdw
rvdw-switch              = 1.0              ; distance to start switching force to 0
DispCorr                 = no               ; long range correction for energy and
pressure from using vdw cutoff

; temperature coupling
tcoupl                   = v-rescale        ; the algorithm to use, v-rescale
generates correct canonical ensemble
tc-grps                  = System           ; groups to couple to temperature bath
tau-t                    = 1.0              ; time constant (in ps), meaning varies by
algorithm
ref-t                    = 328              ; temperature for coupling (K)
nsttcouple               = 1                ; frequency to couple temperature

; velocity generation
gen-vel                  = yes              ; generate velocities according to Maxwell
distr. (no for initial vel 0)
gen-temp                 = 328              ; temperature for Maxwell distribution (K)
gen-seed                 = -1               ; generate a random seed

; pressure coupling
pcoupl                   = no               ; the algorithm to use (no for NVT)

; constraints
constraints              = h-bonds          ; constrains bonds only involving hydrogen
constraint_algorithm     = lincs            ; algorithm to use, lincs should NOT be
used for angle constraining
lincs-order              = 4                ; highest order in constraint coupling
matrix expansion
lincs-iter               = 1                ; accuracy of lincs algorithm
continuation             = no               ; no for applying constraints at start of
run
```

2.4 AMBER forcefield

2.4.1 Topology file

Diethylene glycol

```
;
;   File DEG.top  was generated
;   By user: mmh (1000)
;   On host: smith_lab215_2.brockport.edu
;   At date: Wed. May  3 10:53:15 2021
;
;   This is a standalone topology file
;
;   Created by:
;   ParmEd:       test_parmed.py, VERSION 3.4.1
;   Executable:   test_parmed.py
;   Library dir:  /usr/local/gromacs/share/gromacs/top
;   Command line:
;     test_parmed.py
;

[ defaults ]
; nbfunc        comb-rule       gen-pairs       fudgeLJ fudgeQQ
1               2               yes             0.5          0.83333333

[ atomtypes ]
; name    at.num    mass    charge ptype  sigma      epsilon
ho             1   1.008000  0.00000000  A              0              0
oh             8  16.000000  0.00000000  A     0.30664734      0.8803136
c3             6  12.010000  0.00000000  A     0.33996695      0.4577296
h1             1   1.008000  0.00000000  A      0.2471353      0.0656888
os             8  16.000000  0.00000000  A     0.30000123        0.71128


[ moleculetype ]
; Name            nrexcl
DEG          3

[ atoms ]
;   nr       type  resnr residue  atom   cgnr    charge       mass  typeB    chargeB
massB
; residue    1 DEG rtp DEG q -0.0
    1         ho      1    DEG    HA1      1 0.40500000   1.008000
    2         oh      1    DEG    OA1      2 -0.59530000  16.000000
    3         c3      1    DEG     C1      3 0.12840000  12.010000
    4         h1      1    DEG    HC1      4 0.05670000   1.008000
    5         h1      1    DEG    HC2      5 0.05670000   1.008000
    6         c3      1    DEG     C2      6 0.09090000  12.010000
    7         h1      1    DEG    HC3      7 0.03720000   1.008000
    8         h1      1    DEG    HC4      8 0.03720000   1.008000
    9         os      1    DEG    OE1      9 -0.43360000  16.000000
   10         c3      1    DEG     C3     10 0.09090000  12.010000
   11         h1      1    DEG    HC5     11 0.03720000   1.008000
   12         h1      1    DEG    HC6     12 0.03720000   1.008000
   13         c3      1    DEG     C4     13 0.12840000  12.010000
   14         h1      1    DEG    HC7     14 0.05670000   1.008000
   15         h1      1    DEG    HC8     15 0.05670000   1.008000
   16         oh      1    DEG    OA2     16 -0.59530000  16.000000
   17         ho      1    DEG    HA2     17 0.40500000   1.008000

[ bonds ]
```

```
;    ai     aj funct         c0         c1         c2         c3
      2      3     1   0.14233 265014.560000
      3      6     1   0.15375 251793.120000
      6      9     1   0.14316 258236.480000
      9     10     1   0.14316 258236.480000
     10     13     1   0.15375 251793.120000
     13     16     1   0.14233 265014.560000
      1      2     1   0.09730 310787.520000
      3      4     1   0.10969 276646.080000
      3      5     1   0.10969 276646.080000
      6      7     1   0.10969 276646.080000
      6      8     1   0.10969 276646.080000
     10     11     1   0.10969 276646.080000
     10     12     1   0.10969 276646.080000
     13     14     1   0.10969 276646.080000
     13     15     1   0.10969 276646.080000
     16     17     1   0.09730 310787.520000

[ pairs ]
;    ai     aj funct         c0         c1         c2         c3
      2      9     1
      3     10     1
      6     13     1
      9     16     1
      1      4     1
      1      5     1
      1      6     1
      2      7     1
      2      8     1
      4      7     1
      4      8     1
      4      9     1
      5      7     1
      5      8     1
      5      9     1
      6     11     1
      6     12     1
      7     10     1
      8     10     1
      9     14     1
      9     15     1
     10     17     1
     11     14     1
     11     15     1
     11     16     1
     12     14     1
     12     15     1
     12     16     1
     14     17     1
     15     17     1

[ angles ]
;    ai     aj     ak funct         c0         c1         c2         c3
      2      3      6     1   110.1900474 564.840000
      3      6      9     1   107.9700462 569.024000
      6      9     10     1   112.4800480 524.673600
      9     10     13     1   107.9700462 569.024000
```

```
    10     13     16     1   110.1900474 564.840000
     1      2      3     1   107.2600461 396.643200
     2      3      4     1   110.2600474 425.931200
     2      3      5     1   110.2600474 425.931200
     3      6      7     1   109.5600471 388.275200
     3      6      8     1   109.5600471 388.275200
     4      3      5     1   108.4600466 328.025600
     4      3      6     1   109.5600471 388.275200
     5      3      6     1   109.5600471 388.275200
     7      6      8     1   108.4600466 328.025600
     7      6      9     1   109.7800468 425.094400
     8      6      9     1   109.7800468 425.094400
     9     10     11     1   109.7800468 425.094400
     9     10     12     1   109.7800468 425.094400
    10     13     14     1   109.5600471 388.275200
    10     13     15     1   109.5600471 388.275200
    11     10     12     1   108.4600466 328.025600
    11     10     13     1   109.5600471 388.275200
    12     10     13     1   109.5600471 388.275200
    13     16     17     1   107.2600461 396.643200
    14     13     15     1   108.4600466 328.025600
    14     13     16     1   110.2600474 425.931200
    15     13     16     1   110.2600474 425.931200

[ dihedrals ]
;    ai     aj     ak     al  funct         c0          c1          c2          c3
c4          c5
     2      3      6      9     1  0.0000000  4.9162000  2
     2      3      6      9     1  0.0000000  0.6024960  3
     3      6      9     10     1  180.0000771  0.4184000  2
     3      6      9     10     1  0.0000000  1.6024720  3
     6      9     10     13     1  180.0000771  0.4184000  2
     6      9     10     13     1  0.0000000  1.6024720  3
     9     10     13     16     1  0.0000000  4.9162000  2
     9     10     13     16     1  0.0000000  0.6024960  3
     1      2      3      4     1  0.0000000  0.6973333  3
     1      2      3      5     1  0.0000000  0.6973333  3
     1      2      3      6     1  0.0000000  1.0460000  1
     1      2      3      6     1  0.0000000  0.6694400  3
     2      3      6      7     1  0.0000000  1.0460000  1
     2      3      6      7     1  0.0000000  0.0000000  3
     2      3      6      8     1  0.0000000  1.0460000  1
     2      3      6      8     1  0.0000000  0.0000000  3
     4      3      6      7     1  0.0000000  0.6508444  3
     4      3      6      8     1  0.0000000  0.6508444  3
     4      3      6      9     1  0.0000000  1.0460000  1
     4      3      6      9     1  0.0000000  0.0000000  3
     5      3      6      7     1  0.0000000  0.6508444  3
     5      3      6      8     1  0.0000000  0.6508444  3
     5      3      6      9     1  0.0000000  1.0460000  1
     5      3      6      9     1  0.0000000  0.0000000  3
     6      9     10     11     1  0.0000000  1.6038667  3
     6      9     10     12     1  0.0000000  1.6038667  3
     7      6      9     10     1  0.0000000  1.6038667  3
     8      6      9     10     1  0.0000000  1.6038667  3
     9     10     13     14     1  0.0000000  1.0460000  1
     9     10     13     14     1  0.0000000  0.0000000  3
```

```
      9     10     13     15     1  0.0000000  1.0460000  1
      9     10     13     15     1  0.0000000  0.0000000  3
     10     13     16     17     1  0.0000000  1.0460000  1
     10     13     16     17     1  0.0000000  0.6694400  3
     11     10     13     14     1  0.0000000  0.6508444  3
     11     10     13     15     1  0.0000000  0.6508444  3
     11     10     13     16     1  0.0000000  1.0460000  1
     11     10     13     16     1  0.0000000  0.0000000  3
     12     10     13     14     1  0.0000000  0.6508444  3
     12     10     13     15     1  0.0000000  0.6508444  3
     12     10     13     16     1  0.0000000  1.0460000  1
     12     10     13     16     1  0.0000000  0.0000000  3
     14     13     16     17     1  0.0000000  0.6973333  3
     15     13     16     17     1  0.0000000  0.6973333  3

[ system ]
; Name
Diethylene glycol

[ molecules ]
; Compound       #mols
DEG             250
```

Tetraethylene glycol

```
;
;   File TeEG.top  was generated
;   By user: mmh (1000)
;   On host: smith_lab215_2.brockport.edu
;   At date: Mon. June  1 10:50:31 2021
;
;   This is a standalone topology file
;
;   Created by:
;   ParmEd:       , VERSION 3.4.1
;   Executable:
;   Library dir:  /usr/local/gromacs/share/gromacs/top
;   Command line:
;
;

[ defaults ]
; nbfunc        comb-rule       gen-pairs       fudgeLJ fudgeQQ
1               2               yes             0.5          0.83333333

[ atomtypes ]
; name    at.num    mass    charge ptype  sigma      epsilon
ho             1   1.008000  0.00000000  A            0            0
oh             8  16.000000  0.00000000  A     0.30664734      0.8803136
c3             6  12.010000  0.00000000  A     0.33996695      0.4577296
h1             1   1.008000  0.00000000  A      0.2471353      0.0656888
os             8  16.000000  0.00000000  A     0.30000123        0.71128


[ moleculetype ]
; Name            nrexcl
```

```
TeEG         3

[ atoms ]
;   nr       type  resnr residue  atom   cgnr    charge       mass  typeB    chargeB
massB
; residue    1 TeEGrtp TeEGq 0.0
    1         ho      1    TeEG   HA1      1 0.39950000   1.008000
    2         oh      1    TeEG   OA1      2 -0.59330000  16.000000
    3         c3      1    TeEG    C1      3 0.09440000  12.010000
    4         h1      1    TeEG   HC1      4 0.04095000   1.008000
    5         h1      1    TeEG   HC2      5 0.04095000   1.008000
    6         c3      1    TeEG    C2      6 0.10990000  12.010000
    7         h1      1    TeEG   HC3      7 0.05870000   1.008000
    8         h1      1    TeEG   HC4      8 0.05870000   1.008000
    9         os      1    TeEG   OE1      9 -0.41660000  16.000000
   10         c3      1    TeEG    C3     10 0.10540000  12.010000
   11         h1      1    TeEG   HC5     11 0.03895000   1.008000
   12         h1      1    TeEG   HC6     12 0.03895000   1.008000
   13         c3      1    TeEG    C4     13 0.12490000  12.010000
   14         h1      1    TeEG   HC7     14 0.05470000   1.008000
   15         h1      1    TeEG   HC8     15 0.05470000   1.008000
   16         os      1    TeEG   OE2     16 -0.42060000  16.000000
   17         c3      1    TeEG    C5     17 0.12390000  12.010000
   18         h1      1    TeEG   HC9     18 0.05470000   1.008000
   19         h1      1    TeEG   HCA     19 0.05470000   1.008000
   20         c3      1    TeEG    C6     20 0.10540000  12.010000
   21         h1      1    TeEG   HCB     21 0.03895000   1.008000
   22         h1      1    TeEG   HCC     22 0.03895000   1.008000
   23         os      1    TeEG   OE3     23 -0.41660000  16.000000
   24         c3      1    TeEG    C7     24 0.10990000  12.010000
   25         h1      1    TeEG   HCD     25 0.05870000   1.008000
   26         h1      1    TeEG   HCE     26 0.05870000   1.008000
   27         c3      1    TeEG    C8     27 0.09440000  12.010000
   28         h1      1    TeEG   HCF     28 0.04095000   1.008000
   29         h1      1    TeEG   HCG     29 0.04095000   1.008000
   30         oh      1    TeEG   OA2     30 -0.59330000  16.000000
   31         ho      1    TeEG   HA2     31 0.39950000   1.008000

[ bonds ]
;    ai     aj funct         c0         c1         c2         c3
      2      3     1   0.14233 265014.560000
      3      6     1   0.15375 251793.120000
      6      9     1   0.14316 258236.480000
      9     10     1   0.14316 258236.480000
     10     13     1   0.15375 251793.120000
     13     16     1   0.14316 258236.480000
     16     17     1   0.14316 258236.480000
     17     20     1   0.15375 251793.120000
     20     23     1   0.14316 258236.480000
     23     24     1   0.14316 258236.480000
     24     27     1   0.15375 251793.120000
     27     30     1   0.14233 265014.560000
      1      2     1   0.09730 310787.520000
      3      4     1   0.10969 276646.080000
      3      5     1   0.10969 276646.080000
      6      7     1   0.10969 276646.080000
      6      8     1   0.10969 276646.080000
```

```
    10     11     1   0.10969 276646.080000
    10     12     1   0.10969 276646.080000
    13     14     1   0.10969 276646.080000
    13     15     1   0.10969 276646.080000
    17     18     1   0.10969 276646.080000
    17     19     1   0.10969 276646.080000
    20     21     1   0.10969 276646.080000
    20     22     1   0.10969 276646.080000
    24     25     1   0.10969 276646.080000
    24     26     1   0.10969 276646.080000
    27     28     1   0.10969 276646.080000
    27     29     1   0.10969 276646.080000
    30     31     1   0.09730 310787.520000

[ pairs ]
;    ai     aj funct          c0          c1          c2          c3
     2      9     1
     3     10     1
     6     13     1
     9     16     1
    10     17     1
    13     20     1
    16     23     1
    17     24     1
    20     27     1
    23     30     1
     1      4     1
     1      5     1
     1      6     1
     2      7     1
     2      8     1
     4      7     1
     4      8     1
     4      9     1
     5      7     1
     5      8     1
     5      9     1
     6     11     1
     6     12     1
     7     10     1
     8     10     1
     9     14     1
     9     15     1
    11     14     1
    11     15     1
    11     16     1
    12     14     1
    12     15     1
    12     16     1
    13     18     1
    13     19     1
    14     17     1
    15     17     1
    16     21     1
    16     22     1
    18     21     1
    18     22     1
```

```
    18     23     1
    19     21     1
    19     22     1
    19     23     1
    20     25     1
    20     26     1
    21     24     1
    22     24     1
    23     28     1
    23     29     1
    24     31     1
    25     28     1
    25     29     1
    25     30     1
    26     28     1
    26     29     1
    26     30     1
    28     31     1
    29     31     1

[ angles ]
;    ai     aj     ak funct          c0          c1          c2          c3
      2      3      6     1   110.1900474 564.840000
      3      6      9     1   107.9700462 569.024000
      6      9     10     1   112.4800480 524.673600
      9     10     13     1   107.9700462 569.024000
     10     13     16     1   107.9700462 569.024000
     13     16     17     1   112.4800480 524.673600
     16     17     20     1   107.9700462 569.024000
     17     20     23     1   107.9700462 569.024000
     20     23     24     1   112.4800480 524.673600
     23     24     27     1   107.9700462 569.024000
     24     27     30     1   110.1900474 564.840000
      1      2      3     1   107.2600461 396.643200
      2      3      4     1   110.2600474 425.931200
      2      3      5     1   110.2600474 425.931200
      3      6      7     1   109.5600471 388.275200
      3      6      8     1   109.5600471 388.275200
      4      3      5     1   108.4600466 328.025600
      4      3      6     1   109.5600471 388.275200
      5      3      6     1   109.5600471 388.275200
      7      6      8     1   108.4600466 328.025600
      7      6      9     1   109.7800468 425.094400
      8      6      9     1   109.7800468 425.094400
      9     10     11     1   109.7800468 425.094400
      9     10     12     1   109.7800468 425.094400
     10     13     14     1   109.5600471 388.275200
     10     13     15     1   109.5600471 388.275200
     11     10     12     1   108.4600466 328.025600
     11     10     13     1   109.5600471 388.275200
     12     10     13     1   109.5600471 388.275200
     14     13     15     1   108.4600466 328.025600
     14     13     16     1   109.7800468 425.094400
     15     13     16     1   109.7800468 425.094400
     16     17     18     1   109.7800468 425.094400
     16     17     19     1   109.7800468 425.094400
     17     20     21     1   109.5600471 388.275200
```

```
     17      20      22      1   109.5600471 388.275200
     18      17      19      1   108.4600466 328.025600
     18      17      20      1   109.5600471 388.275200
     19      17      20      1   109.5600471 388.275200
     21      20      22      1   108.4600466 328.025600
     21      20      23      1   109.7800468 425.094400
     22      20      23      1   109.7800468 425.094400
     23      24      25      1   109.7800468 425.094400
     23      24      26      1   109.7800468 425.094400
     24      27      28      1   109.5600471 388.275200
     24      27      29      1   109.5600471 388.275200
     25      24      26      1   108.4600466 328.025600
     25      24      27      1   109.5600471 388.275200
     26      24      27      1   109.5600471 388.275200
     27      30      31      1   107.2600461 396.643200
     28      27      29      1   108.4600466 328.025600
     28      27      30      1   110.2600474 425.931200
     29      27      30      1   110.2600474 425.931200

[ dihedrals ]
;    ai      aj      ak      al funct
      2       3       6       9      9  0.0000000  4.9162000  2
      2       3       6       9      9  0.0000000  0.6024960  3
      3       6       9      10      9  180.0000771  0.4184000  2
      3       6       9      10      9  0.0000000  1.6024720  3
      6       9      10      13      9  180.0000771  0.4184000  2
      6       9      10      13      9  0.0000000  1.6024720  3
      9      10      13      16      9  0.0000000  4.9162000  2
      9      10      13      16      9  0.0000000  0.6024960  3
     10      13      16      17      9  180.0000771  0.4184000  2
     10      13      16      17      9  0.0000000  1.6024720  3
     13      16      17      20      9  180.0000771  0.4184000  2
     13      16      17      20      9  0.0000000  1.6024720  3
     16      17      20      23      9  0.0000000  4.9162000  2
     16      17      20      23      9  0.0000000  0.6024960  3
     17      20      23      24      9  180.0000771  0.4184000  2
     17      20      23      24      9  0.0000000  1.6024720  3
     20      23      24      27      9  180.0000771  0.4184000  2
     20      23      24      27      9  0.0000000  1.6024720  3
     23      24      27      30      9  0.0000000  4.9162000  2
     23      24      27      30      9  0.0000000  0.6024960  3
      1       2       3       4      9  0.0000000  0.6973333  3
      1       2       3       5      9  0.0000000  0.6973333  3
      1       2       3       6      9  0.0000000  1.0460000  1
      1       2       3       6      9  0.0000000  0.6694400  3
      2       3       6       7      9  0.0000000  1.0460000  1
      2       3       6       7      9  0.0000000  0.0000000  3
      2       3       6       8      9  0.0000000  1.0460000  1
      2       3       6       8      9  0.0000000  0.0000000  3
      4       3       6       7      9  0.0000000  0.6508444  3
      4       3       6       8      9  0.0000000  0.6508444  3
      4       3       6       9      9  0.0000000  1.0460000  1
      4       3       6       9      9  0.0000000  0.0000000  3
      5       3       6       7      9  0.0000000  0.6508444  3
      5       3       6       8      9  0.0000000  0.6508444  3
      5       3       6       9      9  0.0000000  1.0460000  1
      5       3       6       9      9  0.0000000  0.0000000  3
```

```
     6      9     10     11     9  0.0000000  1.6038667  3
     6      9     10     12     9  0.0000000  1.6038667  3
     7      6      9     10     9  0.0000000  1.6038667  3
     8      6      9     10     9  0.0000000  1.6038667  3
     9     10     13     14     9  0.0000000  1.0460000  1
     9     10     13     14     9  0.0000000  0.0000000  3
     9     10     13     15     9  0.0000000  1.0460000  1
     9     10     13     15     9  0.0000000  0.0000000  3
    11     10     13     14     9  0.0000000  0.6508444  3
    11     10     13     15     9  0.0000000  0.6508444  3
    11     10     13     16     9  0.0000000  1.0460000  1
    11     10     13     16     9  0.0000000  0.0000000  3
    12     10     13     14     9  0.0000000  0.6508444  3
    12     10     13     15     9  0.0000000  0.6508444  3
    12     10     13     16     9  0.0000000  1.0460000  1
    12     10     13     16     9  0.0000000  0.0000000  3
    13     16     17     18     9  0.0000000  1.6038667  3
    13     16     17     19     9  0.0000000  1.6038667  3
    14     13     16     17     9  0.0000000  1.6038667  3
    15     13     16     17     9  0.0000000  1.6038667  3
    16     17     20     21     9  0.0000000  1.0460000  1
    16     17     20     21     9  0.0000000  0.0000000  3
    16     17     20     22     9  0.0000000  1.0460000  1
    16     17     20     22     9  0.0000000  0.0000000  3
    18     17     20     21     9  0.0000000  0.6508444  3
    18     17     20     22     9  0.0000000  0.6508444  3
    18     17     20     23     9  0.0000000  1.0460000  1
    18     17     20     23     9  0.0000000  0.0000000  3
    19     17     20     21     9  0.0000000  0.6508444  3
    19     17     20     22     9  0.0000000  0.6508444  3
    19     17     20     23     9  0.0000000  1.0460000  1
    19     17     20     23     9  0.0000000  0.0000000  3
    20     23     24     25     9  0.0000000  1.6038667  3
    20     23     24     26     9  0.0000000  1.6038667  3
    21     20     23     24     9  0.0000000  1.6038667  3
    22     20     23     24     9  0.0000000  1.6038667  3
    23     24     27     28     9  0.0000000  1.0460000  1
    23     24     27     28     9  0.0000000  0.0000000  3
    23     24     27     29     9  0.0000000  1.0460000  1
    23     24     27     29     9  0.0000000  0.0000000  3
    24     27     30     31     9  0.0000000  1.0460000  1
    24     27     30     31     9  0.0000000  0.6694400  3
    25     24     27     28     9  0.0000000  0.6508444  3
    25     24     27     29     9  0.0000000  0.6508444  3
    25     24     27     30     9  0.0000000  1.0460000  1
    25     24     27     30     9  0.0000000  0.0000000  3
    26     24     27     28     9  0.0000000  0.6508444  3
    26     24     27     29     9  0.0000000  0.6508444  3
    26     24     27     30     9  0.0000000  1.0460000  1
    26     24     27     30     9  0.0000000  0.0000000  3
    28     27     30     31     9  0.0000000  0.6973333  3
    29     27     30     31     9  0.0000000  0.6973333  3

[ system ]
; Name
Tetraethylene glycol
```

```
[ molecules ]
; Compound        #mols
TeEG      1000
```

2.4.2 Structure Coordinate file

```
Diethylene glycol
GROningen MAchine for Chemical Simulation
   17
    1DEG    HA1    1  -0.350   0.344  -0.416
    1DEG    OA1    2  -0.395   0.259  -0.390
    1DEG     C1    3  -0.309   0.186  -0.307
    1DEG    HC1    4  -0.303   0.093  -0.343
    1DEG    HC2    5  -0.349   0.183  -0.215
    1DEG     C2    6  -0.174   0.251  -0.305
    1DEG    HC3    7  -0.179   0.343  -0.265
    1DEG    HC4    8  -0.136   0.258  -0.397
    1DEG    OE1    9  -0.087   0.175  -0.226
    1DEG     C3   10   0.045   0.200  -0.266
    1DEG    HC5   11   0.069   0.293  -0.238
    1DEG    HC6   12   0.050   0.194  -0.365
    1DEG     C4   13   0.137   0.101  -0.201
    1DEG    HC7   14   0.229   0.138  -0.191
    1DEG    HC8   15   0.141   0.016  -0.254
    1DEG    OA2   16   0.089   0.069  -0.073
    1DEG    HA2   17   0.134  -0.014  -0.041
   1.26000   1.15400   0.99500

Tetraethylene glycol
   31
    1TeEG   HA1    1   0.019   0.578  -0.121
    1TeEG   OA1    2   0.012   0.543  -0.212
    1TeEG    C1    3   0.005   0.401  -0.204
    1TeEG   HC1    4  -0.080   0.375  -0.140
    1TeEG   HC2    5  -0.018   0.362  -0.304
    1TeEG    C2    6   0.136   0.343  -0.153
    1TeEG   HC3    7   0.164   0.386  -0.057
    1TeEG   HC4    8   0.216   0.369  -0.223
    1TeEG   OE1    9   0.132   0.200  -0.146
    1TeEG    C3   10   0.072   0.152  -0.025
    1TeEG   HC5   11   0.131   0.186   0.061
    1TeEG   HC6   12  -0.030   0.192  -0.014
    1TeEG    C4   13   0.067  -0.001  -0.029
    1TeEG   HC7   14   0.006  -0.032  -0.115
    1TeEG   HC8   15   0.167  -0.043  -0.045
    1TeEG   OE2   16   0.006  -0.054   0.088
    1TeEG    C5   17   0.097  -0.067   0.198
    1TeEG   HC9   18   0.184  -0.125   0.167
    1TeEG   HCA   19   0.127   0.033   0.231
    1TeEG    C6   20   0.026  -0.139   0.314
    1TeEG   HCB   21   0.084  -0.125   0.406
    1TeEG   HCC   22  -0.075  -0.098   0.328
    1TeEG   OE3   23   0.018  -0.279   0.285
    1TeEG    C7   24  -0.058  -0.351   0.382
    1TeEG   HCD   25  -0.148  -0.296   0.411
```

```
    1TeEG   HCE   26  -0.092  -0.444   0.334
    1TeEG    C8   27   0.026  -0.388   0.504
    1TeEG   HCF   28   0.113  -0.448   0.474
    1TeEG   HCG   29   0.064  -0.298   0.554
    1TeEG   OA2   30  -0.052  -0.462   0.597
    1TeEG   HA2   31   0.007  -0.489   0.669
   1.19910   1.25550   1.10550;
```

2.4.3 Molecular Dynamics Parameter file

Molecular dynamics parameter file for molecular structure/position equilibrating "em.mdp"

```
very basics of the simulation
;define                  = -DFLEXIBLE      ; flexible water so simulation does not
crash
integrator               = steep           ; steepest decent
nsteps                   = 100000          ; number of steepest decent steps
emstep                   = 0.01            ; 0.01 nm step

; next neighbor search and periodic boundary conditions
nstlist                  = 1               ; freq. to update neighbor list & long
range forces (>= 20 w/ GPUs)
rlist                    = 1.0             ; short-range neighbor list cutoff (nm)
cutoff-scheme            = Verlet          ; atom-based neighbor search with an
implicit buffer region
pbc                      = xyz             ; periodicity in x, y and z

; coulomb interaction
coulombtype              = PME             ; particle-mesh ewald summation for long
range (>rcoulomb)
rcoulomb                 = 1.0             ; short-range electrostatic cutoff (in nm)
(with PME,rcoulomb >= rvdw)
coulomb-modifier         = Potential-shift ; shifts potential by constant so
potential is 0 at cut-off
fourierspacing           = 0.12            ; spacing of FFT in reciprocal space in
PME long range treatment (in nm)
pme-order                = 4               ; cubic PME interpolation order

; lennard-jones potential handling
vdwtype                  = cutoff          ; simple truncation cutoff
vdw-modifier             = Potential-shift ; shifts potential by constant so
potential is 0 at cut-off
rvdw                     = 1.0             ; short-range vdw cutoff (in nm)
```

Molecular dynamics parameter file equilibrating under constant NPT conditions "eq_NPT_328K.mdp"

```
; very basics of the simulation
integrator               = md              ; solve newtown's equation of motion
dt                       = 0.002           ; integration time step / ps
nsteps                   = 5000000         ; number of steps (5000000 * 0.002 = 10
ns)

; remove drifts of the center of mass
comm-mode                = linear    ; remove COM translation
```

```
nstcomm                  = 10              ; number of steps for COM removal
comm-grps                = System          ; COM translation removed from system

; control frequency of output
nstvout                 = 0               ; write velocities to trajectory file every
number of steps
nstfout                 = 0               ; write forces to trajectory file every
number of steps
nstlog                   = 1000            ; update log file every number of steps
nstcalcenergy            = 10              ; calculate energies/pressures every
nstenergy steps
nstenergy                = 50              ; write energies to energy file every
number of steps
nstxout-compressed       = 1000            ; write positions using compression (saves
memory, worse quality)
compressed-x-precision   = 1000            ; precision to write compressed trajectory

; next neighbor search and periodic boundary conditions
nstlist                  = 20              ; freq. to update neighbor list & long
range forces (>= 20 w/ GPUs)
rlist                    = 1.0             ; short-range neighbor list cutoff (nm)
cutoff-scheme            = Verlet          ; atom-based neighbor search with an
implicit buffer region
pbc                      = xyz             ; periodicity in x, y and z

; coulomb interaction
coulombtype              = PME             ; particle-mesh ewald summation for long
range (>rcoulomb)
rcoulomb                 = 1.0             ; short-range electrostatic cutoff (in nm)
(with PME,rcoulomb >= rvdw)
coulomb-modifier         = Potential-shift ; shifts potential by constant so
potential is 0 at cut-off
fourierspacing           = 0.12            ; spacing of FFT in reciprocal space in
PME long range treatment (in nm)
pme-order                = 4               ; cubic PME interpolation order

; lennard-jones potential handling
vdwtype                  = cutoff          ; simple truncation cutoff
vdw-modifier             = Potential-shift ; shifts potential by constant so
potential is 0 at cut-off
rvdw                     = 1.0             ; short-range vdw cutoff (in nm)
DispCorr                 = EnerPres        ; long range correction for energy and
pressure from using vdw cutoff

; temperature coupling
tcoupl                   = v-rescale       ; the algorithm to use, v-rescale
generates correct canonical ensemble
tc-grps                  = System          ; groups to couple to temperature bath
tau-t                    = 1.0             ; time constant (in ps), meaning varies by
algorithm
ref-t                    = 328             ; temperature for coupling (K)
nsttcouple               = 1               ; frequency to couple temperature

; velocity generation
gen-vel                  = yes             ; generate velocities according to Maxwell
distr. (no for initial vel 0)
gen-temp                 = 328             ; temperature for Maxwell distribution (K)
```

```
gen-seed                 = -1              ; generate a random seed

; pressure coupling
pcoupl                   = Parrinello-Rahman  ; the algorithm to use (no for NVT)
pcoupltype               = isotropic       ; all ordinates scaled equally (preserves
the cubic box)
compressibility          = 4.5e-5          ; experimental value for water at 298 K
(in 1/bar)
tau-p                    = 5.0             ; time constant (in ps), should be about
4-5 times larger than tau_t
ref-p                    = 1               ; reference pressure (in bar)
nstpcouple               = 1               ; frequency to couple pressure

; constraints
constraints              = h-bonds         ; constrains bonds only involving hydrogen
constraint_algorithm     = lincs           ; algorithm to use, lincs should NOT be
used for angle constraining
lincs-order              = 4               ; highest order in constraint coupling
matrix expansion
lincs-iter               = 1               ; accuracy of lincs algorithm
continuation             = no              ; no for applying constraints at start of
run
```

Molecular dynamics parameter file for production run und constant NVT conditions "md_NVT_328K.mdp"

```
; very basics of the simulation
integrator               = md              ; solve newtown's equation of motion
dt                       = 0.002           ; integration time step / ps
nsteps                   = 200000000       ; number of steps (200000000 * 0.002 = 400
ns)

; remove drifts of the center of mass
comm-mode                = linear    ; remove COM translation
nstcomm                  = 10              ; number of steps for COM removal
comm-grps                = System          ; COM translation removed from system

; control frequency of output
nstvout                 = 0               ; write velocities to trajectory file every
number of steps
nstfout                 = 0               ; write forces to trajectory file every
number of steps
nstlog                   = 20000           ; update log file every number of steps
nstcalcenergy            = 10              ; calculate energies/pressures every
nstenergy steps
nstenergy                = 10              ; write energies to energy file every
number of steps
nstxout-compressed       = 20000           ; write positions using compression (saves
memory, worse quality)
compressed-x-precision   = 1000            ; precision to write compressed trajectory

; next neighbor search and periodic boundary conditions
nstlist                  = 20              ; freq. to update neighbor list & long
range forces (>= 20 w/ GPUs)
rlist                    = 1.0             ; short-range neighbor list cutoff (nm)
```

```
cutoff-scheme            = Verlet           ; atom-based neighbor search with an
implicit buffer region
pbc                      = xyz              ; periodicity in x, y and z

; coulomb interaction
coulombtype              = PME              ; particle-mesh ewald summation for long
range (>rcoulomb)
rcoulomb                 = 1.0              ; short-range electrostatic cutoff (in nm)
(with PME,rcoulomb >= rvdw)
coulomb-modifier         = Potential-shift ; shifts potential by constant so
potential is 0 at cut-off
fourierspacing           = 0.12             ; spacing of FFT in reciprocal space in
PME long range treatment (in nm)
pme-order                = 4                ; cubic PME interpolation order

; lennard-jones potential handling
vdwtype                  = cutoff           ; simple truncation cutoff
vdw-modifier             = Potential-shift ; shifts potential by constant so
potential is 0 at cut-off
rvdw                     = 1.0              ; short-range vdw cutoff (in nm)
DispCorr                 = EnerPres         ; long range correction for energy and
pressure from using vdw cutoff

; temperature coupling
tcoupl                   = v-rescale        ; the algorithm to use, v-rescale
generates correct canonical ensemble
tc-grps                  = System           ; groups to couple to temperature bath
tau-t                    = 1.0              ; time constant (in ps), meaning varies by
algorithm
ref-t                    = 328              ; temperature for coupling (K)
nsttcouple               = 1                ; frequency to couple temperature

; velocity generation
gen-vel                  = yes              ; generate velocities according to Maxwell
distr. (no for initial vel 0)
gen-temp                 = 328              ; temperature for Maxwell distribution (K)
gen-seed                 = -1               ; generate a random seed

; pressure coupling
pcoupl                   = no               ; the algorithm to use (no for NVT)

; constraints
constraints              = h-bonds          ; constrains bonds only involving hydrogen
constraint_algorithm     = lincs            ; algorithm to use, lincs should NOT be
used for angle constraining
lincs-order              = 4                ; highest order in constraint coupling
matrix expansion
lincs-iter               = 1                ; accuracy of lincs algorithm
continuation             = no               ; no for applying constraints at start of
run
```

2.5 Martini forcefield

2.5.1 Topology file

```
"top_TeEG.top"
#include "martini_v2.0_PEO_PS_CNP.itp"
#include "TeEG.itp"

[ system ]
Grunewald 2018 Martini 2 forcefield tetraethylene glycol

[ molecules ]
PEO 1000


"TeEG.itp"

; Description:
;   A Martini model of a PEO 27mer terminated by two OH groups
;
; Parameterization:
;   See Grunewald et al., J. Phys. Chem B. 2018
;
; Reference(s):
;   Grunewald, F., Rossi, G., De Vries, A. H., Marrink, S. J., & Monticelli, L.
(2018). A Transferable MARTINI Model of Polyethylene Oxide.
;   The Journal of Physical Chemistry B. doi:10.1021/acs.jpcb.8b04760
;
; Warning(s)/Note(s):
; - The model makes use of a special bead type called EO. For the most part EO has
been verified to work
;   well with the other MARTINI 2 beads especially in the context of the model for
this molecule.
; - This topology can also be used with the polarizable water model of Michalowsky et
al. (i.e. use the v2.2refP particle
;   definitions and run-parameters)
; - Note that the free energies of transfer as well as the radius of gyration for the
PEO parameters
;   to be used with polarizable water are in reasonable agreement with the those
obtained when using the non-polarizable
;   water model. But these parameters have not been as extensively tested.
; - Radius of gyration and Solvation free energy for polarizable water are currently
unpublished but available upon request
;
;

[   moleculetype   ]
PEO   1
;
[      atoms       ]
;
1    SP2   1   PEO   SP21   1    0.000 31
2    EO    1   PEO    EO1   2    0.000 44
3    EO    1   PEO    EO2   3    0.000 44
4    EO    1   PEO    EO3   4    0.000 44
5    SP2   1   PEO   SP22   5    0.000 31
```

```
;
[      bonds       ]
;
1     2     1  0.2800   7000.00
2     3     1  0.3220   7000.00
3     4     1  0.3220   7000.00
4     5     1  0.2800   7000.00
[      angles      ]
1     2     3     2  140.0    25.00
2     3     4     10 135.00   75.00
2     3     4     2  135.00   50.00
3     4     5     2  140.0    25.00
[    dihedrals     ]
```

2.5.2 Structure Coordinate file

```
Tetraethylene glycol
 5
    1PEO   SP21    1   1.400   4.034   3.525  0.1910 -0.1510 -0.0080
    1PEO    EO1    2   1.253   4.167   3.699  0.0903 -0.3073  0.4153
    1PEO    EO2    3   1.244   4.480   3.708  0.3374 -0.1818 -0.2497
    1PEO    EO3    4   1.399   4.728   3.854 -0.5408 -0.0337 -0.2379
    1PEO   SP22    5   1.633   4.894   3.784 -0.0628  0.0033  0.3826
   9.00318  10.04484   9.25309
```

2.5.3 Molecular Dynamics Parameter file

Molecular dynamics parameter file for molecular structure/position equilibrating "em.mdp"

```
; very basics of the simulation
;define                   = -DFLEXIBLE      ; flexible water so simulation does not
crash
integrator               = steep           ; steepest decent
nsteps                   = 100000          ; number of steepest decent steps
emstep                   = 0.01            ; 0.01 nm step

; next neighbor search and periodic boundary conditions
nstlist                  = 1               ; freq. to update neighbor list & long
range forces (>= 20 w/ GPUs)
rlist                    = 1.1             ; short-range neighbor list cutoff (nm)
cutoff-scheme            = Verlet          ; atom-based neighbor search with an
implicit buffer region
pbc                      = xyz             ; periodicity in x, y and z

; coulomb interaction
coulombtype              = Reaction-field  ; reaction-field electrostatics
rcoulomb                 = 1.1             ; short-range electrostatic cutoff (in nm)
(with PME,rcoulomb >= rvdw)
coulomb-modifier         = Potential-shift ; shifts potential by constant so
potential is 0 at cut-off
epsilon-r                = 15
epsilon-rf               = 0
```

```
; lennard-jones potential handling
vdwtype                  = cutoff          ; simple truncation cutoff
vdw-modifier             = Potential-shift ; shifts potential by constant so
potential is 0 at cut-off
rvdw                     = 1.1             ; short-range vdw cutoff (in nm)
DispCorr                 = no
```

Molecular dynamics parameter file equilibrating under constant NPT conditions "eq_NPT_328K.mdp"

```
; very basics of the simulation
integrator               = md              ; solve newtown's equation of motion
dt                       = 0.020           ; integration time step / ps
nsteps                   = 500000          ; number of steps (500000 * 0.020 = 10 ns)

; remove drifts of the center of mass
comm-mode                = linear    ; remove COM translation
nstcomm                  = 10              ; number of steps for COM removal
comm-grps                = System          ; COM translation removed from system

; control frequency of output
nstvout                 = 0               ; write velocities to trajectory file every
number of steps
nstfout                 = 0               ; write forces to trajectory file every
number of steps
nstlog                   = 500             ; update log file every number of steps
nstcalcenergy            = 10              ; calculate energies/pressures every
nstenergy steps
nstenergy                = 50              ; write energies to energy file every
number of steps
nstxout-compressed       = 500             ; write positions using compression (saves
memory, worse quality)
compressed-x-precision   = 1000            ; precision to write compressed trajectory

; next neighbor search and periodic boundary conditions
nstlist                  = 20              ; freq. to update neighbor list & long
range forces (>= 20 w/ GPUs)
rlist                    = 1.1             ; short-range neighbor list cutoff (nm)
cutoff-scheme            = Verlet          ; atom-based neighbor search with an
implicit buffer region
pbc                      = xyz             ; periodicity in x, y and z

; coulomb interaction
coulombtype              = Reaction-field  ; reaction-field electrostatics
rcoulomb                 = 1.1             ; short-range electrostatic cutoff (in nm)
(with PME,rcoulomb >= rvdw)
coulomb-modifier         = Potential-shift ; shifts potential by constant so
potential is 0 at cut-off
epsilon-r                = 15
epsilon-rf               = 0

; lennard-jones potential handling
vdwtype                  = cutoff          ; simple truncation cutoff
vdw-modifier             = Potential-shift ; shifts potential by constant so
potential is 0 at cut-off
rvdw                     = 1.1             ; short-range vdw cutoff (in nm)
```

```
DispCorr                 = no               ; long range correction for energy and
pressure from using vdw cutoff

; temperature coupling
tcoupl                   = v-rescale        ; the algorithm to use, v-rescale
generates correct canonical ensemble
tc-grps                  = System           ; groups to couple to temperature bath
tau-t                    = 1.0              ; time constant (in ps), meaning varies by
algorithm
ref-t                    = 328              ; temperature for coupling (K)
nsttcouple               = 1                ; frequency to couple temperature

; velocity generation
gen-vel                  = yes              ; generate velocities according to Maxwell
distr. (no for initial vel 0)
gen-temp                 = 328              ; temperature for Maxwell distribution (K)
gen-seed                 = -1               ; generate a random seed

; pressure coupling
pcoupl                   = Parrinello-Rahman  ; the algorithm to use (no for NVT)
pcoupltype               = isotropic        ; all ordinates scaled equally (preserves
the cubic box)
compressibility          = 4.5e-5           ; experimental value for water at 298 K
(in 1/bar)
tau-p                    = 5.0              ; time constant (in ps), should be about
4-5 times larger than tau_t
ref-p                    = 1                ; reference pressure (in bar)
nstpcouple               = 1                ; frequency to couple pressure

; constraints
constraints              = none             ; constrains bonds only involving hydrogen
```

Molecular dynamics parameter file for production run und constant NVT conditions “md_NVT_328K.mdp”

```
; very basics of the simulation
integrator               = md               ; solve newtown's equation of motion
dt                       = 0.020            ; integration time step / ps
nsteps                   = 15000000           ; number of steps (15000000 * 0.020 =
300 ns)

; remove drifts of the center of mass
comm-mode                = linear    ; remove COM translation
nstcomm                  = 10               ; number of steps for COM removal
comm-grps                = System           ; COM translation removed from system

; control frequency of output
nstvout                 = 0               ; write velocities to trajectory file every
number of steps
nstfout                 = 0               ; write forces to trajectory file every
number of steps
nstlog                   = 500              ; update log file every number of steps
nstcalcenergy            = 10               ; calculate energies/pressures every
nstenergy steps
```

```
nstenergy                = 50                ; write energies to energy file every
number of steps
nstxout-compressed       = 500               ; write positions using compression (saves
memory, worse quality)
compressed-x-precision   = 1000              ; precision to write compressed trajectory

; next neighbor search and periodic boundary conditions
nstlist                  = 20                ; freq. to update neighbor list & long
range forces (>= 20 w/ GPUs)
rlist                    = 1.1               ; short-range neighbor list cutoff (nm)
cutoff-scheme            = Verlet            ; atom-based neighbor search with an
implicit buffer region
pbc                      = xyz               ; periodicity in x, y and z

; coulomb interaction
coulombtype              = Reaction-field  ; reaction-field electrostatics
rcoulomb                 = 1.1               ; short-range electrostatic cutoff (in nm)
(with PME,rcoulomb >= rvdw)
coulomb-modifier         = Potential-shift ; shifts potential by constant so
potential is 0 at cut-off
epsilon-r                = 15
epsilon-rf               = 0

; lennard-jones potential handling
vdwtype                  = cutoff            ; simple truncation cutoff
vdw-modifier             = Potential-shift ; shifts potential by constant so
potential is 0 at cut-off
rvdw                     = 1.1               ; short-range vdw cutoff (in nm)
DispCorr                 = no                ; long range correction for energy and
pressure from using vdw cutoff

; temperature coupling
tcoupl                   = v-rescale         ; the algorithm to use, v-rescale
generates correct canonical ensemble
tc-grps                  = System            ; groups to couple to temperature bath
tau-t                    = 1.0               ; time constant (in ps), meaning varies by
algorithm
ref-t                    = 328               ; temperature for coupling (K)
nsttcouple               = 1                 ; frequency to couple temperature

; velocity generation
gen-vel                  = yes               ; generate velocities according to Maxwell
distr. (no for initial vel 0)
gen-temp                 = 328               ; temperature for Maxwell distribution (K)
gen-seed                 = -1                ; generate a random seed

; pressure coupling
pcoupl                   = no                ; the algorithm to use (no for NVT)

; constraints
constraints              = none              ; constrains bonds only involving hydrogen
```

2.6 CZMP forcefield

2.6.1 Topology file

```
"Top_TeEG_bulk.top"
; parameter-level topology
#include "parameters.itp"

; molecular-level topology
#include "TeEG.itp"
#include "spce.itp"

; system-level topology
[ system ]
Tetraethylene glycol

[ molecules ]
TeEG 250

"TeEG.itp"
; Tetraethylene glycol molecular-level topology

[ moleculetype ]
; Name              nrexcl
TeEG                  3

[ atoms ]
;   nr type  resnr residue  atom   cgnr     charge       mass
     1   H       1    TeEG   HA1      1      0.400     1.0080
     2   O       1    TeEG   OA1      1     -0.700    15.9990
     3   C       1    TeEG   C1       1      0.106    12.0110
     4   H       1    TeEG   HC1      1      0.097     1.0080
     5   H       1    TeEG   HC2      1      0.097     1.0080
     6   C       1    TeEG   C2       1     -0.066    12.0110
     7   H       1    TeEG   HC3      1      0.097     1.0080
     8   H       1    TeEG   HC4      1      0.097     1.0080
     9   O       1    TeEG   OE1      1     -0.256    15.9990
    10   C       1    TeEG   C3       1     -0.066    12.0110
    11   H       1    TeEG   HC5      1      0.097     1.0080
    12   H       1    TeEG   HC6      1      0.097     1.0080
    13   C       1    TeEG   C4       1     -0.066    12.0110
    14   H       1    TeEG   HC7      1      0.097     1.0080
    15   H       1    TeEG   HC8      1      0.097     1.0080
    16   O       1    TeEG   OE2      1     -0.256    15.9990
    17   C       1    TeEG   C5       1     -0.066    12.0110
    18   H       1    TeEG   HC9      1      0.097     1.0080
    19   H       1    TeEG   HCA      1      0.097     1.0080
    20   C       1    TeEG   C6       1     -0.066    12.0110
    21   H       1    TeEG   HCB      1      0.097     1.0080
    22   H       1    TeEG   HCC      1      0.097     1.0080
    23   O       1    TeEG   OE3      1     -0.256    15.9990
    24   C       1    TeEG   C7       1     -0.066    12.0110
    25   H       1    TeEG   HCD      1      0.097     1.0080
    26   H       1    TeEG   HCE      1      0.097     1.0080
    27   C       1    TeEG   C8       1      0.106    12.0110
    28   H       1    TeEG   HCF      1      0.097     1.0080
```

```
    29   H       1    TeEG   HCG      1      0.097     1.0080
    30   O       1    TeEG   OA2      1     -0.700    15.9990
    31   H       1    TeEG   HA2      1      0.400     1.0080

[ constraints ]
;  ai    aj     funct
    2     1     1
    3     2     1
    4     3     1
    5     3     1
    6     3     1
    7     6     1
    8     6     1
    9     6     1
   10     9     1
   11    10     1
   12    10     1
   13    10     1
   14    13     1
   15    13     1
   16    13     1
   17    16     1
   18    17     1
   19    17     1
   20    17     1
   21    20     1
   22    20     1
   23    20     1
   24    23     1
   25    24     1
   26    24     1
   27    24     1
   28    27     1
   29    27     1
   30    27     1
   31    30     1

[ angles ]
;  ai    aj    ak funct
    1     2     3     1
    2     3     4     1
    2     3     5     1
    2     3     6     1
    3     6     7     1
    3     6     8     1
    3     6     9     1
    6     9    10     1
    9    10    11     1
    9    10    12     1
    9    10    13     1
   10    13    14     1
   10    13    15     1
   10    13    16     1
   13    16    17     1
   16    17    18     1
   16    17    19     1
   16    17    20     1
```

```
   17    20    21     1
   17    20    22     1
   17    20    23     1
   20    23    24     1
   23    24    25     1
   23    24    26     1
   23    24    27     1
   24    27    28     1
   24    27    29     1
   24    27    30     1
   27    30    31     1
   28    27    30     1
   15    13    16     1
   26    24    27     1
   11    10    12     1
   21    20    22     1
    7     6     9     1
   12    10    13     1
   18    17    20     1
   28    27    29     1
   14    13    15     1
   21    20    23     1
    4     3     5     1
   25    24    26     1
   18    17    19     1
   22    20    23     1
    7     6     8     1
    4     3     6     1
   29    27    30     1
    8     6     9     1
   11    10    13     1
   14    13    16     1
    5     3     6     1
   19    17    20     1
   25    24    27     1

[ dihedrals ]
; PROPER DIHEDRAL ANGLES
;  ai    aj    ak    al funct
    6    3    2    1        9
   20   17   16   13        9
   27   24   23   20        9
   13   10    9    6        9
   24   23   20   17        9
   17   16   13   10        9
   10    9    6    3        9
   10    9    6    7        9
   24   23   20   22        9
   10    9    6    8        9
   17   16   13   15        9
   24   23   20   21        9
   17   16   13   14        9
   29   27   24   25        9
   14   13   10   12        9
   14   13   10   11        9
   15   13   10   11        9
   21   20   17   18        9
```

```
     8     6     3     4           9
    28    27    24    26           9
    22    20    17    19           9
    21    20    17    19           9
    29    27    24    26           9
     7     6     3     5           9
     8     6     3     5           9
    15    13    10    12           9
    28    27    24    25           9
    22    20    17    18           9
     7     6     3     4           9
     7     6     3     2           9
     8     6     3     2           9
    29    27    24    23           9
    21    20    17    16           9
    15    13    10     9           9
    22    20    17    16           9
    14    13    10     9           9
    28    27    24    23           9
     4     3     2     1           9
     5     3     2     1           9
    11    10     9     6           9
    18    17    16    13           9
    12    10     9     6           9
    25    24    23    20           9
    26    24    23    20           9
    19    17    16    13           9
    31    30    27    24           9
    31    30    27    29           9
    31    30    27    28           9
    30    27    24    25           9
    30    27    24    26           9
    30    27    24    23           9
    23    20    17    19           9
    16    13    10    12           9
     9     6     3     4           9
     9     6     3     5           9
    23    20    17    18           9
    16    13    10    11           9
     9     6     3     2           9
    16    13    10     9           9
    23    20    17    16           9

[ pairs ]
      1     4     1
      1     5     1
      1     6     1
      2     7     1
      2     8     1
      4     7     1
      2     9     1
      5     7     1
      4     8     1
      5     8     1
      4     9     1
      3    10     1
      5     9     1
```

```
      7    10    1
      6    11    1
      8    10    1
      6    12    1
      6    13    1
      9    14    1
      9    15    1
     11    14    1
      9    16    1
     12    14    1
     11    15    1
     12    15    1
     11    16    1
     10    17    1
     12    16    1
     14    17    1
     13    18    1
     15    17    1
     13    19    1
     13    20    1
     16    21    1
     16    22    1
     18    21    1
     16    23    1
     19    21    1
     18    22    1
     19    22    1
     18    23    1
     17    24    1
     19    23    1
     21    24    1
     20    25    1
     22    24    1
     20    26    1
     20    27    1
     23    28    1
     23    29    1
     25    28    1
     23    30    1
     26    28    1
     25    29    1
     26    29    1
     25    30    1
     24    31    1
     26    30    1
     28    31    1
     29    31    1
```

2.6.2 Structure Coordinate file

```
Tetraethylene glycol
   31
    1TeEG   HA1    1   0.019   0.578  -0.121
    1TeEG   OA1    2   0.012   0.543  -0.212
    1TeEG    C1    3   0.005   0.401  -0.204
```

```
    1TeEG   HC1    4  -0.080   0.375  -0.140
    1TeEG   HC2    5  -0.018   0.362  -0.304
    1TeEG    C2    6   0.136   0.343  -0.153
    1TeEG   HC3    7   0.164   0.386  -0.057
    1TeEG   HC4    8   0.216   0.369  -0.223
    1TeEG   OE1    9   0.132   0.200  -0.146
    1TeEG    C3   10   0.072   0.152  -0.025
    1TeEG   HC5   11   0.131   0.186   0.061
    1TeEG   HC6   12  -0.030   0.192  -0.014
    1TeEG    C4   13   0.067  -0.001  -0.029
    1TeEG   HC7   14   0.006  -0.032  -0.115
    1TeEG   HC8   15   0.167  -0.043  -0.045
    1TeEG   OE2   16   0.006  -0.054   0.088
    1TeEG    C5   17   0.097  -0.067   0.198
    1TeEG   HC9   18   0.184  -0.125   0.167
    1TeEG   HCA   19   0.127   0.033   0.231
    1TeEG    C6   20   0.026  -0.139   0.314
    1TeEG   HCB   21   0.084  -0.125   0.406
    1TeEG   HCC   22  -0.075  -0.098   0.328
    1TeEG   OE3   23   0.018  -0.279   0.285
    1TeEG    C7   24  -0.058  -0.351   0.382
    1TeEG   HCD   25  -0.148  -0.296   0.411
    1TeEG   HCE   26  -0.092  -0.444   0.334
    1TeEG    C8   27   0.026  -0.388   0.504
    1TeEG   HCF   28   0.113  -0.448   0.474
    1TeEG   HCG   29   0.064  -0.298   0.554
    1TeEG   OA2   30  -0.052  -0.462   0.597
    1TeEG   HA2   31   0.007  -0.489   0.669
   0.00000   0.00000   0.00000
```

2.6.3 Molecular Dynamics Parameter file

Molecular dynamics parameter file for molecular structure/position equilibrating "em.mdp"

```
; very basics of the simulation
define                   = -DFLEXIBLE      ; flexible water so simulation does not
crash
integrator               = steep           ; steepest decent
nsteps                   = 100000          ; number of steepest decent steps
emstep                   = 0.01            ; 0.01 nm step

; next neighbor search and periodic boundary conditions
nstlist                  = 1               ; freq. to update neighbor list & long
range forces (>= 20 w/ GPUs)
rlist                    = 1.0             ; short-range neighbor list cutoff (nm)
cutoff-scheme            = Verlet          ; atom-based neighbor search with an
implicit buffer region
pbc                      = xyz             ; periodicity in x, y and z

; coulomb interaction
coulombtype              = PME             ; particle-mesh ewald summation for long
range (>rcoulomb)
rcoulomb                 = 1.0             ; short-range electrostatic cutoff (in nm)
(with PME,rcoulomb >= rvdw)
```

```
coulomb-modifier         = Potential-shift ; shifts potential by constant so
potential is 0 at cut-off
fourierspacing           = 0.12            ; spacing of FFT in reciprocal space in
PME long range treatment (in nm)
pme-order                = 4               ; cubic PME interpolation order

; lennard-jones potential handling
vdwtype                  = cutoff          ; simple truncation cutoff
vdw-modifier             = Potential-shift ; shifts potential by constant so
potential is 0 at cut-off
rvdw                     = 1.0             ; short-range vdw cutoff (in nm)
```

Molecular dynamics parameter file equilibrating under constant NPT conditions "eq_NPT_328K.mdp"

```
; very basics of the simulation
integrator               = md              ; solve newtown's equation of motion
dt                       = 0.002           ; integration time step / ps
nsteps                   = 5000000         ; number of steps (5000000 * 0.002 = 10
ns)

; remove drifts of the center of mass
comm-mode                = linear    ; remove COM translation
nstcomm                  = 10              ; number of steps for COM removal
comm-grps                = System          ; COM translation removed from system

; control frequency of output
nstvout                 = 0               ; write velocities to trajectory file every
number of steps
nstfout                 = 0               ; write forces to trajectory file every
number of steps
nstlog                   = 1000            ; update log file every number of steps
nstcalcenergy            = 10              ; calculate energies/pressures every
nstenergy steps
nstenergy                = 500             ; write energies to energy file every
number of steps
nstxout-compressed       = 1000            ; write positions using compression (saves
memory, worse quality)
compressed-x-precision   = 1000            ; precision to write compressed trajectory

; next neighbor search and periodic boundary conditions
nstlist                  = 20              ; freq. to update neighbor list & long
range forces (>= 20 w/ GPUs)
rlist                    = 1.0             ; short-range neighbor list cutoff (nm)
cutoff-scheme            = Verlet          ; atom-based neighbor search with an
implicit buffer region
pbc                      = xyz             ; periodicity in x, y and z

; coulomb interaction
coulombtype              = PME             ; particle-mesh ewald summation for long
range (>rcoulomb)
rcoulomb                 = 1.0             ; short-range electrostatic cutoff (in nm)
(with PME,rcoulomb >= rvdw)
coulomb-modifier         = Potential-shift ; shifts potential by constant so
potential is 0 at cut-off
fourierspacing           = 0.12            ; spacing of FFT in reciprocal space in
PME long range treatment (in nm)
pme-order                = 4               ; cubic PME interpolation order
```

```
; lennard-jones potential handling
vdwtype                  = cutoff          ; simple truncation cutoff
vdw-modifier             = Potential-shift ; shifts potential by constant so
potential is 0 at cut-off
rvdw                     = 1.0             ; short-range vdw cutoff (in nm)
DispCorr                 = EnerPres        ; long range correction for energy and
pressure from using vdw cutoff

; temperature coupling
tcoupl                   = v-rescale       ; the algorithm to use, v-rescale
generates correct canonical ensemble
tc-grps                  = System          ; groups to couple to temperature bath
tau-t                    = 1.0             ; time constant (in ps), meaning varies by
algorithm
ref-t                    = 328             ; temperature for coupling (K)
nsttcouple               = 1               ; frequency to couple temperature

; velocity generation
gen-vel                  = yes             ; generate velocities according to Maxwell
distr. (no for initial vel 0)
gen-temp                 = 328             ; temperature for Maxwell distribution (K)
gen-seed                 = -1              ; generate a random seed

; pressure coupling
pcoupl                   = Parrinello-Rahman  ; the algorithm to use (no for NVT)
pcoupltype               = isotropic       ; all ordinates scaled equally (preserves
the cubic box)
compressibility          = 4.5e-5          ; experimental value for water at 298 K
(in 1/bar)
tau-p                    = 5.0             ; time constant (in ps), should be about
4-5 times larger than tau_t
ref-p                    = 1               ; reference pressure (in bar)
nstpcouple               = 1               ; frequency to couple pressure

; constraints
constraints              = all-bonds       ; constrains all bonds
constraint_algorithm     = lincs           ; algorithm to use, lincs should NOT be
used for angle constraining
lincs-order              = 4               ; highest order in constraint coupling
matrix expansion
lincs-iter               = 1               ; accuracy of lincs algorithm
continuation             = no              ; no for applying constraints at start of
run
```

Molecular dynamics parameter file for production run und constant NVT conditions “md_NVT_328K.mdp”

```
; very basics of the simulation
integrator               = md              ; solve newtown's equation of motion
dt                       = 0.002           ; integration time step / ps
nsteps                   = 50000000        ; number of steps (50000000 * 0.002 = 100
ns)

; remove drifts of the center of mass
```

```
comm-mode                = linear    ; remove COM translation
nstcomm                  = 10              ; number of steps for COM removal
comm-grps                = System          ; COM translation removed from system

; control frequency of output
nstvout                 = 0               ; write velocities to trajectory file every
number of steps
nstfout                 = 0               ; write forces to trajectory file every
number of steps
nstlog                   = 10000           ; update log file every number of steps
nstcalcenergy            = 10              ; calculate energies/pressures every
nstenergy steps
nstenergy                = 10              ; write energies to energy file every
number of steps
nstxout-compressed       = 10000           ; write positions using compression (saves
memory, worse quality)
compressed-x-precision   = 1000            ; precision to write compressed trajectory

; next neighbor search and periodic boundary conditions
nstlist                  = 20              ; freq. to update neighbor list & long
range forces (>= 20 w/ GPUs)
rlist                    = 1.0             ; short-range neighbor list cutoff (nm)
cutoff-scheme            = Verlet          ; atom-based neighbor search with an
implicit buffer region
pbc                      = xyz             ; periodicity in x, y and z

; coulomb interaction
coulombtype              = PME             ; particle-mesh ewald summation for long
range (>rcoulomb)
rcoulomb                 = 1.0             ; short-range electrostatic cutoff (in nm)
(with PME,rcoulomb >= rvdw)
coulomb-modifier         = Potential-shift ; shifts potential by constant so
potential is 0 at cut-off
fourierspacing           = 0.12            ; spacing of FFT in reciprocal space in
PME long range treatment (in nm)
pme-order                = 4               ; cubic PME interpolation order

; lennard-jones potential handling
vdwtype                  = cutoff          ; simple truncation cutoff
vdw-modifier             = Potential-shift ; shifts potential by constant so
potential is 0 at cut-off
rvdw                     = 1.0             ; short-range vdw cutoff (in nm)
DispCorr                 = EnerPres        ; long range correction for energy and
pressure from using vdw cutoff

; temperature coupling
tcoupl                   = v-rescale       ; the algorithm to use, v-rescale
generates correct canonical ensemble
tc-grps                  = System          ; groups to couple to temperature bath
tau-t                    = 1.0             ; time constant (in ps), meaning varies by
algorithm
ref-t                    = 328             ; temperature for coupling (K)
nsttcouple               = 1               ; frequency to couple temperature

; velocity generation
gen-vel                  = yes             ; generate velocities according to Maxwell
distr. (no for initial vel 0)
```

```
gen-temp                 = 328              ; temperature for Maxwell distribution (K)
gen-seed                 = -1               ; generate a random seed

; pressure coupling
pcoupl                   = no               ; the algorithm to use (no for NVT)

; constraints
constraints              = all-bonds        ; constrains all bonds
constraint_algorithm     = lincs            ; algorithm to use, lincs should NOT be
used for angle constraining
lincs-order              = 4                ; highest order in constraint coupling
matrix expansion
lincs-iter               = 1                ; accuracy of lincs algorithm
continuation             = no               ; no for applying constraints at start of
run
```

3 Comparison of dihedral potential functions used by different forcefields

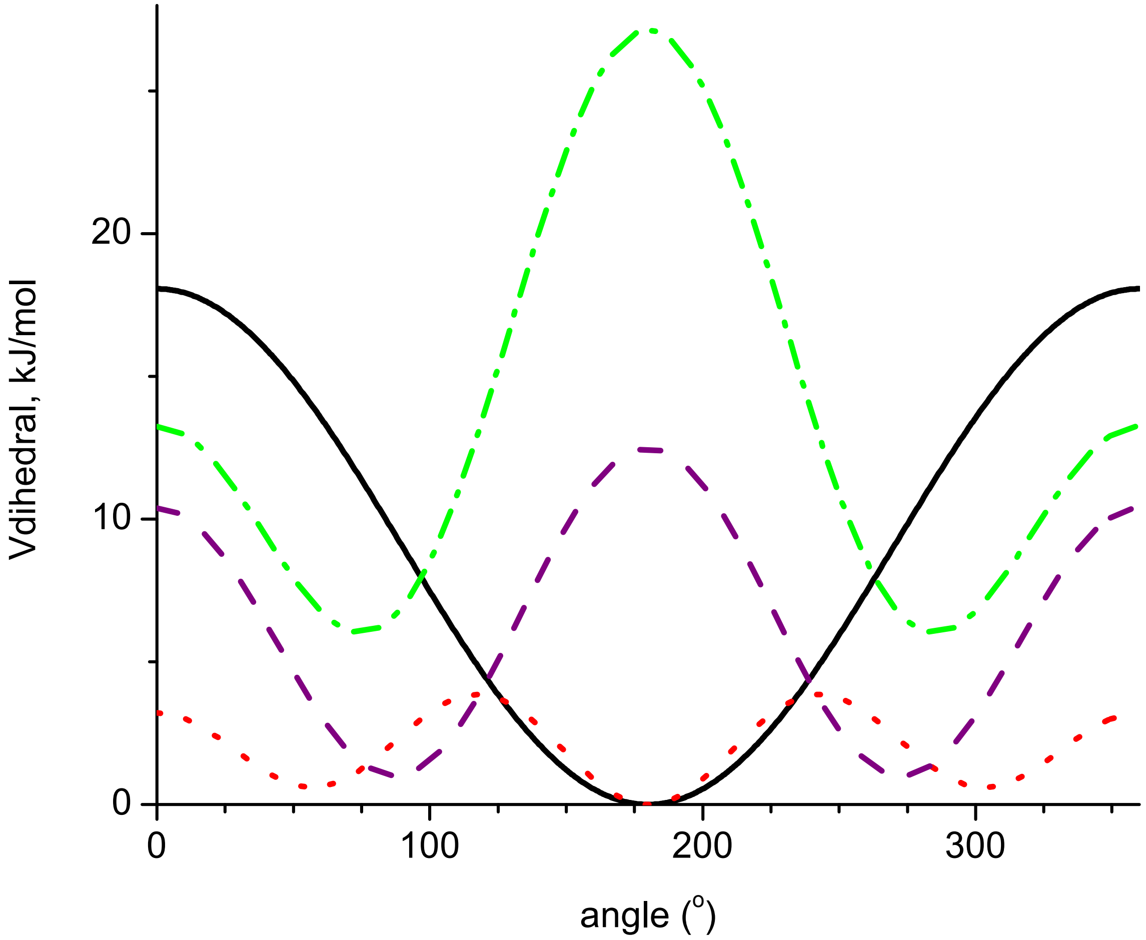

Figure S1: (HO)-C-C-O Dihedral potential according to the parameters of several forcefields as tabulated below: OPLS (black solid line, Ryckaert-Bellemans function with C0 and C1 = 9.035 and -9.035, respectively), AMBER (red dotted line), GROMOS (green dashed-dotted line), and CHARMM (purple dashed line).

Dihedral potential function parameters

Force field	k	phi_0	n
AMBER	0.4184	180	2
	1.60247	0	3
GROMOS	6.942	180	1
	3.312	0	2
	3.312	0	2
CHARMM	1.046	180	1
	5.18816	0	2

4 Supporting Information for MD simulations of PEG200

4.1 Using OPLS forcefield

4.1.1 Topology files

Note: to topology file includes also includes water models, which were not used in this study but are planned for future studies.

“top_mixtures.top”

```
#include "oplsaa.ff/forcefield.itp"
#include "/home/mmh/BrockportMDFiles/mixture_start_files/PolyEGs.itp"
#include
"/home/mmh/BrockportMDFiles/mixture_start_files/atomtypes_tip4p2005water.itp"
#include "/home/mmh/BrockportMDFiles/mixture_start_files/MEG.itp"
#include "/home/mmh/BrockportMDFiles/mixture_start_files/DEG.itp"
#include "/home/mmh/BrockportMDFiles/mixture_start_files/REG.itp"
#include "/home/mmh/BrockportMDFiles/mixture_start_files/TEG.itp"
#include "/home/mmh/BrockportMDFiles/mixture_start_files/PEG.itp"
#include "/home/mmh/BrockportMDFiles/mixture_start_files/XEG.itp"
#include "/home/mmh/BrockportMDFiles/mixture_start_files/HEG.itp"
#include "/home/mmh/BrockportMDFiles/mixture_start_files/OEG.itp"
#include "/home/mmh/BrockportMDFiles/mixture_start_files/NEG.itp"
#include "/home/mmh/BrockportMDFiles/mixture_start_files/tip4p2005.itp"
#include "/home/mmh/BrockportMDFiles/mixture_start_files/spce.itp"



[ system ]
mixtures

[ molecules ]
MEG MEGNMOL
DEG DEGNMOL
REG REGNMOL
TEG TEGNMOL
PEG PEGNMOL
XEG XEGNMOL
HEG HEGNMOL
OEG OEGNMOL
NEG NEGNMOL
TIP4P WATERNMOL
SPCE WATERNMOL
```

“PolyEG.itp”

```
; Polyethylene glycol nonbonded atomtypes
;Monoethylene Glycol : MEG
;Diethylene Glycol : DEG
;Triethylene Glycol : REG
;Tetraethylene Glycol : TEG
```

```
;Pentaethylene Glycol : PEG
;Hexaethylene Glycol : XEG
;Heptaethylene Glycol: HEG
;Octaethylene Glycol :OEG
;Nonaethylene Glycol :NEG

[ atomtypes ]
  peg_HA     H807     1.0080     0.000    A    0.00000E+00   0.00000E+00
  peg_OA     O800    15.9990     0.000    A    3.12000E-01   7.11280E-01
  peg_HC     H808     1.0080     0.000    A    2.50000E-01   1.25520E-01
  peg_C      C801    12.0110     0.000    A    3.50000E-01   2.76144E-01
  peg_OE     O803    15.9990     0.000    A    2.90000E-01   5.85760E-01

Diethylene glycol
;
; GENERATED BY LigParGen Server
; Jorgensen Lab @ Yale University
;

[ moleculetype ]
; Name               nrexcl
DEG                   3
[ atoms ]
;   nr       type  resnr residue  atom   cgnr     charge       mass
     1   peg_OA         1    DEG  OAD1      1    -0.6885    15.9990
     2   peg_C          1    DEG  CD1       1     0.1082    12.0110
     3   peg_C          1    DEG  CD2       1     0.0069    12.0110
     4   peg_OE         1    DEG  OED1      1    -0.4007    15.9990
     5   peg_C          1    DEG  CD3       1    -0.0411    12.0110
     6   peg_C          1    DEG  CD4       1     0.1078    12.0110
     7   peg_OA         1    DEG  OAD2      1    -0.6862    15.9990
     8   peg_HA         1    DEG  HAD1      1     0.4182     1.0080
     9   peg_HC         1    DEG  HCD1      1      0.082     1.0080
    10   peg_HC         1    DEG  HCD2      1      0.082     1.0080
    11   peg_HC         1    DEG  HCD3      1     0.0927     1.0080
    12   peg_HC         1    DEG  HCD4      1     0.0927     1.0080
    13   peg_HC         1    DEG  HCD5      1     0.0947     1.0080
    14   peg_HC         1    DEG  HCD6      1     0.0947     1.0080
    15   peg_HC         1    DEG  HCD7      1     0.1066     1.0080
    16   peg_HC         1    DEG  HCD8      1     0.1066     1.0080
    17   peg_HA         1    DEG  HAD2      1     0.4234     1.0080
[ bonds ]
    2     1     1      0.1410 267776.000
    3     2     1      0.1529 224262.400
    4     3     1      0.1410 267776.000
    5     4     1      0.1410 267776.000
    6     5     1      0.1529 224262.400
    7     6     1      0.1410 267776.000
    8     1     1      0.0945 462750.400
    9     2     1      0.1090 284512.000
   10     2     1      0.1090 284512.000
   11     3     1      0.1090 284512.000
   12     3     1      0.1090 284512.000
   13     5     1      0.1090 284512.000
   14     5     1      0.1090 284512.000
   15     6     1      0.1090 284512.000
   16     6     1      0.1090 284512.000
```

```
   17     7     1      0.0945 462750.400

[ angles ]
;  ai    aj    ak funct            c0            c1            c2            c3
    1     2     3     1    109.500    418.400
    2     3     4     1    109.500    418.400
    3     4     5     1    109.500    502.080
    4     5     6     1    109.500    418.400
    5     6     7     1    109.500    418.400
    2     1     8     1    108.500    460.240
    1     2     9     1    109.500    292.880
    1     2    10     1    109.500    292.880
    2     3    11     1    110.700    313.800
    2     3    12     1    110.700    313.800
    4     5    13     1    109.500    292.880
    4     5    14     1    109.500    292.880
    5     6    15     1    110.700    313.800
    5     6    16     1    110.700    313.800
    6     7    17     1    108.500    460.240
    4     3    11     1    109.500    292.880
    9     2    10     1    107.800    276.144
   15     6    16     1    107.800    276.144
   13     5    14     1    107.800    276.144
    6     5    14     1    110.700    313.800
   11     3    12     1    107.800    276.144
    3     2     9     1    110.700    313.800
    6     5    13     1    110.700    313.800
    7     6    15     1    109.500    292.880
    7     6    16     1    109.500    292.880
    4     3    12     1    109.500    292.880
    3     2    10     1    110.700    313.800

[ dihedrals ]
; IMPROPER DIHEDRAL ANGLES
;  ai    aj    ak    al funct            c0            c1            c2            c3
c4            c5

[ dihedrals ]
; PROPER DIHEDRAL ANGLES
;  ai    aj    ak    al funct            c0            c1            c2            c3
c4            c5
    6    5    4    3        3       1.715   2.845   1.046  -5.607  -0.000   0.000
    5    4    3    2        3       1.715   2.845   1.046  -5.607  -0.000   0.000
   12    3    2    9        3       0.628   1.883   0.000  -2.510  -0.000   0.000
   16    6    5   13        3       0.628   1.883   0.000  -2.510  -0.000   0.000
   12    3    2   10        3       0.628   1.883   0.000  -2.510  -0.000   0.000
   16    6    5   14        3       0.628   1.883   0.000  -2.510  -0.000   0.000
   11    3    2   10        3       0.628   1.883   0.000  -2.510  -0.000   0.000
   15    6    5   13        3       0.628   1.883   0.000  -2.510  -0.000   0.000
   11    3    2    9        3       0.628   1.883   0.000  -2.510  -0.000   0.000
   15    6    5   14        3       0.628   1.883   0.000  -2.510  -0.000   0.000
   14    5    6    7        3       0.979   2.937   0.000  -3.916  -0.000   0.000
   12    3    2    1        3       0.979   2.937   0.000  -3.916  -0.000   0.000
   13    5    6    7        3       0.979   2.937   0.000  -3.916  -0.000   0.000
   11    3    2    1        3       0.979   2.937   0.000  -3.916  -0.000   0.000
   15    6    5    4        3       0.979   2.937   0.000  -3.916  -0.000   0.000
   16    6    5    4        3       0.979   2.937   0.000  -3.916  -0.000   0.000
```

```
   10    2    3    4        3       0.979   2.937   0.000  -3.916  -0.000   0.000
    9    2    3    4        3       0.979   2.937   0.000  -3.916  -0.000   0.000
    9    2    1    8        3       0.736   2.209   0.000  -2.946  -0.000   0.000
   10    2    1    8        3       0.736   2.209   0.000  -2.946  -0.000   0.000
   13    5    4    3        3       1.590   4.770   0.000  -6.360  -0.000   0.000
   11    3    4    5        3       1.590   4.770   0.000  -6.360  -0.000   0.000
   14    5    4    3        3       1.590   4.770   0.000  -6.360  -0.000   0.000
   12    3    4    5        3       1.590   4.770   0.000  -6.360  -0.000   0.000
   17    7    6    5        3      -0.444   3.833   0.728  -4.117  -0.000   0.000
    8    1    2    3        3      -0.444   3.833   0.728  -4.117  -0.000   0.000
   17    7    6   15        3       0.736   2.209   0.000  -2.946  -0.000   0.000
   17    7    6   16        3       0.736   2.209   0.000  -2.946  -0.000   0.000
    7    6    5    4        3       9.035  -9.035   0.000  -0.000  -0.000   0.000
    4    3    2    1        3       9.035  -9.035   0.000  -0.000  -0.000   0.000

[ pairs ]
     1     4    1
     2     5    1
     3     6    1
     4     7    1
     3     8    1
     1    11    1
     4     9    1
     1    12    1
     4    10    1
     5    11    1
     3    13    1
     8     9    1
     5    12    1
     3    14    1
     8    10    1
     4    15    1
     9    11    1
     7    13    1
     4    16    1
    10    11    1
     9    12    1
     7    14    1
    10    12    1
     5    17    1
    13    15    1
    14    15    1
    13    16    1
    14    16    1
    15    17    1
    16    17    1
```

Triethylene glycol

```
;
; GENERATED BY LigParGen Server
; Jorgensen Lab @ Yale University
;
[ moleculetype ]
; Name               nrexcl
REG                   3
```

```
[ atoms ]
;   nr       type  resnr residue  atom   cgnr     charge       mass
     1   peg_OA        1    REG   OAR1      1    -0.6904    15.9990
     2   peg_C         1    REG   CR1       1     0.1087    12.0110
     3   peg_C         1    REG   CR2       1     0.0049    12.0110
     4   peg_OE        1    REG   OER1      1    -0.3783    15.9990
     5   peg_C         1    REG   CR3       1     0.0099    12.0110
     6   peg_C         1    REG   CR4       1     0.0105    12.0110
     7   peg_OE        1    REG   OER2      1    -0.3919    15.9990
     8   peg_C         1    REG   CR5       1    -0.0445    12.0110
     9   peg_C         1    REG   CR6       1     0.1087    12.0110
    10   peg_OA        1    REG   OAR2      1     -0.687    15.9990
    11   peg_HA        1    REG   HAR1      1     0.4169     1.0080
    12   peg_HC        1    REG   HCR1      1     0.0829     1.0080
    13   peg_HC        1    REG   HCR2      1     0.0829     1.0080
    14   peg_HC        1    REG   HCR3      1     0.0892     1.0080
    15   peg_HC        1    REG   HCR4      1     0.0892     1.0080
    16   peg_HC        1    REG   HCR5      1     0.0928     1.0080
    17   peg_HC        1    REG   HCR6      1     0.0928     1.0080
    18   peg_HC        1    REG   HCR7      1     0.0924     1.0080
    19   peg_HC        1    REG   HCR8      1     0.0924     1.0080
    20   peg_HC        1    REG   HCR9      1     0.0906     1.0080
    21   peg_HC        1    REG   HCRA      1     0.0906     1.0080
    22   peg_HC        1    REG   HCRB      1     0.1057     1.0080
    23   peg_HC        1    REG   HCRC      1     0.1057     1.0080
    24   peg_HA        1    REG   HAR2      1     0.4253     1.0080
[ bonds ]
    2     1     1      0.1410 267776.000
    3     2     1      0.1529 224262.400
    4     3     1      0.1410 267776.000
    5     4     1      0.1410 267776.000
    6     5     1      0.1529 224262.400
    7     6     1      0.1410 267776.000
    8     7     1      0.1410 267776.000
    9     8     1      0.1529 224262.400
   10     9     1      0.1410 267776.000
   11     1     1      0.0945 462750.400
   12     2     1      0.1090 284512.000
   13     2     1      0.1090 284512.000
   14     3     1      0.1090 284512.000
   15     3     1      0.1090 284512.000
   16     5     1      0.1090 284512.000
   17     5     1      0.1090 284512.000
   18     6     1      0.1090 284512.000
   19     6     1      0.1090 284512.000
   20     8     1      0.1090 284512.000
   21     8     1      0.1090 284512.000
   22     9     1      0.1090 284512.000
   23     9     1      0.1090 284512.000
   24    10     1      0.0945 462750.400

[ angles ]
;  ai    aj    ak funct            c0            c1            c2            c3
    1     2     3     1    109.500    418.400
    2     3     4     1    109.500    418.400
    3     4     5     1    109.500    502.080
    4     5     6     1    109.500    418.400
```

```
    5     6     7     1    109.500    418.400
    6     7     8     1    109.500    502.080
    7     8     9     1    109.500    418.400
    8     9    10     1    109.500    418.400
    2     1    11     1    108.500    460.240
    1     2    12     1    109.500    292.880
    1     2    13     1    109.500    292.880
    2     3    14     1    110.700    313.800
    2     3    15     1    110.700    313.800
    4     5    16     1    109.500    292.880
    4     5    17     1    109.500    292.880
    5     6    18     1    110.700    313.800
    5     6    19     1    110.700    313.800
    7     8    20     1    109.500    292.880
    7     8    21     1    109.500    292.880
    8     9    22     1    110.700    313.800
    8     9    23     1    110.700    313.800
    9    10    24     1    108.500    460.240
    7     6    18     1    109.500    292.880
   12     2    13     1    107.800    276.144
   18     6    19     1    107.800    276.144
    9     8    20     1    110.700    313.800
   16     5    17     1    107.800    276.144
    6     5    17     1    110.700    313.800
   10     9    23     1    109.500    292.880
   22     9    23     1    107.800    276.144
    7     6    19     1    109.500    292.880
   14     3    15     1    107.800    276.144
   10     9    22     1    109.500    292.880
    3     2    12     1    110.700    313.800
   20     8    21     1    107.800    276.144
    6     5    16     1    110.700    313.800
    3     2    13     1    110.700    313.800
    4     3    14     1    109.500    292.880
    9     8    21     1    110.700    313.800
    4     3    15     1    109.500    292.880

[ dihedrals ]
; IMPROPER DIHEDRAL ANGLES
;  ai    aj    ak    al funct            c0            c1            c2            c3
c4            c5

[ dihedrals ]
; PROPER DIHEDRAL ANGLES
;  ai    aj    ak    al funct            c0            c1            c2            c3
c4            c5
    6    5    4    3        3       1.715   2.845   1.046  -5.607  -0.000   0.000
    9    8    7    6        3       1.715   2.845   1.046  -5.607  -0.000   0.000
    5    4    3    2        3       1.715   2.845   1.046  -5.607  -0.000   0.000
    8    7    6    5        3       1.715   2.845   1.046  -5.607  -0.000   0.000
   14    3    2   12        3       0.628   1.883   0.000  -2.510  -0.000   0.000
   23    9    8   20        3       0.628   1.883   0.000  -2.510  -0.000   0.000
   22    9    8   21        3       0.628   1.883   0.000  -2.510  -0.000   0.000
   18    6    5   16        3       0.628   1.883   0.000  -2.510  -0.000   0.000
   15    3    2   12        3       0.628   1.883   0.000  -2.510  -0.000   0.000
   14    3    2   13        3       0.628   1.883   0.000  -2.510  -0.000   0.000
   23    9    8   21        3       0.628   1.883   0.000  -2.510  -0.000   0.000
```

```
   22    9    8   20         3       0.628   1.883   0.000  -2.510  -0.000   0.000
   19    6    5   17         3       0.628   1.883   0.000  -2.510  -0.000   0.000
   18    6    5   17         3       0.628   1.883   0.000  -2.510  -0.000   0.000
   15    3    2   13         3       0.628   1.883   0.000  -2.510  -0.000   0.000
   19    6    5   16         3       0.628   1.883   0.000  -2.510  -0.000   0.000
   21    8    9   10         3       0.979   2.937   0.000  -3.916  -0.000   0.000
   14    3    2    1         3       0.979   2.937   0.000  -3.916  -0.000   0.000
   20    8    9   10         3       0.979   2.937   0.000  -3.916  -0.000   0.000
   15    3    2    1         3       0.979   2.937   0.000  -3.916  -0.000   0.000
   23    9    8    7         3       0.979   2.937   0.000  -3.916  -0.000   0.000
   18    6    5    4         3       0.979   2.937   0.000  -3.916  -0.000   0.000
   13    2    3    4         3       0.979   2.937   0.000  -3.916  -0.000   0.000
   19    6    5    4         3       0.979   2.937   0.000  -3.916  -0.000   0.000
   16    5    6    7         3       0.979   2.937   0.000  -3.916  -0.000   0.000
   17    5    6    7         3       0.979   2.937   0.000  -3.916  -0.000   0.000
   22    9    8    7         3       0.979   2.937   0.000  -3.916  -0.000   0.000
   12    2    3    4         3       0.979   2.937   0.000  -3.916  -0.000   0.000
   12    2    1   11         3       0.736   2.209   0.000  -2.946  -0.000   0.000
   13    2    1   11         3       0.736   2.209   0.000  -2.946  -0.000   0.000
   19    6    7    8         3       1.590   4.770   0.000  -6.360  -0.000   0.000
   20    8    7    6         3       1.590   4.770   0.000  -6.360  -0.000   0.000
   18    6    7    8         3       1.590   4.770   0.000  -6.360  -0.000   0.000
   16    5    4    3         3       1.590   4.770   0.000  -6.360  -0.000   0.000
   17    5    4    3         3       1.590   4.770   0.000  -6.360  -0.000   0.000
   14    3    4    5         3       1.590   4.770   0.000  -6.360  -0.000   0.000
   15    3    4    5         3       1.590   4.770   0.000  -6.360  -0.000   0.000
   21    8    7    6         3       1.590   4.770   0.000  -6.360  -0.000   0.000
   24   10    9    8         3      -0.444   3.833   0.728  -4.117  -0.000   0.000
   11    1    2    3         3      -0.444   3.833   0.728  -4.117  -0.000   0.000
   24   10    9   22         3       0.736   2.209   0.000  -2.946  -0.000   0.000
   24   10    9   23         3       0.736   2.209   0.000  -2.946  -0.000   0.000
   10    9    8    7         3       9.035  -9.035   0.000  -0.000  -0.000   0.000
    4    3    2    1         3       9.035  -9.035   0.000  -0.000  -0.000   0.000
    7    6    5    4         3      -1.151   1.151   0.000  -0.000  -0.000   0.000

[ pairs ]
     1     4    1
     2     5    1
     3     6    1
     4     7    1
     5     8    1
     3    11    1
     6     9    1
     1    14    1
     4    12    1
     1    15    1
     7    10    1
     4    13    1
     5    14    1
     3    16    1
     5    15    1
     3    17    1
     4    18    1
    11    12    1
     7    16    1
     4    19    1
    11    13    1
```

```
    7    17    1
   12    14    1
    8    18    1
    6    20    1
   13    14    1
   12    15    1
    8    19    1
    6    21    1
   13    15    1
    7    22    1
   10    20    1
    7    23    1
   10    21    1
    8    24    1
   16    18    1
   17    18    1
   16    19    1
   17    19    1
   20    22    1
   21    22    1
   20    23    1
   21    23    1
   22    24    1
   23    24    1
```

Tetraethyene glycol

```
;
; GENERATED BY LigParGen Server
; Jorgensen Lab @ Yale University
;
[ moleculetype ]
; Name               nrexcl
TEG                    3
[ atoms ]
;   nr       type  resnr residue  atom   cgnr     charge       mass
     1   peg_OA        1    TEG   OAT1      1    -0.6887    15.9990
     2   peg_C         1    TEG   CT1       1     0.1087    12.0110
     3   peg_C         1    TEG   CT2       1     0.0083    12.0110
     4   peg_OE        1    TEG   OET1      1    -0.3851    15.9990
     5   peg_C         1    TEG   CT3       1     0.0083    12.0110
     6   peg_C         1    TEG   CT4       1     0.0088    12.0110
     7   peg_OE        1    TEG   OET2      1    -0.3849    15.9990
     8   peg_C         1    TEG   CT5       1     0.0089    12.0110
     9   peg_C         1    TEG   CT6       1     0.0068    12.0110
    10   peg_OE        1    TEG   OET3      1    -0.3989    15.9990
    11   peg_C         1    TEG   CT7       1    -0.0408    12.0110
    12   peg_C         1    TEG   CT8       1     0.1081    12.0110
    13   peg_OA        1    TEG   OAT2      1    -0.6858    15.9990
    14   peg_HA        1    TEG   HAT1      1     0.4177     1.0080
    15   peg_HC        1    TEG   HCT1      1     0.0822     1.0080
    16   peg_HC        1    TEG   HCT2      1     0.0822     1.0080
    17   peg_HC        1    TEG   HCT3      1     0.0925     1.0080
    18   peg_HC        1    TEG   HCT4      1     0.0925     1.0080
    19   peg_HC        1    TEG   HCT5      1     0.0916     1.0080
    20   peg_HC        1    TEG   HCT6      1     0.0916     1.0080
```

```
    21   peg_HC        1    TEG   HCT7      1     0.0917     1.0080
    22   peg_HC        1    TEG   HCT8      1     0.0917     1.0080
    23   peg_HC        1    TEG   HCT9      1     0.0913     1.0080
    24   peg_HC        1    TEG   HCTA      1     0.0913     1.0080
    25   peg_HC        1    TEG   HCTB      1     0.0919     1.0080
    26   peg_HC        1    TEG   HCTC      1     0.0919     1.0080
    27   peg_HC        1    TEG   HCTD      1     0.0946     1.0080
    28   peg_HC        1    TEG   HCTE      1     0.0946     1.0080
    29   peg_HC        1    TEG   HCTF      1     0.1068     1.0080
    30   peg_HC        1    TEG   HCTG      1     0.1068     1.0080
    31   peg_HA        1    TEG   HAT2      1     0.4234     1.0080
[ bonds ]
    2     1     1      0.1410 267776.000
    3     2     1      0.1529 224262.400
    4     3     1      0.1410 267776.000
    5     4     1      0.1410 267776.000
    6     5     1      0.1529 224262.400
    7     6     1      0.1410 267776.000
    8     7     1      0.1410 267776.000
    9     8     1      0.1529 224262.400
   10     9     1      0.1410 267776.000
   11    10     1      0.1410 267776.000
   12    11     1      0.1529 224262.400
   13    12     1      0.1410 267776.000
   14     1     1      0.0945 462750.400
   15     2     1      0.1090 284512.000
   16     2     1      0.1090 284512.000
   17     3     1      0.1090 284512.000
   18     3     1      0.1090 284512.000
   19     5     1      0.1090 284512.000
   20     5     1      0.1090 284512.000
   21     6     1      0.1090 284512.000
   22     6     1      0.1090 284512.000
   23     8     1      0.1090 284512.000
   24     8     1      0.1090 284512.000
   25     9     1      0.1090 284512.000
   26     9     1      0.1090 284512.000
   27    11     1      0.1090 284512.000
   28    11     1      0.1090 284512.000
   29    12     1      0.1090 284512.000
   30    12     1      0.1090 284512.000
   31    13     1      0.0945 462750.400

[ angles ]
;  ai    aj    ak funct            c0            c1            c2            c3
    1     2     3     1    109.500    418.400
    2     3     4     1    109.500    418.400
    3     4     5     1    109.500    502.080
    4     5     6     1    109.500    418.400
    5     6     7     1    109.500    418.400
    6     7     8     1    109.500    502.080
    7     8     9     1    109.500    418.400
    8     9    10     1    109.500    418.400
    9    10    11     1    109.500    502.080
   10    11    12     1    109.500    418.400
   11    12    13     1    109.500    418.400
    2     1    14     1    108.500    460.240
```

```
     1     2    15     1    109.500    292.880
     1     2    16     1    109.500    292.880
     2     3    17     1    110.700    313.800
     2     3    18     1    110.700    313.800
     4     5    19     1    109.500    292.880
     4     5    20     1    109.500    292.880
     5     6    21     1    110.700    313.800
     5     6    22     1    110.700    313.800
     7     8    23     1    109.500    292.880
     7     8    24     1    109.500    292.880
     8     9    25     1    110.700    313.800
     8     9    26     1    110.700    313.800
    10    11    27     1    109.500    292.880
    10    11    28     1    109.500    292.880
    11    12    29     1    110.700    313.800
    11    12    30     1    110.700    313.800
    12    13    31     1    108.500    460.240
    25     9    26     1    107.800    276.144
    23     8    24     1    107.800    276.144
     3     2    15     1    110.700    313.800
    29    12    30     1    107.800    276.144
    12    11    27     1    110.700    313.800
    15     2    16     1    107.800    276.144
     7     6    22     1    109.500    292.880
    21     6    22     1    107.800    276.144
    27    11    28     1    107.800    276.144
     6     5    19     1    110.700    313.800
     9     8    23     1    110.700    313.800
    13    12    29     1    109.500    292.880
     3     2    16     1    110.700    313.800
     4     3    18     1    109.500    292.880
     9     8    24     1    110.700    313.800
    19     5    20     1    107.800    276.144
    13    12    30     1    109.500    292.880
     6     5    20     1    110.700    313.800
    10     9    25     1    109.500    292.880
    10     9    26     1    109.500    292.880
     4     3    17     1    109.500    292.880
    17     3    18     1    107.800    276.144
    12    11    28     1    110.700    313.800
     7     6    21     1    109.500    292.880

[ dihedrals ]
; IMPROPER DIHEDRAL ANGLES
;  ai    aj    ak    al funct            c0            c1            c2            c3
c4            c5

[ dihedrals ]
; PROPER DIHEDRAL ANGLES
;  ai    aj    ak    al funct            c0            c1            c2            c3
c4            c5
     6    5    4    3        3       1.715   2.845   1.046  -5.607  -0.000   0.000
     9    8    7    6        3       1.715   2.845   1.046  -5.607  -0.000   0.000
    12   11   10    9        3       1.715   2.845   1.046  -5.607  -0.000   0.000
     5    4    3    2        3       1.715   2.845   1.046  -5.607  -0.000   0.000
     8    7    6    5        3       1.715   2.845   1.046  -5.607  -0.000   0.000
    11   10    9    8        3       1.715   2.845   1.046  -5.607  -0.000   0.000
```

18	3	2	15	3	0.628	1.883	0.000	-2.510	-0.000	0.000
18	3	2	16	3	0.628	1.883	0.000	-2.510	-0.000	0.000
21	6	5	20	3	0.628	1.883	0.000	-2.510	-0.000	0.000
21	6	5	19	3	0.628	1.883	0.000	-2.510	-0.000	0.000
22	6	5	19	3	0.628	1.883	0.000	-2.510	-0.000	0.000
29	12	11	28	3	0.628	1.883	0.000	-2.510	-0.000	0.000
30	12	11	28	3	0.628	1.883	0.000	-2.510	-0.000	0.000
17	3	2	16	3	0.628	1.883	0.000	-2.510	-0.000	0.000
22	6	5	20	3	0.628	1.883	0.000	-2.510	-0.000	0.000
30	12	11	27	3	0.628	1.883	0.000	-2.510	-0.000	0.000
17	3	2	15	3	0.628	1.883	0.000	-2.510	-0.000	0.000
26	9	8	24	3	0.628	1.883	0.000	-2.510	-0.000	0.000
26	9	8	23	3	0.628	1.883	0.000	-2.510	-0.000	0.000
29	12	11	27	3	0.628	1.883	0.000	-2.510	-0.000	0.000
25	9	8	23	3	0.628	1.883	0.000	-2.510	-0.000	0.000
25	9	8	24	3	0.628	1.883	0.000	-2.510	-0.000	0.000
28	11	12	13	3	0.979	2.937	0.000	-3.916	-0.000	0.000
18	3	2	1	3	0.979	2.937	0.000	-3.916	-0.000	0.000
27	11	12	13	3	0.979	2.937	0.000	-3.916	-0.000	0.000
17	3	2	1	3	0.979	2.937	0.000	-3.916	-0.000	0.000
19	5	6	7	3	0.979	2.937	0.000	-3.916	-0.000	0.000
20	5	6	7	3	0.979	2.937	0.000	-3.916	-0.000	0.000
30	12	11	10	3	0.979	2.937	0.000	-3.916	-0.000	0.000
26	9	8	7	3	0.979	2.937	0.000	-3.916	-0.000	0.000
24	8	9	10	3	0.979	2.937	0.000	-3.916	-0.000	0.000
22	6	5	4	3	0.979	2.937	0.000	-3.916	-0.000	0.000
23	8	9	10	3	0.979	2.937	0.000	-3.916	-0.000	0.000
29	12	11	10	3	0.979	2.937	0.000	-3.916	-0.000	0.000
21	6	5	4	3	0.979	2.937	0.000	-3.916	-0.000	0.000
25	9	8	7	3	0.979	2.937	0.000	-3.916	-0.000	0.000
15	2	3	4	3	0.979	2.937	0.000	-3.916	-0.000	0.000
16	2	3	4	3	0.979	2.937	0.000	-3.916	-0.000	0.000
16	2	1	14	3	0.736	2.209	0.000	-2.946	-0.000	0.000
15	2	1	14	3	0.736	2.209	0.000	-2.946	-0.000	0.000
21	6	7	8	3	1.590	4.770	0.000	-6.360	-0.000	0.000
24	8	7	6	3	1.590	4.770	0.000	-6.360	-0.000	0.000
22	6	7	8	3	1.590	4.770	0.000	-6.360	-0.000	0.000
28	11	10	9	3	1.590	4.770	0.000	-6.360	-0.000	0.000
26	9	10	11	3	1.590	4.770	0.000	-6.360	-0.000	0.000
19	5	4	3	3	1.590	4.770	0.000	-6.360	-0.000	0.000
25	9	10	11	3	1.590	4.770	0.000	-6.360	-0.000	0.000
17	3	4	5	3	1.590	4.770	0.000	-6.360	-0.000	0.000
20	5	4	3	3	1.590	4.770	0.000	-6.360	-0.000	0.000
23	8	7	6	3	1.590	4.770	0.000	-6.360	-0.000	0.000
18	3	4	5	3	1.590	4.770	0.000	-6.360	-0.000	0.000
27	11	10	9	3	1.590	4.770	0.000	-6.360	-0.000	0.000
31	13	12	11	3	-0.444	3.833	0.728	-4.117	-0.000	0.000
14	1	2	3	3	-0.444	3.833	0.728	-4.117	-0.000	0.000
31	13	12	30	3	0.736	2.209	0.000	-2.946	-0.000	0.000
31	13	12	29	3	0.736	2.209	0.000	-2.946	-0.000	0.000
13	12	11	10	3	9.035	-9.035	0.000	-0.000	-0.000	0.000
4	3	2	1	3	9.035	-9.035	0.000	-0.000	-0.000	0.000
7	6	5	4	3	-1.151	1.151	0.000	-0.000	-0.000	0.000
10	9	8	7	3	-1.151	1.151	0.000	-0.000	-0.000	0.000

[pairs]

```
    1     4    1
```

2	5	1
3	6	1
4	7	1
5	8	1
6	9	1
7	10	1
3	14	1
1	17	1
8	11	1
4	15	1
1	18	1
4	16	1
9	12	1
5	17	1
3	19	1
10	13	1
5	18	1
3	20	1
4	21	1
7	19	1
4	22	1
7	20	1
14	15	1
8	21	1
6	23	1
14	16	1
8	22	1
6	24	1
15	17	1
7	25	1
16	17	1
15	18	1
10	23	1
7	26	1
16	18	1
10	24	1
11	25	1
9	27	1
11	26	1
9	28	1
10	29	1
19	21	1
13	27	1
10	30	1
20	21	1
19	22	1
13	28	1
20	22	1
11	31	1
23	25	1
24	25	1
23	26	1
24	26	1
27	29	1
28	29	1
27	30	1
28	30	1

```
    29    31    1
    30    31    1
```

Pentaethylene glycol

```
LIGPARGEN GENERATED GRO FILE
   38
    1PEG   OAP1    1   0.100   0.100   0.000
    1PEG   CP1     2  -0.042   0.100   0.000
    1PEG   CP2     3  -0.095   0.100   0.143
    1PEG   OEP1    4  -0.238   0.100   0.141
    1PEG   CP3     5  -0.291   0.100   0.273
    1PEG   CP4     6  -0.443   0.099   0.266
    1PEG   OEP2    7  -0.496   0.093   0.399
    1PEG   CP5     8  -0.639   0.103   0.400
    1PEG   CP6     9  -0.688   0.102   0.544
    1PEG   OEP3   10  -0.649   0.224   0.609
    1PEG   CP7    11  -0.687   0.226   0.746
    1PEG   CP8    12  -0.631   0.352   0.811
    1PEG   OEP4   13  -0.673   0.359   0.947
    1PEG   CP9    14  -0.622   0.476   1.011
    1PEG   CPA    15  -0.671   0.478   1.156
    1PEG   OAP2   16  -0.623   0.362   1.224
    1PEG   HAP1   17   0.128   0.100  -0.093
    1PEG   HCP1   18  -0.078   0.012  -0.054
    1PEG   HCP2   19  -0.078   0.189  -0.054
    1PEG   HCP3   20  -0.058   0.189   0.196
    1PEG   HCP4   21  -0.058   0.011   0.196
    1PEG   HCP5   22  -0.257   0.189   0.328
    1PEG   HCP6   23  -0.256   0.011   0.328
    1PEG   HCP7   24  -0.478   0.013   0.208
    1PEG   HCP8   25  -0.478   0.190   0.216
    1PEG   HCP9   26  -0.680   0.017   0.346
    1PEG   HCPA   27  -0.669   0.196   0.350
    1PEG   HCPB   28  -0.645   0.017   0.598
    1PEG   HCPC   29  -0.798   0.095   0.546
    1PEG   HCPD   30  -0.649   0.137   0.798
    1PEG   HCPE   31  -0.796   0.225   0.754
    1PEG   HCPF   32  -0.666   0.440   0.757
    1PEG   HCPG   33  -0.521   0.350   0.806
    1PEG   HCPH   34  -0.657   0.566   0.959
    1PEG   HCPI   35  -0.512   0.472   1.009
    1PEG   HCPJ   36  -0.780   0.476   1.160
    1PEG   HCPK   37  -0.634   0.567   1.208
    1PEG   HAP2   38  -0.645   0.287   1.166
   1.00000   1.00000   1.00000
```

Hexathylene glycol

```
;
; GENERATED BY LigParGen Server
; Jorgensen Lab @ Yale University
;
[ moleculetype ]
; Name               nrexcl
XEG                   3
```

```
[ atoms ]
;   nr       type  resnr residue  atom   cgnr     charge       mass
     1   peg_OA        1    XEG   OAX1      1    -0.6884    15.9990
     2   peg_C         1    XEG   CX1       1     0.1086    12.0110
     3   peg_C         1    XEG   CX2       1     0.0082    12.0110
     4   peg_OE        1    XEG   OEX1      1    -0.3849    15.9990
     5   peg_C         1    XEG   CX3       1     0.0078    12.0110
     6   peg_C         1    XEG   CX4       1     0.0085    12.0110
     7   peg_OE        1    XEG   OEX2      1     -0.384    15.9990
     8   peg_C         1    XEG   CX5       1     0.0086    12.0110
     9   peg_C         1    XEG   CX6       1     0.0084    12.0110
    10   peg_OE        1    XEG   OEX3      1     -0.384    15.9990
    11   peg_C         1    XEG   CX7       1     0.0087    12.0110
    12   peg_C         1    XEG   CX8       1     0.0082    12.0110
    13   peg_OE        1    XEG   OEX4      1     -0.384    15.9990
    14   peg_C         1    XEG   CX9       1     0.0084    12.0110
    15   peg_C         1    XEG   CXA       1     0.0067    12.0110
    16   peg_OE        1    XEG   OEX5      1    -0.3986    15.9990
    17   peg_C         1    XEG   CXB       1    -0.0411    12.0110
    18   peg_C         1    XEG   CXC       1     0.1084    12.0110
    19   peg_OA        1    XEG   OAX2      1    -0.6855    15.9990
    20   peg_HA        1    XEG   HAX1      1     0.4175     1.0080
    21   peg_HC        1    XEG   HCX1      1     0.0821     1.0080
    22   peg_HC        1    XEG   HCX2      1     0.0821     1.0080
    23   peg_HC        1    XEG   HCX3      1     0.0925     1.0080
    24   peg_HC        1    XEG   HCX4      1     0.0925     1.0080
    25   peg_HC        1    XEG   HCX5      1     0.0917     1.0080
    26   peg_HC        1    XEG   HCX6      1     0.0917     1.0080
    27   peg_HC        1    XEG   HCX7      1     0.0916     1.0080
    28   peg_HC        1    XEG   HCX8      1     0.0916     1.0080
    29   peg_HC        1    XEG   HCX9      1     0.0916     1.0080
    30   peg_HC        1    XEG   HCXA      1     0.0916     1.0080
    31   peg_HC        1    XEG   HCXB      1     0.0918     1.0080
    32   peg_HC        1    XEG   HCXC      1     0.0918     1.0080
    33   peg_HC        1    XEG   HCXD      2     0.0917     1.0080
    34   peg_HC        1    XEG   HCXE      2     0.0917     1.0080
    35   peg_HC        1    XEG   HCXF      2      0.092     1.0080
    36   peg_HC        1    XEG   HCXG      2      0.092     1.0080
    37   peg_HC        1    XEG   HCXH      2     0.0914     1.0080
    38   peg_HC        1    XEG   HCXI      2     0.0914     1.0080
    39   peg_HC        1    XEG   HCXJ      2     0.0919     1.0080
    40   peg_HC        1    XEG   HCXK      2     0.0919     1.0080
    41   peg_HC        1    XEG   HCXL      2     0.0945     1.0080
    42   peg_HC        1    XEG   HCXM      2     0.0945     1.0080
    43   peg_HC        1    XEG   HCXN      2     0.1067     1.0080
    44   peg_HC        1    XEG   HCXO      2     0.1067     1.0080
    45   peg_HA        1    XEG   HAX2      2     0.4235     1.0080
[ bonds ]
    2      1     1      0.1410 267776.000
    3      2     1      0.1529 224262.400
    4      3     1      0.1410 267776.000
    5      4     1      0.1410 267776.000
    6      5     1      0.1529 224262.400
    7      6     1      0.1410 267776.000
    8      7     1      0.1410 267776.000
    9      8     1      0.1529 224262.400
   10      9     1      0.1410 267776.000
```

```
   11    10     1      0.1410 267776.000
   12    11     1      0.1529 224262.400
   13    12     1      0.1410 267776.000
   14    13     1      0.1410 267776.000
   15    14     1      0.1529 224262.400
   16    15     1      0.1410 267776.000
   17    16     1      0.1410 267776.000
   18    17     1      0.1529 224262.400
   19    18     1      0.1410 267776.000
   20     1     1      0.0945 462750.400
   21     2     1      0.1090 284512.000
   22     2     1      0.1090 284512.000
   23     3     1      0.1090 284512.000
   24     3     1      0.1090 284512.000
   25     5     1      0.1090 284512.000
   26     5     1      0.1090 284512.000
   27     6     1      0.1090 284512.000
   28     6     1      0.1090 284512.000
   29     8     1      0.1090 284512.000
   30     8     1      0.1090 284512.000
   31     9     1      0.1090 284512.000
   32     9     1      0.1090 284512.000
   33    11     1      0.1090 284512.000
   34    11     1      0.1090 284512.000
   35    12     1      0.1090 284512.000
   36    12     1      0.1090 284512.000
   37    14     1      0.1090 284512.000
   38    14     1      0.1090 284512.000
   39    15     1      0.1090 284512.000
   40    15     1      0.1090 284512.000
   41    17     1      0.1090 284512.000
   42    17     1      0.1090 284512.000
   43    18     1      0.1090 284512.000
   44    18     1      0.1090 284512.000
   45    19     1      0.0945 462750.400

[ angles ]
;  ai    aj    ak funct            c0            c1            c2            c3
    1     2     3     1    109.500    418.400
    2     3     4     1    109.500    418.400
    3     4     5     1    109.500    502.080
    4     5     6     1    109.500    418.400
    5     6     7     1    109.500    418.400
    6     7     8     1    109.500    502.080
    7     8     9     1    109.500    418.400
    8     9    10     1    109.500    418.400
    9    10    11     1    109.500    502.080
   10    11    12     1    109.500    418.400
   11    12    13     1    109.500    418.400
   12    13    14     1    109.500    502.080
   13    14    15     1    109.500    418.400
   14    15    16     1    109.500    418.400
   15    16    17     1    109.500    502.080
   16    17    18     1    109.500    418.400
   17    18    19     1    109.500    418.400
    2     1    20     1    108.500    460.240
    1     2    21     1    109.500    292.880
```

1	2	22	1	109.500	292.880
2	3	23	1	110.700	313.800
2	3	24	1	110.700	313.800
4	5	25	1	109.500	292.880
4	5	26	1	109.500	292.880
5	6	27	1	110.700	313.800
5	6	28	1	110.700	313.800
7	8	29	1	109.500	292.880
7	8	30	1	109.500	292.880
8	9	31	1	110.700	313.800
8	9	32	1	110.700	313.800
10	11	33	1	109.500	292.880
10	11	34	1	109.500	292.880
11	12	35	1	110.700	313.800
11	12	36	1	110.700	313.800
13	14	37	1	109.500	292.880
13	14	38	1	109.500	292.880
14	15	39	1	110.700	313.800
14	15	40	1	110.700	313.800
16	17	41	1	109.500	292.880
16	17	42	1	109.500	292.880
17	18	43	1	110.700	313.800
17	18	44	1	110.700	313.800
18	19	45	1	108.500	460.240
9	8	30	1	110.700	313.800
18	17	41	1	110.700	313.800
10	9	32	1	109.500	292.880
7	6	28	1	109.500	292.880
3	2	21	1	110.700	313.800
27	6	28	1	107.800	276.144
9	8	29	1	110.700	313.800
4	3	23	1	109.500	292.880
37	14	38	1	107.800	276.144
12	11	33	1	110.700	313.800
13	12	36	1	109.500	292.880
23	3	24	1	107.800	276.144
43	18	44	1	107.800	276.144
3	2	22	1	110.700	313.800
13	12	35	1	109.500	292.880
16	15	39	1	109.500	292.880
35	12	36	1	107.800	276.144
15	14	37	1	110.700	313.800
15	14	38	1	110.700	313.800
39	15	40	1	107.800	276.144
16	15	40	1	109.500	292.880
19	18	43	1	109.500	292.880
33	11	34	1	107.800	276.144
7	6	27	1	109.500	292.880
25	5	26	1	107.800	276.144
19	18	44	1	109.500	292.880
4	3	24	1	109.500	292.880
10	9	31	1	109.500	292.880
12	11	34	1	110.700	313.800
41	17	42	1	107.800	276.144
6	5	26	1	110.700	313.800
31	9	32	1	107.800	276.144
6	5	25	1	110.700	313.800

```
   18    17    42     1    110.700    313.800
   29     8    30     1    107.800    276.144
   21     2    22     1    107.800    276.144

[ dihedrals ]
; IMPROPER DIHEDRAL ANGLES
;  ai    aj    ak    al funct             c0             c1             c2             c3
c4             c5

[ dihedrals ]
; PROPER DIHEDRAL ANGLES
;  ai    aj    ak    al funct             c0             c1             c2             c3
c4             c5
   12   11   10    9        3       1.715   2.845   1.046  -5.607  -0.000   0.000
    6    5    4    3        3       1.715   2.845   1.046  -5.607  -0.000   0.000
   18   17   16   15        3       1.715   2.845   1.046  -5.607  -0.000   0.000
    9    8    7    6        3       1.715   2.845   1.046  -5.607  -0.000   0.000
   15   14   13   12        3       1.715   2.845   1.046  -5.607  -0.000   0.000
    5    4    3    2        3       1.715   2.845   1.046  -5.607  -0.000   0.000
    8    7    6    5        3       1.715   2.845   1.046  -5.607  -0.000   0.000
   17   16   15   14        3       1.715   2.845   1.046  -5.607  -0.000   0.000
   11   10    9    8        3       1.715   2.845   1.046  -5.607  -0.000   0.000
   14   13   12   11        3       1.715   2.845   1.046  -5.607  -0.000   0.000
   32    9    8   30        3       0.628   1.883   0.000  -2.510  -0.000   0.000
   35   12   11   34        3       0.628   1.883   0.000  -2.510  -0.000   0.000
   43   18   17   42        3       0.628   1.883   0.000  -2.510  -0.000   0.000
   31    9    8   29        3       0.628   1.883   0.000  -2.510  -0.000   0.000
   40   15   14   38        3       0.628   1.883   0.000  -2.510  -0.000   0.000
   44   18   17   42        3       0.628   1.883   0.000  -2.510  -0.000   0.000
   27    6    5   25        3       0.628   1.883   0.000  -2.510  -0.000   0.000
   24    3    2   21        3       0.628   1.883   0.000  -2.510  -0.000   0.000
   23    3    2   22        3       0.628   1.883   0.000  -2.510  -0.000   0.000
   40   15   14   37        3       0.628   1.883   0.000  -2.510  -0.000   0.000
   44   18   17   41        3       0.628   1.883   0.000  -2.510  -0.000   0.000
   39   15   14   37        3       0.628   1.883   0.000  -2.510  -0.000   0.000
   35   12   11   33        3       0.628   1.883   0.000  -2.510  -0.000   0.000
   43   18   17   41        3       0.628   1.883   0.000  -2.510  -0.000   0.000
   39   15   14   38        3       0.628   1.883   0.000  -2.510  -0.000   0.000
   24    3    2   22        3       0.628   1.883   0.000  -2.510  -0.000   0.000
   23    3    2   21        3       0.628   1.883   0.000  -2.510  -0.000   0.000
   32    9    8   29        3       0.628   1.883   0.000  -2.510  -0.000   0.000
   28    6    5   26        3       0.628   1.883   0.000  -2.510  -0.000   0.000
   31    9    8   30        3       0.628   1.883   0.000  -2.510  -0.000   0.000
   36   12   11   34        3       0.628   1.883   0.000  -2.510  -0.000   0.000
   36   12   11   33        3       0.628   1.883   0.000  -2.510  -0.000   0.000
   27    6    5   26        3       0.628   1.883   0.000  -2.510  -0.000   0.000
   28    6    5   25        3       0.628   1.883   0.000  -2.510  -0.000   0.000
   23    3    2    1        3       0.979   2.937   0.000  -3.916  -0.000   0.000
   41   17   18   19        3       0.979   2.937   0.000  -3.916  -0.000   0.000
   24    3    2    1        3       0.979   2.937   0.000  -3.916  -0.000   0.000
   42   17   18   19        3       0.979   2.937   0.000  -3.916  -0.000   0.000
   27    6    5    4        3       0.979   2.937   0.000  -3.916  -0.000   0.000
   29    8    9   10        3       0.979   2.937   0.000  -3.916  -0.000   0.000
   38   14   15   16        3       0.979   2.937   0.000  -3.916  -0.000   0.000
   32    9    8    7        3       0.979   2.937   0.000  -3.916  -0.000   0.000
   31    9    8    7        3       0.979   2.937   0.000  -3.916  -0.000   0.000
   22    2    3    4        3       0.979   2.937   0.000  -3.916  -0.000   0.000
```

```
   36   12   11   10         3       0.979   2.937   0.000  -3.916  -0.000   0.000
   33   11   12   13         3       0.979   2.937   0.000  -3.916  -0.000   0.000
   26    5    6    7         3       0.979   2.937   0.000  -3.916  -0.000   0.000
   34   11   12   13         3       0.979   2.937   0.000  -3.916  -0.000   0.000
   21    2    3    4         3       0.979   2.937   0.000  -3.916  -0.000   0.000
   43   18   17   16         3       0.979   2.937   0.000  -3.916  -0.000   0.000
   44   18   17   16         3       0.979   2.937   0.000  -3.916  -0.000   0.000
   37   14   15   16         3       0.979   2.937   0.000  -3.916  -0.000   0.000
   39   15   14   13         3       0.979   2.937   0.000  -3.916  -0.000   0.000
   35   12   11   10         3       0.979   2.937   0.000  -3.916  -0.000   0.000
   28    6    5    4         3       0.979   2.937   0.000  -3.916  -0.000   0.000
   25    5    6    7         3       0.979   2.937   0.000  -3.916  -0.000   0.000
   30    8    9   10         3       0.979   2.937   0.000  -3.916  -0.000   0.000
   40   15   14   13         3       0.979   2.937   0.000  -3.916  -0.000   0.000
   22    2    1   20         3       0.736   2.209   0.000  -2.946  -0.000   0.000
   21    2    1   20         3       0.736   2.209   0.000  -2.946  -0.000   0.000
   33   11   10    9         3       1.590   4.770   0.000  -6.360  -0.000   0.000
   23    3    4    5         3       1.590   4.770   0.000  -6.360  -0.000   0.000
   38   14   13   12         3       1.590   4.770   0.000  -6.360  -0.000   0.000
   29    8    7    6         3       1.590   4.770   0.000  -6.360  -0.000   0.000
   39   15   16   17         3       1.590   4.770   0.000  -6.360  -0.000   0.000
   36   12   13   14         3       1.590   4.770   0.000  -6.360  -0.000   0.000
   34   11   10    9         3       1.590   4.770   0.000  -6.360  -0.000   0.000
   30    8    7    6         3       1.590   4.770   0.000  -6.360  -0.000   0.000
   32    9   10   11         3       1.590   4.770   0.000  -6.360  -0.000   0.000
   25    5    4    3         3       1.590   4.770   0.000  -6.360  -0.000   0.000
   28    6    7    8         3       1.590   4.770   0.000  -6.360  -0.000   0.000
   27    6    7    8         3       1.590   4.770   0.000  -6.360  -0.000   0.000
   42   17   16   15         3       1.590   4.770   0.000  -6.360  -0.000   0.000
   26    5    4    3         3       1.590   4.770   0.000  -6.360  -0.000   0.000
   24    3    4    5         3       1.590   4.770   0.000  -6.360  -0.000   0.000
   41   17   16   15         3       1.590   4.770   0.000  -6.360  -0.000   0.000
   31    9   10   11         3       1.590   4.770   0.000  -6.360  -0.000   0.000
   35   12   13   14         3       1.590   4.770   0.000  -6.360  -0.000   0.000
   40   15   16   17         3       1.590   4.770   0.000  -6.360  -0.000   0.000
   37   14   13   12         3       1.590   4.770   0.000  -6.360  -0.000   0.000
   20    1    2    3         3      -0.444   3.833   0.728  -4.117  -0.000   0.000
   45   19   18   17         3      -0.444   3.833   0.728  -4.117  -0.000   0.000
   45   19   18   44         3       0.736   2.209   0.000  -2.946  -0.000   0.000
   45   19   18   43         3       0.736   2.209   0.000  -2.946  -0.000   0.000
   19   18   17   16         3       9.035  -9.035   0.000  -0.000  -0.000   0.000
    4    3    2    1         3       9.035  -9.035   0.000  -0.000  -0.000   0.000
   13   12   11   10         3      -1.151   1.151   0.000  -0.000  -0.000   0.000
   10    9    8    7         3      -1.151   1.151   0.000  -0.000  -0.000   0.000
    7    6    5    4         3      -1.151   1.151   0.000  -0.000  -0.000   0.000
   16   15   14   13         3      -1.151   1.151   0.000  -0.000  -0.000   0.000

[ pairs ]
     1     4    1
     2     5    1
     3     6    1
     4     7    1
     5     8    1
     6     9    1
     7    10    1
     8    11    1
     9    12    1
```

10	13	1
3	20	1
1	23	1
11	14	1
4	21	1
1	24	1
4	22	1
12	15	1
5	23	1
3	25	1
13	16	1
5	24	1
3	26	1
14	17	1
4	27	1
7	25	1
4	28	1
15	18	1
7	26	1
16	19	1
8	27	1
6	29	1
8	28	1
6	30	1
7	31	1
10	29	1
7	32	1
10	30	1
20	21	1
20	22	1
11	31	1
9	33	1
11	32	1
9	34	1
21	23	1
22	23	1
21	24	1
10	35	1
22	24	1
13	33	1
10	36	1
13	34	1
14	35	1
12	37	1
14	36	1
12	38	1
25	27	1
13	39	1
26	27	1
25	28	1
16	37	1
13	40	1
26	28	1
16	38	1
17	39	1
15	41	1
17	40	1

```
    15    42    1
    16    43    1
    29    31    1
    19    41    1
    16    44    1
    30    31    1
    29    32    1
    19    42    1
    30    32    1
    17    45    1
    33    35    1
    34    35    1
    33    36    1
    34    36    1
    37    39    1
    38    39    1
    37    40    1
    38    40    1
    41    43    1
    42    43    1
    41    44    1
    42    44    1
    43    45    1
    44    45    1
```

Heptaethylene Glycol

```
;
; GENERATED BY LigParGen Server
; Jorgensen Lab @ Yale University
;
[ moleculetype ]
; Name               nrexcl
HEG                   3
[ atoms ]
;   nr       type  resnr residue  atom   cgnr     charge       mass
     1   peg_OA        1    HEG   OAH1      1    -0.6890    15.9990
     2   peg_C         1    HEG   CH1       1     0.1083    12.0110
     3   peg_C         1    HEG   CH2       1      0.008    12.0110
     4   peg_OE        1    HEG   OEH1      1    -0.3846    15.9990
     5   peg_C         1    HEG   CH3       1     0.0073    12.0110
     6   peg_C         1    HEG   CH4       1     0.0087    12.0110
     7   peg_OE        1    HEG   OEH2      1    -0.3855    15.9990
     8   peg_C         1    HEG   CH5       1     0.0088    12.0110
     9   peg_C         1    HEG   CH6       1      0.005    12.0110
    10   peg_OE        1    HEG   OEH3      1    -0.3762    15.9990
    11   peg_C         1    HEG   CH7       1     0.0105    12.0110
    12   peg_C         1    HEG   CH8       1     0.0107    12.0110
    13   peg_OE        1    HEG   OEH4      1    -0.3759    15.9990
    14   peg_C         1    HEG   CH9       1     0.0055    12.0110
    15   peg_C         1    HEG   CHA       1     0.0085    12.0110
    16   peg_OE        1    HEG   OEH5      1    -0.3859    15.9990
    17   peg_C         1    HEG   CHB       1     0.0087    12.0110
    18   peg_C         1    HEG   CHC       1     0.0065    12.0110
    19   peg_OE        1    HEG   OEH6      1    -0.3985    15.9990
    20   peg_C         1    HEG   CHD       1     -0.041    12.0110
```

```
    21   peg_C         1    HEG   CHE       1     0.1082    12.0110
    22   peg_OA        1    HEG   OAH2      1    -0.6863    15.9990
    23   peg_HA        1    HEG   HAH1      1     0.4173     1.0080
    24   peg_HC        1    HEG   HCH1      1     0.0821     1.0080
    25   peg_HC        1    HEG   HCH2      1     0.0821     1.0080
    26   peg_HC        1    HEG   HCH3      1     0.0923     1.0080
    27   peg_HC        1    HEG   HCH4      1     0.0923     1.0080
    28   peg_HC        1    HEG   HCH5      1     0.0913     1.0080
    29   peg_HC        1    HEG   HCH6      1     0.0913     1.0080
    30   peg_HC        1    HEG   HCH7      1     0.0915     1.0080
    31   peg_HC        1    HEG   HCH8      1     0.0915     1.0080
    32   peg_HC        1    HEG   HCH9      1     0.0925     1.0080
    33   peg_HC        1    HEG   HCHA      2     0.0925     1.0080
    34   peg_HC        1    HEG   HCHB      2     0.0881     1.0080
    35   peg_HC        1    HEG   HCHC      2     0.0881     1.0080
    36   peg_HC        1    HEG   HCHD      2     0.0931     1.0080
    37   peg_HC        1    HEG   HCHE      2     0.0931     1.0080
    38   peg_HC        1    HEG   HCHF      2     0.0929     1.0080
    39   peg_HC        1    HEG   HCHG      2     0.0929     1.0080
    40   peg_HC        1    HEG   HCHH      2     0.0878     1.0080
    41   peg_HC        1    HEG   HCHI      2     0.0878     1.0080
    42   peg_HC        1    HEG   HCHJ      2      0.093     1.0080
    43   peg_HC        1    HEG   HCHK      2      0.093     1.0080
    44   peg_HC        1    HEG   HCHL      2     0.0915     1.0080
    45   peg_HC        1    HEG   HCHM      2     0.0915     1.0080
    46   peg_HC        1    HEG   HCHN      2     0.0915     1.0080
    47   peg_HC        1    HEG   HCHO      2     0.0915     1.0080
    48   peg_HC        1    HEG   HCHP      2     0.0943     1.0080
    49   peg_HC        1    HEG   HCHQ      2     0.0943     1.0080
    50   peg_HC        1    HEG   HCHR      2     0.1067     1.0080
    51   peg_HC        1    HEG   HCHS      2     0.1067     1.0080
    52   peg_HA        1    HEG   HAH2      2     0.4237     1.0080
[ bonds ]
    2     1     1      0.1410 267776.000
    3     2     1      0.1529 224262.400
    4     3     1      0.1410 267776.000
    5     4     1      0.1410 267776.000
    6     5     1      0.1529 224262.400
    7     6     1      0.1410 267776.000
    8     7     1      0.1410 267776.000
    9     8     1      0.1529 224262.400
   10     9     1      0.1410 267776.000
   11    10     1      0.1410 267776.000
   12    11     1      0.1529 224262.400
   13    12     1      0.1410 267776.000
   14    13     1      0.1410 267776.000
   15    14     1      0.1529 224262.400
   16    15     1      0.1410 267776.000
   17    16     1      0.1410 267776.000
   18    17     1      0.1529 224262.400
   19    18     1      0.1410 267776.000
   20    19     1      0.1410 267776.000
   21    20     1      0.1529 224262.400
   22    21     1      0.1410 267776.000
   23     1     1      0.0945 462750.400
   24     2     1      0.1090 284512.000
   25     2     1      0.1090 284512.000
```

```
    26     3     1       0.1090 284512.000
    27     3     1       0.1090 284512.000
    28     5     1       0.1090 284512.000
    29     5     1       0.1090 284512.000
    30     6     1       0.1090 284512.000
    31     6     1       0.1090 284512.000
    32     8     1       0.1090 284512.000
    33     8     1       0.1090 284512.000
    34     9     1       0.1090 284512.000
    35     9     1       0.1090 284512.000
    36    11     1       0.1090 284512.000
    37    11     1       0.1090 284512.000
    38    12     1       0.1090 284512.000
    39    12     1       0.1090 284512.000
    40    14     1       0.1090 284512.000
    41    14     1       0.1090 284512.000
    42    15     1       0.1090 284512.000
    43    15     1       0.1090 284512.000
    44    17     1       0.1090 284512.000
    45    17     1       0.1090 284512.000
    46    18     1       0.1090 284512.000
    47    18     1       0.1090 284512.000
    48    20     1       0.1090 284512.000
    49    20     1       0.1090 284512.000
    50    21     1       0.1090 284512.000
    51    21     1       0.1090 284512.000
    52    22     1       0.0945 462750.400

[ angles ]
;  ai    aj    ak funct            c0            c1            c2            c3
     1     2     3     1    109.500    418.400
     2     3     4     1    109.500    418.400
     3     4     5     1    109.500    502.080
     4     5     6     1    109.500    418.400
     5     6     7     1    109.500    418.400
     6     7     8     1    109.500    502.080
     7     8     9     1    109.500    418.400
     8     9    10     1    109.500    418.400
     9    10    11     1    109.500    502.080
    10    11    12     1    109.500    418.400
    11    12    13     1    109.500    418.400
    12    13    14     1    109.500    502.080
    13    14    15     1    109.500    418.400
    14    15    16     1    109.500    418.400
    15    16    17     1    109.500    502.080
    16    17    18     1    109.500    418.400
    17    18    19     1    109.500    418.400
    18    19    20     1    109.500    502.080
    19    20    21     1    109.500    418.400
    20    21    22     1    109.500    418.400
     2     1    23     1    108.500    460.240
     1     2    24     1    109.500    292.880
     1     2    25     1    109.500    292.880
     2     3    26     1    110.700    313.800
     2     3    27     1    110.700    313.800
     4     5    28     1    109.500    292.880
     4     5    29     1    109.500    292.880
```

5	6	30	1	110.700	313.800
5	6	31	1	110.700	313.800
7	8	32	1	109.500	292.880
7	8	33	1	109.500	292.880
8	9	34	1	110.700	313.800
8	9	35	1	110.700	313.800
10	11	36	1	109.500	292.880
10	11	37	1	109.500	292.880
11	12	38	1	110.700	313.800
11	12	39	1	110.700	313.800
13	14	40	1	109.500	292.880
13	14	41	1	109.500	292.880
14	15	42	1	110.700	313.800
14	15	43	1	110.700	313.800
16	17	44	1	109.500	292.880
16	17	45	1	109.500	292.880
17	18	46	1	110.700	313.800
17	18	47	1	110.700	313.800
19	20	48	1	109.500	292.880
19	20	49	1	109.500	292.880
20	21	50	1	110.700	313.800
20	21	51	1	110.700	313.800
21	22	52	1	108.500	460.240
12	11	37	1	110.700	313.800
19	18	47	1	109.500	292.880
13	12	38	1	109.500	292.880
15	14	40	1	110.700	313.800
44	17	45	1	107.800	276.144
16	15	42	1	109.500	292.880
7	6	31	1	109.500	292.880
40	14	41	1	107.800	276.144
48	20	49	1	107.800	276.144
3	2	25	1	110.700	313.800
28	5	29	1	107.800	276.144
16	15	43	1	109.500	292.880
15	14	41	1	110.700	313.800
36	11	37	1	107.800	276.144
21	20	49	1	110.700	313.800
6	5	28	1	110.700	313.800
21	20	48	1	110.700	313.800
32	8	33	1	107.800	276.144
13	12	39	1	109.500	292.880
12	11	36	1	110.700	313.800
42	15	43	1	107.800	276.144
4	3	27	1	109.500	292.880
24	2	25	1	107.800	276.144
10	9	35	1	109.500	292.880
22	21	51	1	109.500	292.880
22	21	50	1	109.500	292.880
18	17	45	1	110.700	313.800
4	3	26	1	109.500	292.880
9	8	32	1	110.700	313.800
46	18	47	1	107.800	276.144
34	9	35	1	107.800	276.144
9	8	33	1	110.700	313.800
7	6	30	1	109.500	292.880
30	6	31	1	107.800	276.144

```
   26     3    27     1    107.800    276.144
   18    17    44     1    110.700    313.800
   10     9    34     1    109.500    292.880
   38    12    39     1    107.800    276.144
   19    18    46     1    109.500    292.880
    3     2    24     1    110.700    313.800
    6     5    29     1    110.700    313.800
   50    21    51     1    107.800    276.144

[ dihedrals ]
; IMPROPER DIHEDRAL ANGLES
;  ai    aj    ak    al funct             c0             c1             c2             c3
c4             c5

[ dihedrals ]
; PROPER DIHEDRAL ANGLES
;  ai    aj    ak    al funct             c0             c1             c2             c3
c4             c5
   12   11   10    9         3       1.715   2.845   1.046  -5.607  -0.000   0.000
   18   17   16   15         3       1.715   2.845   1.046  -5.607  -0.000   0.000
   15   14   13   12         3       1.715   2.845   1.046  -5.607  -0.000   0.000
    9    8    7    6         3       1.715   2.845   1.046  -5.607  -0.000   0.000
   21   20   19   18         3       1.715   2.845   1.046  -5.607  -0.000   0.000
    6    5    4    3         3       1.715   2.845   1.046  -5.607  -0.000   0.000
   11   10    9    8         3       1.715   2.845   1.046  -5.607  -0.000   0.000
   20   19   18   17         3       1.715   2.845   1.046  -5.607  -0.000   0.000
   14   13   12   11         3       1.715   2.845   1.046  -5.607  -0.000   0.000
   17   16   15   14         3       1.715   2.845   1.046  -5.607  -0.000   0.000
    5    4    3    2         3       1.715   2.845   1.046  -5.607  -0.000   0.000
    8    7    6    5         3       1.715   2.845   1.046  -5.607  -0.000   0.000
   43   15   14   41         3       0.628   1.883   0.000  -2.510  -0.000   0.000
   35    9    8   32         3       0.628   1.883   0.000  -2.510  -0.000   0.000
   34    9    8   33         3       0.628   1.883   0.000  -2.510  -0.000   0.000
   27    3    2   24         3       0.628   1.883   0.000  -2.510  -0.000   0.000
   26    3    2   25         3       0.628   1.883   0.000  -2.510  -0.000   0.000
   34    9    8   32         3       0.628   1.883   0.000  -2.510  -0.000   0.000
   46   18   17   45         3       0.628   1.883   0.000  -2.510  -0.000   0.000
   39   12   11   37         3       0.628   1.883   0.000  -2.510  -0.000   0.000
   50   21   20   48         3       0.628   1.883   0.000  -2.510  -0.000   0.000
   30    6    5   29         3       0.628   1.883   0.000  -2.510  -0.000   0.000
   51   21   20   48         3       0.628   1.883   0.000  -2.510  -0.000   0.000
   38   12   11   36         3       0.628   1.883   0.000  -2.510  -0.000   0.000
   42   15   14   40         3       0.628   1.883   0.000  -2.510  -0.000   0.000
   47   18   17   44         3       0.628   1.883   0.000  -2.510  -0.000   0.000
   43   15   14   40         3       0.628   1.883   0.000  -2.510  -0.000   0.000
   39   12   11   36         3       0.628   1.883   0.000  -2.510  -0.000   0.000
   38   12   11   37         3       0.628   1.883   0.000  -2.510  -0.000   0.000
   35    9    8   33         3       0.628   1.883   0.000  -2.510  -0.000   0.000
   50   21   20   49         3       0.628   1.883   0.000  -2.510  -0.000   0.000
   30    6    5   28         3       0.628   1.883   0.000  -2.510  -0.000   0.000
   26    3    2   24         3       0.628   1.883   0.000  -2.510  -0.000   0.000
   31    6    5   29         3       0.628   1.883   0.000  -2.510  -0.000   0.000
   46   18   17   44         3       0.628   1.883   0.000  -2.510  -0.000   0.000
   51   21   20   49         3       0.628   1.883   0.000  -2.510  -0.000   0.000
   27    3    2   25         3       0.628   1.883   0.000  -2.510  -0.000   0.000
   42   15   14   41         3       0.628   1.883   0.000  -2.510  -0.000   0.000
   47   18   17   45         3       0.628   1.883   0.000  -2.510  -0.000   0.000
```

31	6	5	28	3	0.628	1.883	0.000	-2.510	-0.000	0.000
48	20	21	22	3	0.979	2.937	0.000	-3.916	-0.000	0.000
27	3	2	1	3	0.979	2.937	0.000	-3.916	-0.000	0.000
49	20	21	22	3	0.979	2.937	0.000	-3.916	-0.000	0.000
26	3	2	1	3	0.979	2.937	0.000	-3.916	-0.000	0.000
29	5	6	7	3	0.979	2.937	0.000	-3.916	-0.000	0.000
43	15	14	13	3	0.979	2.937	0.000	-3.916	-0.000	0.000
42	15	14	13	3	0.979	2.937	0.000	-3.916	-0.000	0.000
34	9	8	7	3	0.979	2.937	0.000	-3.916	-0.000	0.000
35	9	8	7	3	0.979	2.937	0.000	-3.916	-0.000	0.000
24	2	3	4	3	0.979	2.937	0.000	-3.916	-0.000	0.000
51	21	20	19	3	0.979	2.937	0.000	-3.916	-0.000	0.000
37	11	12	13	3	0.979	2.937	0.000	-3.916	-0.000	0.000
28	5	6	7	3	0.979	2.937	0.000	-3.916	-0.000	0.000
32	8	9	10	3	0.979	2.937	0.000	-3.916	-0.000	0.000
47	18	17	16	3	0.979	2.937	0.000	-3.916	-0.000	0.000
46	18	17	16	3	0.979	2.937	0.000	-3.916	-0.000	0.000
45	17	18	19	3	0.979	2.937	0.000	-3.916	-0.000	0.000
33	8	9	10	3	0.979	2.937	0.000	-3.916	-0.000	0.000
31	6	5	4	3	0.979	2.937	0.000	-3.916	-0.000	0.000
38	12	11	10	3	0.979	2.937	0.000	-3.916	-0.000	0.000
44	17	18	19	3	0.979	2.937	0.000	-3.916	-0.000	0.000
40	14	15	16	3	0.979	2.937	0.000	-3.916	-0.000	0.000
25	2	3	4	3	0.979	2.937	0.000	-3.916	-0.000	0.000
36	11	12	13	3	0.979	2.937	0.000	-3.916	-0.000	0.000
39	12	11	10	3	0.979	2.937	0.000	-3.916	-0.000	0.000
41	14	15	16	3	0.979	2.937	0.000	-3.916	-0.000	0.000
50	21	20	19	3	0.979	2.937	0.000	-3.916	-0.000	0.000
30	6	5	4	3	0.979	2.937	0.000	-3.916	-0.000	0.000
25	2	1	23	3	0.736	2.209	0.000	-2.946	-0.000	0.000
24	2	1	23	3	0.736	2.209	0.000	-2.946	-0.000	0.000
31	6	7	8	3	1.590	4.770	0.000	-6.360	-0.000	0.000
45	17	16	15	3	1.590	4.770	0.000	-6.360	-0.000	0.000
40	14	13	12	3	1.590	4.770	0.000	-6.360	-0.000	0.000
29	5	4	3	3	1.590	4.770	0.000	-6.360	-0.000	0.000
47	18	19	20	3	1.590	4.770	0.000	-6.360	-0.000	0.000
36	11	10	9	3	1.590	4.770	0.000	-6.360	-0.000	0.000
41	14	13	12	3	1.590	4.770	0.000	-6.360	-0.000	0.000
32	8	7	6	3	1.590	4.770	0.000	-6.360	-0.000	0.000
26	3	4	5	3	1.590	4.770	0.000	-6.360	-0.000	0.000
27	3	4	5	3	1.590	4.770	0.000	-6.360	-0.000	0.000
35	9	10	11	3	1.590	4.770	0.000	-6.360	-0.000	0.000
46	18	19	20	3	1.590	4.770	0.000	-6.360	-0.000	0.000
48	20	19	18	3	1.590	4.770	0.000	-6.360	-0.000	0.000
39	12	13	14	3	1.590	4.770	0.000	-6.360	-0.000	0.000
49	20	19	18	3	1.590	4.770	0.000	-6.360	-0.000	0.000
37	11	10	9	3	1.590	4.770	0.000	-6.360	-0.000	0.000
33	8	7	6	3	1.590	4.770	0.000	-6.360	-0.000	0.000
28	5	4	3	3	1.590	4.770	0.000	-6.360	-0.000	0.000
42	15	16	17	3	1.590	4.770	0.000	-6.360	-0.000	0.000
30	6	7	8	3	1.590	4.770	0.000	-6.360	-0.000	0.000
43	15	16	17	3	1.590	4.770	0.000	-6.360	-0.000	0.000
44	17	16	15	3	1.590	4.770	0.000	-6.360	-0.000	0.000
38	12	13	14	3	1.590	4.770	0.000	-6.360	-0.000	0.000
34	9	10	11	3	1.590	4.770	0.000	-6.360	-0.000	0.000
52	22	21	20	3	-0.444	3.833	0.728	-4.117	-0.000	0.000
23	1	2	3	3	-0.444	3.833	0.728	-4.117	-0.000	0.000

```
   52   22   21   51        3       0.736   2.209   0.000  -2.946  -0.000   0.000
   52   22   21   50        3       0.736   2.209   0.000  -2.946  -0.000   0.000
   22   21   20   19        3       9.035  -9.035   0.000  -0.000  -0.000   0.000
    4    3    2    1        3       9.035  -9.035   0.000  -0.000  -0.000   0.000
   10    9    8    7        3      -1.151   1.151   0.000  -0.000  -0.000   0.000
   13   12   11   10        3      -1.151   1.151   0.000  -0.000  -0.000   0.000
   16   15   14   13        3      -1.151   1.151   0.000  -0.000  -0.000   0.000
    7    6    5    4        3      -1.151   1.151   0.000  -0.000  -0.000   0.000
   19   18   17   16        3      -1.151   1.151   0.000  -0.000  -0.000   0.000

[ pairs ]
     1     4    1
     2     5    1
     3     6    1
     4     7    1
     5     8    1
     6     9    1
     7    10    1
     8    11    1
     9    12    1
    10    13    1
    11    14    1
     3    23    1
    12    15    1
     1    26    1
     4    24    1
     1    27    1
    13    16    1
     4    25    1
    14    17    1
     5    26    1
     3    28    1
     5    27    1
     3    29    1
    15    18    1
     4    30    1
    16    19    1
     7    28    1
     4    31    1
     7    29    1
    17    20    1
     8    30    1
     6    32    1
    18    21    1
     8    31    1
     6    33    1
    19    22    1
     7    34    1
    10    32    1
     7    35    1
    10    33    1
    11    34    1
     9    36    1
    11    35    1
     9    37    1
    23    24    1
    23    25    1
```

10	38	1
13	36	1
10	39	1
24	26	1
13	37	1
25	26	1
24	27	1
25	27	1
14	38	1
12	40	1
14	39	1
12	41	1
13	42	1
16	40	1
13	43	1
16	41	1
28	30	1
29	30	1
28	31	1
17	42	1
15	44	1
29	31	1
17	43	1
15	45	1
16	46	1
19	44	1
16	47	1
19	45	1
32	34	1
20	46	1
18	48	1
33	34	1
32	35	1
20	47	1
18	49	1
33	35	1
19	50	1
22	48	1
19	51	1
22	49	1
20	52	1
36	38	1
37	38	1
36	39	1
37	39	1
40	42	1
41	42	1
40	43	1
41	43	1
44	46	1
45	46	1
44	47	1
45	47	1
48	50	1
49	50	1
48	51	1
49	51	1

```
    50    52    1
    51    52    1
```

4.1.2 Structure Coordinate files

```
Diethylene glycol

LIGPARGEN GENERATED GRO FILE
   17
    1DEG   OAD1    1   0.100   0.100   0.000
    1DEG   CD1     2  -0.042   0.100   0.000
    1DEG   CD2     3  -0.094   0.100   0.143
    1DEG   OED1    4  -0.237   0.100   0.143
    1DEG   CD3     5  -0.289   0.100   0.276
    1DEG   CD4     6  -0.441   0.100   0.269
    1DEG   OAD2    7  -0.487  -0.016   0.201
    1DEG   HAD1    8   0.128   0.100  -0.093
    1DEG   HCD1    9  -0.078   0.012  -0.054
    1DEG   HCD2   10  -0.078   0.189  -0.054
    1DEG   HCD3   11  -0.057   0.189   0.196
    1DEG   HCD4   12  -0.057   0.011   0.196
    1DEG   HCD5   13  -0.254   0.189   0.330
    1DEG   HCD6   14  -0.254   0.009   0.327
    1DEG   HCD7   15  -0.477   0.187   0.213
    1DEG   HCD8   16  -0.485   0.101   0.369
    1DEG   HAD2   17  -0.433  -0.021   0.119
   1.00000   1.00000   1.00000

Triethylene glycol

LIGPARGEN GENERATED GRO FILE
   24
    1REG   OAR1    1   0.100   0.100   0.000
    1REG   CR1     2  -0.042   0.100   0.000
    1REG   CR2     3  -0.095   0.100   0.143
    1REG   OER1    4  -0.237   0.100   0.140
    1REG   CR3     5  -0.292   0.088   0.272
    1REG   CR4     6  -0.445   0.082   0.263
    1REG   OER2    7  -0.485  -0.041   0.202
    1REG   CR5     8  -0.627  -0.050   0.191
    1REG   CR6     9  -0.661  -0.177   0.114
    1REG   OAR2   10  -0.608  -0.291   0.182
    1REG   HAR1   11   0.128   0.100  -0.093
    1REG   HCR1   12  -0.078   0.011  -0.054
    1REG   HCR2   13  -0.078   0.188  -0.054
    1REG   HCR3   14  -0.058   0.189   0.196
    1REG   HCR4   15  -0.058   0.011   0.196
    1REG   HCR5   16  -0.262   0.175   0.331
    1REG   HCR6   17  -0.254  -0.003   0.319
    1REG   HCR7   18  -0.482   0.166   0.203
    1REG   HCR8   19  -0.488   0.087   0.364
    1REG   HCR9   20  -0.667   0.037   0.139
    1REG   HCRA   21  -0.669  -0.056   0.293
    1REG   HCRB   22  -0.617  -0.175   0.014
    1REG   HCRC   23  -0.770  -0.190   0.106
```

```
    1REG   HAR2   24  -0.514  -0.268   0.199
   1.00000   1.00000   1.00000
```

Tetraethyene glycol

```
LIGPARGEN GENERATED GRO FILE
   31
    1TEG   OAT1    1   0.100   0.100   0.000
    1TEG   CT1     2  -0.042   0.100   0.000
    1TEG   CT2     3  -0.095   0.100   0.143
    1TEG   OET1    4  -0.238   0.100   0.141
    1TEG   CT3     5  -0.291   0.100   0.274
    1TEG   CT4     6  -0.443   0.100   0.267
    1TEG   OET2    7  -0.496   0.101   0.400
    1TEG   CT5     8  -0.639   0.101   0.400
    1TEG   CT6     9  -0.689   0.101   0.544
    1TEG   OET3   10  -0.832   0.101   0.545
    1TEG   CT7    11  -0.881   0.101   0.680
    1TEG   CT8    12  -1.034   0.102   0.675
    1TEG   OAT2   13  -1.081  -0.015   0.608
    1TEG   HAT1   14   0.128   0.100  -0.093
    1TEG   HCT1   15  -0.078   0.012  -0.054
    1TEG   HCT2   16  -0.078   0.189  -0.054
    1TEG   HCT3   17  -0.058   0.189   0.196
    1TEG   HCT4   18  -0.058   0.011   0.196
    1TEG   HCT5   19  -0.256   0.189   0.328
    1TEG   HCT6   20  -0.256   0.012   0.328
    1TEG   HCT7   21  -0.478   0.012   0.213
    1TEG   HCT8   22  -0.478   0.189   0.213
    1TEG   HCT9   23  -0.676   0.012   0.347
    1TEG   HCTA   24  -0.676   0.190   0.347
    1TEG   HCTB   25  -0.651   0.190   0.596
    1TEG   HCTC   26  -0.651   0.012   0.596
    1TEG   HCTD   27  -0.845   0.190   0.733
    1TEG   HCTE   28  -0.846   0.011   0.731
    1TEG   HCTF   29  -1.071   0.189   0.620
    1TEG   HCTG   30  -1.076   0.103   0.776
    1TEG   HAT2   31  -1.030  -0.018   0.525
   1.00000   1.00000   1.00000
```

Pentaethylene glycol

```
LIGPARGEN GENERATED GRO FILE
   38
    1PEG   OAP1    1   0.100   0.100   0.000
    1PEG   CP1     2  -0.042   0.100   0.000
    1PEG   CP2     3  -0.095   0.100   0.143
    1PEG   OEP1    4  -0.238   0.100   0.141
    1PEG   CP3     5  -0.291   0.100   0.273
    1PEG   CP4     6  -0.443   0.099   0.266
    1PEG   OEP2    7  -0.496   0.093   0.399
    1PEG   CP5     8  -0.639   0.103   0.400
    1PEG   CP6     9  -0.688   0.102   0.544
    1PEG   OEP3   10  -0.649   0.224   0.609
    1PEG   CP7    11  -0.687   0.226   0.746
    1PEG   CP8    12  -0.631   0.352   0.811
    1PEG   OEP4   13  -0.673   0.359   0.947
```

```
    1PEG   CP9    14  -0.622   0.476   1.011
    1PEG   CPA    15  -0.671   0.478   1.156
    1PEG   OAP2   16  -0.623   0.362   1.224
    1PEG   HAP1   17   0.128   0.100  -0.093
    1PEG   HCP1   18  -0.078   0.012  -0.054
    1PEG   HCP2   19  -0.078   0.189  -0.054
    1PEG   HCP3   20  -0.058   0.189   0.196
    1PEG   HCP4   21  -0.058   0.011   0.196
    1PEG   HCP5   22  -0.257   0.189   0.328
    1PEG   HCP6   23  -0.256   0.011   0.328
    1PEG   HCP7   24  -0.478   0.013   0.208
    1PEG   HCP8   25  -0.478   0.190   0.216
    1PEG   HCP9   26  -0.680   0.017   0.346
    1PEG   HCPA   27  -0.669   0.196   0.350
    1PEG   HCPB   28  -0.645   0.017   0.598
    1PEG   HCPC   29  -0.798   0.095   0.546
    1PEG   HCPD   30  -0.649   0.137   0.798
    1PEG   HCPE   31  -0.796   0.225   0.754
    1PEG   HCPF   32  -0.666   0.440   0.757
    1PEG   HCPG   33  -0.521   0.350   0.806
    1PEG   HCPH   34  -0.657   0.566   0.959
    1PEG   HCPI   35  -0.512   0.472   1.009
    1PEG   HCPJ   36  -0.780   0.476   1.160
    1PEG   HCPK   37  -0.634   0.567   1.208
    1PEG   HAP2   38  -0.645   0.287   1.166
   1.00000   1.00000   1.00000
```

Hexaethylene glycol

```
LIGPARGEN GENERATED GRO FILE
   45
    1XEG   OAX1    1   0.100   0.100   0.000
    1XEG   CX1     2  -0.042   0.100   0.000
    1XEG   CX2     3  -0.094   0.100   0.143
    1XEG   OEX1    4  -0.237   0.100   0.141
    1XEG   CX3     5  -0.290   0.100   0.274
    1XEG   CX4     6  -0.443   0.100   0.267
    1XEG   OEX2    7  -0.495   0.100   0.400
    1XEG   CX5     8  -0.638   0.100   0.399
    1XEG   CX6     9  -0.688   0.100   0.543
    1XEG   OEX3   10  -0.831   0.100   0.543
    1XEG   CX7    11  -0.883   0.100   0.675
    1XEG   CX8    12  -1.036   0.100   0.669
    1XEG   OEX4   13  -1.089   0.101   0.802
    1XEG   CX9    14  -1.231   0.101   0.801
    1XEG   CXA    15  -1.282   0.101   0.944
    1XEG   OEX5   16  -1.425   0.101   0.945
    1XEG   CXB    17  -1.475   0.101   1.079
    1XEG   CXC    18  -1.628   0.102   1.074
    1XEG   OAX2   19  -1.674  -0.015   1.006
    1XEG   HAX1   20   0.128   0.100  -0.093
    1XEG   HCX1   21  -0.078   0.012  -0.054
    1XEG   HCX2   22  -0.078   0.189  -0.054
    1XEG   HCX3   23  -0.058   0.189   0.196
    1XEG   HCX4   24  -0.057   0.011   0.196
    1XEG   HCX5   25  -0.256   0.189   0.328
    1XEG   HCX6   26  -0.256   0.012   0.328
```

```
    1XEG   HCX7   27  -0.477   0.011   0.213
    1XEG   HCX8   28  -0.478   0.188   0.212
    1XEG   HCX9   29  -0.675   0.011   0.347
    1XEG   HCXA   30  -0.675   0.188   0.347
    1XEG   HCXB   31  -0.651   0.189   0.596
    1XEG   HCXC   32  -0.651   0.011   0.596
    1XEG   HCXD   33  -0.849   0.189   0.729
    1XEG   HCXE   34  -0.848   0.012   0.730
    1XEG   HCXF   35  -1.070   0.011   0.615
    1XEG   HCXG   36  -1.071   0.189   0.614
    1XEG   HCXH   37  -1.269   0.012   0.748
    1XEG   HCXI   38  -1.269   0.189   0.748
    1XEG   HCXJ   39  -1.244   0.190   0.997
    1XEG   HCXK   40  -1.244   0.013   0.997
    1XEG   HCXL   41  -1.439   0.190   1.132
    1XEG   HCXM   42  -1.440   0.011   1.130
    1XEG   HCXN   43  -1.664   0.189   1.019
    1XEG   HCXO   44  -1.670   0.102   1.175
    1XEG   HAX2   45  -1.623  -0.017   0.922
   1.00000   1.00000   1.00000
```

Heptaethylene glycol

```
LIGPARGEN GENERATED GRO FILE
   52
    1HEG   OAH1    1   0.100   0.100   0.000
    1HEG   CH1     2  -0.042   0.100   0.000
    1HEG   CH2     3  -0.094   0.100   0.143
    1HEG   OEH1    4  -0.237   0.100   0.141
    1HEG   CH3     5  -0.290   0.100   0.273
    1HEG   CH4     6  -0.443   0.099   0.267
    1HEG   OEH2    7  -0.495   0.099   0.400
    1HEG   CH5     8  -0.637   0.099   0.400
    1HEG   CH6     9  -0.687   0.099   0.544
    1HEG   OEH3   10  -0.829   0.105   0.544
    1HEG   CH7    11  -0.882   0.095   0.676
    1HEG   CH8    12  -1.035   0.093   0.669
    1HEG   OEH4   13  -1.077  -0.029   0.608
    1HEG   CH9    14  -1.218  -0.034   0.591
    1HEG   CHA    15  -1.256  -0.161   0.516
    1HEG   OEH5   16  -1.398  -0.168   0.504
    1HEG   CHB    17  -1.438  -0.286   0.434
    1HEG   CHC    18  -1.590  -0.288   0.425
    1HEG   OEH6   19  -1.633  -0.406   0.356
    1HEG   CHD    20  -1.776  -0.410   0.346
    1HEG   CHE    21  -1.815  -0.537   0.271
    1HEG   OAH2   22  -1.768  -0.652   0.342
    1HEG   HAH1   23   0.128   0.100  -0.093
    1HEG   HCH1   24  -0.078   0.012  -0.054
    1HEG   HCH2   25  -0.078   0.189  -0.054
    1HEG   HCH3   26  -0.058   0.189   0.196
    1HEG   HCH4   27  -0.057   0.011   0.196
    1HEG   HCH5   28  -0.256   0.188   0.328
    1HEG   HCH6   29  -0.255   0.011   0.328
    1HEG   HCH7   30  -0.477   0.011   0.213
    1HEG   HCH8   31  -0.478   0.188   0.212
    1HEG   HCH9   32  -0.675   0.010   0.348
```

```
    1HEG   HCHA   33  -0.675   0.188   0.347
    1HEG   HCHB   34  -0.646   0.186   0.597
    1HEG   HCHC   35  -0.652   0.009   0.595
    1HEG   HCHD   36  -0.849   0.182   0.734
    1HEG   HCHE   37  -0.846   0.003   0.723
    1HEG   HCHF   38  -1.070   0.177   0.609
    1HEG   HCHG   39  -1.077   0.099   0.770
    1HEG   HCHH   40  -1.253   0.054   0.534
    1HEG   HCHI   41  -1.268  -0.032   0.689
    1HEG   HCHJ   42  -1.218  -0.248   0.570
    1HEG   HCHK   43  -1.209  -0.160   0.417
    1HEG   HCHL   44  -1.403  -0.376   0.488
    1HEG   HCHM   45  -1.394  -0.288   0.334
    1HEG   HCHN   46  -1.625  -0.199   0.372
    1HEG   HCHO   47  -1.633  -0.287   0.526
    1HEG   HCHP   48  -1.811  -0.322   0.292
    1HEG   HCHQ   49  -1.818  -0.412   0.447
    1HEG   HCHR   50  -1.769  -0.539   0.171
    1HEG   HCHS   51  -1.923  -0.544   0.261
    1HEG   HAH2   52  -1.674  -0.634   0.361
   1.00000   1.00000   1.00000
```

4.1.3 Molecular Dynamics files

For reaching positional equilibrium “em.mdp”:

```
; very basics of the simulation
define                   = -DFLEXIBLE      ; flexible water so simulation does not
crash
integrator               = steep           ; steepest decent
nsteps                   = 100000          ; number of steepest decent steps
emstep                   = 0.01            ; 0.01 nm step

; next neighbor search and periodic boundary conditions
nstlist                  = 1               ; freq. to update neighbor list & long
range forces (>= 20 w/ GPUs)
rlist                    = 1.4             ; short-range neighbor list cutoff (nm)
cutoff-scheme            = Verlet          ; atom-based neighbor search with an
implicit buffer region
pbc                      = xyz             ; periodicity in x, y and z

; coulomb interaction
coulombtype              = PME             ; particle-mesh ewald summation for long
range (>rcoulomb)
rcoulomb                 = 1.4             ; short-range electrostatic cutoff (in nm)
(with PME,rcoulomb >= rvdw)
coulomb-modifier         = Potential-shift-Verlet ; shifts potential by constant so
potential is 0 at cut-off
fourierspacing           = 0.168           ; spacing of FFT in reciprocal space in
PME long range treatment (in nm)
pme-order                = 4               ; cubic PME interpolation order

; lennard-jones potential handling
vdwtype                  = cutoff          ; simple truncation cutoff
```

```
vdw-modifier              = Potential-shift-Verlet ; shifts potential by constant so
potential is 0 at cut-off
rvdw                      = 1.4             ; short-range vdw cutoff (in nm)
```

For reaching NPT equilibrium, “eq_NPT_328.mdp”

```
; very basics of the simulation
integrator                = md              ; solve newtown's equation of motion
dt                        = 0.002           ; integration time step / ps
nsteps                    = 5000000          ; number of steps (5000000 * 0.002 =
10000 ps = 10 ns)

; remove drifts of the center of mass
comm-mode                 = linear    ; remove COM translation
nstcomm                   = 10              ; number of steps for COM removal
comm-grps                 = System          ; COM translation removed from system

; control frequency of output
nstvout                  = 0                ; write velocities to trajectory file every
number of steps
nstfout                  = 0                ; write forces to trajectory file every
number of steps
nstlog                    = 5000             ; update log file every number of steps
nstcalcenergy             = 10              ; calculate energies/pressures every
nstenergy steps
nstenergy                 = 50              ; write energies to energy file every
number of steps
nstxout-compressed        = 5000             ; write positions using compression
(saves memory, worse quality)
compressed-x-precision    = 1000            ; precision to write compressed trajectory

; next neighbor search and periodic boundary conditions
nstlist                   = 20              ; freq. to update neighbor list & long
range forces (>= 20 w/ GPUs)
rlist                     = 1.4             ; short-range neighbor list cutoff (nm)
cutoff-scheme             = Verlet          ; atom-based neighbor search with an
implicit buffer region
pbc                       = xyz             ; periodicity in x, y and z

; coulomb interaction
coulombtype               = PME             ; particle-mesh ewald summation for long
range (>rcoulomb)
rcoulomb                  = 1.4             ; short-range electrostatic cutoff (in nm)
(with PME,rcoulomb >= rvdw)
coulomb-modifier          = Potential-shift-Verlet ; shifts potential by constant so
potential is 0 at cut-off
fourierspacing            = 0.168           ; spacing of FFT in reciprocal space in
PME long range treatment (in nm)
pme-order                 = 4               ; cubic PME interpolation order

; lennard-jones potential handling
vdwtype                   = cutoff          ; simple truncation cutoff
vdw-modifier              = Potential-shift-Verlet ; shifts potential by constant so
potential is 0 at cut-off
rvdw                      = 1.4             ; short-range vdw cutoff (in nm)
```

```
DispCorr                 = EnerPres        ; long range correction for energy and
pressure from using vdw cutoff

; temperature coupling
tcoupl                   = v-rescale       ; the algorithm to use, v-rescale
generates correct canonical ensemble
tc-grps                  = System          ; groups to couple to temperature bath
tau-t                    = 1.0             ; time constant (in ps), meaning varies by
algorithm
ref-t                    = 328             ; temperature for coupling (K)
nsttcouple               = 1               ; frequency to couple temperature

; velocity generation
gen-vel                  = yes             ; generate velocities according to Maxwell
distr. (no for initial vel 0)
gen-temp                 = 328             ; temperature for Maxwell distribution (K)
gen-seed                 = -1              ; generate a random seed

; pressure coupling
pcoupl                   = Parrinello-Rahman  ; the algorithm to use (no for NVT)
pcoupltype               = isotropic       ; all ordinates scaled equally (preserves
the cubic box)
compressibility          = 4.5e-5          ; experimental value for water at 298 K
(in 1/bar)
tau-p                    = 5.0             ; time constant (in ps), should be about
4-5 times larger than tau_t
ref-p                    = 1               ; reference pressure (in bar)
nstpcouple               = 1               ; frequency to couple pressure

; constraints
constraints              = h-bonds         ; constrains bonds only involving hydrogen
constraint_algorithm     = lincs           ; algorithm to use, lincs should NOT be
used for angle constraining
lincs-order              = 4               ; highest order in constraint coupling
matrix expansion
lincs-iter               = 1               ; accuracy of lincs algorithm
continuation             = no              ; no for applying constraints at start of
run
```

For NVT production run “md_NVT_328K.mdp”

```
; very basics of the simulation
integrator               = md              ; solve newtown's equation of motion
dt                       = 0.002           ; integration time step / ps
nsteps                   = 150000000         ; number of steps (150000000 * 0.002 =
300 ns)

; remove drifts of the center of mass
comm-mode                = linear    ; remove COM translation
nstcomm                  = 10              ; number of steps for COM removal
comm-grps                = System          ; COM translation removed from system

; control frequency of output
nstvout                 = 0               ; write velocities to trajectory file every
number of steps
```

```
nstfout                 = 0               ; write forces to trajectory file every
number of steps
nstlog                   = 15000000            ; update log file every number of
steps
nstcalcenergy            = 5               ; calculate energies/pressures every
nstenergy steps
nstenergy                = 5               ; write energies to energy file every
number of steps
nstxout-compressed       = 30000             ; write positions using compression
(saves memory, worse quality)
compressed-x-precision   = 1000             ; precision to write compressed trajectory

; next neighbor search and periodic boundary conditions
nstlist                  = 20               ; freq. to update neighbor list & long
range forces (>= 20 w/ GPUs)
rlist                    = 1.4              ; short-range neighbor list cutoff (nm)
cutoff-scheme            = Verlet           ; atom-based neighbor search with an
implicit buffer region
pbc                      = xyz              ; periodicity in x, y and z

; coulomb interaction
coulombtype              = PME              ; particle-mesh ewald summation for long
range (>rcoulomb)
rcoulomb                 = 1.4              ; short-range electrostatic cutoff (in nm)
(with PME,rcoulomb >= rvdw)
coulomb-modifier         = Potential-shift-Verlet ; shifts potential by constant so
potential is 0 at cut-off
fourierspacing           = 0.168            ; spacing of FFT in reciprocal space in
PME long range treatment (in nm)
pme-order                = 4                ; cubic PME interpolation order

; lennard-jones potential handling
vdwtype                  = cutoff           ; simple truncation cutoff
vdw-modifier             = Potential-shift-Verlet ; shifts potential by constant so
potential is 0 at cut-off
rvdw                     = 1.4              ; short-range vdw cutoff (in nm)
DispCorr                 = EnerPres         ; long range correction for energy and
pressure from using vdw cutoff

; temperature coupling
tcoupl                   = v-rescale        ; the algorithm to use, v-rescale
generates correct canonical ensemble
tc-grps                  = System           ; groups to couple to temperature bath
tau-t                    = 1.0              ; time constant (in ps), meaning varies by
algorithm
ref-t                    = 328              ; temperature for coupling (K)
nsttcouple               = 1                ; frequency to couple temperature

; velocity generation
gen-vel                  = yes              ; generate velocities according to Maxwell
distr. (no for initial vel 0)
gen-temp                 = 328              ; temperature for Maxwell distribution (K)
gen-seed                 = -1               ; generate a random seed

; pressure coupling
pcoupl                   = no               ; the algorithm to use (no for NVT)
```

```
; constraints
constraints              = h-bonds        ; constrains bonds only involving hydrogen
constraint_algorithm     = lincs          ; algorithm to use, lincs should NOT be
used for angle constraining
lincs-order              = 4              ; highest order in constraint coupling
matrix expansion
lincs-iter               = 1              ; accuracy of lincs algorithm
continuation             = no             ; no for applying constraints at start of
run
```

4.2 Using modified OPLS forcefield

4.2.1 Topology files

“top_mod_mixtures.top” and “PolyEGs.itp” files are unchanged. Only the individual itp files are modified

Diethylene glycol

```
;
; GENERATED BY LigParGen Server
; Jorgensen Lab @ Yale University
; MODIFIED: 1/2 dihedral potential

[ moleculetype ]
; Name               nrexcl
DEG                   3
[ atoms ]
;   nr       type  resnr residue  atom   cgnr     charge       mass
     1   peg_OA         1    DEG  OAD1      1    -0.6885    15.9990
     2   peg_C          1    DEG  CD1       1     0.1082    12.0110
     3   peg_C          1    DEG  CD2       1     0.0069    12.0110
     4   peg_OE         1    DEG  OED1      1    -0.4007    15.9990
     5   peg_C          1    DEG  CD3       1    -0.0411    12.0110
     6   peg_C          1    DEG  CD4       1     0.1078    12.0110
     7   peg_OA         1    DEG  OAD2      1    -0.6862    15.9990
     8   peg_HA         1    DEG  HAD1      1     0.4182     1.0080
     9   peg_HC         1    DEG  HCD1      1      0.082     1.0080
    10   peg_HC         1    DEG  HCD2      1      0.082     1.0080
    11   peg_HC         1    DEG  HCD3      1     0.0927     1.0080
    12   peg_HC         1    DEG  HCD4      1     0.0927     1.0080
    13   peg_HC         1    DEG  HCD5      1     0.0947     1.0080
    14   peg_HC         1    DEG  HCD6      1     0.0947     1.0080
    15   peg_HC         1    DEG  HCD7      1     0.1066     1.0080
    16   peg_HC         1    DEG  HCD8      1     0.1066     1.0080
    17   peg_HA         1    DEG  HAD2      1     0.4234     1.0080
[ bonds ]
    2     1     1      0.1410 267776.000
    3     2     1      0.1529 224262.400
    4     3     1      0.1410 267776.000
    5     4     1      0.1410 267776.000
    6     5     1      0.1529 224262.400
    7     6     1      0.1410 267776.000
```

```
    8     1     1      0.0945 462750.400
    9     2     1      0.1090 284512.000
   10     2     1      0.1090 284512.000
   11     3     1      0.1090 284512.000
   12     3     1      0.1090 284512.000
   13     5     1      0.1090 284512.000
   14     5     1      0.1090 284512.000
   15     6     1      0.1090 284512.000
   16     6     1      0.1090 284512.000
   17     7     1      0.0945 462750.400

[ angles ]
;  ai    aj    ak funct            c0            c1            c2            c3
    1     2     3     1    109.500    418.400
    2     3     4     1    109.500    418.400
    3     4     5     1    109.500    502.080
    4     5     6     1    109.500    418.400
    5     6     7     1    109.500    418.400
    2     1     8     1    108.500    460.240
    1     2     9     1    109.500    292.880
    1     2    10     1    109.500    292.880
    2     3    11     1    110.700    313.800
    2     3    12     1    110.700    313.800
    4     5    13     1    109.500    292.880
    4     5    14     1    109.500    292.880
    5     6    15     1    110.700    313.800
    5     6    16     1    110.700    313.800
    6     7    17     1    108.500    460.240
    4     3    11     1    109.500    292.880
    9     2    10     1    107.800    276.144
   15     6    16     1    107.800    276.144
   13     5    14     1    107.800    276.144
    6     5    14     1    110.700    313.800
   11     3    12     1    107.800    276.144
    3     2     9     1    110.700    313.800
    6     5    13     1    110.700    313.800
    7     6    15     1    109.500    292.880
    7     6    16     1    109.500    292.880
    4     3    12     1    109.500    292.880
    3     2    10     1    110.700    313.800

[ dihedrals ]
; IMPROPER DIHEDRAL ANGLES
;  ai    aj    ak    al funct            c0            c1            c2            c3
c4            c5

[ dihedrals ]
; PROPER DIHEDRAL ANGLES
;  ai    aj    ak    al funct            c0            c1            c2            c3
c4            c5
    6    5    4    3        3       1.715   2.845   1.046  -5.607  -0.000   0.000
    5    4    3    2        3       1.715   2.845   1.046  -5.607  -0.000   0.000
   12    3    2    9        3       0.628   1.883   0.000  -2.510  -0.000   0.000
   16    6    5   13        3       0.628   1.883   0.000  -2.510  -0.000   0.000
   12    3    2   10        3       0.628   1.883   0.000  -2.510  -0.000   0.000
   16    6    5   14        3       0.628   1.883   0.000  -2.510  -0.000   0.000
   11    3    2   10        3       0.628   1.883   0.000  -2.510  -0.000   0.000
```

15	6	5	13	3	0.628	1.883	0.000	-2.510	-0.000	0.000
11	3	2	9	3	0.628	1.883	0.000	-2.510	-0.000	0.000
15	6	5	14	3	0.628	1.883	0.000	-2.510	-0.000	0.000
14	5	6	7	3	0.979	2.937	0.000	-3.916	-0.000	0.000
12	3	2	1	3	0.979	2.937	0.000	-3.916	-0.000	0.000
13	5	6	7	3	0.979	2.937	0.000	-3.916	-0.000	0.000
11	3	2	1	3	0.979	2.937	0.000	-3.916	-0.000	0.000
15	6	5	4	3	0.979	2.937	0.000	-3.916	-0.000	0.000
16	6	5	4	3	0.979	2.937	0.000	-3.916	-0.000	0.000
10	2	3	4	3	0.979	2.937	0.000	-3.916	-0.000	0.000
9	2	3	4	3	0.979	2.937	0.000	-3.916	-0.000	0.000
9	2	1	8	3	0.736	2.209	0.000	-2.946	-0.000	0.000
10	2	1	8	3	0.736	2.209	0.000	-2.946	-0.000	0.000
13	5	4	3	3	1.590	4.770	0.000	-6.360	-0.000	0.000
11	3	4	5	3	1.590	4.770	0.000	-6.360	-0.000	0.000
14	5	4	3	3	1.590	4.770	0.000	-6.360	-0.000	0.000
12	3	4	5	3	1.590	4.770	0.000	-6.360	-0.000	0.000
17	7	6	5	3	-0.444	3.833	0.728	-4.117	-0.000	0.000
8	1	2	3	3	-0.444	3.833	0.728	-4.117	-0.000	0.000
17	7	6	15	3	0.736	2.209	0.000	-2.946	-0.000	0.000
17	7	6	16	3	0.736	2.209	0.000	-2.946	-0.000	0.000
7	6	5	4	3	4.518	-4.518	0.000	-0.000	-0.000	0.000
4	3	2	1	3	4.518	-4.518	0.000	-0.000	-0.000	0.000

[pairs]

1	4	1
2	5	1
3	6	1
4	7	1
3	8	1
1	11	1
4	9	1
1	12	1
4	10	1
5	11	1
3	13	1
8	9	1
5	12	1
3	14	1
8	10	1
4	15	1
9	11	1
7	13	1
4	16	1
10	11	1
9	12	1
7	14	1
10	12	1
5	17	1
13	15	1
14	15	1
13	16	1
14	16	1
15	17	1
16	17	1

Triethylene glycol

```
;
; GENERATED BY LigParGen Server
; Jorgensen Lab @ Yale University
;MODIFIED: 1/2 dihedral potential

[ moleculetype ]
; Name               nrexcl
REG                   3
[ atoms ]
;   nr       type  resnr residue  atom   cgnr     charge       mass
     1   peg_OA        1    REG   OAR1      1    -0.6904    15.9990
     2   peg_C         1    REG   CR1       1     0.1087    12.0110
     3   peg_C         1    REG   CR2       1     0.0049    12.0110
     4   peg_OE        1    REG   OER1      1    -0.3783    15.9990
     5   peg_C         1    REG   CR3       1     0.0099    12.0110
     6   peg_C         1    REG   CR4       1     0.0105    12.0110
     7   peg_OE        1    REG   OER2      1    -0.3919    15.9990
     8   peg_C         1    REG   CR5       1    -0.0445    12.0110
     9   peg_C         1    REG   CR6       1     0.1087    12.0110
    10   peg_OA        1    REG   OAR2      1     -0.687    15.9990
    11   peg_HA        1    REG   HAR1      1     0.4169     1.0080
    12   peg_HC        1    REG   HCR1      1     0.0829     1.0080
    13   peg_HC        1    REG   HCR2      1     0.0829     1.0080
    14   peg_HC        1    REG   HCR3      1     0.0892     1.0080
    15   peg_HC        1    REG   HCR4      1     0.0892     1.0080
    16   peg_HC        1    REG   HCR5      1     0.0928     1.0080
    17   peg_HC        1    REG   HCR6      1     0.0928     1.0080
    18   peg_HC        1    REG   HCR7      1     0.0924     1.0080
    19   peg_HC        1    REG   HCR8      1     0.0924     1.0080
    20   peg_HC        1    REG   HCR9      1     0.0906     1.0080
    21   peg_HC        1    REG   HCRA      1     0.0906     1.0080
    22   peg_HC        1    REG   HCRB      1     0.1057     1.0080
    23   peg_HC        1    REG   HCRC      1     0.1057     1.0080
    24   peg_HA        1    REG   HAR2      1     0.4253     1.0080
[ bonds ]
    2     1     1      0.1410 267776.000
    3     2     1      0.1529 224262.400
    4     3     1      0.1410 267776.000
    5     4     1      0.1410 267776.000
    6     5     1      0.1529 224262.400
    7     6     1      0.1410 267776.000
    8     7     1      0.1410 267776.000
    9     8     1      0.1529 224262.400
   10     9     1      0.1410 267776.000
   11     1     1      0.0945 462750.400
   12     2     1      0.1090 284512.000
   13     2     1      0.1090 284512.000
   14     3     1      0.1090 284512.000
   15     3     1      0.1090 284512.000
   16     5     1      0.1090 284512.000
   17     5     1      0.1090 284512.000
   18     6     1      0.1090 284512.000
   19     6     1      0.1090 284512.000
   20     8     1      0.1090 284512.000
   21     8     1      0.1090 284512.000
```

```
   22     9     1      0.1090 284512.000
   23     9     1      0.1090 284512.000
   24    10     1      0.0945 462750.400

[ angles ]
;  ai    aj    ak funct             c0             c1             c2             c3
    1     2     3     1    109.500    418.400
    2     3     4     1    109.500    418.400
    3     4     5     1    109.500    502.080
    4     5     6     1    109.500    418.400
    5     6     7     1    109.500    418.400
    6     7     8     1    109.500    502.080
    7     8     9     1    109.500    418.400
    8     9    10     1    109.500    418.400
    2     1    11     1    108.500    460.240
    1     2    12     1    109.500    292.880
    1     2    13     1    109.500    292.880
    2     3    14     1    110.700    313.800
    2     3    15     1    110.700    313.800
    4     5    16     1    109.500    292.880
    4     5    17     1    109.500    292.880
    5     6    18     1    110.700    313.800
    5     6    19     1    110.700    313.800
    7     8    20     1    109.500    292.880
    7     8    21     1    109.500    292.880
    8     9    22     1    110.700    313.800
    8     9    23     1    110.700    313.800
    9    10    24     1    108.500    460.240
    7     6    18     1    109.500    292.880
   12     2    13     1    107.800    276.144
   18     6    19     1    107.800    276.144
    9     8    20     1    110.700    313.800
   16     5    17     1    107.800    276.144
    6     5    17     1    110.700    313.800
   10     9    23     1    109.500    292.880
   22     9    23     1    107.800    276.144
    7     6    19     1    109.500    292.880
   14     3    15     1    107.800    276.144
   10     9    22     1    109.500    292.880
    3     2    12     1    110.700    313.800
   20     8    21     1    107.800    276.144
    6     5    16     1    110.700    313.800
    3     2    13     1    110.700    313.800
    4     3    14     1    109.500    292.880
    9     8    21     1    110.700    313.800
    4     3    15     1    109.500    292.880

[ dihedrals ]
; IMPROPER DIHEDRAL ANGLES
;  ai    aj    ak    al funct             c0             c1             c2             c3
c4             c5

[ dihedrals ]
; PROPER DIHEDRAL ANGLES
;  ai    aj    ak    al funct             c0             c1             c2             c3
c4             c5
    6    5    4    3        3      1.715   2.845   1.046  -5.607  -0.000   0.000
```

9	8	7	6	3	1.715	2.845	1.046	-5.607	-0.000	0.000
5	4	3	2	3	1.715	2.845	1.046	-5.607	-0.000	0.000
8	7	6	5	3	1.715	2.845	1.046	-5.607	-0.000	0.000
14	3	2	12	3	0.628	1.883	0.000	-2.510	-0.000	0.000
23	9	8	20	3	0.628	1.883	0.000	-2.510	-0.000	0.000
22	9	8	21	3	0.628	1.883	0.000	-2.510	-0.000	0.000
18	6	5	16	3	0.628	1.883	0.000	-2.510	-0.000	0.000
15	3	2	12	3	0.628	1.883	0.000	-2.510	-0.000	0.000
14	3	2	13	3	0.628	1.883	0.000	-2.510	-0.000	0.000
23	9	8	21	3	0.628	1.883	0.000	-2.510	-0.000	0.000
22	9	8	20	3	0.628	1.883	0.000	-2.510	-0.000	0.000
19	6	5	17	3	0.628	1.883	0.000	-2.510	-0.000	0.000
18	6	5	17	3	0.628	1.883	0.000	-2.510	-0.000	0.000
15	3	2	13	3	0.628	1.883	0.000	-2.510	-0.000	0.000
19	6	5	16	3	0.628	1.883	0.000	-2.510	-0.000	0.000
21	8	9	10	3	0.979	2.937	0.000	-3.916	-0.000	0.000
14	3	2	1	3	0.979	2.937	0.000	-3.916	-0.000	0.000
20	8	9	10	3	0.979	2.937	0.000	-3.916	-0.000	0.000
15	3	2	1	3	0.979	2.937	0.000	-3.916	-0.000	0.000
23	9	8	7	3	0.979	2.937	0.000	-3.916	-0.000	0.000
18	6	5	4	3	0.979	2.937	0.000	-3.916	-0.000	0.000
13	2	3	4	3	0.979	2.937	0.000	-3.916	-0.000	0.000
19	6	5	4	3	0.979	2.937	0.000	-3.916	-0.000	0.000
16	5	6	7	3	0.979	2.937	0.000	-3.916	-0.000	0.000
17	5	6	7	3	0.979	2.937	0.000	-3.916	-0.000	0.000
22	9	8	7	3	0.979	2.937	0.000	-3.916	-0.000	0.000
12	2	3	4	3	0.979	2.937	0.000	-3.916	-0.000	0.000
12	2	1	11	3	0.736	2.209	0.000	-2.946	-0.000	0.000
13	2	1	11	3	0.736	2.209	0.000	-2.946	-0.000	0.000
19	6	7	8	3	1.590	4.770	0.000	-6.360	-0.000	0.000
20	8	7	6	3	1.590	4.770	0.000	-6.360	-0.000	0.000
18	6	7	8	3	1.590	4.770	0.000	-6.360	-0.000	0.000
16	5	4	3	3	1.590	4.770	0.000	-6.360	-0.000	0.000
17	5	4	3	3	1.590	4.770	0.000	-6.360	-0.000	0.000
14	3	4	5	3	1.590	4.770	0.000	-6.360	-0.000	0.000
15	3	4	5	3	1.590	4.770	0.000	-6.360	-0.000	0.000
21	8	7	6	3	1.590	4.770	0.000	-6.360	-0.000	0.000
24	10	9	8	3	-0.444	3.833	0.728	-4.117	-0.000	0.000
11	1	2	3	3	-0.444	3.833	0.728	-4.117	-0.000	0.000
24	10	9	22	3	0.736	2.209	0.000	-2.946	-0.000	0.000
24	10	9	23	3	0.736	2.209	0.000	-2.946	-0.000	0.000
10	9	8	7	3	4.518	-4.518	0.000	-0.000	-0.000	0.000
4	3	2	1	3	4.518	-4.518	0.000	-0.000	-0.000	0.000
7	6	5	4	3	-1.151	1.151	0.000	-0.000	-0.000	0.000

[pairs]

1	4	1
2	5	1
3	6	1
4	7	1
5	8	1
3	11	1
6	9	1
1	14	1
4	12	1
1	15	1
7	10	1

```
     4    13    1
     5    14    1
     3    16    1
     5    15    1
     3    17    1
     4    18    1
    11    12    1
     7    16    1
     4    19    1
    11    13    1
     7    17    1
    12    14    1
     8    18    1
     6    20    1
    13    14    1
    12    15    1
     8    19    1
     6    21    1
    13    15    1
     7    22    1
    10    20    1
     7    23    1
    10    21    1
     8    24    1
    16    18    1
    17    18    1
    16    19    1
    17    19    1
    20    22    1
    21    22    1
    20    23    1
    21    23    1
    22    24    1
    23    24    1
```

Tetraethylene glycol

```
;
; GENERATED BY LigParGen Server
; Jorgensen Lab @ Yale University
; MODIFIED: 1/4 dihedral potential

[ moleculetype ]
; Name               nrexcl
TEG                   3
[ atoms ]
;   nr       type  resnr residue  atom   cgnr     charge       mass
     1   peg_OA        1    TEG   OAT1      1    -0.6887    15.9990
     2   peg_C         1    TEG   CT1       1     0.1087    12.0110
     3   peg_C         1    TEG   CT2       1     0.0083    12.0110
     4   peg_OE        1    TEG   OET1      1    -0.3851    15.9990
     5   peg_C         1    TEG   CT3       1     0.0083    12.0110
     6   peg_C         1    TEG   CT4       1     0.0088    12.0110
     7   peg_OE        1    TEG   OET2      1    -0.3849    15.9990
     8   peg_C         1    TEG   CT5       1     0.0089    12.0110
     9   peg_C         1    TEG   CT6       1     0.0068    12.0110
```

```
    10   peg_OE        1    TEG   OET3      1    -0.3989    15.9990
    11   peg_C         1    TEG   CT7       1    -0.0408    12.0110
    12   peg_C         1    TEG   CT8       1     0.1081    12.0110
    13   peg_OA        1    TEG   OAT2      1    -0.6858    15.9990
    14   peg_HA        1    TEG   HAT1      1     0.4177     1.0080
    15   peg_HC        1    TEG   HCT1      1     0.0822     1.0080
    16   peg_HC        1    TEG   HCT2      1     0.0822     1.0080
    17   peg_HC        1    TEG   HCT3      1     0.0925     1.0080
    18   peg_HC        1    TEG   HCT4      1     0.0925     1.0080
    19   peg_HC        1    TEG   HCT5      1     0.0916     1.0080
    20   peg_HC        1    TEG   HCT6      1     0.0916     1.0080
    21   peg_HC        1    TEG   HCT7      1     0.0917     1.0080
    22   peg_HC        1    TEG   HCT8      1     0.0917     1.0080
    23   peg_HC        1    TEG   HCT9      1     0.0913     1.0080
    24   peg_HC        1    TEG   HCTA      1     0.0913     1.0080
    25   peg_HC        1    TEG   HCTB      1     0.0919     1.0080
    26   peg_HC        1    TEG   HCTC      1     0.0919     1.0080
    27   peg_HC        1    TEG   HCTD      1     0.0946     1.0080
    28   peg_HC        1    TEG   HCTE      1     0.0946     1.0080
    29   peg_HC        1    TEG   HCTF      1     0.1068     1.0080
    30   peg_HC        1    TEG   HCTG      1     0.1068     1.0080
    31   peg_HA        1    TEG   HAT2      1     0.4234     1.0080
[ bonds ]
    2     1     1      0.1410 267776.000
    3     2     1      0.1529 224262.400
    4     3     1      0.1410 267776.000
    5     4     1      0.1410 267776.000
    6     5     1      0.1529 224262.400
    7     6     1      0.1410 267776.000
    8     7     1      0.1410 267776.000
    9     8     1      0.1529 224262.400
   10     9     1      0.1410 267776.000
   11    10     1      0.1410 267776.000
   12    11     1      0.1529 224262.400
   13    12     1      0.1410 267776.000
   14     1     1      0.0945 462750.400
   15     2     1      0.1090 284512.000
   16     2     1      0.1090 284512.000
   17     3     1      0.1090 284512.000
   18     3     1      0.1090 284512.000
   19     5     1      0.1090 284512.000
   20     5     1      0.1090 284512.000
   21     6     1      0.1090 284512.000
   22     6     1      0.1090 284512.000
   23     8     1      0.1090 284512.000
   24     8     1      0.1090 284512.000
   25     9     1      0.1090 284512.000
   26     9     1      0.1090 284512.000
   27    11     1      0.1090 284512.000
   28    11     1      0.1090 284512.000
   29    12     1      0.1090 284512.000
   30    12     1      0.1090 284512.000
   31    13     1      0.0945 462750.400

[ angles ]
;  ai    aj    ak funct            c0            c1            c2            c3
    1     2     3     1    109.500    418.400
```

```
    2     3     4     1    109.500    418.400
    3     4     5     1    109.500    502.080
    4     5     6     1    109.500    418.400
    5     6     7     1    109.500    418.400
    6     7     8     1    109.500    502.080
    7     8     9     1    109.500    418.400
    8     9    10     1    109.500    418.400
    9    10    11     1    109.500    502.080
   10    11    12     1    109.500    418.400
   11    12    13     1    109.500    418.400
    2     1    14     1    108.500    460.240
    1     2    15     1    109.500    292.880
    1     2    16     1    109.500    292.880
    2     3    17     1    110.700    313.800
    2     3    18     1    110.700    313.800
    4     5    19     1    109.500    292.880
    4     5    20     1    109.500    292.880
    5     6    21     1    110.700    313.800
    5     6    22     1    110.700    313.800
    7     8    23     1    109.500    292.880
    7     8    24     1    109.500    292.880
    8     9    25     1    110.700    313.800
    8     9    26     1    110.700    313.800
   10    11    27     1    109.500    292.880
   10    11    28     1    109.500    292.880
   11    12    29     1    110.700    313.800
   11    12    30     1    110.700    313.800
   12    13    31     1    108.500    460.240
   25     9    26     1    107.800    276.144
   23     8    24     1    107.800    276.144
    3     2    15     1    110.700    313.800
   29    12    30     1    107.800    276.144
   12    11    27     1    110.700    313.800
   15     2    16     1    107.800    276.144
    7     6    22     1    109.500    292.880
   21     6    22     1    107.800    276.144
   27    11    28     1    107.800    276.144
    6     5    19     1    110.700    313.800
    9     8    23     1    110.700    313.800
   13    12    29     1    109.500    292.880
    3     2    16     1    110.700    313.800
    4     3    18     1    109.500    292.880
    9     8    24     1    110.700    313.800
   19     5    20     1    107.800    276.144
   13    12    30     1    109.500    292.880
    6     5    20     1    110.700    313.800
   10     9    25     1    109.500    292.880
   10     9    26     1    109.500    292.880
    4     3    17     1    109.500    292.880
   17     3    18     1    107.800    276.144
   12    11    28     1    110.700    313.800
    7     6    21     1    109.500    292.880

[ dihedrals ]
; IMPROPER DIHEDRAL ANGLES
;  ai    aj    ak    al  funct            c0            c1            c2            c3
c4            c5
```

```
[ dihedrals ]
; PROPER DIHEDRAL ANGLES
;  ai    aj    ak    al funct            c0            c1            c2            c3
c4            c5
    6     5     4     3         3       1.715    2.845    1.046   -5.607  -0.000   0.000
    9     8     7     6         3       1.715    2.845    1.046   -5.607  -0.000   0.000
   12    11    10     9         3       1.715    2.845    1.046   -5.607  -0.000   0.000
    5     4     3     2         3       1.715    2.845    1.046   -5.607  -0.000   0.000
    8     7     6     5         3       1.715    2.845    1.046   -5.607  -0.000   0.000
   11    10     9     8         3       1.715    2.845    1.046   -5.607  -0.000   0.000
   18     3     2    15         3       0.628    1.883    0.000   -2.510  -0.000   0.000
   18     3     2    16         3       0.628    1.883    0.000   -2.510  -0.000   0.000
   21     6     5    20         3       0.628    1.883    0.000   -2.510  -0.000   0.000
   21     6     5    19         3       0.628    1.883    0.000   -2.510  -0.000   0.000
   22     6     5    19         3       0.628    1.883    0.000   -2.510  -0.000   0.000
   29    12    11    28         3       0.628    1.883    0.000   -2.510  -0.000   0.000
   30    12    11    28         3       0.628    1.883    0.000   -2.510  -0.000   0.000
   17     3     2    16         3       0.628    1.883    0.000   -2.510  -0.000   0.000
   22     6     5    20         3       0.628    1.883    0.000   -2.510  -0.000   0.000
   30    12    11    27         3       0.628    1.883    0.000   -2.510  -0.000   0.000
   17     3     2    15         3       0.628    1.883    0.000   -2.510  -0.000   0.000
   26     9     8    24         3       0.628    1.883    0.000   -2.510  -0.000   0.000
   26     9     8    23         3       0.628    1.883    0.000   -2.510  -0.000   0.000
   29    12    11    27         3       0.628    1.883    0.000   -2.510  -0.000   0.000
   25     9     8    23         3       0.628    1.883    0.000   -2.510  -0.000   0.000
   25     9     8    24         3       0.628    1.883    0.000   -2.510  -0.000   0.000
   28    11    12    13         3       0.979    2.937    0.000   -3.916  -0.000   0.000
   18     3     2     1         3       0.979    2.937    0.000   -3.916  -0.000   0.000
   27    11    12    13         3       0.979    2.937    0.000   -3.916  -0.000   0.000
   17     3     2     1         3       0.979    2.937    0.000   -3.916  -0.000   0.000
   19     5     6     7         3       0.979    2.937    0.000   -3.916  -0.000   0.000
   20     5     6     7         3       0.979    2.937    0.000   -3.916  -0.000   0.000
   30    12    11    10         3       0.979    2.937    0.000   -3.916  -0.000   0.000
   26     9     8     7         3       0.979    2.937    0.000   -3.916  -0.000   0.000
   24     8     9    10         3       0.979    2.937    0.000   -3.916  -0.000   0.000
   22     6     5     4         3       0.979    2.937    0.000   -3.916  -0.000   0.000
   23     8     9    10         3       0.979    2.937    0.000   -3.916  -0.000   0.000
   29    12    11    10         3       0.979    2.937    0.000   -3.916  -0.000   0.000
   21     6     5     4         3       0.979    2.937    0.000   -3.916  -0.000   0.000
   25     9     8     7         3       0.979    2.937    0.000   -3.916  -0.000   0.000
   15     2     3     4         3       0.979    2.937    0.000   -3.916  -0.000   0.000
   16     2     3     4         3       0.979    2.937    0.000   -3.916  -0.000   0.000
   16     2     1    14         3       0.736    2.209    0.000   -2.946  -0.000   0.000
   15     2     1    14         3       0.736    2.209    0.000   -2.946  -0.000   0.000
   21     6     7     8         3       1.590    4.770    0.000   -6.360  -0.000   0.000
   24     8     7     6         3       1.590    4.770    0.000   -6.360  -0.000   0.000
   22     6     7     8         3       1.590    4.770    0.000   -6.360  -0.000   0.000
   28    11    10     9         3       1.590    4.770    0.000   -6.360  -0.000   0.000
   26     9    10    11         3       1.590    4.770    0.000   -6.360  -0.000   0.000
   19     5     4     3         3       1.590    4.770    0.000   -6.360  -0.000   0.000
   25     9    10    11         3       1.590    4.770    0.000   -6.360  -0.000   0.000
   17     3     4     5         3       1.590    4.770    0.000   -6.360  -0.000   0.000
   20     5     4     3         3       1.590    4.770    0.000   -6.360  -0.000   0.000
   23     8     7     6         3       1.590    4.770    0.000   -6.360  -0.000   0.000
   18     3     4     5         3       1.590    4.770    0.000   -6.360  -0.000   0.000
   27    11    10     9         3       1.590    4.770    0.000   -6.360  -0.000   0.000
```

```
   31   13   12   11        3      -0.444   3.833   0.728  -4.117  -0.000   0.000
   14    1    2    3        3      -0.444   3.833   0.728  -4.117  -0.000   0.000
   31   13   12   30        3       0.736   2.209   0.000  -2.946  -0.000   0.000
   31   13   12   29        3       0.736   2.209   0.000  -2.946  -0.000   0.000
   13   12   11   10        3       2.259  -2.259   0.000  -0.000  -0.000   0.000
    4    3    2    1        3       2.259  -2.259   0.000  -0.000  -0.000   0.000
    7    6    5    4        3      -1.151   1.151   0.000  -0.000  -0.000   0.000
   10    9    8    7        3      -1.151   1.151   0.000  -0.000  -0.000   0.000

[ pairs ]
     1     4    1
     2     5    1
     3     6    1
     4     7    1
     5     8    1
     6     9    1
     7    10    1
     3    14    1
     1    17    1
     8    11    1
     4    15    1
     1    18    1
     4    16    1
     9    12    1
     5    17    1
     3    19    1
    10    13    1
     5    18    1
     3    20    1
     4    21    1
     7    19    1
     4    22    1
     7    20    1
    14    15    1
     8    21    1
     6    23    1
    14    16    1
     8    22    1
     6    24    1
    15    17    1
     7    25    1
    16    17    1
    15    18    1
    10    23    1
     7    26    1
    16    18    1
    10    24    1
    11    25    1
     9    27    1
    11    26    1
     9    28    1
    10    29    1
    19    21    1
    13    27    1
    10    30    1
    20    21    1
    19    22    1
```

```
    13    28    1
    20    22    1
    11    31    1
    23    25    1
    24    25    1
    23    26    1
    24    26    1
    27    29    1
    28    29    1
    27    30    1
    28    30    1
    29    31    1
    30    31    1
```

Pentaethylene glycol

```
;
; GENERATED BY LigParGen Server
; Jorgensen Lab @ Yale University
; MODIFIED: 1/4 dihedral potential

[ moleculetype ]
; Name               nrexcl
PEG                   3
[ atoms ]
;   nr       type  resnr residue  atom   cgnr     charge       mass
     1   peg_OA        1    PEG   OAP1      1    -0.6888    15.9990
     2   peg_C         1    PEG   CP1       1     0.1083    12.0110
     3   peg_C         1    PEG   CP2       1     0.0083    12.0110
     4   peg_OE        1    PEG   OEP1      1    -0.3868    15.9990
     5   peg_C         1    PEG   CP3       1     0.0083    12.0110
     6   peg_C         1    PEG   CP4       1     0.0054    12.0110
     7   peg_OE        1    PEG   OEP2      1    -0.3768    15.9990
     8   peg_C         1    PEG   CP5       1     0.0112    12.0110
     9   peg_C         1    PEG   CP6       1      0.011    12.0110
    10   peg_OE        1    PEG   OEP3      1    -0.3767    15.9990
    11   peg_C         1    PEG   CP7       1     0.0054    12.0110
    12   peg_C         1    PEG   CP8       1     0.0071    12.0110
    13   peg_OE        1    PEG   OEP4      1    -0.4008    15.9990
    14   peg_C         1    PEG   CP9       1    -0.0409    12.0110
    15   peg_C         1    PEG   CPA       1      0.108    12.0110
    16   peg_OA        1    PEG   OAP2      1    -0.6862    15.9990
    17   peg_HA        1    PEG   HAP1      1     0.4172     1.0080
    18   peg_HC        1    PEG   HCP1      1     0.0815     1.0080
    19   peg_HC        1    PEG   HCP2      1     0.0815     1.0080
    20   peg_HC        1    PEG   HCP3      1     0.0924     1.0080
    21   peg_HC        1    PEG   HCP4      1     0.0924     1.0080
    22   peg_HC        1    PEG   HCP5      1     0.0926     1.0080
    23   peg_HC        1    PEG   HCP6      1     0.0926     1.0080
    24   peg_HC        1    PEG   HCP7      1     0.0878     1.0080
    25   peg_HC        1    PEG   HCP8      1     0.0878     1.0080
    26   peg_HC        1    PEG   HCP9      1     0.0929     1.0080
    27   peg_HC        1    PEG   HCPA      1     0.0929     1.0080
    28   peg_HC        1    PEG   HCPB      1     0.0929     1.0080
    29   peg_HC        1    PEG   HCPC      1     0.0929     1.0080
    30   peg_HC        1    PEG   HCPD      1     0.0878     1.0080
```

```
    31   peg_HC        1    PEG   HCPE      1     0.0878     1.0080
    32   peg_HC        1    PEG   HCPF      1      0.093     1.0080
    33   peg_HC        1    PEG   HCPG      2      0.093     1.0080
    34   peg_HC        1    PEG   HCPH      2     0.0945     1.0080
    35   peg_HC        1    PEG   HCPI      2     0.0945     1.0080
    36   peg_HC        1    PEG   HCPJ      2     0.1062     1.0080
    37   peg_HC        1    PEG   HCPK      2     0.1062     1.0080
    38   peg_HA        1    PEG   HAP2      2     0.4236     1.0080
[ bonds ]
    2     1     1      0.1410 267776.000
    3     2     1      0.1529 224262.400
    4     3     1      0.1410 267776.000
    5     4     1      0.1410 267776.000
    6     5     1      0.1529 224262.400
    7     6     1      0.1410 267776.000
    8     7     1      0.1410 267776.000
    9     8     1      0.1529 224262.400
   10     9     1      0.1410 267776.000
   11    10     1      0.1410 267776.000
   12    11     1      0.1529 224262.400
   13    12     1      0.1410 267776.000
   14    13     1      0.1410 267776.000
   15    14     1      0.1529 224262.400
   16    15     1      0.1410 267776.000
   17     1     1      0.0945 462750.400
   18     2     1      0.1090 284512.000
   19     2     1      0.1090 284512.000
   20     3     1      0.1090 284512.000
   21     3     1      0.1090 284512.000
   22     5     1      0.1090 284512.000
   23     5     1      0.1090 284512.000
   24     6     1      0.1090 284512.000
   25     6     1      0.1090 284512.000
   26     8     1      0.1090 284512.000
   27     8     1      0.1090 284512.000
   28     9     1      0.1090 284512.000
   29     9     1      0.1090 284512.000
   30    11     1      0.1090 284512.000
   31    11     1      0.1090 284512.000
   32    12     1      0.1090 284512.000
   33    12     1      0.1090 284512.000
   34    14     1      0.1090 284512.000
   35    14     1      0.1090 284512.000
   36    15     1      0.1090 284512.000
   37    15     1      0.1090 284512.000
   38    16     1      0.0945 462750.400

[ angles ]
;  ai    aj    ak funct            c0            c1            c2            c3
    1     2     3     1    109.500    418.400
    2     3     4     1    109.500    418.400
    3     4     5     1    109.500    502.080
    4     5     6     1    109.500    418.400
    5     6     7     1    109.500    418.400
    6     7     8     1    109.500    502.080
    7     8     9     1    109.500    418.400
    8     9    10     1    109.500    418.400
```

9	10	11	1	109.500	502.080
10	11	12	1	109.500	418.400
11	12	13	1	109.500	418.400
12	13	14	1	109.500	502.080
13	14	15	1	109.500	418.400
14	15	16	1	109.500	418.400
2	1	17	1	108.500	460.240
1	2	18	1	109.500	292.880
1	2	19	1	109.500	292.880
2	3	20	1	110.700	313.800
2	3	21	1	110.700	313.800
4	5	22	1	109.500	292.880
4	5	23	1	109.500	292.880
5	6	24	1	110.700	313.800
5	6	25	1	110.700	313.800
7	8	26	1	109.500	292.880
7	8	27	1	109.500	292.880
8	9	28	1	110.700	313.800
8	9	29	1	110.700	313.800
10	11	30	1	109.500	292.880
10	11	31	1	109.500	292.880
11	12	32	1	110.700	313.800
11	12	33	1	110.700	313.800
13	14	34	1	109.500	292.880
13	14	35	1	109.500	292.880
14	15	36	1	110.700	313.800
14	15	37	1	110.700	313.800
15	16	38	1	108.500	460.240
15	14	34	1	110.700	313.800
12	11	31	1	110.700	313.800
15	14	35	1	110.700	313.800
7	6	24	1	109.500	292.880
13	12	33	1	109.500	292.880
22	5	23	1	107.800	276.144
3	2	18	1	110.700	313.800
10	9	29	1	109.500	292.880
6	5	22	1	110.700	313.800
16	15	36	1	109.500	292.880
7	6	25	1	109.500	292.880
32	12	33	1	107.800	276.144
18	2	19	1	107.800	276.144
34	14	35	1	107.800	276.144
12	11	30	1	110.700	313.800
30	11	31	1	107.800	276.144
9	8	27	1	110.700	313.800
36	15	37	1	107.800	276.144
3	2	19	1	110.700	313.800
4	3	21	1	109.500	292.880
16	15	37	1	109.500	292.880
28	9	29	1	107.800	276.144
24	6	25	1	107.800	276.144
6	5	23	1	110.700	313.800
9	8	26	1	110.700	313.800
10	9	28	1	109.500	292.880
13	12	32	1	109.500	292.880
4	3	20	1	109.500	292.880
26	8	27	1	107.800	276.144

```
   20     3    21     1    107.800    276.144

[ dihedrals ]
; IMPROPER DIHEDRAL ANGLES
;  ai    aj    ak    al funct            c0            c1            c2            c3
c4            c5

[ dihedrals ]
; PROPER DIHEDRAL ANGLES
;  ai    aj    ak    al funct            c0            c1            c2            c3
c4            c5
   12   11   10    9        3       1.715   2.845   1.046  -5.607  -0.000   0.000
    6    5    4    3        3       1.715   2.845   1.046  -5.607  -0.000   0.000
    9    8    7    6        3       1.715   2.845   1.046  -5.607  -0.000   0.000
   15   14   13   12        3       1.715   2.845   1.046  -5.607  -0.000   0.000
    5    4    3    2        3       1.715   2.845   1.046  -5.607  -0.000   0.000
    8    7    6    5        3       1.715   2.845   1.046  -5.607  -0.000   0.000
   11   10    9    8        3       1.715   2.845   1.046  -5.607  -0.000   0.000
   14   13   12   11        3       1.715   2.845   1.046  -5.607  -0.000   0.000
   25    6    5   22        3       0.628   1.883   0.000  -2.510  -0.000   0.000
   28    9    8   27        3       0.628   1.883   0.000  -2.510  -0.000   0.000
   29    9    8   27        3       0.628   1.883   0.000  -2.510  -0.000   0.000
   33   12   11   31        3       0.628   1.883   0.000  -2.510  -0.000   0.000
   29    9    8   26        3       0.628   1.883   0.000  -2.510  -0.000   0.000
   20    3    2   18        3       0.628   1.883   0.000  -2.510  -0.000   0.000
   24    6    5   22        3       0.628   1.883   0.000  -2.510  -0.000   0.000
   36   15   14   34        3       0.628   1.883   0.000  -2.510  -0.000   0.000
   36   15   14   35        3       0.628   1.883   0.000  -2.510  -0.000   0.000
   33   12   11   30        3       0.628   1.883   0.000  -2.510  -0.000   0.000
   21    3    2   19        3       0.628   1.883   0.000  -2.510  -0.000   0.000
   20    3    2   19        3       0.628   1.883   0.000  -2.510  -0.000   0.000
   37   15   14   34        3       0.628   1.883   0.000  -2.510  -0.000   0.000
   37   15   14   35        3       0.628   1.883   0.000  -2.510  -0.000   0.000
   21    3    2   18        3       0.628   1.883   0.000  -2.510  -0.000   0.000
   32   12   11   30        3       0.628   1.883   0.000  -2.510  -0.000   0.000
   24    6    5   23        3       0.628   1.883   0.000  -2.510  -0.000   0.000
   32   12   11   31        3       0.628   1.883   0.000  -2.510  -0.000   0.000
   25    6    5   23        3       0.628   1.883   0.000  -2.510  -0.000   0.000
   28    9    8   26        3       0.628   1.883   0.000  -2.510  -0.000   0.000
   34   14   15   16        3       0.979   2.937   0.000  -3.916  -0.000   0.000
   35   14   15   16        3       0.979   2.937   0.000  -3.916  -0.000   0.000
   20    3    2    1        3       0.979   2.937   0.000  -3.916  -0.000   0.000
   21    3    2    1        3       0.979   2.937   0.000  -3.916  -0.000   0.000
   19    2    3    4        3       0.979   2.937   0.000  -3.916  -0.000   0.000
   28    9    8    7        3       0.979   2.937   0.000  -3.916  -0.000   0.000
   30   11   12   13        3       0.979   2.937   0.000  -3.916  -0.000   0.000
   22    5    6    7        3       0.979   2.937   0.000  -3.916  -0.000   0.000
   33   12   11   10        3       0.979   2.937   0.000  -3.916  -0.000   0.000
   31   11   12   13        3       0.979   2.937   0.000  -3.916  -0.000   0.000
   24    6    5    4        3       0.979   2.937   0.000  -3.916  -0.000   0.000
   37   15   14   13        3       0.979   2.937   0.000  -3.916  -0.000   0.000
   36   15   14   13        3       0.979   2.937   0.000  -3.916  -0.000   0.000
   32   12   11   10        3       0.979   2.937   0.000  -3.916  -0.000   0.000
   23    5    6    7        3       0.979   2.937   0.000  -3.916  -0.000   0.000
   26    8    9   10        3       0.979   2.937   0.000  -3.916  -0.000   0.000
   18    2    3    4        3       0.979   2.937   0.000  -3.916  -0.000   0.000
   27    8    9   10        3       0.979   2.937   0.000  -3.916  -0.000   0.000
```

```
   29    9    8    7         3       0.979   2.937   0.000  -3.916  -0.000   0.000
   25    6    5    4         3       0.979   2.937   0.000  -3.916  -0.000   0.000
   18    2    1   17         3       0.736   2.209   0.000  -2.946  -0.000   0.000
   19    2    1   17         3       0.736   2.209   0.000  -2.946  -0.000   0.000
   25    6    7    8         3       1.590   4.770   0.000  -6.360  -0.000   0.000
   35   14   13   12         3       1.590   4.770   0.000  -6.360  -0.000   0.000
   33   12   13   14         3       1.590   4.770   0.000  -6.360  -0.000   0.000
   31   11   10    9         3       1.590   4.770   0.000  -6.360  -0.000   0.000
   22    5    4    3         3       1.590   4.770   0.000  -6.360  -0.000   0.000
   28    9   10   11         3       1.590   4.770   0.000  -6.360  -0.000   0.000
   27    8    7    6         3       1.590   4.770   0.000  -6.360  -0.000   0.000
   24    6    7    8         3       1.590   4.770   0.000  -6.360  -0.000   0.000
   34   14   13   12         3       1.590   4.770   0.000  -6.360  -0.000   0.000
   23    5    4    3         3       1.590   4.770   0.000  -6.360  -0.000   0.000
   26    8    7    6         3       1.590   4.770   0.000  -6.360  -0.000   0.000
   32   12   13   14         3       1.590   4.770   0.000  -6.360  -0.000   0.000
   21    3    4    5         3       1.590   4.770   0.000  -6.360  -0.000   0.000
   29    9   10   11         3       1.590   4.770   0.000  -6.360  -0.000   0.000
   20    3    4    5         3       1.590   4.770   0.000  -6.360  -0.000   0.000
   30   11   10    9         3       1.590   4.770   0.000  -6.360  -0.000   0.000
   38   16   15   14         3      -0.444   3.833   0.728  -4.117  -0.000   0.000
   17    1    2    3         3      -0.444   3.833   0.728  -4.117  -0.000   0.000
   38   16   15   37         3       0.736   2.209   0.000  -2.946  -0.000   0.000
   38   16   15   36         3       0.736   2.209   0.000  -2.946  -0.000   0.000
   16   15   14   13         3       2.259  -2.259   0.000  -0.000  -0.000   0.000
    4    3    2    1         3       2.259  -2.259   0.000  -0.000  -0.000   0.000
   13   12   11   10         3      -1.151   1.151   0.000  -0.000  -0.000   0.000
   10    9    8    7         3      -1.151   1.151   0.000  -0.000  -0.000   0.000
    7    6    5    4         3      -1.151   1.151   0.000  -0.000  -0.000   0.000

[ pairs ]
     1     4    1
     2     5    1
     3     6    1
     4     7    1
     5     8    1
     6     9    1
     7    10    1
     8    11    1
     3    17    1
     9    12    1
     1    20    1
     4    18    1
     1    21    1
    10    13    1
     4    19    1
    11    14    1
     5    20    1
     3    22    1
     5    21    1
     3    23    1
    12    15    1
     4    24    1
    13    16    1
     7    22    1
     4    25    1
     7    23    1
```

```
     8    24    1
     6    26    1
     8    25    1
     6    27    1
    17    18    1
     7    28    1
    17    19    1
    10    26    1
     7    29    1
    10    27    1
    18    20    1
    19    20    1
    18    21    1
    11    28    1
     9    30    1
    19    21    1
    11    29    1
     9    31    1
    10    32    1
    13    30    1
    10    33    1
    13    31    1
    22    24    1
    14    32    1
    12    34    1
    23    24    1
    22    25    1
    14    33    1
    12    35    1
    23    25    1
    13    36    1
    16    34    1
    13    37    1
    16    35    1
    14    38    1
    26    28    1
    27    28    1
    26    29    1
    27    29    1
    30    32    1
    31    32    1
    30    33    1
    31    33    1
    34    36    1
    35    36    1
    34    37    1
    35    37    1
    36    38    1
    37    38    1
```

Hexaethylene Glycol

```
;
; GENERATED BY LigParGen Server
; Jorgensen Lab @ Yale University
; MODIFIED: 1/2 dihedral potential, OH charge less polar
```

```
[ moleculetype ]
; Name              nrexcl
XEG                   3
[ atoms ]
;   nr       type  resnr residue  atom   cgnr     charge       mass
     1   peg_OA        1     XEG   OAX1      1    -0.5884    15.9990
     2   peg_C         1     XEG   CX1       1     0.1086    12.0110
     3   peg_C         1     XEG   CX2       1     0.0082    12.0110
     4   peg_OE        1     XEG   OEX1      1    -0.3849    15.9990
     5   peg_C         1     XEG   CX3       1     0.0078    12.0110
     6   peg_C         1     XEG   CX4       1     0.0085    12.0110
     7   peg_OE        1     XEG   OEX2      1     -0.384    15.9990
     8   peg_C         1     XEG   CX5       1     0.0086    12.0110
     9   peg_C         1     XEG   CX6       1     0.0084    12.0110
    10   peg_OE        1     XEG   OEX3      1     -0.384    15.9990
    11   peg_C         1     XEG   CX7       1     0.0087    12.0110
    12   peg_C         1     XEG   CX8       1     0.0082    12.0110
    13   peg_OE        1     XEG   OEX4      1     -0.384    15.9990
    14   peg_C         1     XEG   CX9       1     0.0084    12.0110
    15   peg_C         1     XEG   CXA       1     0.0067    12.0110
    16   peg_OE        1     XEG   OEX5      1    -0.3986    15.9990
    17   peg_C         1     XEG   CXB       1    -0.0411    12.0110
    18   peg_C         1     XEG   CXC       1     0.1084    12.0110
    19   peg_OA        1     XEG   OAX2      1    -0.5855    15.9990
    20   peg_HA        1     XEG   HAX1      1     0.3175     1.0080
    21   peg_HC        1     XEG   HCX1      1     0.0821     1.0080
    22   peg_HC        1     XEG   HCX2      1     0.0821     1.0080
    23   peg_HC        1     XEG   HCX3      1     0.0925     1.0080
    24   peg_HC        1     XEG   HCX4      1     0.0925     1.0080
    25   peg_HC        1     XEG   HCX5      1     0.0917     1.0080
    26   peg_HC        1     XEG   HCX6      1     0.0917     1.0080
    27   peg_HC        1     XEG   HCX7      1     0.0916     1.0080
    28   peg_HC        1     XEG   HCX8      1     0.0916     1.0080
    29   peg_HC        1     XEG   HCX9      1     0.0916     1.0080
    30   peg_HC        1     XEG   HCXA      1     0.0916     1.0080
    31   peg_HC        1     XEG   HCXB      1     0.0918     1.0080
    32   peg_HC        1     XEG   HCXC      1     0.0918     1.0080
    33   peg_HC        1     XEG   HCXD      2     0.0917     1.0080
    34   peg_HC        1     XEG   HCXE      2     0.0917     1.0080
    35   peg_HC        1     XEG   HCXF      2      0.092     1.0080
    36   peg_HC        1     XEG   HCXG      2      0.092     1.0080
    37   peg_HC        1     XEG   HCXH      2     0.0914     1.0080
    38   peg_HC        1     XEG   HCXI      2     0.0914     1.0080
    39   peg_HC        1     XEG   HCXJ      2     0.0919     1.0080
    40   peg_HC        1     XEG   HCXK      2     0.0919     1.0080
    41   peg_HC        1     XEG   HCXL      2     0.0945     1.0080
    42   peg_HC        1     XEG   HCXM      2     0.0945     1.0080
    43   peg_HC        1     XEG   HCXN      2     0.1067     1.0080
    44   peg_HC        1     XEG   HCXO      2     0.1067     1.0080
    45   peg_HA        1     XEG   HAX2      2     0.3235     1.0080
[ bonds ]
    2     1     1      0.1410 267776.000
    3     2     1      0.1529 224262.400
    4     3     1      0.1410 267776.000
    5     4     1      0.1410 267776.000
    6     5     1      0.1529 224262.400
```

```
    7     6     1      0.1410 267776.000
    8     7     1      0.1410 267776.000
    9     8     1      0.1529 224262.400
   10     9     1      0.1410 267776.000
   11    10     1      0.1410 267776.000
   12    11     1      0.1529 224262.400
   13    12     1      0.1410 267776.000
   14    13     1      0.1410 267776.000
   15    14     1      0.1529 224262.400
   16    15     1      0.1410 267776.000
   17    16     1      0.1410 267776.000
   18    17     1      0.1529 224262.400
   19    18     1      0.1410 267776.000
   20     1     1      0.0945 462750.400
   21     2     1      0.1090 284512.000
   22     2     1      0.1090 284512.000
   23     3     1      0.1090 284512.000
   24     3     1      0.1090 284512.000
   25     5     1      0.1090 284512.000
   26     5     1      0.1090 284512.000
   27     6     1      0.1090 284512.000
   28     6     1      0.1090 284512.000
   29     8     1      0.1090 284512.000
   30     8     1      0.1090 284512.000
   31     9     1      0.1090 284512.000
   32     9     1      0.1090 284512.000
   33    11     1      0.1090 284512.000
   34    11     1      0.1090 284512.000
   35    12     1      0.1090 284512.000
   36    12     1      0.1090 284512.000
   37    14     1      0.1090 284512.000
   38    14     1      0.1090 284512.000
   39    15     1      0.1090 284512.000
   40    15     1      0.1090 284512.000
   41    17     1      0.1090 284512.000
   42    17     1      0.1090 284512.000
   43    18     1      0.1090 284512.000
   44    18     1      0.1090 284512.000
   45    19     1      0.0945 462750.400

[ angles ]
;  ai    aj    ak funct           c0           c1           c2           c3
    1     2     3     1    109.500    418.400
    2     3     4     1    109.500    418.400
    3     4     5     1    109.500    502.080
    4     5     6     1    109.500    418.400
    5     6     7     1    109.500    418.400
    6     7     8     1    109.500    502.080
    7     8     9     1    109.500    418.400
    8     9    10     1    109.500    418.400
    9    10    11     1    109.500    502.080
   10    11    12     1    109.500    418.400
   11    12    13     1    109.500    418.400
   12    13    14     1    109.500    502.080
   13    14    15     1    109.500    418.400
   14    15    16     1    109.500    418.400
   15    16    17     1    109.500    502.080
```

16	17	18	1	109.500	418.400
17	18	19	1	109.500	418.400
2	1	20	1	108.500	460.240
1	2	21	1	109.500	292.880
1	2	22	1	109.500	292.880
2	3	23	1	110.700	313.800
2	3	24	1	110.700	313.800
4	5	25	1	109.500	292.880
4	5	26	1	109.500	292.880
5	6	27	1	110.700	313.800
5	6	28	1	110.700	313.800
7	8	29	1	109.500	292.880
7	8	30	1	109.500	292.880
8	9	31	1	110.700	313.800
8	9	32	1	110.700	313.800
10	11	33	1	109.500	292.880
10	11	34	1	109.500	292.880
11	12	35	1	110.700	313.800
11	12	36	1	110.700	313.800
13	14	37	1	109.500	292.880
13	14	38	1	109.500	292.880
14	15	39	1	110.700	313.800
14	15	40	1	110.700	313.800
16	17	41	1	109.500	292.880
16	17	42	1	109.500	292.880
17	18	43	1	110.700	313.800
17	18	44	1	110.700	313.800
18	19	45	1	108.500	460.240
9	8	30	1	110.700	313.800
18	17	41	1	110.700	313.800
10	9	32	1	109.500	292.880
7	6	28	1	109.500	292.880
3	2	21	1	110.700	313.800
27	6	28	1	107.800	276.144
9	8	29	1	110.700	313.800
4	3	23	1	109.500	292.880
37	14	38	1	107.800	276.144
12	11	33	1	110.700	313.800
13	12	36	1	109.500	292.880
23	3	24	1	107.800	276.144
43	18	44	1	107.800	276.144
3	2	22	1	110.700	313.800
13	12	35	1	109.500	292.880
16	15	39	1	109.500	292.880
35	12	36	1	107.800	276.144
15	14	37	1	110.700	313.800
15	14	38	1	110.700	313.800
39	15	40	1	107.800	276.144
16	15	40	1	109.500	292.880
19	18	43	1	109.500	292.880
33	11	34	1	107.800	276.144
7	6	27	1	109.500	292.880
25	5	26	1	107.800	276.144
19	18	44	1	109.500	292.880
4	3	24	1	109.500	292.880
10	9	31	1	109.500	292.880
12	11	34	1	110.700	313.800

```
   41    17    42     1    107.800    276.144
    6     5    26     1    110.700    313.800
   31     9    32     1    107.800    276.144
    6     5    25     1    110.700    313.800
   18    17    42     1    110.700    313.800
   29     8    30     1    107.800    276.144
   21     2    22     1    107.800    276.144

[ dihedrals ]
; IMPROPER DIHEDRAL ANGLES
;  ai    aj    ak    al funct            c0            c1            c2            c3
c4            c5

[ dihedrals ]
; PROPER DIHEDRAL ANGLES
;  ai    aj    ak    al funct            c0            c1            c2            c3
c4            c5
   12   11   10    9         3       1.715   2.845   1.046  -5.607  -0.000   0.000
    6    5    4    3         3       1.715   2.845   1.046  -5.607  -0.000   0.000
   18   17   16   15         3       1.715   2.845   1.046  -5.607  -0.000   0.000
    9    8    7    6         3       1.715   2.845   1.046  -5.607  -0.000   0.000
   15   14   13   12         3       1.715   2.845   1.046  -5.607  -0.000   0.000
    5    4    3    2         3       1.715   2.845   1.046  -5.607  -0.000   0.000
    8    7    6    5         3       1.715   2.845   1.046  -5.607  -0.000   0.000
   17   16   15   14         3       1.715   2.845   1.046  -5.607  -0.000   0.000
   11   10    9    8         3       1.715   2.845   1.046  -5.607  -0.000   0.000
   14   13   12   11         3       1.715   2.845   1.046  -5.607  -0.000   0.000
   32    9    8   30         3       0.628   1.883   0.000  -2.510  -0.000   0.000
   35   12   11   34         3       0.628   1.883   0.000  -2.510  -0.000   0.000
   43   18   17   42         3       0.628   1.883   0.000  -2.510  -0.000   0.000
   31    9    8   29         3       0.628   1.883   0.000  -2.510  -0.000   0.000
   40   15   14   38         3       0.628   1.883   0.000  -2.510  -0.000   0.000
   44   18   17   42         3       0.628   1.883   0.000  -2.510  -0.000   0.000
   27    6    5   25         3       0.628   1.883   0.000  -2.510  -0.000   0.000
   24    3    2   21         3       0.628   1.883   0.000  -2.510  -0.000   0.000
   23    3    2   22         3       0.628   1.883   0.000  -2.510  -0.000   0.000
   40   15   14   37         3       0.628   1.883   0.000  -2.510  -0.000   0.000
   44   18   17   41         3       0.628   1.883   0.000  -2.510  -0.000   0.000
   39   15   14   37         3       0.628   1.883   0.000  -2.510  -0.000   0.000
   35   12   11   33         3       0.628   1.883   0.000  -2.510  -0.000   0.000
   43   18   17   41         3       0.628   1.883   0.000  -2.510  -0.000   0.000
   39   15   14   38         3       0.628   1.883   0.000  -2.510  -0.000   0.000
   24    3    2   22         3       0.628   1.883   0.000  -2.510  -0.000   0.000
   23    3    2   21         3       0.628   1.883   0.000  -2.510  -0.000   0.000
   32    9    8   29         3       0.628   1.883   0.000  -2.510  -0.000   0.000
   28    6    5   26         3       0.628   1.883   0.000  -2.510  -0.000   0.000
   31    9    8   30         3       0.628   1.883   0.000  -2.510  -0.000   0.000
   36   12   11   34         3       0.628   1.883   0.000  -2.510  -0.000   0.000
   36   12   11   33         3       0.628   1.883   0.000  -2.510  -0.000   0.000
   27    6    5   26         3       0.628   1.883   0.000  -2.510  -0.000   0.000
   28    6    5   25         3       0.628   1.883   0.000  -2.510  -0.000   0.000
   23    3    2    1         3       0.979   2.937   0.000  -3.916  -0.000   0.000
   41   17   18   19         3       0.979   2.937   0.000  -3.916  -0.000   0.000
   24    3    2    1         3       0.979   2.937   0.000  -3.916  -0.000   0.000
   42   17   18   19         3       0.979   2.937   0.000  -3.916  -0.000   0.000
   27    6    5    4         3       0.979   2.937   0.000  -3.916  -0.000   0.000
   29    8    9   10         3       0.979   2.937   0.000  -3.916  -0.000   0.000
```

```
   38   14   15   16        3       0.979   2.937   0.000  -3.916  -0.000   0.000
   32    9    8    7        3       0.979   2.937   0.000  -3.916  -0.000   0.000
   31    9    8    7        3       0.979   2.937   0.000  -3.916  -0.000   0.000
   22    2    3    4        3       0.979   2.937   0.000  -3.916  -0.000   0.000
   36   12   11   10        3       0.979   2.937   0.000  -3.916  -0.000   0.000
   33   11   12   13        3       0.979   2.937   0.000  -3.916  -0.000   0.000
   26    5    6    7        3       0.979   2.937   0.000  -3.916  -0.000   0.000
   34   11   12   13        3       0.979   2.937   0.000  -3.916  -0.000   0.000
   21    2    3    4        3       0.979   2.937   0.000  -3.916  -0.000   0.000
   43   18   17   16        3       0.979   2.937   0.000  -3.916  -0.000   0.000
   44   18   17   16        3       0.979   2.937   0.000  -3.916  -0.000   0.000
   37   14   15   16        3       0.979   2.937   0.000  -3.916  -0.000   0.000
   39   15   14   13        3       0.979   2.937   0.000  -3.916  -0.000   0.000
   35   12   11   10        3       0.979   2.937   0.000  -3.916  -0.000   0.000
   28    6    5    4        3       0.979   2.937   0.000  -3.916  -0.000   0.000
   25    5    6    7        3       0.979   2.937   0.000  -3.916  -0.000   0.000
   30    8    9   10        3       0.979   2.937   0.000  -3.916  -0.000   0.000
   40   15   14   13        3       0.979   2.937   0.000  -3.916  -0.000   0.000
   22    2    1   20        3       0.736   2.209   0.000  -2.946  -0.000   0.000
   21    2    1   20        3       0.736   2.209   0.000  -2.946  -0.000   0.000
   33   11   10    9        3       1.590   4.770   0.000  -6.360  -0.000   0.000
   23    3    4    5        3       1.590   4.770   0.000  -6.360  -0.000   0.000
   38   14   13   12        3       1.590   4.770   0.000  -6.360  -0.000   0.000
   29    8    7    6        3       1.590   4.770   0.000  -6.360  -0.000   0.000
   39   15   16   17        3       1.590   4.770   0.000  -6.360  -0.000   0.000
   36   12   13   14        3       1.590   4.770   0.000  -6.360  -0.000   0.000
   34   11   10    9        3       1.590   4.770   0.000  -6.360  -0.000   0.000
   30    8    7    6        3       1.590   4.770   0.000  -6.360  -0.000   0.000
   32    9   10   11        3       1.590   4.770   0.000  -6.360  -0.000   0.000
   25    5    4    3        3       1.590   4.770   0.000  -6.360  -0.000   0.000
   28    6    7    8        3       1.590   4.770   0.000  -6.360  -0.000   0.000
   27    6    7    8        3       1.590   4.770   0.000  -6.360  -0.000   0.000
   42   17   16   15        3       1.590   4.770   0.000  -6.360  -0.000   0.000
   26    5    4    3        3       1.590   4.770   0.000  -6.360  -0.000   0.000
   24    3    4    5        3       1.590   4.770   0.000  -6.360  -0.000   0.000
   41   17   16   15        3       1.590   4.770   0.000  -6.360  -0.000   0.000
   31    9   10   11        3       1.590   4.770   0.000  -6.360  -0.000   0.000
   35   12   13   14        3       1.590   4.770   0.000  -6.360  -0.000   0.000
   40   15   16   17        3       1.590   4.770   0.000  -6.360  -0.000   0.000
   37   14   13   12        3       1.590   4.770   0.000  -6.360  -0.000   0.000
   20    1    2    3        3      -0.444   3.833   0.728  -4.117  -0.000   0.000
   45   19   18   17        3      -0.444   3.833   0.728  -4.117  -0.000   0.000
   45   19   18   44        3       0.736   2.209   0.000  -2.946  -0.000   0.000
   45   19   18   43        3       0.736   2.209   0.000  -2.946  -0.000   0.000
   19   18   17   16        3       4.518  -4.518   0.000  -0.000  -0.000   0.000
    4    3    2    1        3       4.518  -4.518   0.000  -0.000  -0.000   0.000
   13   12   11   10        3      -1.151   1.151   0.000  -0.000  -0.000   0.000
   10    9    8    7        3      -1.151   1.151   0.000  -0.000  -0.000   0.000
    7    6    5    4        3      -1.151   1.151   0.000  -0.000  -0.000   0.000
   16   15   14   13        3      -1.151   1.151   0.000  -0.000  -0.000   0.000

[ pairs ]
     1     4    1
     2     5    1
     3     6    1
     4     7    1
     5     8    1
```

6	9	1
7	10	1
8	11	1
9	12	1
10	13	1
3	20	1
1	23	1
11	14	1
4	21	1
1	24	1
4	22	1
12	15	1
5	23	1
3	25	1
13	16	1
5	24	1
3	26	1
14	17	1
4	27	1
7	25	1
4	28	1
15	18	1
7	26	1
16	19	1
8	27	1
6	29	1
8	28	1
6	30	1
7	31	1
10	29	1
7	32	1
10	30	1
20	21	1
20	22	1
11	31	1
9	33	1
11	32	1
9	34	1
21	23	1
22	23	1
21	24	1
10	35	1
22	24	1
13	33	1
10	36	1
13	34	1
14	35	1
12	37	1
14	36	1
12	38	1
25	27	1
13	39	1
26	27	1
25	28	1
16	37	1
13	40	1
26	28	1

```
    16    38    1
    17    39    1
    15    41    1
    17    40    1
    15    42    1
    16    43    1
    29    31    1
    19    41    1
    16    44    1
    30    31    1
    29    32    1
    19    42    1
    30    32    1
    17    45    1
    33    35    1
    34    35    1
    33    36    1
    34    36    1
    37    39    1
    38    39    1
    37    40    1
    38    40    1
    41    43    1
    42    43    1
    41    44    1
    42    44    1
    43    45    1
    44    45    1
```

Heptaethylene Glycol

```
;
; GENERATED BY LigParGen Server
; Jorgensen Lab @ Yale University
; MODIFIED: 1/2 dihedral potential, OH charge less polar

[ moleculetype ]
; Name               nrexcl
HEG                   3
[ atoms ]
;   nr       type  resnr residue  atom   cgnr     charge       mass
     1   peg_OA        1    HEG   OAH1      1    -0.5890    15.9990
     2   peg_C         1    HEG   CH1       1     0.1083    12.0110
     3   peg_C         1    HEG   CH2       1      0.008    12.0110
     4   peg_OE        1    HEG   OEH1      1    -0.3846    15.9990
     5   peg_C         1    HEG   CH3       1     0.0073    12.0110
     6   peg_C         1    HEG   CH4       1     0.0087    12.0110
     7   peg_OE        1    HEG   OEH2      1    -0.3855    15.9990
     8   peg_C         1    HEG   CH5       1     0.0088    12.0110
     9   peg_C         1    HEG   CH6       1      0.005    12.0110
    10   peg_OE        1    HEG   OEH3      1    -0.3762    15.9990
    11   peg_C         1    HEG   CH7       1     0.0105    12.0110
    12   peg_C         1    HEG   CH8       1     0.0107    12.0110
    13   peg_OE        1    HEG   OEH4      1    -0.3759    15.9990
    14   peg_C         1    HEG   CH9       1     0.0055    12.0110
    15   peg_C         1    HEG   CHA       1     0.0085    12.0110
```

```
    16   peg_OE        1    HEG   OEH5      1    -0.3859    15.9990
    17   peg_C         1    HEG   CHB       1     0.0087    12.0110
    18   peg_C         1    HEG   CHC       1     0.0065    12.0110
    19   peg_OE        1    HEG   OEH6      1    -0.3985    15.9990
    20   peg_C         1    HEG   CHD       1     -0.041    12.0110
    21   peg_C         1    HEG   CHE       1     0.1082    12.0110
    22   peg_OA        1    HEG   OAH2      1    -0.5863    15.9990
    23   peg_HA        1    HEG   HAH1      1     0.3173     1.0080
    24   peg_HC        1    HEG   HCH1      1     0.0821     1.0080
    25   peg_HC        1    HEG   HCH2      1     0.0821     1.0080
    26   peg_HC        1    HEG   HCH3      1     0.0923     1.0080
    27   peg_HC        1    HEG   HCH4      1     0.0923     1.0080
    28   peg_HC        1    HEG   HCH5      1     0.0913     1.0080
    29   peg_HC        1    HEG   HCH6      1     0.0913     1.0080
    30   peg_HC        1    HEG   HCH7      1     0.0915     1.0080
    31   peg_HC        1    HEG   HCH8      1     0.0915     1.0080
    32   peg_HC        1    HEG   HCH9      1     0.0925     1.0080
    33   peg_HC        1    HEG   HCHA      2     0.0925     1.0080
    34   peg_HC        1    HEG   HCHB      2     0.0881     1.0080
    35   peg_HC        1    HEG   HCHC      2     0.0881     1.0080
    36   peg_HC        1    HEG   HCHD      2     0.0931     1.0080
    37   peg_HC        1    HEG   HCHE      2     0.0931     1.0080
    38   peg_HC        1    HEG   HCHF      2     0.0929     1.0080
    39   peg_HC        1    HEG   HCHG      2     0.0929     1.0080
    40   peg_HC        1    HEG   HCHH      2     0.0878     1.0080
    41   peg_HC        1    HEG   HCHI      2     0.0878     1.0080
    42   peg_HC        1    HEG   HCHJ      2      0.093     1.0080
    43   peg_HC        1    HEG   HCHK      2      0.093     1.0080
    44   peg_HC        1    HEG   HCHL      2     0.0915     1.0080
    45   peg_HC        1    HEG   HCHM      2     0.0915     1.0080
    46   peg_HC        1    HEG   HCHN      2     0.0915     1.0080
    47   peg_HC        1    HEG   HCHO      2     0.0915     1.0080
    48   peg_HC        1    HEG   HCHP      2     0.0943     1.0080
    49   peg_HC        1    HEG   HCHQ      2     0.0943     1.0080
    50   peg_HC        1    HEG   HCHR      2     0.1067     1.0080
    51   peg_HC        1    HEG   HCHS      2     0.1067     1.0080
    52   peg_HA        1    HEG   HAH2      2     0.3237     1.0080
[ bonds ]
    2     1     1      0.1410 267776.000
    3     2     1      0.1529 224262.400
    4     3     1      0.1410 267776.000
    5     4     1      0.1410 267776.000
    6     5     1      0.1529 224262.400
    7     6     1      0.1410 267776.000
    8     7     1      0.1410 267776.000
    9     8     1      0.1529 224262.400
   10     9     1      0.1410 267776.000
   11    10     1      0.1410 267776.000
   12    11     1      0.1529 224262.400
   13    12     1      0.1410 267776.000
   14    13     1      0.1410 267776.000
   15    14     1      0.1529 224262.400
   16    15     1      0.1410 267776.000
   17    16     1      0.1410 267776.000
   18    17     1      0.1529 224262.400
   19    18     1      0.1410 267776.000
   20    19     1      0.1410 267776.000
```

```
    21    20     1      0.1529 224262.400
    22    21     1      0.1410 267776.000
    23     1     1      0.0945 462750.400
    24     2     1      0.1090 284512.000
    25     2     1      0.1090 284512.000
    26     3     1      0.1090 284512.000
    27     3     1      0.1090 284512.000
    28     5     1      0.1090 284512.000
    29     5     1      0.1090 284512.000
    30     6     1      0.1090 284512.000
    31     6     1      0.1090 284512.000
    32     8     1      0.1090 284512.000
    33     8     1      0.1090 284512.000
    34     9     1      0.1090 284512.000
    35     9     1      0.1090 284512.000
    36    11     1      0.1090 284512.000
    37    11     1      0.1090 284512.000
    38    12     1      0.1090 284512.000
    39    12     1      0.1090 284512.000
    40    14     1      0.1090 284512.000
    41    14     1      0.1090 284512.000
    42    15     1      0.1090 284512.000
    43    15     1      0.1090 284512.000
    44    17     1      0.1090 284512.000
    45    17     1      0.1090 284512.000
    46    18     1      0.1090 284512.000
    47    18     1      0.1090 284512.000
    48    20     1      0.1090 284512.000
    49    20     1      0.1090 284512.000
    50    21     1      0.1090 284512.000
    51    21     1      0.1090 284512.000
    52    22     1      0.0945 462750.400

 [ angles ]
;  ai    aj    ak funct            c0            c1            c2            c3
     1     2     3     1    109.500    418.400
     2     3     4     1    109.500    418.400
     3     4     5     1    109.500    502.080
     4     5     6     1    109.500    418.400
     5     6     7     1    109.500    418.400
     6     7     8     1    109.500    502.080
     7     8     9     1    109.500    418.400
     8     9    10     1    109.500    418.400
     9    10    11     1    109.500    502.080
    10    11    12     1    109.500    418.400
    11    12    13     1    109.500    418.400
    12    13    14     1    109.500    502.080
    13    14    15     1    109.500    418.400
    14    15    16     1    109.500    418.400
    15    16    17     1    109.500    502.080
    16    17    18     1    109.500    418.400
    17    18    19     1    109.500    418.400
    18    19    20     1    109.500    502.080
    19    20    21     1    109.500    418.400
    20    21    22     1    109.500    418.400
     2     1    23     1    108.500    460.240
     1     2    24     1    109.500    292.880
```

1	2	25	1	109.500	292.880
2	3	26	1	110.700	313.800
2	3	27	1	110.700	313.800
4	5	28	1	109.500	292.880
4	5	29	1	109.500	292.880
5	6	30	1	110.700	313.800
5	6	31	1	110.700	313.800
7	8	32	1	109.500	292.880
7	8	33	1	109.500	292.880
8	9	34	1	110.700	313.800
8	9	35	1	110.700	313.800
10	11	36	1	109.500	292.880
10	11	37	1	109.500	292.880
11	12	38	1	110.700	313.800
11	12	39	1	110.700	313.800
13	14	40	1	109.500	292.880
13	14	41	1	109.500	292.880
14	15	42	1	110.700	313.800
14	15	43	1	110.700	313.800
16	17	44	1	109.500	292.880
16	17	45	1	109.500	292.880
17	18	46	1	110.700	313.800
17	18	47	1	110.700	313.800
19	20	48	1	109.500	292.880
19	20	49	1	109.500	292.880
20	21	50	1	110.700	313.800
20	21	51	1	110.700	313.800
21	22	52	1	108.500	460.240
12	11	37	1	110.700	313.800
19	18	47	1	109.500	292.880
13	12	38	1	109.500	292.880
15	14	40	1	110.700	313.800
44	17	45	1	107.800	276.144
16	15	42	1	109.500	292.880
7	6	31	1	109.500	292.880
40	14	41	1	107.800	276.144
48	20	49	1	107.800	276.144
3	2	25	1	110.700	313.800
28	5	29	1	107.800	276.144
16	15	43	1	109.500	292.880
15	14	41	1	110.700	313.800
36	11	37	1	107.800	276.144
21	20	49	1	110.700	313.800
6	5	28	1	110.700	313.800
21	20	48	1	110.700	313.800
32	8	33	1	107.800	276.144
13	12	39	1	109.500	292.880
12	11	36	1	110.700	313.800
42	15	43	1	107.800	276.144
4	3	27	1	109.500	292.880
24	2	25	1	107.800	276.144
10	9	35	1	109.500	292.880
22	21	51	1	109.500	292.880
22	21	50	1	109.500	292.880
18	17	45	1	110.700	313.800
4	3	26	1	109.500	292.880
9	8	32	1	110.700	313.800

```
   46    18    47     1    107.800    276.144
   34     9    35     1    107.800    276.144
    9     8    33     1    110.700    313.800
    7     6    30     1    109.500    292.880
   30     6    31     1    107.800    276.144
   26     3    27     1    107.800    276.144
   18    17    44     1    110.700    313.800
   10     9    34     1    109.500    292.880
   38    12    39     1    107.800    276.144
   19    18    46     1    109.500    292.880
    3     2    24     1    110.700    313.800
    6     5    29     1    110.700    313.800
   50    21    51     1    107.800    276.144

[ dihedrals ]
; IMPROPER DIHEDRAL ANGLES
;  ai    aj    ak    al funct             c0             c1             c2             c3
c4             c5

[ dihedrals ]
; PROPER DIHEDRAL ANGLES
;  ai    aj    ak    al funct             c0             c1             c2             c3
c4             c5
   12   11   10    9         3       1.715   2.845   1.046  -5.607  -0.000   0.000
   18   17   16   15         3       1.715   2.845   1.046  -5.607  -0.000   0.000
   15   14   13   12         3       1.715   2.845   1.046  -5.607  -0.000   0.000
    9    8    7    6         3       1.715   2.845   1.046  -5.607  -0.000   0.000
   21   20   19   18         3       1.715   2.845   1.046  -5.607  -0.000   0.000
    6    5    4    3         3       1.715   2.845   1.046  -5.607  -0.000   0.000
   11   10    9    8         3       1.715   2.845   1.046  -5.607  -0.000   0.000
   20   19   18   17         3       1.715   2.845   1.046  -5.607  -0.000   0.000
   14   13   12   11         3       1.715   2.845   1.046  -5.607  -0.000   0.000
   17   16   15   14         3       1.715   2.845   1.046  -5.607  -0.000   0.000
    5    4    3    2         3       1.715   2.845   1.046  -5.607  -0.000   0.000
    8    7    6    5         3       1.715   2.845   1.046  -5.607  -0.000   0.000
   43   15   14   41         3       0.628   1.883   0.000  -2.510  -0.000   0.000
   35    9    8   32         3       0.628   1.883   0.000  -2.510  -0.000   0.000
   34    9    8   33         3       0.628   1.883   0.000  -2.510  -0.000   0.000
   27    3    2   24         3       0.628   1.883   0.000  -2.510  -0.000   0.000
   26    3    2   25         3       0.628   1.883   0.000  -2.510  -0.000   0.000
   34    9    8   32         3       0.628   1.883   0.000  -2.510  -0.000   0.000
   46   18   17   45         3       0.628   1.883   0.000  -2.510  -0.000   0.000
   39   12   11   37         3       0.628   1.883   0.000  -2.510  -0.000   0.000
   50   21   20   48         3       0.628   1.883   0.000  -2.510  -0.000   0.000
   30    6    5   29         3       0.628   1.883   0.000  -2.510  -0.000   0.000
   51   21   20   48         3       0.628   1.883   0.000  -2.510  -0.000   0.000
   38   12   11   36         3       0.628   1.883   0.000  -2.510  -0.000   0.000
   42   15   14   40         3       0.628   1.883   0.000  -2.510  -0.000   0.000
   47   18   17   44         3       0.628   1.883   0.000  -2.510  -0.000   0.000
   43   15   14   40         3       0.628   1.883   0.000  -2.510  -0.000   0.000
   39   12   11   36         3       0.628   1.883   0.000  -2.510  -0.000   0.000
   38   12   11   37         3       0.628   1.883   0.000  -2.510  -0.000   0.000
   35    9    8   33         3       0.628   1.883   0.000  -2.510  -0.000   0.000
   50   21   20   49         3       0.628   1.883   0.000  -2.510  -0.000   0.000
   30    6    5   28         3       0.628   1.883   0.000  -2.510  -0.000   0.000
   26    3    2   24         3       0.628   1.883   0.000  -2.510  -0.000   0.000
   31    6    5   29         3       0.628   1.883   0.000  -2.510  -0.000   0.000
```

46	18	17	44	3	0.628	1.883	0.000	-2.510	-0.000	0.000
51	21	20	49	3	0.628	1.883	0.000	-2.510	-0.000	0.000
27	3	2	25	3	0.628	1.883	0.000	-2.510	-0.000	0.000
42	15	14	41	3	0.628	1.883	0.000	-2.510	-0.000	0.000
47	18	17	45	3	0.628	1.883	0.000	-2.510	-0.000	0.000
31	6	5	28	3	0.628	1.883	0.000	-2.510	-0.000	0.000
48	20	21	22	3	0.979	2.937	0.000	-3.916	-0.000	0.000
27	3	2	1	3	0.979	2.937	0.000	-3.916	-0.000	0.000
49	20	21	22	3	0.979	2.937	0.000	-3.916	-0.000	0.000
26	3	2	1	3	0.979	2.937	0.000	-3.916	-0.000	0.000
29	5	6	7	3	0.979	2.937	0.000	-3.916	-0.000	0.000
43	15	14	13	3	0.979	2.937	0.000	-3.916	-0.000	0.000
42	15	14	13	3	0.979	2.937	0.000	-3.916	-0.000	0.000
34	9	8	7	3	0.979	2.937	0.000	-3.916	-0.000	0.000
35	9	8	7	3	0.979	2.937	0.000	-3.916	-0.000	0.000
24	2	3	4	3	0.979	2.937	0.000	-3.916	-0.000	0.000
51	21	20	19	3	0.979	2.937	0.000	-3.916	-0.000	0.000
37	11	12	13	3	0.979	2.937	0.000	-3.916	-0.000	0.000
28	5	6	7	3	0.979	2.937	0.000	-3.916	-0.000	0.000
32	8	9	10	3	0.979	2.937	0.000	-3.916	-0.000	0.000
47	18	17	16	3	0.979	2.937	0.000	-3.916	-0.000	0.000
46	18	17	16	3	0.979	2.937	0.000	-3.916	-0.000	0.000
45	17	18	19	3	0.979	2.937	0.000	-3.916	-0.000	0.000
33	8	9	10	3	0.979	2.937	0.000	-3.916	-0.000	0.000
31	6	5	4	3	0.979	2.937	0.000	-3.916	-0.000	0.000
38	12	11	10	3	0.979	2.937	0.000	-3.916	-0.000	0.000
44	17	18	19	3	0.979	2.937	0.000	-3.916	-0.000	0.000
40	14	15	16	3	0.979	2.937	0.000	-3.916	-0.000	0.000
25	2	3	4	3	0.979	2.937	0.000	-3.916	-0.000	0.000
36	11	12	13	3	0.979	2.937	0.000	-3.916	-0.000	0.000
39	12	11	10	3	0.979	2.937	0.000	-3.916	-0.000	0.000
41	14	15	16	3	0.979	2.937	0.000	-3.916	-0.000	0.000
50	21	20	19	3	0.979	2.937	0.000	-3.916	-0.000	0.000
30	6	5	4	3	0.979	2.937	0.000	-3.916	-0.000	0.000
25	2	1	23	3	0.736	2.209	0.000	-2.946	-0.000	0.000
24	2	1	23	3	0.736	2.209	0.000	-2.946	-0.000	0.000
31	6	7	8	3	1.590	4.770	0.000	-6.360	-0.000	0.000
45	17	16	15	3	1.590	4.770	0.000	-6.360	-0.000	0.000
40	14	13	12	3	1.590	4.770	0.000	-6.360	-0.000	0.000
29	5	4	3	3	1.590	4.770	0.000	-6.360	-0.000	0.000
47	18	19	20	3	1.590	4.770	0.000	-6.360	-0.000	0.000
36	11	10	9	3	1.590	4.770	0.000	-6.360	-0.000	0.000
41	14	13	12	3	1.590	4.770	0.000	-6.360	-0.000	0.000
32	8	7	6	3	1.590	4.770	0.000	-6.360	-0.000	0.000
26	3	4	5	3	1.590	4.770	0.000	-6.360	-0.000	0.000
27	3	4	5	3	1.590	4.770	0.000	-6.360	-0.000	0.000
35	9	10	11	3	1.590	4.770	0.000	-6.360	-0.000	0.000
46	18	19	20	3	1.590	4.770	0.000	-6.360	-0.000	0.000
48	20	19	18	3	1.590	4.770	0.000	-6.360	-0.000	0.000
39	12	13	14	3	1.590	4.770	0.000	-6.360	-0.000	0.000
49	20	19	18	3	1.590	4.770	0.000	-6.360	-0.000	0.000
37	11	10	9	3	1.590	4.770	0.000	-6.360	-0.000	0.000
33	8	7	6	3	1.590	4.770	0.000	-6.360	-0.000	0.000
28	5	4	3	3	1.590	4.770	0.000	-6.360	-0.000	0.000
42	15	16	17	3	1.590	4.770	0.000	-6.360	-0.000	0.000
30	6	7	8	3	1.590	4.770	0.000	-6.360	-0.000	0.000
43	15	16	17	3	1.590	4.770	0.000	-6.360	-0.000	0.000

44	17	16	15	3	1.590	4.770	0.000	-6.360	-0.000	0.000
38	12	13	14	3	1.590	4.770	0.000	-6.360	-0.000	0.000
34	9	10	11	3	1.590	4.770	0.000	-6.360	-0.000	0.000
52	22	21	20	3	-0.444	3.833	0.728	-4.117	-0.000	0.000
23	1	2	3	3	-0.444	3.833	0.728	-4.117	-0.000	0.000
52	22	21	51	3	0.736	2.209	0.000	-2.946	-0.000	0.000
52	22	21	50	3	0.736	2.209	0.000	-2.946	-0.000	0.000
22	21	20	19	3	4.518	-4.518	0.000	-0.000	-0.000	0.000
4	3	2	1	3	4.518	-4.518	0.000	-0.000	-0.000	0.000
10	9	8	7	3	-1.151	1.151	0.000	-0.000	-0.000	0.000
13	12	11	10	3	-1.151	1.151	0.000	-0.000	-0.000	0.000
16	15	14	13	3	-1.151	1.151	0.000	-0.000	-0.000	0.000
7	6	5	4	3	-1.151	1.151	0.000	-0.000	-0.000	0.000
19	18	17	16	3	-1.151	1.151	0.000	-0.000	-0.000	0.000

[pairs]

1	4	1
2	5	1
3	6	1
4	7	1
5	8	1
6	9	1
7	10	1
8	11	1
9	12	1
10	13	1
11	14	1
3	23	1
12	15	1
1	26	1
4	24	1
1	27	1
13	16	1
4	25	1
14	17	1
5	26	1
3	28	1
5	27	1
3	29	1
15	18	1
4	30	1
16	19	1
7	28	1
4	31	1
7	29	1
17	20	1
8	30	1
6	32	1
18	21	1
8	31	1
6	33	1
19	22	1
7	34	1
10	32	1
7	35	1
10	33	1
11	34	1

9	36	1
11	35	1
9	37	1
23	24	1
23	25	1
10	38	1
13	36	1
10	39	1
24	26	1
13	37	1
25	26	1
24	27	1
25	27	1
14	38	1
12	40	1
14	39	1
12	41	1
13	42	1
16	40	1
13	43	1
16	41	1
28	30	1
29	30	1
28	31	1
17	42	1
15	44	1
29	31	1
17	43	1
15	45	1
16	46	1
19	44	1
16	47	1
19	45	1
32	34	1
20	46	1
18	48	1
33	34	1
32	35	1
20	47	1
18	49	1
33	35	1
19	50	1
22	48	1
19	51	1
22	49	1
20	52	1
36	38	1
37	38	1
36	39	1
37	39	1
40	42	1
41	42	1
40	43	1
41	43	1
44	46	1
45	46	1
44	47	1

```
    45    47    1
    48    50    1
    49    50    1
    48    51    1
    49    51    1
    50    52    1
    51    52    1
```

4.2.2 Structure Coordinate files

Same as for simulations with OPLS forcefield without any modifications.

4.2.3 Molecular Dynamics files

Same as for simulations with OPLS forcefield without any modifications.

5 Script files

5.1 Script files for finding slice having density equal to average density as starting configuration for NPT production run: “boxcut.sh” and “boxsize.py”

boxcut.sh

```
#!/bin/bash
shopt -s expand_aliases
gmx 1&>/dev/null || module load gromacs/2018.3-CUDA-8.0 2>/dev/null
#|| alias gmx="/nfsopt/gromacs/gromacs-5.1-sse41/jessie/bin/gmx"
usage() { echo "Usage: $0 -p directoryPath/ -e endfrac[0.5] -a automatic -s
startfrac[0.05] -f forcefrac[-1] -d difference[0.00005] [-m dt modulo in ps (for -dt
of gmx traj)] -r redo box.xvg; fractions mean how much of the trajectory is skipped,
4*startfrac is standard!, if automatic fails and -f is not used no gro will be cut"
1>&2; exit 1; }
path="$(pwd)"
FORCEFRAC=-1
difference=0.00005
redo=0
STARTFRAC=0.05
ENDFRAC=0.5
AUTOMATIC=""
IGNORE=0
MODULO=""
while getopts "hf:d:p:ras:e:im:" opt; do
  case $opt in
    f )
        FORCEFRAC=$OPTARG
        ;;
    d )
        difference=$OPTARG
        ;;
    p )
        path="$OPTARG"
        ;;
    a )
        AUTOMATIC=" -a"
        ;;
    s )
        STARTFRAC=$OPTARG
        ;;
    e )
        ENDFRAC=$OPTARG
        ;;
    m )
        MODULO=" -dt $OPTARG"
        ;;
    r )
        redo=1
        ;;
```

```
    i )
        IGNORE=1
        ;;
    h )
      usage
      ;;
   \? )
      usage
      ;;
  esac
done
if [ "${path: -1}" != "/" ] ; then
      path="${path}/"
fi
echo "going to cut optimal box for $path"
#find box.xvg and if not make it
boxfile="${path}box.xvg"
if find "${path}"*/ -maxdepth 1 -name "*.gro" | grep -q .; then
    OUTGRO="$(find "${path}"*/ -maxdepth 1 -name "*.gro")"
    OUTGRO="${OUTGRO[0]}"
    echo "$OUTGRO"
fi
if [ ! -f "$OUTGRO" ] && [ "$IGNORE" == "0" ] ; then
    echo "simulation seems to not have finished, no */out.gro found; exiting"
    echo "use -i if sure"
    exit 1
fi
if [ -f "$OUTGRO" ] && [ -f "$boxfile" ] && [ "$OUTGRO" -nt "$boxfile" ] ; then
    echo "box.xvg existed but out.gro was newer; redoing"
    redo=1
fi
if find "$path" -name *.xtc | grep -q .; then
    trajectory="$(find "$path" -name *.xtc)"
    trajectory="${trajectory[0]}"
else
    echo no trajectory found
    exit 1
fi
if find "$path" -name run*.tpr | grep -q .; then
    runfile="$(find "$path" -name run*.tpr)"
    runfile="${runfile[0]}"
elif find "$path" -name *.tpr | grep -q .; then
    runfile="$(find "$path" -name *.tpr)"
    runfile="${runfile[0]}"
else
    echo "no run*.tpr or *.tpr file found"
    exit 1
fi
if [ -f "$boxfile" ] && [ $redo == 0 ] ; then
      echo "box.xvg already existed"
else
      echo "box.xvg not found or redoing box.xvg"
      echo "using: yes 0 | gmx traj -s $runfile -f $trajectory -ob $boxfile -fp
$MODULO"
      yes 0 | gmx traj -s "$runfile" -f "$trajectory" -ob "$boxfile" -fp $MODULO
2>/dev/null
fi
```

```
echo "----------------------"
echo "Determining optimal box for the trajectory"
FRAMETIME=($(python3 /home/mmh/BrockportMDFiles/python_scripts/boxsize.py -x
"$boxfile" -f $FORCEFRAC -d $difference $AUTOMATIC -s $STARTFRAC -e $ENDFRAC))
echo $FRAMETIME
if [ "$FRAMETIME" != "" ]; then
    equilibriumfile="${path}"equilibrium.gro
    echo "Will cut $equilibriumfile from $trajectory at $frameTime using"
    echo "yes 0 | gmx trjconv -s $runfile -f $trajectory -o $equilibriumfile -b
$FRAMETIME -e $FRAMETIME"
    yes 0 | gmx trjconv -s "$runfile" -f "$trajectory" -o "$equilibriumfile" -b
$FRAMETIME -e $FRAMETIME 2>/dev/null
    echo "done"
    echo "$runfile" "$trajectory"
else
    echo "Automatic determination of cutfraction failed. change parameters, prolong
simulation or force cut with -f!"
    exit 1
fi
exit 0
```

boxsize.py

```
__author__ = 'robin'
import numpy as np
import argparse, sys
from scipy.stats import linregress
def eprint(*args, **kwargs):
    print(*args, file=sys.stderr, **kwargs)
parser = argparse.ArgumentParser(description='Calculates the average box for a given
window and finds the optimal frame for a given tolerance')
parser.add_argument('-x', help='path to xvgfile from gromacs with boxsizes')
parser.add_argument('-f', type=float, default=-1, required=False, help='path to
xvgfile from gromacs with boxsizes')
parser.add_argument('-d', type=float, default=1e-04, required=False, help='path to
xvgfile from gromacs with boxsizes')
parser.add_argument('-a', action='store_true', default=False, required=False,
help='automatic fitting')
parser.add_argument('-s', type=float, default=0.05, required=False, help='Skip
fraction')
parser.add_argument('-e', type=float, default=0.4, required=False, help='End
fraction')
args = parser.parse_args()
xvgfile = args.x
fraction = args.f
diff = args.d
AUTOMATIC = args.a
STARTFRAC = args.s
ENDFRAC = args.e
data = np.loadtxt(xvgfile, skiprows=29, usecols=(0,1,2,3))
#fix times if larger than 1 mus, stupid gromacs
l1 = len(data[data[:,0] < 1.0e+06,0])
l2 = len(data[data[:,0] >= 1.0e+06,0])
if l2 > 0:
    eprint("having to fix timings, i hope it works")
    dt = data[1,0] - data[0,0]
    eprint("data points below and above 1mus ", l1, l2, " dt: ", dt, data[l1-1,0])
```

```
    eprint("before")
    eprint(data[l1-5:l1+5,0])
    #data[l1:,0] = (np.arange(l2)+1)*dt + data[l1-1,0]
    data[:,0] = np.arange(len(data))*dt
    eprint("after")
    eprint(data[l1-5:l1+5,0])
    eprint("does this look correct")
def boxcut(data,CMIN=0.05,CMAX=0.5,CSTEP=0.0001,THRESH=0.00005):
    success = True
    data = np.copy(data)
    data[:,1] = np.prod(data[:,1:],axis=-1)
    def gerade(x,a,b):
        return x*a+b
    ms = []
    for r in np.arange(0.0,0.9001,0.05):
        i = int(len(data)*r)
        m = np.mean(data[i:,1])
        ms.append([r,m])
    c = CMIN
    i = int(len(data)*c)
    m, b, _, _, _ = linregress(data[i:,0],data[i:,1])
    popt = [m,b]
    diff = np.fabs(popt[0])*(data[-1,0]-data[i,0])/np.mean(data[i:,1])
    minpoint = np.array([0, np.inf])
    while (diff > THRESH) & (c <= CMAX):
        c = c+CSTEP
        if c > CMAX:
            eprint("failed")
            success = False
            c = CMAX
            break
        i = int(len(data)*c)
        m, b, _, _, _ = linregress(data[i:,0],data[i:,1])
        popt = [m,b]
        diff = np.fabs(popt[0])*(data[-1,0]-data[i,0])/np.mean(data[i:,1])
        eprint("skipfraction=" + str(round(c,5)).ljust(6,'0') + "
diff="+str(round(diff,5)).ljust(7,'0') + " diff/THRESH=" +
str(round(diff/THRESH,2)).ljust(5,'0'), end='\r')
        if diff < minpoint[1]:
            minpoint = [c, diff]
    mavg = np.mean(data[i:,1])
    eprint("frame, fraction and fitparameters")
    eprint(i, round(c,6), data[i,0], popt)
    eprint("relative drift and averages")
    eprint(diff,mavg, mavg**(1/3))
    eprint("minimum diff point, c and diff, diff/THRESH")
    eprint(str(round(minpoint[0],5)).ljust(6,'0'),str(round(minpoint[1],6)),
str(round(minpoint[1]/THRESH,2)).ljust(5,'0'))
    return c, success
def boxsize_frame(data, fraction, diff=1e-06):
    start = int(len(data)*fraction)-1
    datarest = np.copy(data[start:])
    volumes = datarest[:,0]*datarest[:,1]*datarest[:,2]
    vmean = volumes.mean()
    eprint('Mean boxsize = ' + str(vmean) + ' edge = ' + str(vmean**(1/3)))
    volumes -= vmean
    volumes = np.fabs(volumes)
```

```
    min(volumes)
    volumes[volumes < diff] = 0
    eprint("index is ", len(volumes)-np.argmin(np.flipud(volumes), axis=0)+start-1)
    return len(volumes)-np.argmin(np.flipud(volumes), axis=0)+start-1
if AUTOMATIC:
    eprint("Trying automatic determination of cutting fraction first")
    autofrac, success = boxcut(data, CMIN=STARTFRAC, CMAX=ENDFRAC, THRESH=diff)
    if success:
        frame = boxsize_frame(data[:,1:], autofrac, diff)
    elif fraction > 0:
        eprint("Using forced fraction instead ", fraction)
        frame = boxsize_frame(data[:,1:], fraction, diff)
    else:
        eprint("No forced cut, causing failure")
        print("")
        quit()
else:
    eprint("Using 4*startfrac as fraction, ", 4*STARTFRAC)
    frame = boxsize_frame(data[:,1:], 4*STARTFRAC, diff)
eprint(data[frame])
eprint('ps of frame: ' + str(data[frame,0]) + ' of ' + str(data[len(data)-1, 0]))
print(data[frame,0])
exit
```

5.2 Viscosity analysis scripts “viscosity.sh” and “viscosity.py”

The script files split the trajectory into a chosen number of blocks for analysis and fit the averaged running Green Kubo integrals to obtain the viscosity from the convergence at longer autocorrelation times as exemplary shown in Figure S2.

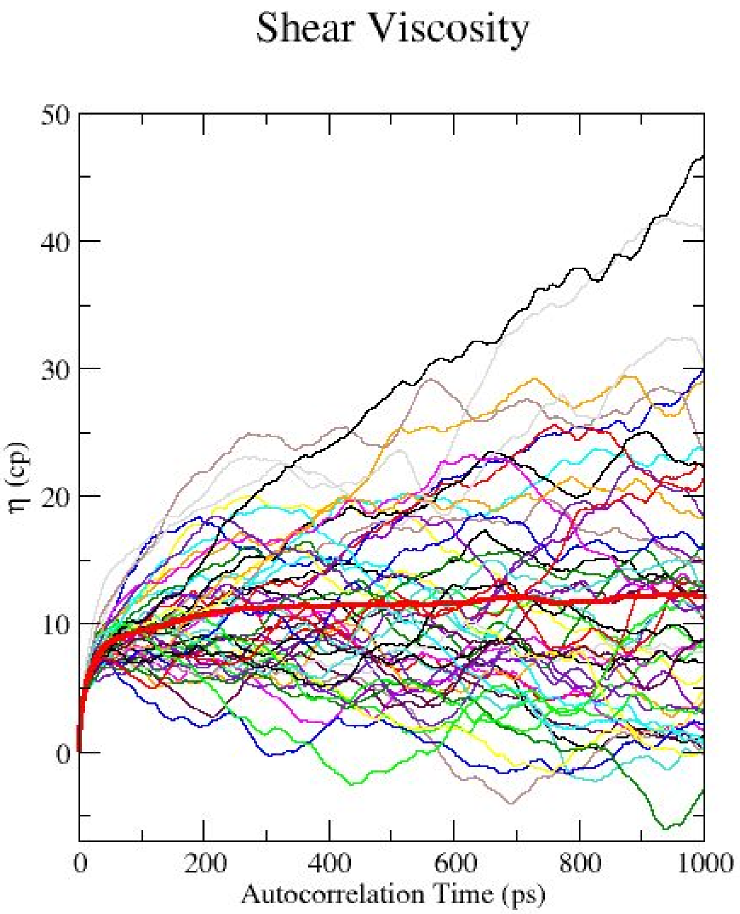

Figure S2: Green-Kubo running integrals for 50 time blocks, with each block containing a simulation time length of 2 ns, for a 1000 molecule neat diethylene glycol system at temperature 328 K. The bolded red line is the average of the 50 running integrals and shows reduced fluctuations at longer autocorrelation times.

a)

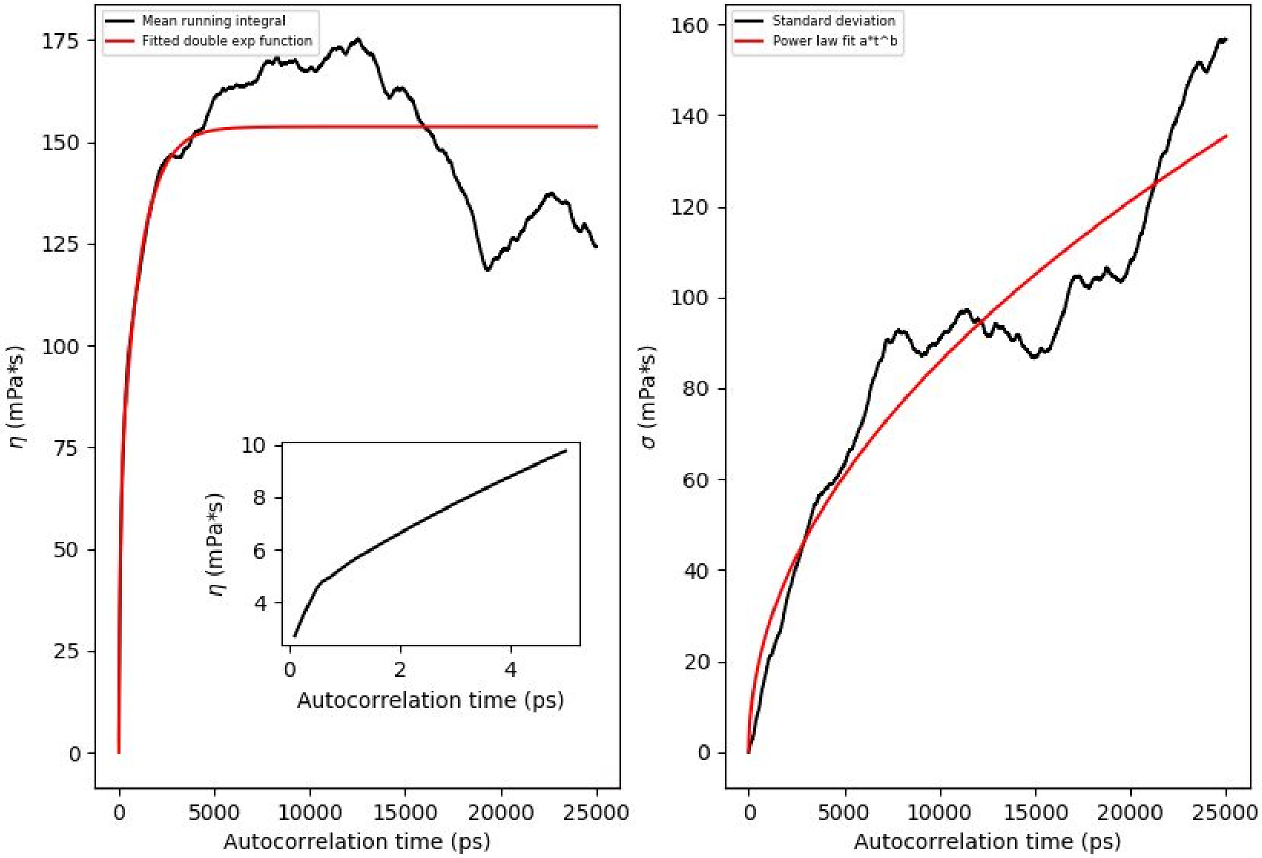
Mean running integral
Fitted double exp function
η (mPa*s)
Autocorrelation time (ps)
Standard deviation
Power law fit a*t^b
σ (mPa*s)

b)

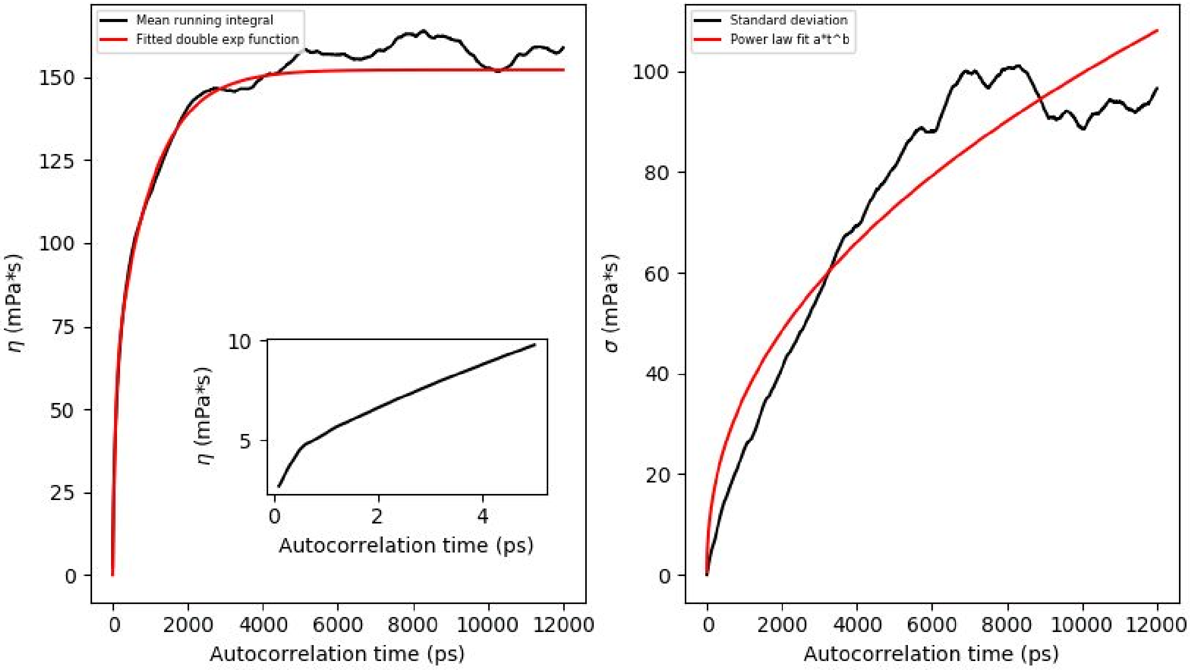
Mean running integral
Fitted double exp function
η (mPa*s)
Autocorrelation time (ps)
Standard deviation
Power law fit a*t^b
σ (mPa*s)

c)

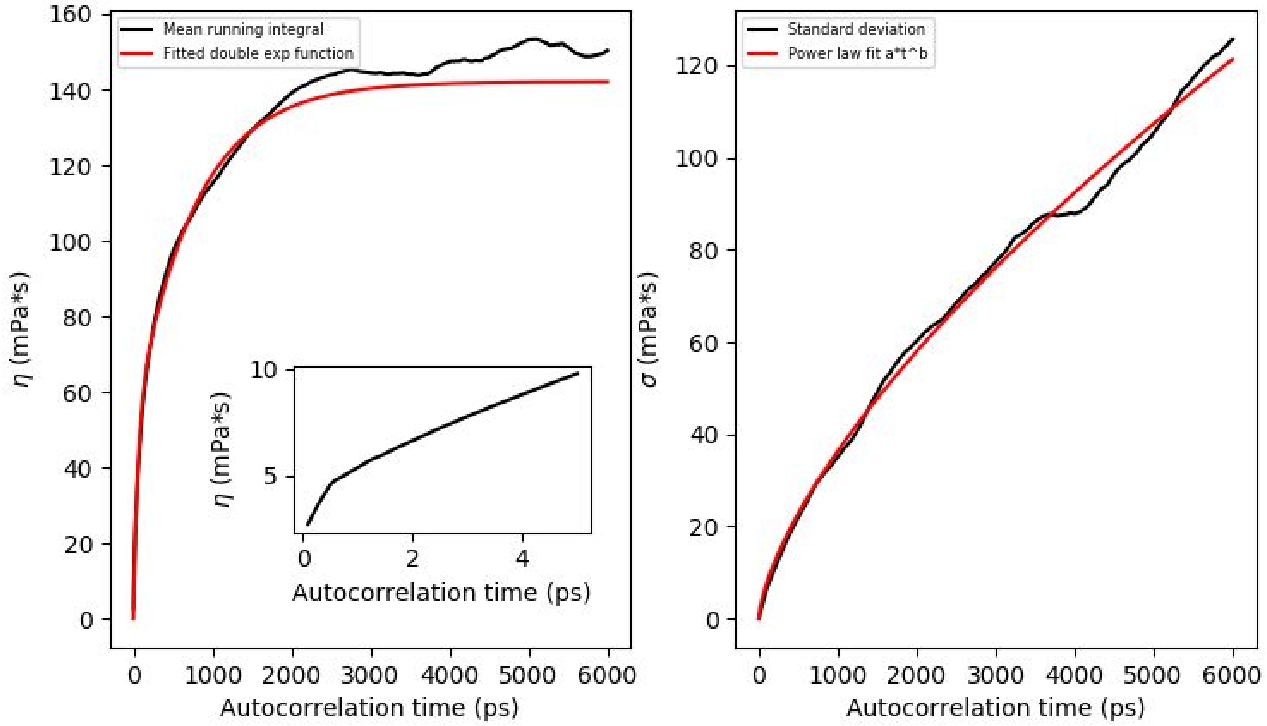

d)

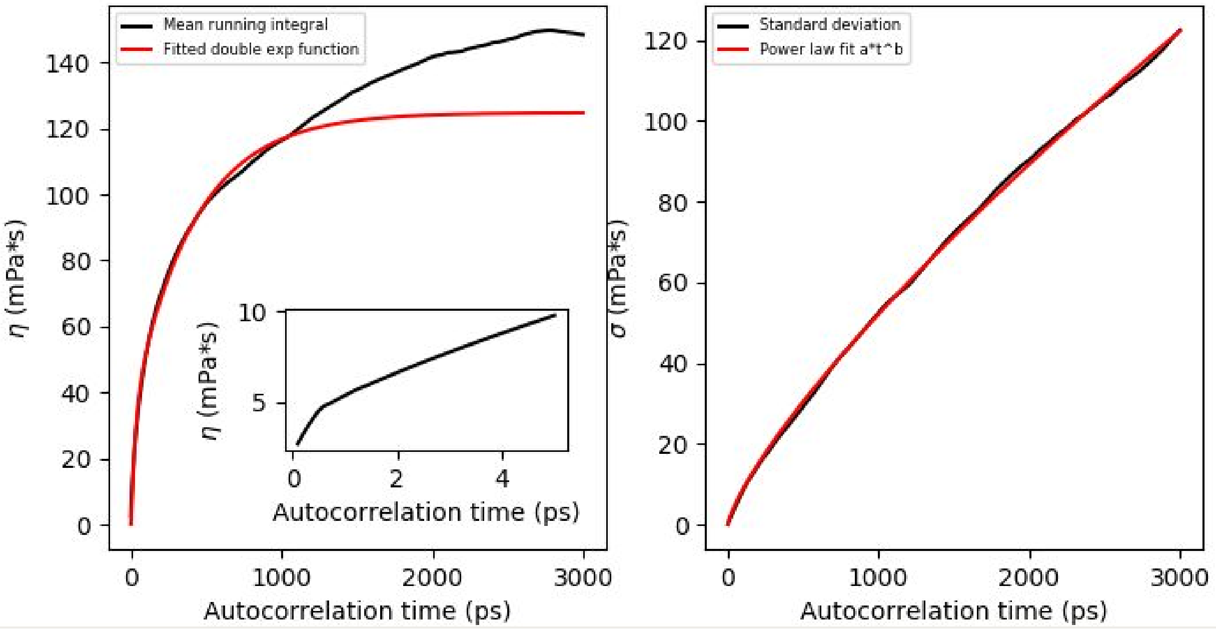

Figure S3: Graphical output graphs obtained from the python.sh and python.py scripts repeating analysis of same simulation trajectory of pentaethylene glycol at 328K using a) 12 boxes of 50,000 ps length, b) 25 boxes of 24,000 ps length, c) 50 boxes of 12,000 ps length, and d) 100 boxes of 6,000 ps length. While convergence improves with fewer boxes of longer time length, the noise unfortunately increases as well. The viscosity result from 25 boxes was chosen as the most reliable, i.e., best compromise of convergence and noise level.

viscosity.sh

```
#!/bin/bash
usage() {
      echo
      echo "Calculates viscosity using time decomposition method"
      echo "J. Chem. Theory Comput. 2015, 11, 3537−3546"
      echo
      echo "USAGE"
      echo "      -b Number of blocks"
      echo "      -t Time length of each block (ps)"
      echo "      -x Total time length of full trajectory (ps))"
      echo "      -g Displays graphs of running integral and its standard deviation"
      echo
      exit 1
}
# setting working directory and GROMACS stuff
WORKDIR="$(dirname "$(readlink -f "$0")")"
PWD="$(pwd)"
source /usr/local/gromacs/bin/GMXRC
# set variables from command line
GRAPH=false
while getopts ":b:t:x:gh" options; do
      case ${options} in
            b)
                  BLOCKNUM=$OPTARG ;;
            t)
                  BLOCKTIME=$OPTARG ;;
            x)
                  FULLTIME=$OPTARG ;;
            g)
                  GRAPH=true ;;
            h)
                  usage
                  exit 1 ;;
            \?)
                  echo "Unknown flag. Please fix input and check the help with -h"
                  exit 1 ;;
            :)
                  echo "Invalid input. You must input an argument for -${OPTARG}.
Check the help with -h"
                  exit 1 ;;
      esac
done
# check for input on command line
if [ -z $BLOCKNUM ] || [ -z $BLOCKNUM ] | [ -z $BLOCKNUM ]; then
      echo "Missing one of the required flags -b, -t, or -x for command line input.
Stopping script"
      exit 1
fi
# Check to make sure the trajectory can be broken down evenly
# if blocks can split the trajectory evenly, then mention perfect even split
# else if total blocking time is less than full trajectory time, then still possible
to do blocking
# else consists of instances where total blocking time exceeds full trajectory time,
which cannot be done
BLOCKTOTALTIME=$(( $BLOCKNUM * $BLOCKTIME ))
```

```
if [ $BLOCKTOTALTIME -eq $FULLTIME ]; then
      echo "Number of blocks $BLOCKNUM of length $BLOCKTIME ps split the entire
trajectory of time $FULLTIME ps evenly."
elif [ $BLOCKTOTALTIME -lt $FULLTIME ]; then
      echo "Number of blocks $BLOCKNUM of length $BLOCKTIME ps can be taken from part
of the trajectory of time $FULLTIME ps."
      echo "Will only use first $BLOCKTOTALTIME ps of full trajectory $FULLTIME ps
for blocking."
      echo "Number of blocks $BLOCKNUM of length $BLOCKTIME ps taken from first
$BLOCKTOTALTIME ps of trajectory."
      echo "If this is not what you want, then please stop the script and re-enter
the correct blocking values."
else
      echo "Number of blocks $BLOCKNUM of length $BLOCKTIME ps cannot be taken from
trajectory of time $FULLTIME ps."
      echo "Please fix the number of blocks and their lengths so it does fit in the
trajectory."
      exit 1
fi

# Running green-kubo calculations
mkdir "$WORKDIR"/viscosity
VISCODIR="$WORKDIR"/viscosity
VISCONUM=1
BEGINTIME=0
ENDTIME=$BLOCKTIME
NVTBOXLENGTH=$(tail -1 "$WORKDIR"/*.gro | rev | cut -d " " -f 1 | rev ) # box length
in nm
NVTVOLUME=$(echo "${NVTBOXLENGTH}^3" | bc -l )  # box volume in nm^3
echo "Box volume is $NVTVOLUME nm^3"
while [ $VISCONUM -lt $(( $BLOCKNUM + 1 )) ]; do
      echo $NVTVOLUME | gmx energy \
            -f "$WORKDIR"/out/*.edr \
            -o "$VISCODIR"/energy_visco${VISCONUM}.xvg \
            -vis "$VISCODIR"/visco${VISCONUM}.xvg \
            -evisco "$VISCODIR"/evisco${VISCONUM}.xvg \
            -eviscoi "$VISCODIR"/eviscoi${VISCONUM}.xvg \
            -b $BEGINTIME \
            -e $ENDTIME \
            -quiet
      mv "$PWD"/enecorr.xvg "$VISCODIR"/enecorrbulkvisc${VISCONUM}.xvg 2>/dev/null;
true
      mv "$PWD"/"#enecorr.xvg.1#" "$VISCODIR"/enecorrshearvisc${VISCONUM}.xvg
2>/dev/null; true
      let BEGINTIME=$BEGINTIME+$BLOCKTIME
      let ENDTIME=$ENDTIME+$BLOCKTIME
      let VISCONUM++
done
if [ "$GRAPH" = true ]; then  # if we want a graph (i.e. GRAPH = TRUE)
      python3 /home/mmh/BrockportMDFiles/python_scripts/viscosity.py
"$VISCODIR"/visco*.xvg -g
else
      python3 /home/mmh/BrockportMDFiles/python_scripts/viscosity.py
"$VISCODIR"/visco*.xvg
fi
```

viscosity.py

```
__author__ = 'matthew'
import numpy as np
from matplotlib import pyplot as plt
import argparse
from scipy.optimize import curve_fit
from scipy.stats import linregress
import math
parser = argparse.ArgumentParser(description="Viscosity time decomposition analysis")
parser.add_argument('files', nargs='+', help="list all visco*.xvg files")
parser.add_argument('-c', default=10000, type=int, help="chunk size for determining
zero slope plateau time, default 10000 energy frames")
parser.add_argument('-s', default=0.01, type=float, help="slope tolerance for
determining zero slope plateau time, default 0.01")
parser.add_argument('-i', default=2, type=float, help="ignore this beginning amount
of time (in ps) for fit to running integral, default 2 ps")
parser.add_argument('-g', action='store_true', help="display graphs. Default is no.")
args = parser.parse_args()
files = args.files
chunksize = args.c
slope_tolerance = args.s
begin_fit_time = args.i
graphs = args.g
nblocks = len(files)
# loading in running integral data for each file
# note that times are same in each file, so take from 1st file (i.e. time is 1D
array)
# y-values are taken from all files, so y is a 2D array with nblocks columns)
time = np.loadtxt(fname=files[0], skiprows=25)[:,0]
dt = time[1] - time[0]
y = np.empty(shape=[len(time), nblocks])
for j in range(0, nblocks):
      y[:,j] = np.loadtxt(fname=files[j], skiprows=25)[:,1]
# find average running integral and stdev as function of time from other running
integrals
# axis 1 means analyze across a row over the columns
print()
print("Determining mean and standard deviation (sigma) of running integrals as
functions of time.")
print()
runningint = np.mean(y, axis=1)
sigma = np.std(y, axis=1, ddof=1)
# fitting sigma(t) to y = a*t^b
def power_law_fit(t, a, b):
      return a * t ** b
sigma_fit_popt, sigma_fit_pcov = curve_fit(power_law_fit, xdata=time, ydata=sigma)
a = sigma_fit_popt[0]
b = sigma_fit_popt[1]
sigma_fit = power_law_fit(time, *sigma_fit_popt)
print("Fitting sigma to power law function.")
print(f"Power law fit: y = {round(a, 3)}*t^{round(b, 3)}")
print()
# need to find zero slope for plateau time and eventually cutoff time for fitting
# define function that returns the index with 0 slope within some slope tolerances
# purpose is to find flat region of running integral
def zero_slope_index(x, y, chunksize, max_slope):
```

```
      for index in range(0, len(x) - chunksize + 1):
            result = linregress(x[index:(index + chunksize)], y[index:(index +
chunksize)])
            slope = result.slope
            if 0 <= abs(slope) <= max_slope:
                  return int((index + (index + chunksize)) / 2)
      # if criteria is not met, function returns nothing
      return None;
# run through a bunch of different max_slope in increments of 0.001 up until
slope_tolerance
# if a slope of approximately zero is found, the time which that occurs will be
reported
# if no slope is found within the specified slope_tolerance on command line, stop
script.
max_slope = 0
while max_slope <= slope_tolerance:
      flat_start_index = zero_slope_index(time, runningint, chunksize=chunksize,
max_slope=max_slope)
      if flat_start_index != None:
            print(f"Zero slope achieved at {time[flat_start_index]} ps using chunks of
{chunksize} energy frames "
                  f"({chunksize*dt} ps) with max slope {max_slope} (mPa*s)/s.")
            break
      max_slope += 0.001
if max_slope > slope_tolerance:
      print(f"Cannot determine plateau time based on current slope tolerance of
{slope_tolerance} (mPa*s)/s.")
      print("Either increase the number of blocks, increase the slope tolerance, or
decrease chunk size. The first option is preferable.")
      print("Stopping script.")
      exit()
# find mean of flat running integral region and use that for time cutoff
determination
runningint_flat_avg = np.mean(runningint[flat_start_index:])
print(f"Average of running integral after zero slope time {time[flat_start_index]} ps
(flat region) is {round(runningint_flat_avg, 4)} mPa*s.")
# t_cutoff is chosen such that sigma(t_cutoff) = 0.4*mean(running integral flat
region)
print(f"t_cutoff should be chosen such that sigma(t_cutoff) is about 40% of the
average over the flat region of {round(runningint_flat_avg, 4)} mPa*s.")
cutoff_sigma = 0.4*runningint_flat_avg
cutoff_time_index_actual=0
print("Calculating cutoff time with exactly 40% criterion.")
while(True):
      if sigma[cutoff_time_index_actual] > cutoff_sigma:
            break
      cutoff_time_index_actual += 1

cutoff_time_actual = time[cutoff_time_index_actual] # actual cutoff time
cutoff_time_rounded = np.ceil(cutoff_time_actual / 100) * 100 # cutoff time rounded
upwards to the hundred; use this
cutoff_time_index_rounded = np.where(time == cutoff_time_rounded)[0][0]
print(f"Actual cutoff time is {cutoff_time_actual} ps. Will round upwards to nearest
hundred to "
      f"{cutoff_time_rounded} ps and use this as the upper limit for the double exp
fit to running integral.")
```

```
print(f"sigma({cutoff_time_rounded} ps) = {round(sigma[cutoff_time_index_rounded], 3)} mPa*s is only "
      f"{np.round(sigma[cutoff_time_index_rounded]/runningint_flat_avg*100, 1)}% of the average over the flat region of running integral of {round(runningint_flat_avg, 4)} mPa*s.")
print("If the percentage is between 30-50%, result is likely satisfactory.")
print(f"Please check that the rounded cutoff time of {cutoff_time_rounded} ps is reasonable and allows the double exp fit to the running integral to cover a moderately reasonable amount of the plateau region.")
print(f"Also please check that the size (time length) of your blocks are at least 10x that of the plateau time {time[flat_start_index]} ps. If not, then the running integral may not converge properly.")
print("However, if increasing the number of blocks is more important (for good statistics) than the length itself, it may be relevant to disregard the previous check within reason.")
print()
# Fitting running integral to complex double exponential function with following conditions
# 1. ignore first few picoseconds (let's do 2 ps as default)
# 2. weight according to 1/t^b
# note that weight = 1 / "sigma"^2 in curve_fit ("sigma" is curve_fit sigma), so "sigma" = 1/sqrt(weight) = t^(b/2)
def double_exp_fit(t, A, alpha, tau_1, tau_2):
      return A*alpha*tau_1*(1 - np.exp(-t/tau_1)) + A*(1 - alpha)*tau_2*(1 - np.exp(-t/tau_2))
weight = 1 / np.power(time, b)
begin_fit_index = np.where(time == begin_fit_time)[0][0]
end_fit_index = np.where(time == cutoff_time_rounded)[0][0]
print(f"Will fit the running integral to a double exp function from {time[begin_fit_index]} ps to {time[end_fit_index]} ps with weight 1/t^{round(b, 3)}.")

runningint_fit_popt, runningint_fit_pcov = curve_fit(double_exp_fit, xdata=time[begin_fit_index:(end_fit_index + 1)],
      ydata=runningint[begin_fit_index:(end_fit_index + 1)], sigma = 1/np.sqrt(weight[begin_fit_index:(end_fit_index + 1)]), absolute_sigma = False)
A = runningint_fit_popt[0]
alpha = runningint_fit_popt[1]
tau_1 = runningint_fit_popt[2]
tau_2 = runningint_fit_popt[3]
runningint_fit = double_exp_fit(time, *runningint_fit_popt)
print(f"Double exp fit: y = {round(A, 3)}*{round(alpha, 3)}*{round(tau_1, 3)}*(1 - e^(-t/{round(tau_1, 3)})) + "
      f"{round(A, 3)}*(1 - {round(alpha, 3)})*{round(tau_2, 3)}*(1 - e^(-t/{round(tau_2, 3)}))")
viscosity_limit = A*alpha*tau_1 + A*(1-alpha)*tau_2
print("Viscosity is calculated by taking the infinite time limit of the double exp fit.")
print(f"Viscosity: {viscosity_limit} mPa*s")
print()
fig, (ax1, ax2) = plt.subplots(1,2, figsize=(8, 4))
ax1.set_xlabel("Autocorrelation time (ps)")
ax1.set_ylabel("$\eta$ (mPa*s)")
ax1.plot(time, runningint, color='black', linestyle='solid', label="Mean running integral")
ax1.plot(time, runningint_fit, color='red', linestyle='solid', label="Fitted double exp function")
```

```
ax1.legend(loc='upper left', prop={'size':6})
left, bottom, width, height = [0.25, 0.2, 0.2, 0.2]
ax3 = fig.add_axes([left, bottom, width, height])
ax3.set_xlabel("Autocorrelation time (ps)")
ax3.set_ylabel("$\eta$ (mPa*s)")
five_ps_index = np.where(time == 5)[0][0]
ax3.plot(time[0:(five_ps_index + 1)], runningint[0:(five_ps_index + 1)],
color='black', linestyle='solid', label="Mean running integral")
ax2.set_xlabel("Autocorrelation time (ps)")
ax2.set_ylabel("$\sigma$ (mPa*s)")
ax2.plot(time, sigma, color='black', linestyle='solid', label="Standard deviation")
ax2.plot(time, sigma_fit, color='red', linestyle='solid', label="Power law fit
a*t^b")
ax2.legend(loc="upper left", prop={'size':6})
if graphs == True:
      plt.show()
```

5.3 Bash script "repeated_simulations.sh" for running multiple MD simulations of same system

```
#!/bin/bash
usage() {
    echo
    echo "Script file for repeating the PEG_water_simulation.sh script which runs a
single simulation."
    echo "See J. Chem. Theory Comput. 2018, 14, 11, 5959-5968 for why we need
repetitions"
    echo
    echo "The script will copy the files below and create as many folders as needed.
Thus, you only "
    echo "need to include the files in the working directory once and let the script
do copying for you."
    echo
    echo "NOTE: This script ONLY runs repeated INITIAL runs (i.e. goes through
insert-molecules and energy min)"
    echo
    echo "Usage:"
    echo "  -b [string]     PEG basename/molecule's acronym (e.g. DEG) (double quotes
around string will work, but not necessary unless there are spaces)"
    echo "                  For pure water, type 'water'"
    echo "  -m [string]     model to simulate (enter 'bulk' for bulk PEG, 'spce' for
spc/e water, or 'tip4p2005' for tip4p/2005 water)"
    echo "  -t [value]      temperature in K (use underscore instead of decimal if
necessary (e.g. 298_15)"
    echo "  -d [value]      density in g/cm^3 at desired temperature"
    echo "  -n [value]      total number of molecules in system"
    echo "  -p [value]      how many number of molecules are PEG"
    echo "  -w [value]      how many number of molecules are water"
    echo "  -r [value]      how many total simulations are desired"
    echo
    echo "Require following files in working directory:"
    echo "  - [BASENAME].itp (PEG itp file) [for bulk PEG and PEG/water mixture
simulations only]"
```

```
    echo "  - [MODEL].itp (water itp file) [for PEG/water mixture and pure water simulations only]"
    echo "  - top_[BASENAME]_bulk.top [for bulk PEG only] or top_[BASENAME]_water.top [for PEG/water mixtures only] or top_pure_water.top [for pure water only]"
    echo "  - single_[BASENAME].gro [for bulk PEG and PEG/water mixture simulations only]"
    echo "  - single_[MODEL].gro [for PEG/water mixture and pure water simulations only]"
    echo "  - em.mdp"
    echo "  - eq_NPT_[TEMP]K.mdp"
    echo "  - md_NVT_[TEMP]K.mdp"
    echo "  - PEG_water_simulation.sh"
    echo "  - This shell file"
    echo
    echo "To start the script, use either one of the following:"
    echo "  - pass no arguments to command line (user input will be asked for)"
    echo "  - pass all 8 flags to command line"
    echo
    exit 1
}
WORKDIR="$(dirname "$(readlink -f "$0")")"
##### Getting parameters and checking that everything is alright to go
# passing arguments to command line
while getopts ":b:m:t:d:n:p:w:r:h" options; do
    case ${options} in
        b)
            BASENAME=$OPTARG ;;
        m)
            MODEL=$OPTARG ;;
        t)
            TEMP=$OPTARG ;;
        d)
            DENSITY=$OPTARG ;;
        n)
            TOTALNMOL=$OPTARG ;;
        p)
            PEGNMOL=$OPTARG ;;
        w)
            WATERNMOL=$OPTARG ;;
        r)
            TOTALRUN=$OPTARG ;;
        h)
            usage ;;
        \?)
            echo "Invalid input. Unknown flag. Refer to the help with -h"
            exit 1 ;;
        :)
            echo "Invalid input: -$OPTARG requires an argument. Refer to the help with -h"
            exit 1 ;;
    esac
done
# if nothing passed to command line, go for user input
if [ $OPTIND -eq 1 ]; then
    read -p "Molecule's acronym: " BASENAME
    read -p "Model to simulate (enter 'bulk' for bulk PEG, 'spce' for spc/e water, or 'tip4p2005' for tip4p/2005 water): " MODEL
```

```
    read -p "Temperature (in K) (Use underscore instead of decimal if necessary, e.g.
298_15): " TEMP
    read -p "${BASENAME}'s density at ${TEMP}K in g/cm^3 (if unknown, choose
something reasonable): " DENSITY
    read -p "Number of total molecules in system: " TOTALNMOL
    read -p "How many of those molecules are PEG: " PEGNMOL
    read -p "How many of those molecules are water: " WATERNMOL
    read -p "Number of simulations to run: " TOTALRUN
fi
# check to see, if user used command line, that all parameters are defined
if [ $OPTIND -gt 1 ]; then
    # forgot basename
    if [ -z $BASENAME ]; then
        echo "You forgot to set the basename. Either use no flags, or use -b"
        exit 1
    fi
    # forgot water model to use
    if [ -z $MODEL ]; then
        echo "You forgot to choose a model to simulate. Either use no flags, or use -
m"
        exit 1
    fi
    # typed an unsupported model
    if [ $MODEL != "bulk" ] && [ $MODEL != "spce" ] && [ $MODEL != "tip4p2005" ];
then
        echo "You have chosen an unsupported model. Please correct -m input"
        exit 1
    fi
    # forgot temperature
    if [ -z $TEMP ]; then
        echo "You forgot to set the temperature. Either use no flags, or use -t"
        exit 1
    fi
    # forgot density
    if [ -z $DENSITY ]; then
        echo "You forgot to set the density. Either use no flags, or use -d"
        exit 1
    fi
    # forgot to specify total number of molecules
    if [ -z $TOTALNMOL ]; then
        echo "You forgot to set the total number of molecules. Either use no flags,
or use -n"
        exit 1
    fi
    # forgot to specify number of PEG molecules
    if [ -z $PEGNMOL ]; then
        echo "You forgot to set the number of PEG molecules. Either use no flags, or
use -p"
        exit 1
    fi
    # forgot to specify number of water molecules
    if [ -z $WATERNMOL ]; then
        echo "You forgot to set the number of water molecules. Either use no flags,
or use -w"
        exit 1
    fi
    # forgot to specify total number of simulations
```

```
    if [ -z $TOTALRUN ]; then
        echo "You forgot to set the number of simulations to run. Either use no
flags, or use -r"
        exit 1
    fi
fi

# if the number of PEG and water molecules do not sum to total molecules in system,
then stop script
if [ $(( $PEGNMOL + $WATERNMOL )) -ne $TOTALNMOL ]; then
    echo "Sum of PEG and water molecules is not equal to total number of molecules
specified. Please correct input."
    exit 1
fi
##### END STEP
##### Creating as many folders as number of desired simulations w/ files. Comment
this section out if continuing set
mkdir "$WORKDIR"/run1
mv "$WORKDIR"/*.itp "$WORKDIR"/run1
mv "$WORKDIR"/*.top "$WORKDIR"/run1
mv "$WORKDIR"/*.gro "$WORKDIR"/run1
mv "$WORKDIR"/*.mdp "$WORKDIR"/run1
mv "$WORKDIR"/PEG_water_simulation.sh "$WORKDIR"/run1
RUN=2
while [ $RUN -lt $(($TOTALRUN + 1)) ]; do
    cp -r "$WORKDIR"/run1 "$WORKDIR"/run${RUN}
    let RUN=$RUN+1
done
##### END STEP
##### Running the simulations as many times as desired. If continuing set, set RUN
and re-run script
RUN=1
while [ $RUN -lt $(($TOTALRUN + 1)) ]; do
    bash "$WORKDIR"/run${RUN}/PEG_water_simulation.sh -b $BASENAME -m $MODEL -t $TEMP
-d $DENSITY -n $TOTALNMOL -p $PEGNMOL -w $WATERNMOL -i
    # mv "$WORKDIR"/energy.xvg "$WORKDIR"/run${RUN}/3_prod_NVT_${TEMP}K/analysis
    # mv "$WORKDIR"/enecorr.xvg
"$WORKDIR"/run${RUN}/3_prod_NVT_${TEMP}K/analysis/enecorrbulkvisc.xvg
    # mv "$WORKDIR"/"#enecorr.xvg.1#"
"$WORKDIR"/run${RUN}/3_prod_NVT_${TEMP}K/analysis/enecorrshearvisc.xvg
    let RUN=$RUN+1
done
##### END STEP
```

5.4 Script for running MD simulation of PEG200

```
#!/bin/bash

# help and usage (called from command line with -h)
usage() {
      echo
      echo "Script file made for GROMACS 2020.4 version compatibility."
```

```
      echo "Individual simulation runs for determining self-diffusion in
thermodynamic limit and shear viscosity via finite-sized effects."
      echo "See J. Chem. Theory Comput. 2018, 14, 11, 5959–5968"
      echo "This bash file must be in the desired working directory."
      echo
      echo "Start initial run:"
      echo "  - pass no arguments to command line (user input will be asked for)"
      echo "  - pass all flags to command line "
      echo
      echo "NOTE 1: Please choose of the above or the script will not run."
      echo "NOTE 2: If using '-m bulk', then use '-w 0' when specifying molecule
numbers."
      echo "NOTE 3: If using '-b water', then use '-p 0' when specifying molecule
numbers."
      echo "NOTE 4: You must use either -s or all integer flags."
      echo "NOTE 5: If -s is not used all integer flags must have a value. Use 0 for
0. Do not skip any integer flags or leave blank."
      echo
      echo "For all simulations make sure the following files are in the mixtures
files directory"
      echo "  - all itp files for desired Poly EGs"
      echo "  - all single molecule gro files for desired Poly EGs"
      echo "  - [MODEL].itp (water itp file) (where [MODEL] is chosen with '-m spce'
or '-m tip4p2005')"
      echo "  - single_[MODEL].gro"
      echo "  - top_mixtures.top"
      echo
      echo "and the following files in working directory:"
      echo "  - em.mdp"
      echo "  - eq_NPT_[TEMP]K.mdp"
      echo "  - md_NVT_[TEMP]K.mdp"
      echo "  - This shell file"
      echo
      echo "Usage:"
      echo "  -b [string]     basename: identifying name for the simulation (use
underscore instead of space if necessary e.g. PEG200_neat)"
      echo "  -m [string]     model to simulate (enter 'bulk' for bulk PolyEG, 'spce'
for spc/e water, or 'tip4p2005' for tip4p/2005 water)"
      echo "  -t [value]      temperature in K (use underscore instead of decimal if
necessary e.g. 298_15)"
      echo "  -f [string]     forcefield (enter 'OPLS' for unmodified OPLS-AA, or
'OPLSMOD' for modified OPLS-AA"
      echo "  -d [value]      density in g/cm^3 at desired temperature"
      echo "  -n [value]      total number of molecules in system"
      echo "  -s [string]     stock mixture. if this option is used integer flags
should not be used. enter 'none' if not using a stock mixture. current options are:
PEG200, none."
      echo "  -1 [value]      how many MEG (monoethylene glycol) molecules. enter 0
if none"
      echo "  -2 [value]      how many DEG (diethylene glycol) molecules. enter 0 if
none"
      echo "  -3 [value]      how many REG (triethylene glycol) molecules. enter 0 if
none"
      echo "  -4 [value]      how many TEG (tetraethylene glycol) molecules. enter 0
if none"
      echo "  -5 [value]      how many PEG (pentaethylene glycol) molecules. enter 0
if none"
```

```
	echo "  -6 [value]      how many XEG (hexaethylene glycol) molecules. enter 0 
if none"
	echo "  -7 [value]      how many HEG (heptaethylene glycol) molecules. enter 0 
if none"
	echo "  -8 [value]      how many OEG (octaethylene glycol) molecules. enter 0 
if none"
	echo "  -9 [value]      how many NEG (nonaethylene glycol) molecules. enter 0 
if none"
	echo "  -p [value]      total number of PolyEG (polyethylene glycol) molecules"
	echo "  -w [value]      how many molecules are water"
	echo
	exit 1
}

# setting working directory and getting gromacs stuff.
WORKDIR="$(dirname "$(readlink -f "$0")")"
source /usr/local/gromacs/bin/GMXRC
export GMX_MAXCONSTRWARN=-1;

##### Begin taking in parameters
# command line inputs
while getopts ":b:1:2:3:4:5:6:7:8:9:p:m:t:d:n:w:s:f:h" options; do
	case ${options} in
		b)
			BASENAME=$OPTARG ;;
		m)
			MODEL=$OPTARG ;;
		t)
			TEMP=$OPTARG ;;
		f)
			FORCEFIELD=$OPTARG ;;
		d)
			DENSITY=$OPTARG ;;
		n)
			TOTALNMOL=$OPTARG ;;
		s)
			STOCKMIX=$OPTARG ;;
		1)
			MEGNMOL=$OPTARG ;;
		2)
			DEGNMOL=$OPTARG ;;
		3)
			REGNMOL=$OPTARG ;;
		4)
			TEGNMOL=$OPTARG ;;
		5)
			PEGNMOL=$OPTARG ;;
		6)
			XEGNMOL=$OPTARG ;;
		7)
			HEGNMOL=$OPTARG ;;
		8)
			OEGNMOL=$OPTARG ;;
		9)
			NEGNMOL=$OPTARG ;;
		p)
			POLYEGNMOL=$OPTARG ;;
```

```
		w)
			WATERNMOL=$OPTARG ;;
		h)
			usage ;;
		\?)
			echo "Invalid input. Unknown flag. Refer to the help with -h"
			exit 1 ;;
		:)
			echo "Invalid input: -$OPTARG requires an argument. Refer to the
help with -h"
			exit 1 ;;
	esac
done

# user input if nothing is specified to command line
if [ $OPTIND -eq 1 ]; then
	read -p "Simulation name: " BASENAME
	read -p "Model to simulate (enter 'bulk' for bulk PolyEG, 'spce' for spc/e
water, or 'tip4p2005' for tip4p/2005 water): " MODEL
	read -p "Temperature (in K) (Use underscore instead of decimal if necessary,
e.g. 298_15): " TEMP
	read -p "Forcefield (enter 'OPLS' for unmodified OPLS-AA, or 'OPLSMOD' for
modified OPLS-AA" FORCEFIELD
	read -p "${BASENAME}'s density at ${TEMP}K in g/cm^3 (if unknown, choose
something reasonable): " DENSITY
	read -p "Number of total molecules in system: " TOTALNMOL
	read -p "Stock mixture (PEG200 or none):" STOCKMIX
	if [ $STOCKMIX = "none" ]; then
		read -p "How many monoethylene glycol molecules: " MEGNMOL
		read -p "How many diethylene glycol molecules: " DEGNMOL
		read -p "How many triethylene glycol molecules: " REGNMOL
		read -p "How many tetraethylene glycol molecules: " TEGNMOL
		read -p "How many pentaethylene glycol molecules: " PEGNMOL
		read -p "How many hexaethylene glycol molecules: " XEGNMOL
		read -p "How many heptaethylene glycol molecules: " HEGNMOL
		read -p "How many octaethylene glycol molecules: " OEGNMOL
		read -p "How many nonaethylene glycol molecules: " NEGNMOL
	fi
	read -p "How many total polyethylene glycol molecules: " POLYEGNMOL
	read -p "How many of those molecules are water: " WATERNMOL
fi
##### END OF STEP

####calculating nmol values based on stock mixtures
if [ $STOCKMIX = "PEG200" ]; then
	MEGNMOL=0
	DEGNMOL=$(printf "%.0f" $(echo "($POLYEGNMOL * 0.03453)" | bc -l))
	REGNMOL=$(printf "%.0f" $(echo "($POLYEGNMOL * 0.22270)" | bc -l))
	TEGNMOL=$(printf "%.0f" $(echo "($POLYEGNMOL * 0.31989)" | bc -l))
	PEGNMOL=$(printf "%.0f" $(echo "($POLYEGNMOL * 0.24644)" | bc -l))
	XEGNMOL=$(printf "%.0f" $(echo "($POLYEGNMOL * 0.12901)" | bc -l))
	HEGNMOL=$(printf "%.0f" $(echo "($POLYEGNMOL * 0.04727)" | bc -l))
	OEGNMOL=0
	NEGNMOL=0

	NMOLCHECK=$(($MEGNMOL + $DEGNMOL + $REGNMOL + $TEGNMOL + $PEGNMOL + $XEGNMOL +
$HEGNMOL + $OEGNMOL + $NEGNMOL))
```

```
	if [ $NMOLCHECK \> $POLYEGNMOL ]; then
		MORE=$(($NMOLCHECK - $POLYEGNMOL))
		HEGNMOL=$(($HEGNMOL - $MORE))
		NMOLCHECK=$(($MEGNMOL + $DEGNMOL + $REGNMOL + $TEGNMOL + $PEGNMOL +
$XEGNMOL + $HEGNMOL + $OEGNMOL + $NEGNMOL))
	echo "removed $MORE molecules of HEG to achieve requested number of molecules"

	elif [ $NMOLCHECK \< $POLYEGNMOL ]; then
		LESS=$(($POLYEGNMOL - $NMOLCHECK))
		DEGNMOL=$(($DEGNMOL + $LESS))
		NMOLCHECK=$(($MEGNMOL + $DEGNMOL + $REGNMOL + $TEGNMOL + $PEGNMOL +
$XEGNMOL + $HEGNMOL + $OEGNMOL + $NEGNMOL))
	echo "added $LESS molecules of DEG to achieve requested number of molecules"

	else
		echo "successfully calculated molecule numbers from mole fractions with no
changes"
	fi

	if [ $NMOLCHECK != $POLYEGNMOL ]; then
		echo "error with mole fractions computation"
		exit 1
	fi

	echo "MEGNMOL = $MEGNMOL, DEGNMOL = $DEGNMOL, REGNMOL = $REGNMOL, TEGNMOL =
$TEGNMOL, PEGNMOL = $PEGNMOL, XEGNMOL = $XEGNMOL, HEGNMOL = $HEGNMOL, OEGNMOL =
$OEGNMOL, NEGNMOL = $NEGNMOL, POLYEGNMOL = $NMOLCHECK"

fi
####END OF STEP

####lists for indexed loops
POLYA=("M" "D" "R" "T" "P" "X" "H" "O" "N")
POLYNUM=("$MEGNMOL" "$DEGNMOL" "$REGNMOL" "$TEGNMOL" "$PEGNMOL" "$XEGNMOL" "$HEGNMOL"
"$OEGNMOL" "$NEGNMOL")
POLYS=("MEGNMOL" "DEGNMOL" "REGNMOL" "TEGNMOL" "PEGNMOL" "XEGNMOL" "HEGNMOL"
"OEGNMOL" "NEGNMOL")
POLYNAME=("MEG" "DEG" "REG" "TEG" "PEG" "XEG" "HEG" "OEG" "NEG")
VARIABLES=("BASENAME" "MODEL" "TEMP" "FORCEFIELD" "DENSITY" "TOTALNMOL" "STOCKMIX"
"MEGNMOL" "DEGNMOL" "REGNMOL" "TEGNMOL" "PEGNMOL" "XEGNMOL" "HEGNMOL" "OEGNMOL"
"NEGNMOL" "POLYEGNMOL" "WATERNMOL")
VALUES=("$BASENAME" "$MODEL" "$TEMP" "$FORCEFIELD" "$DENSITY" "$TOTALNMOL"
"$STOCKMIX" "$MEGNMOL" "$DEGNMOL" "$REGNMOL" "$TEGNMOL" "$PEGNMOL" "$XEGNMOL"
"$HEGNMOL" "$OEGNMOL" "$NEGNMOL" "$POLYEGNMOL" "$WATERNMOL")
#18 variables
####END OF STEP

#####check inputted variables
#are all variables defined
index=0
while [ $index -lt 18 ]; do
	if [ "${VALUES[$index]}" = "" ] ; then
		echo "${VARIABLES[$index]} was not defined"
		exit 1
	fi
((index++))
```

```
done

#was a valid model selected
if [ $MODEL != "bulk" ] && [ $MODEL != "spce" ] && [ $MODEL != "tip4p2005" ]; then
      echo "You have chosen an unsupported model. Please correct -m input"
      exit 1
fi

#was a valid forcefield selected
if [ $FORCEFIELD != "OPLS" ] && [ $FORCEFIELD != "OPLSMOD" ]; then
      echo "You have chosen an unsupported forcefield. Please correct -f input"
      exit 1
fi

#density cannot be 0
if [ $DENSITY = 0 ]; then
      echo "density must be nonzero. Please correct -d input"
      exit 1
fi

#do molecule totals add up correctly
if [ $(( $POLYEGNMOL + $WATERNMOL )) -ne $TOTALNMOL ]; then
        echo "Sum of PolyEG and water molecules is not equal to total number of
molecules specified. Please correct input."
        exit 1
fi

#was a valid stock mixture selected
if [ $STOCKMIX != "none" ] && [ $STOCKMIX != "PEG200" ]; then
      echo "You have chosen an unsupported stock mixture. Please correct -s input."
      exit 1
fi

#do molecule totals add up correctly
if [ $(( $MEGNMOL + $DEGNMOL + $REGNMOL + $TEGNMOL + $PEGNMOL + $XEGNMOL + $HEGNMOL +
$OEGNMOL + $NEGNMOL )) -ne $POLYEGNMOL ]; then
      echo "Sum of polyethylene glycol molecules does not equal total specified with
-p. Please correct input."
      exit 1
fi

#does number of water molecules match model
if [ $MODEL = "bulk" ] && [ $WATERNMOL -ne 0 ]; then
      echo "bulk model indicates no water molecules. Please correct either -m or -w
input."
      exit 1
elif [ $MODEL != "bulk" ] && [ $WATERNMOL = 0 ]; then
      echo "conflicting information from -m and -w"
      exit 1
else
      echo ""
fi

#export variables to be used by the analysis script
cp ~/BrockportMDFiles/shell_scripts/variables.txt $WORKDIR
index=0
while [ $index -lt 18 ]; do
```

```
      sed -i "/${VARIABLES[$index]}/s/${VARIABLES[$index]}/${VALUES[$index]}/g"
"$WORKDIR"/variables.txt
      ((index++))
done
####END OF STEP

####create top file
# to understand sed replacing, see point 33 at https://catonmat.net/sed-one-liners-
explained-part-one
# note sed -i means editing existing files (no backup)

if [ $FORCEFIELD = "OPLS" ]; then
      MOLEDIR=/home/mmh/BrockportMDFiles/mixture_start_files
      cp "$MOLEDIR"/top_mixtures.top "$WORKDIR"/top_${BASENAME}.top
else
      MOLEDIR=/home/mmh/BrockportMDFiles/modified_mixture_start_files
      cp "$MOLEDIR"/top_mod_mixtures.top "$WORKDIR"/top_${BASENAME}.top
fi

#set molecule numbers for oligomers and remove unused oligomers
index=0
while [ $index -lt 9 ]; do
      if [ "${POLYNUM[$index]}" != "0" ]; then
            sed -i "/"${POLYS[$index]}"/s/"${POLYS[$index]}"/"${POLYNUM[$index]}"/g"
"$WORKDIR"/top_${BASENAME}.top
      else
            sed -i "/"${POLYS[$index]}"/d" "$WORKDIR"/top_${BASENAME}.top
      fi
      ((index++))
done

#set water molecules for model used and remove unused models
if [ $WATERNMOL -eq 0 ]; then
      sed -i "/WATERNMOL/d" "$WORKDIR"/top_${BASENAME}.top
elif [ $MODEL = "tip4p2005" ]; then
      sed -i "/TIP4P/s/WATERNMOL/$WATERNMOL/g" "$WORKDIR"/top_${BASENAME}.top
      sed -i "/SPCE/d" "$WORKDIR"/top_${BASENAME}.top
else
      sed -i "/SPCE/s/WATERNMOL/$WATERNMOL/g" "$WORKDIR"/top_${BASENAME}.top
      sed -i "/TIP4P/d" "$WORKDIR"/top_${BASENAME}.top
fi

####END OF STEP

####use insert-molecules to create start.gro
# setting molecular weights and scaling of density
# molecules inserted into a box such that [BOXDENSITY] = [DENSITY] * [SCALE]
AVAGADRO=$(echo "6.02214076 * 10^23" | bc)

#molecular weight and density scale for each PolyEG and water
#MEG
      MWM=62.0676
      DSCALEM=0.65
#DEG
      MWD=106.12
      DSCALED=0.60
#REG
```

```
      MWR=150.1724
      DSCALER=0.55
#TEG
      MWT=194.2248
      DSCALET=0.50
#PEG
      MWP=238.2772
      DSCALEP=0.45
#XEG
      MWX=282.3296
      DSCALEX=0.40
#HEG
      MWH=326.3820
      DSCALEH=0.35
#OEG
      MWO=370.4344
      DSCALEO=0.30
#NEG
      MWN=414.4868
      DSCALEN=0.25
#water
      MWW=18.02
      DSCALEW=0.9

# bc does not handle fractional powers; use exp(ln(ans)/3) to get ans^(1/3) with math
library (-l)
#formula for single oligomers:
#BOXSIDE=$(echo "scale=10; e( l(($TOTALNMOL * $MW * 10^21)/($AVAGADRO * $DENSITY *
$DENSITYSCALE))/3 )" | bc -l )  # in nm
BOXSIDE=$(echo "scale=10; e( l( ((($MEGNMOL * $MWM)/$DSCALEM) + (($DEGNMOL *
$MWD)/$DSCALED) + (($REGNMOL * $MWR)/$DSCALER) + (($TEGNMOL * $MWT)/$DSCALET) +
(($PEGNMOL * $MWP)/$DSCALEP) + (($XEGNMOL * $MWX)/$DSCALEX) + (($HEGNMOL *
$MWH)/$DSCALEH) + (($OEGNMOL * $MWO)/$DSCALEO) + (($NEGNMOL * $MWN)/$DSCALEN) +
(($WATERNMOL * $MWW)/$DSCALEW)) * (10^21/($AVAGADRO * $DENSITY)) )/3 )" | bc -l)
echo "The dimensions of the box for gmx insert-molecules is $BOXSIDE nm x $BOXSIDE nm
x $BOXSIDE nm."

#test
index=0
while [ $index -lt 9 ] ; do
if [ "${POLYNUM[$index]}" != "0" ]; then
echo " "${POLYNAME[$index]}"="${POLYNUM[$index]}" "
fi
((index++))
done

# insert molecules with final output saved at start.gro
mkdir $WORKDIR/gro_intermediates
index=0
intermediate=1
while  [ $index -lt 9 ]; do
      if [ "${POLYNUM[$index]}" != "0" ]; then
            if [ -e $WORKDIR/gro_intermediates/intermediate_$(expr $intermediate -
1).gro ]; then
                  gmx insert-molecules \
                  -ci $MOLEDIR/single_"${POLYNAME[$index]}".gro \
```

```
                -f $WORKDIR/gro_intermediates/intermediate_$(expr $intermediate -
1).gro \
                -o $WORKDIR/gro_intermediates/intermediate_${intermediate}.gro \
                -nmol "${POLYNUM[$index]}" \
                -try 10000
                ((intermediate++))
            else
                gmx insert-molecules \
                -ci $MOLEDIR/single_"${POLYNAME[$index]}".gro \
                -o $WORKDIR/gro_intermediates/intermediate_${intermediate}.gro \
                -nmol "${POLYNUM[$index]}" \
                -box $BOXSIDE $BOXSIDE $BOXSIDE \
                -try 10000
                ((intermediate++))
            fi
        fi
        ((index++))
done

if [ $WATERNMOL -gt 0 ]; then
        if [ -e $WORKDIR/gro_intermediates/intermediate_$(expr $intermediate - 1).gro
]; then
            gmx insert-molecules \
            -ci $MOLEDIR/single_${MODEL}.gro \
            -f $WORKDIR/gro_intermediates/intermediate_$(expr $intermediate - 1).gro \
            -o $WORKDIR/gro_intermediates/intermediate_${intermediate}.gro \
            -nmol $WATERNMOL \
            -try 10000
            ((intermediate++))
        else
            gmx insert-molecules \
            -ci $MOLEDIR/single_${MODEL}.gro \
            -o $WORKDIR/gro_intermediates/intermediate_${intermediate}.gro \
            -nmol $WATERNMOL \
            -box $BOXSIDE $BOXSIDE $BOXSIDE \
            -try 10000
            ((intermediate++))
        fi
fi
mv $WORKDIR/gro_intermediates/intermediate_$(expr $intermediate - 1).gro
$WORKDIR/start.gro

####END OF STEP

if [ -e $WORKDIR/start.gro ] && [ -e $WORKDIR/top_${BASENAME}.top ] && [ -e
"$WORKDIR"/em.mdp ];then
        echo "starting energy minimization"
else
        echo "missing one or more files needed to start energy minimization"
        exit 1
fi

####energy minimization
mkdir -p "$WORKDIR"/1_EM/out
EM="$WORKDIR"/1_EM
mv "$WORKDIR"/em.mdp "$EM"
mv "$WORKDIR"/start.gro "$EM"
```

```
gmx grompp \
      -f "$EM"/em.mdp \
      -c "$EM"/start.gro \
      -p "$WORKDIR"/top_${BASENAME}.top \
      -o "$EM"/em.tpr \
      -po "$EM"/out/mdout.mdp

gmx mdrun \
      -s "$EM"/em.tpr \
      -c "$EM"/out/em.gro \
      -o "$EM"/out/traj.trr \
      -e "$EM"/out/energy.edr \
      -g "$EM"/out/log.log \
      -pme cpu -pmefft cpu -update cpu -bonded cpu -nb gpu

# energy minimization potential energy analysis
      mkdir -p "$EM"/analysis
      echo "Potential" | gmx energy -f "$EM"/out/energy.edr -o
"$EM"/analysis/potential.xvg
##### END STEP

##### NPT equilibration run
mkdir -p "$WORKDIR"/2_equil_NPT_${TEMP}K/out
EQUIL="$WORKDIR"/2_equil_NPT_${TEMP}K
cp "$EM"/out/em.gro "$EQUIL"
mv "$WORKDIR"/eq_NPT_${TEMP}K.mdp "$EQUIL"

if [ -e $EQUIL/em.gro ] && [ -e $WORKDIR/top_${BASENAME}.top ] && [ -e
"$EQUIL"/eq_NPT_${TEMP}K.mdp ];then
      echo "starting equilibration"
else
      echo "missing one or more files needed to start equilibration"
      exit 1
fi

gmx grompp \
      -f "$EQUIL"/eq_NPT_${TEMP}K.mdp  \
      -c "$EQUIL"/*.gro \
      -p "$WORKDIR"/top_${BASENAME}.top \
      -o "$EQUIL"/npt.tpr \
      -po "$EQUIL"/out/mdout.mdp \
      -maxwarn 1

gmx mdrun \
      -s "$EQUIL"/npt.tpr \
      -c "$EQUIL"/out/npt.gro \
      -x "$EQUIL"/out/traj.xtc \
      -e "$EQUIL"/out/energy.edr \
      -g "$EQUIL"/out/log.log \
      -cpo "$EQUIL"/out/npt.cpt \
      -cpt 1 \
      -pme cpu -pmefft cpu -update cpu -bonded cpu -nb gpu -v

# NPT equilibration energy analysis
mkdir -p "$EQUIL"/analysis
```

```
echo "Potential"   | gmx energy -f "$EQUIL"/out/energy.edr -o
"$EQUIL"/analysis/potential.xvg
echo "Temperature" | gmx energy -f "$EQUIL"/out/energy.edr -o
"$EQUIL"/analysis/temperature.xvg
echo "Pressure"    | gmx energy -f "$EQUIL"/out/energy.edr -o
"$EQUIL"/analysis/pressure.xvg
echo "Volume"      | gmx energy -f "$EQUIL"/out/energy.edr -o
"$EQUIL"/analysis/volume.xvg
echo "Density"     | gmx energy -f "$EQUIL"/out/energy.edr -o
"$EQUIL"/analysis/density.xvg

# Choose frame from NPT of average density for NVT
# use automatic feature of boxcut.sh for cutting frame
bash /home/mmh/BrockportMDFiles/shell_scripts/boxcut.sh -a -p "$EQUIL"/
# only use manual cutting of frame if automatic fails
if [ ! -f "$EQUIL"/equilibrium.gro ]; then
      bash /home/mmh/BrockportMDFiles/shell_scripts/boxcut.sh -f 0.5 -p "$EQUIL"/
fi
##### END STEP

##### NVT production run

mkdir -p "$WORKDIR"/3_prod_NVT_${TEMP}K/out
PROD="$WORKDIR"/3_prod_NVT_${TEMP}K
cp "$EQUIL"/equilibrium.gro "$PROD"
mv "$WORKDIR"/md_NVT_${TEMP}K.mdp "$PROD"

if [ -e $PROD/equilibrium.gro ] && [ -e $WORKDIR/top_${BASENAME}.top ] && [ -e
"$PROD"/md_NVT_${TEMP}K.mdp ];then
      echo "starting production"
else
      echo "missing one or more files needed to start production"
      exit 1
fi

gmx grompp \
      -f "$PROD"/md_NVT_${TEMP}K.mdp \
      -c "$PROD"/equilibrium.gro \
      -p "$WORKDIR"/top_${BASENAME}.top \
      -o "$PROD"/md.tpr \
      -po "$PROD"/out/mdout.mdp

gmx mdrun \
      -s "$PROD"/md.tpr \
      -c "$PROD"/out/md.gro \
      -x "$PROD"/out/traj.xtc \
      -e "$PROD"/out/energy.edr \
      -g "$PROD"/out/log.log \
      -cpo "$PROD"/out/md.cpt \
      -cpt 1 \
      -pme cpu -pmefft cpu -update cpu -bonded cpu -nb gpu -v

##### END NVT

##### ANALYSIS

if [ -e "$PROD"/out/md.gro ];then
```

```
      echo "starting post-production analysis"
else
      echo "production did not finish, analysis cannot be done"
      exit 1
fi

cp ~/BrockportMDFiles/shell_scripts/analysis.sh $WORKDIR
bash analysis.sh
##### END ANALYSIS
```

5.5 Scripts for analyzing completed MD simulation of PEG200

Note these all rely on a small file called "variables" with the following content

```
b BASENAME
m MODEL
t TEMP
f FORCEFIELD
n TOTALNMOL
s STOCKMIX
1 MEGNMOL
2 DEGNMOL
3 REGNMOL
4 TEGNMOL
5 PEGNMOL
6 XEGNMOL
7 HEGNMOL
8 OEGNMOL
9 NEGNMOL
p POLYEGNMOL
w WATERNMOL
```

5.5.1 Python script file for handling analysis of radial distribution functions

```
"rdf_ave"
import numpy as np
import os
import shutil
#function to save xvg into arrays
#open the xvg, print needed info in txt
#nondata line start with # or @
#find returns -1 if false
#create an empty list to be appended
#count the number of lines to get the array size
#split each line into a list and save into another list
#create an empty array of the right size then fill with the list
#this is faster than appending an np array
#T switches rows and columns
#this will be necessary for averaging once all data is gathered
c = 0
def xvg_array (info):
```

```
    xvg = info + ".xvg"
    txt = info + ".txt"
    with open( xvg,'r') as file:
        lines = file.readlines()
    with open( txt,'w') as file:
        for line in lines:
            if line.find("#") != -1:
                pass
            elif line.find("@") != -1:
                pass
            else:
                file.write(line)
    rdf_file_l = []
    global c
    c = 0
    with open( txt, 'r') as file:
        lines = file.readlines()
        for line in lines:
            value = line.split()
            rdf_file_l.append(value)
            c = c + 1
    rdf_file_a = np.zeros((c,3))
    rdf_file_a = np.array(list(rdf_file_l), float)
    rdf_file_a = rdf_file_a.T
    return(rdf_file_a)

#runs xvg_array for each of ten files and saves into 1 array
#averages these and makes a new array containing the average
#write a new xvg file with the header from file 1
#then puts the averaged array in it
rdf_ave = np.array((0,0,0))
fold_rdf = os.environ["PROD"] + "/analysis/rdf/"
fold_int = fold_rdf + "intra/"
def averaging (residue):

    name_nmol = residue + "NMOL"
    nmol = int(os.environ[name_nmol])
    print(nmol)

    if nmol < 10:
        num = [num + 1 for num in range(nmol)]
        rdf_res_l = []
        rdf_res_a = np.zeros((nmol, 3, c))
        for i in num:
            inp = fold_int + "rdf_" + residue + "_intra_" + str(i)
            out = xvg_array(inp)
            rdf_res_l.append(out)
    else:
        num = [num + 1 for num in range(10)]
        rdf_res_l = []
        rdf_res_a = np.zeros((10, 3, c))
        for i in num:
            inp = fold_int + "rdf_" + residue + "_intra_" + str(i)
            out = xvg_array(inp)
            rdf_res_l.append(out)

    global rdf_ave
```

```
    rdf_res_a = np.array(list(rdf_res_l), float)
    rdf_ave = rdf_res_a.mean(axis = 0)
    rdf_ave = np.round(rdf_ave, 3)

    x = [x for x in range(c)]
    header = fold_int + "rdf_" + residue + "_intra_1.xvg"
    with open(header, 'r') as file:
        lines = file.readlines()
    avefile = fold_rdf + "rdf_" + residue + "_intra_ave.xvg"
    with open(avefile, 'w+') as file:
        for line in lines:
            if line.find("#") != -1:
                file.write(line)
            elif line.find("@") != -1:
                file.write(line)
        for v in x:
            dist = str(rdf_ave[0,v])
            alc = str(rdf_ave[1,v])
            ether = str(rdf_ave[2,v])
            content = "      " + dist + "    " + alc + "    " + ether + '\n'
            file.write(content)

#calculates inter_same by subtracting intra from total
#intra must be scaled by dividing by nmol
#then the header is copied from the total file
#and data added
fold_tot = fold_rdf + "total/"
def inter (residue):
    inp = fold_tot + "rdf_" + residue + "_total"
    rdf_total = xvg_array(inp)
    name_nmol = residue + "NMOL"
    nmol = int(os.environ[name_nmol])
    rdf_intra = (rdf_ave / nmol)
    rdf_inter = (rdf_total - rdf_intra)
    rdf_inter = np.round(rdf_inter, 3)

    x = [x for x in range(c)]
    header = fold_tot + "rdf_" + residue + "_total.xvg"
    with open(header, 'r') as file:
        lines = file.readlines()
    intfile = fold_rdf + "rdf_" + residue + "_inter_same.xvg"
    with open(intfile, 'w+') as file:
        for line in lines:
            if line.find("#") != -1:
                file.write(line)
            elif line.find("@") != -1:
                file.write(line)
        for v in x:
            dist = str(rdf_total[0,v])
            alc = str(rdf_inter[1,v])
            ether = str(rdf_inter[2,v])
            content = "      " + dist + "    " + alc + "    " + ether + '\n'
            file.write(content)

#input is provided through a shell file
#run both averaging and inter for each residue
res_name = input("residue?")
```

```
averaging(res_name)
inter(res_name)

“rdf_norm”
import numpy as np
import os
import shutil

#adaptation of rdf_ave.py
c = 0
def xvg_array (info):
    xvg = info + ".xvg"
    txt = info + ".txt"
    with open( xvg,'r') as file:
        lines = file.readlines()
    with open( txt,'w') as file:
        for line in lines:
            if line.find("#") != -1:
                pass
            elif line.find("@") != -1:
                pass
            else:
                file.write(line)
    rdf_file_l = []
    global c
    c = 0
    with open( txt, 'r') as file:
        lines = file.readlines()
        for line in lines:
            value = line.split()
            rdf_file_l.append(value)
            c = c + 1
    rdf_file_a = np.zeros((c,3))
    rdf_file_a = np.array(list(rdf_file_l), float)
    return(rdf_file_a)


fold_main = os.getcwd()
fold_rdf = fold_main + "/3_prod_NVT_328K/analysis/rdf/"
fold_int = fold_rdf + "intra/"

def normalize (residue):
#save the ave file into an array
    ave = fold_rdf + "rdf_" + residue + "_intra_ave"
    rdf_ave = xvg_array(ave)

#get the integrals
    int_alc = np.trapz(rdf_ave[:,1], x=rdf_ave[:,0])
    int_ether = np.trapz(rdf_ave[:,2], x=rdf_ave[:,0])

#make a new file for normalized intra
    x = [x for x in range(c)]
    header = fold_rdf + "rdf_" + residue + "_intra_ave.xvg"
    with open(header, 'r') as file:
        lines = file.readlines()
    avefile = fold_rdf + "rdf_" + residue + "_intra_norm.xvg"
    with open(avefile, 'w+') as file:
```

```
        for line in lines:
            if line.find("#") != -1:
                file.write(line)
            elif line.find("@") != -1:
                file.write(line)
#these lines write the array into an xvg
#normalize with integral as they are entered
        for v in x:
            dist = str(rdf_ave[v,0])
            alc = str(round(rdf_ave[v,1] / int_alc , 3))
            ether = str(round(rdf_ave[v,2] / int_ether, 3))
            content = "      " + dist + "    " + alc + "    " + ether + '\n'
            file.write(content)

res_name = input("residue?")
normalize(res_name)
```

5.5.2 Bash script file for analysis from NPT run

"equil_analysis"

```
#!/bin/bash

usage () {
      echo
      echo "Script file made for GROMACS 2020.4 version compatibility."
      echo "This bash file must be in the desired working directory."
      echo "Usage:"
      echo "   -c [string]     convergence time: time in ps density converges
(overestimate)"
      echo
      exit 1
      }

while getopts ":c:h" options; do
      case ${options} in
            c)
                  CONVERGE=$OPTARG ;;
            h)
                  usage ;;
            \?)
                  echo "Invalid input. Unknown flag. Refer to the help with -h"
                  exit 1 ;;
            :)
                  echo "Invalid input: -$OPTARG requires an argument. Refer to the
help with -h"
                  exit 1 ;;
      esac
done

if [ $OPTIND -eq 1 ]; then
      echo "please specify convergence time with -c"
      exit 1
fi
```

```
#fetch variables from variables.txt
#if variables.txt was not created by another program it must be created and placed in
the workdir
#copy and edit the file from the shell scripts folder

while read -r flag value ;do
      case $flag in
            b)
                  BASENAME=$value ;;
            m)
                  MODEL=$value ;;
            t)
                  TEMP=$value ;;
            f)
                  FORCEFIELD=$value ;;
            n)
                  TOTALNMOL=$value ;;
            s)
                  STOCKMIX=$value ;;
            1)
                  MEGNMOL=$value ;;
            2)
                  DEGNMOL=$value ;;
            3)
                  REGNMOL=$value ;;
            4)
                  TEGNMOL=$value ;;
            5)
                  PEGNMOL=$value ;;
            6)
                  XEGNMOL=$value ;;
            7)
                  HEGNMOL=$value ;;
            8)
                  OEGNMOL=$value ;;
            9)
                  NEGNMOL=$value ;;
            p)
                  POLYEGNMOL=$value ;;
            w)
                  WATERNMOL=$value ;;
      esac
done < variables.txt

VALUES=("$BASENAME" "$MODEL" "$TEMP" "$FORCEFIELD" "$TOTALNMOL" "$STOCKMIX"
"$MEGNMOL" "$DEGNMOL" "$REGNMOL" "$TEGNMOL" "$PEGNMOL" "$XEGNMOL" "$HEGNMOL"
"$OEGNMOL" "$NEGNMOL" "$POLYEGNMOL" "$WATERNMOL")
VARIABLES=("BASENAME" "MODEL" "TEMP" "FORCEFIELD" "TOTALNMOL" "STOCKMIX" "MEGNMOL"
"DEGNMOL" "REGNMOL" "TEGNMOL" "PEGNMOL" "XEGNMOL" "HEGNMOL" "OEGNMOL" "NEGNMOL"
"POLYEGNMOL" "WATERNMOL")
#check that all requested variables are defined
if [ -e variables.txt ]; then
      echo "variables.txt exists."
      index=0
      while [ $index -lt 17 ]; do
            echo " ${VARIABLES[$index]} = ${VALUES[$index]}"
            if [ "${VALUES[$index]}" = "" ] ; then
```

```
                echo "${VARIABLES[$index]} was not defined"
                exit 1
            fi
        ((index++))
        done
else
        echo "variables.txt does not exist"
        exit 1
fi

WORKDIR="$(dirname "$(readlink -f "$0")")"
EM="$WORKDIR"/1_EM
EQUIL="$WORKDIR"/2_equil_NPT_${TEMP}K
PROD="$WORKDIR"/3_prod_NVT_${TEMP}K

#lists for indexed loops
POLYA=("M" "D" "R" "T" "P" "X" "H" "O" "N")
POLYNUM=("$MEGNMOL" "$DEGNMOL" "$REGNMOL" "$TEGNMOL" "$PEGNMOL" "$XEGNMOL" "$HEGNMOL"
"$OEGNMOL" "$NEGNMOL")
POLYS=("MEGNMOL" "DEGNMOL" "REGNMOL" "TEGNMOL" "PEGNMOL" "XEGNMOL" "HEGNMOL"
"OEGNMOL" "NEGNMOL")
POLYNAME=("MEG" "DEG" "REG" "TEG" "PEG" "XEG" "HEG" "OEG" "NEG")

#get average density
gmx analyze -f "$EQUIL"/analysis/density.xvg -b $CONVERGE >>
"$EQUIL"/analysis/density.txt

#fluctuation properties
(echo "Volume" && echo "Temperature" && echo "Enthalpy") | gmx energy\
        -f "$EQUIL"/out/energy.edr\
        -o "$EQUIL"/analysis/fluct.xvg\
        -nmol $TOTALNMOL\
        -fluct_props -driftcorr \
        -b $CONVERGE >> "$EQUIL"/analysis/fluctuation.txt

#dielectric constant
echo "0" | gmx dipoles\
        -f "$EQUIL"/out/traj.xtc\
        -s "$EQUIL"/npt.tpr\
        -temp $TEMP\
        -o "$EQUIL"/analysis/Mtot.xvg\
        -d "$EQUIL"/analysis/dipsist.xvg\
        -eps "$EQUIL"/analysis/epsilon.xvg\
        -a "$EQUIL"/analysis/aver.xvg >> "$EQUIL"/analysis/epsilon.txt

#dipole moment
mkdir $EQUIL/analysis/dipole

echo "q" | gmx make_ndx \
        -f $EQUIL/npt.tpr\
        -o $EQUIL/analysis/dipole/dipole.ndx

dip_group=2
index=0
while [ $index -lt 9 ]; do
        if [ "${POLYNUM[$index]}" != "0" ] ; then
            echo "$dip_group" | gmx dipoles\
```

```
                  -f $EQUIL/out/traj.xtc\
                  -s $EQUIL/npt.tpr\
                  -n $EQUIL/analysis/dipole/dipole.ndx\
                  -temp $TEMP\
                  -eps $EQUIL/analysis/dipole/epsilon_"${POLYNAME[$index]}".xvg\
                  -o $EQUIL/analysis/dipole/Mtot_"${POLYNAME[$index]}".xvg\
                  -d $EQUIL/analysis/dipole/dipdist_"${POLYNAME[$index]}".xvg\
                  -a $EQUIL/analysis/dipole/aver_"${POLYNAME[$index]}".xvg
            echo "${POLYNAME[$index]}" >> $EQUIL/analysis/dipole.txt
            gmx analyze -f $EQUIL/analysis/dipole/epsilon_"${POLYNAME[$index]}".xvg >> 
$EQUIL/analysis/dipole.txt
            ((dip_group++))
      fi
((index++))
done

if [ $WATERNMOL != 0 ]; then
      echo "$dip_group" | gmx dipoles\
            -f $EQUIL/out/traj.xtc\
            -s $EQUIL/npt.tpr\
            -n $EQUIL/analysis/dipole/dipole.ndx\
            -temp $TEMP\
            -eps $EQUIL/analysis/dipole/epsilon_water.xvg\
            -d $EQUIL/analysis/dipole/dipdist_water.xvg\
            -o $EQUIL/analysis/dipole/Mtot_water.xvg\
            -a $EQUIL/analysis/dipole/aver_water.xvg
      echo "water" >> $EQUIL/analysis/dipole.txt
      gmx analyze -f $EQUIL/analysis/dipole/epsilon_water.xvg >> 
$EQUIL/analysis/dipole.txt
      ((dip_group++))
fi
```

5.5.3 Bash script for analysis from NVT production run

```
"analysis"

#!/bin/bash

#fetch variables from variables.txt
#if variables.txt was not created by another program it must be created and placed in 
the workdir
#copy and edit the file from the shell scripts folder
while read -r flag value ;do
      case $flag in
            b)
                  BASENAME=$value ;;
            m)
                  MODEL=$value ;;
            t)
                  TEMP=$value ;;
            f)
                  FORCEFIELD=$value ;;
            n)
                  TOTALNMOL=$value ;;
            s)
```

```
                  STOCKMIX=$value ;;
            1)
                  MEGNMOL=$value ;;
            2)
                  DEGNMOL=$value ;;
            3)
                  REGNMOL=$value ;;
            4)
                  TEGNMOL=$value ;;
            5)
                  PEGNMOL=$value ;;
            6)
                  XEGNMOL=$value ;;
            7)
                  HEGNMOL=$value ;;
            8)
                  OEGNMOL=$value ;;
            9)
                  NEGNMOL=$value ;;
            p)
                  POLYEGNMOL=$value ;;
            w)
                  WATERNMOL=$value ;;
      esac
done < variables.txt

#check that all requested variables are defined
VALUES=("$BASENAME" "$MODEL" "$TEMP" "$FORCEFIELD" "$TOTALNMOL" "$STOCKMIX"
"$MEGNMOL" "$DEGNMOL" "$REGNMOL" "$TEGNMOL" "$PEGNMOL" "$XEGNMOL" "$HEGNMOL"
"$OEGNMOL" "$NEGNMOL" "$POLYEGNMOL" "$WATERNMOL")
VARIABLES=("BASENAME" "MODEL" "TEMP" "FORCEFIELD" "TOTALNMOL" "STOCKMIX" "MEGNMOL"
"DEGNMOL" "REGNMOL" "TEGNMOL" "PEGNMOL" "XEGNMOL" "HEGNMOL" "OEGNMOL" "NEGNMOL"
"POLYEGNMOL" "WATERNMOL")

if [ -e variables.txt ]; then
      echo "variables.txt exists."
      index=0
      while [ $index -lt 17 ]; do
            if [ "${VALUES[$index]}" = "" ] ; then
                  echo "${VARIABLES[$index]} is not defined in variables.txt"
                  exit 1
            fi
      ((index++))
      done
else
      echo "variables.txt does not exist"
      exit 1
fi

WORKDIR="$(dirname "$(readlink -f "$0")")"
EM="$WORKDIR"/1_EM
PROD="$WORKDIR"/3_prod_NVT_${TEMP}K

#lists for indexed loops
POLYA=("M" "D" "R" "T" "P" "X" "H" "O" "N")
POLYNUM=("$MEGNMOL" "$DEGNMOL" "$REGNMOL" "$TEGNMOL" "$PEGNMOL" "$XEGNMOL" "$HEGNMOL"
"$OEGNMOL" "$NEGNMOL")
```

```
POLYS=("MEGNMOL" "DEGNMOL" "REGNMOL" "TEGNMOL" "PEGNMOL" "XEGNMOL" "HEGNMOL"
"OEGNMOL" "NEGNMOL")
POLYNAME=("MEG" "DEG" "REG" "TEG" "PEG" "XEG" "HEG" "OEG" "NEG")

#counting: move past index groups for the systems, PEG, and water, as they will not
be used
#$start is the first customized index group
start=2
index=0
while [ $index -lt 9 ]; do
      if [ "${POLYNUM[$index]}" != "0" ] ; then
            ((start++))
      fi
((index++))
done

if [ ${WATERNMOL} -gt 0 ]; then
      ((start++))
fi

##### ANALYSIS
# NVT production energy, mean squared displacement, and green-kubo/einstein viscosity
analysis
mkdir -p "$PROD"/analysis
echo "Potential"   | gmx energy -f "$PROD"/out/energy.edr -o
"$PROD"/analysis/potential.xvg
echo "Temperature" | gmx energy -f "$PROD"/out/energy.edr -o
"$PROD"/analysis/temperature.xvg
echo "Pressure"    | gmx energy -f "$PROD"/out/energy.edr -o
"$PROD"/analysis/pressure.xvg

###MSD
# gmx msd for the system, all individual oligomers, water, and the PolyEG as a group
#repeat calculations will occur if system is water
#run gmx msd for the system
echo "0" | gmx msd -f "$PROD"/out/traj.xtc -s "$PROD"/md.tpr -o
"$PROD"/analysis/msd_system.xvg
#run gmx msd for each oligomer
#variable concantenation for indexing: \n is used as a line break. the variable
concantenates with itself until all inputs are gathered
#then the variable is used as the input to make the index file
index=0
msdgroup=2
msd=" "
while [ $index -lt 9 ]; do
      if [ "${POLYNUM[$index]}" != "0" ] ; then
            echo "$msdgroup" | gmx msd -f "$PROD"/out/traj.xtc -s "$PROD"/md.tpr -o
"$PROD"/analysis/msd_"${POLYNAME[$index]}".xvg
            msd="${msd}r ${POLYNAME[index]} | "
            ((msdgroup++))
      fi
      ((index++))
done

#run gmx msd for water
if [ $WATERNMOL -gt 0 ]; then
```

```
      echo "$msdgroup" | gmx msd -f "$PROD"/out/traj.xtc -s "$PROD"/md.tpr -o
"$PROD"/analysis/msd_${MODEL}.xvg
      ((msdgroup++))
fi

#index Polyeg and run gmx msd on new group
if [ $POLYEGNMOL -gt 0 ]; then
      if [ $MODEL != "bulk" ]; then
            echo -e  "$msd \n q" | gmx make_ndx -f "$EM"/start.gro -o
"$PROD"/analysis/msd.ndx
            echo "$msdgroup" | gmx msd -f "$PROD"/out/traj.xtc -s "$PROD"/md.tpr -n
"$PROD"/analysis/msd.ndx -o "$PROD"/analysis/msd_PolyEG.xvg
      fi
fi

### END MSD

###DISTANCE
#distance index
index=0
distance=" "
while [ $index -lt 9 ]; do
      if [ "${POLYNUM[$index]}" != "0" ] ; then
      distance="${distance}a OA${POLYA[index]}1 | a OA${POLYA[index]}2 \n "
      fi
      ((index++))
done
echo -e "${distance} q" | gmx make_ndx -f "$EM"/start.gro -o
"$PROD"/analysis/distance.ndx

#run gmx distance and save to a txt
#save the output of a command to a text file: command >> output.txt (use > to
overwrite the file)
#similarly: << for an input file
index=0
group=$start
while [ $index -lt 9 ]; do
      if [ "${POLYNUM[$index]}" != "0" ] ; then
      gmx distance\
            -f "$PROD"/out/traj.xtc\
            -s "$PROD"/md.tpr\
            -n "$PROD"/analysis/distance.ndx\
            -oav "$PROD"/analysis/distance_"${POLYNAME[$index]}".xvg\
            -select $group >> "$PROD"/analysis/distance.txt
      ((group++))
      fi
((index++))
done
###END DISTANCE

###RDF
mkdir $PROD/analysis/rdf
mkdir $PROD/analysis/rdf/total
#intermolecular total
group=$start
index=0
rdf=" "
```

```
while [ $index -lt 9 ]; do
      if [ "${POLYNUM[$index]}" != "0" ] ; then
      rdf="${rdf}a OA${POLYA[$index]}1 \n a OA${POLYA[$index]}2 \n"
      ((group += 2))
            if [ ${POLYNAME[$index]} != "MEG" ]; then
            ether="t peg_OE & r ${POLYNAME[$index]}\n name $group
OE_${POLYNAME[$index]}\n"
            rdf="${rdf}${ether}"
            ((group++))
            fi
      fi
((index++))
done
echo -e "${rdf}\n q" | gmx make_ndx\
      -f "$PROD"/md.tpr\
      -o "$PROD"/analysis/rdf/rdf_total.ndx
#echo -e "rdf input:$rdf"

mkdir "$PROD"/analysis/rdf/"$BASENAME"_pngs

group=$start
index=0
while [ $index -lt 9 ]; do
      if [ "${POLYNUM[$index]}" != "0" ] ; then
            gmx rdf\
                  -f "$PROD"/out/traj.xtc\
                  -s "$PROD"/md.tpr\
                  -n "$PROD"/analysis/rdf/rdf_total.ndx\
                  -o "$PROD"/analysis/rdf/total/rdf_${POLYNAME[$index]}_total.xvg\
                  -ref $group -sel $(expr $group + 1) $(expr $group + 2)
            ((group += 3))
      fi
((index++))
done

mkdir $PROD/analysis/rdf/intra
#intramolecular
group=$start
index=0
rdf=" "
offset=0
while [ $index -lt 9 ]; do
      if [ "${POLYNUM[$index]}" != "0" ]; then
            mol=1
            if [ ${POLYNUM[$index]} -lt 10 ]; then
                  while [ $mol -le ${POLYNUM[$index]} ]; do
                  num=$(($mol + $offset))
                  rdf="${rdf}a OA${POLYA[$index]}1 & ri "$num"\n name $group
OA"${POLYA[$index]}"1_m"$num"\n"
                  ((group++))
                  rdf="${rdf}a OA${POLYA[$index]}2 & ri "$num"\n name $group
OA"${POLYA[$index]}"2_m"$num"\n"
                  ((group++))
                        if [ ${POLYNAME[$index]} != "MEG" ]; then
                        ether="t peg_OE & r ${POLYNAME[$index]} &ri "$num"\n name
$group OE_"${POLYNAME[$index]}"_m"$num"\n"
                        ((group++))
```

```
                        rdf="${rdf}${ether}"
                        fi
                  ((mol++))
                  done
            else
                  while [ $mol -le 10 ]; do
                  num=$(($mol + $offset))
                  rdf="${rdf}a OA${POLYA[$index]}1 & ri "$num"\n name $group
OA"${POLYA[$index]}"1_m"$num"\n"
                  ((group++))
                  rdf="${rdf}a OA${POLYA[$index]}2 & ri "$num"\n name $group
OA"${POLYA[$index]}"2_m"$num"\n"
                  ((group++))
                        if [ ${POLYNAME[$index]} != "MEG" ]; then
                        ether="t peg_OE & r ${POLYNAME[$index]} &ri "$num"\n name
$group OE_"${POLYNAME[$index]}"_m"$num"\n"
                        ((group++))
                        rdf="${rdf}${ether}"
                        fi
                  ((mol++))
                  done
            fi
      offset=(${offset}+${POLYNUM[$index]})
      fi
((index++))
done
echo -e "${rdf}\n q" | gmx make_ndx -f "$PROD"/md.tpr -o
"$PROD"/analysis/rdf/rdf_intra.ndx
#echo -e "rdf intra input:\n$rdf"

group=$start
index=0
while [ $index -lt 9 ]; do
      if [ "${POLYNUM[$index]}" != "0" ] ; then
      mol=1
            if [ ${POLYNUM[$index]} -lt 10 ]; then
                  while [ $mol -le ${POLYNUM[$index]} ]; do
                  gmx rdf\
                        -f "$PROD"/out/traj.xtc\
                        -s "$PROD"/md.tpr\
                        -n "$PROD"/analysis/rdf/rdf_intra.ndx\
                        -o
$PROD/analysis/rdf/intra/rdf_${POLYNAME[$index]}_intra_${mol}.xvg\
                        -ref $group -sel $(expr $group + 1) $(expr $group + 2)
                  ((group += 3))
                  ((mol++))
                  done
            else
                  while [ $mol -le 10 ]; do
                  gmx rdf\
                        -f "$PROD"/out/traj.xtc\
                        -s "$PROD"/md.tpr\
                        -n "$PROD"/analysis/rdf/rdf_intra.ndx\
                        -o
$PROD/analysis/rdf/intra/rdf_${POLYNAME[$index]}_intra_${mol}.xvg\
                        -ref $group -sel $(expr $group + 1) $(expr $group + 2)
                  ((group += 3))
```

```
                  ((mol++))
                  done
            fi
      fi
((index++))
done

#intermolecular interresidual

#makes a list of all OA1 atoms present
#ie OAD1 OAR1 etc
index=0
OA1=" "
while [ $index -lt 9 ]; do
      if [ "${POLYNUM[$index]}" != "0" ] ; then
      OA1="${OA1}a OA${POLYA[$index]}1|"
      fi
((index++))
done

#ref group OA1 for the residue
#sel group 1 ethers in other residues
#sel group 2 OA1 atoms in other residues
group=$start
index=0
rdf=" "
while [ $index -lt 9 ]; do
      if [ "${POLYNUM[$index]}" != "0" ] ; then
      rdf="${rdf}a OA${POLYA[$index]}1 \n"
      ((group++))
      ether="t peg_OE &! r ${POLYNAME[$index]}\n name $group
OE_!"${POLYNAME[$index]}"\n"
      ((group++))
      rdf="${rdf}${ether}${OA1}&! r ${POLYNAME[$index]}\n name $group
OA1_!"${POLYNAME[$index]}"\n"
      ((group++))
      fi
((index++))
done
#echo -e "rdf inter_dif input:\n$rdf"
echo -e "${rdf}\n q" | gmx make_ndx -f "$PROD"/md.tpr -o
"$PROD"/analysis/rdf/rdf_inter_dif.ndx

group=$start
index=0
while [ $index -lt 9 ]; do
      if [ "${POLYNUM[$index]}" != "0" ] ; then
            gmx rdf\
                  -f "$PROD"/out/traj.xtc\
                  -s "$PROD"/md.tpr\
                  -n "$PROD"/analysis/rdf/rdf_inter_dif.ndx\
                  -o $PROD/analysis/rdf/rdf_${POLYNAME[$index]}_inter_dif.xvg\
                  -ref $group -sel $(expr $group + 1) $(expr $group + 2)
            ((group += 3))
            xmgrace\
                  -nxy $PROD/analysis/rdf/rdf_${POLYNAME[$index]}_inter_dif.xvg\
                  -hdevice PNG -hardcopy\
```

```
                  -printfile
$PROD/analysis/rdf/"$BASENAME"_pngs/rdf_${POLYNAME[$index]}_inter_dif.png
      fi
((index++))
done

#begin python analysis
#rdf_ave.py:
#creates an xvg that is the average of the 10 intra files
#also subtracts intra from total to give the inter_same graph
#rdf_norm.py
#creates a normalized intra average from intra_ave.xvg

#export variables python needs from this script
export PROD

#the python code asks the user to input the residue acronym.
#input only residues which are present or the code will error
index=0
while [ $index -lt 9 ]; do
export ${POLYS[index]}
      if [ "${POLYNUM[$index]}" != "0" ]; then
            echo "${POLYNAME[$index]}" | python3
~/BrockportMDFiles/python_scripts/rdf_ave.py
            echo "${POLYNAME[$index]}" | python3
~/BrockportMDFiles/python_scripts/rdf_norm.py
      fi
((index++))
done

#the newly constructed xvg files copy the header from an existing file. change the
data labels.
index=0
num=1
while [ $index -lt 9 ]; do
      if [ "${POLYNUM[$index]}" != "0" ]; then
            sed -i "/subtitle/s/OA${POLYA[$index]}1_m$num/OA${POLYA[$index]}1/g"
$PROD/analysis/rdf/rdf_${POLYNAME[$index]}_intra_ave.xvg
            sed -i "/s0/s/OA${POLYA[$index]}2_m$num/OA${POLYA[$index]}2/g"
$PROD/analysis/rdf/rdf_${POLYNAME[$index]}_intra_ave.xvg
            sed -i "/s1/s/OE_${POLYNAME[$index]}_m$num/OE_${POLYNAME[$index]}/g"
$PROD/analysis/rdf/rdf_${POLYNAME[$index]}_intra_ave.xvg
            sed -i "/subtitle/s/OA${POLYA[$index]}1_m$num/OA${POLYA[$index]}1/g"
$PROD/analysis/rdf/rdf_${POLYNAME[$index]}_intra_norm.xvg
            sed -i "/s0/s/OA${POLYA[$index]}2_m$num/OA${POLYA[$index]}2/g"
$PROD/analysis/rdf/rdf_${POLYNAME[$index]}_intra_norm.xvg
            sed -i "/s1/s/OE_${POLYNAME[$index]}_m$num/OE_${POLYNAME[$index]}/g"
$PROD/analysis/rdf/rdf_${POLYNAME[$index]}_intra_norm.xvg
            num=$(( $num + ${POLYNUM[$index]} ))
            xmgrace -nxy $PROD/analysis/rdf/rdf_${POLYNAME[$index]}_intra_ave.xvg\
                  -hdevice PNG -hardcopy\
                  -printfile
$PROD/analysis/rdf/"$BASENAME"_pngs/rdf_${POLYNAME[$index]}_intra_ave.png
            xmgrace -nxy $PROD/analysis/rdf/rdf_${POLYNAME[$index]}_inter_same.xvg\
                  -hdevice PNG -hardcopy\
                  -printfile
$PROD/analysis/rdf/"$BASENAME"_pngs/rdf_${POLYNAME[$index]}_inter_same.png
```

```
            xmgrace -nxy $PROD/analysis/rdf/rdf_${POLYNAME[$index]}_intra_norm.xvg\
                  -hdevice PNG -hardcopy\
                  -printfile
$PROD/analysis/rdf/"$BASENAME"_pngs/rdf_${POLYNAME[$index]}_intra_norm.png
      fi
((index++))
done

#water rdf
#these are done using the water oxygen atom instead of the whole molecule due to
artifacts caused by the virtual site
if [ $WATERNMOL -gt 0 ]; then

      group=$start
      index=0
      rdf=" "
      water="a OW\n "
      ((group++))

      while [ $index -lt 9 ]; do
            if [ "${POLYNUM[$index]}" != "0" ] ; then
                  rdf="${rdf}t peg_OA & r ${POLYNAME[$index]}\n name $group
OA_${POLYNAME[$index]}\n"
                  ((group++))
                  ether="t peg_OE & r ${POLYNAME[$index]}\n name $group
OE_${POLYNAME[$index]}\n"
                  ((group++))
                  rdf="${rdf}${ether}"
            fi
            ((index++))
      done

      rdf=${water}${rdf}

      echo -e "${rdf}\n q" | gmx make_ndx\
            -f "$PROD"/md.tpr\
            -o "$PROD"/analysis/rdf/rdf_water.ndx
#water-water rdf
      gmx rdf\
            -f "$PROD"/out/traj.xtc\
            -s "$PROD"/md.tpr\
            -n "$PROD"/analysis/rdf/rdf_water.ndx\
            -o "$PROD"/analysis/rdf/rdf_W_W.xvg\
            -ref $start -sel $start
            >> "$PROD"/analysis/hbond/possible.txt
      xmgrace\
            -nxy "$PROD"/analysis/rdf/rdf_W_W.xvg\
            -hdevice PNG -hardcopy\
            -printfile $PROD/analysis/rdf/"$BASENAME"_pngs/rdf_W_W.png

#water-OH and water_OE rdf
      group=$start
      index=0
      ((group++))
      while [ $index -lt 9 ]; do
            if [ "${POLYNUM[$index]}" != "0" ] ; then
            gmx rdf\
```

```
				-f "$PROD"/out/traj.xtc\
				-s "$PROD"/md.tpr\
				-n "$PROD"/analysis/rdf/rdf_water.ndx\
				-o "$PROD"/analysis/rdf/rdf_W_"${POLYNAME[$index]}".xvg\
				-ref $start -sel $group $(expr $group + 1)
				>> "$PROD"/analysis/hbond/possible.txt
			((group+= 2))
			xmgrace\
				-nxy "$PROD"/analysis/rdf/rdf_W_"${POLYNAME[$index]}".xvg\
				-hdevice PNG -hardcopy\
				-printfile
$PROD/analysis/rdf/"$BASENAME"_pngs/rdf_W_"${POLYNAME[$index]}".png
			fi
		((index++))
		done


fi

###END RDF

###HBOND
mkdir $PROD/analysis/hbond

#water-water hbond
if [ $WATERNMOL -gt 0 ]; then
	echo -e "$(expr $start - 1)\n$(expr $start - 1)\n" | gmx hbond\
		-f "$PROD"/out/traj.xtc\
		-s "$PROD"/md.tpr\
		-num "$PROD"/analysis/hbond/hbond_W_W.xvg\
		>> "$PROD"/analysis/hbond/possible.txt
fi

#intermolecular (within the same residue: includes intramolecular, which need to be
subtracted away)
#index
group=$start
index=0
hbond=" "
while [ $index -lt 9 ]; do
	if [ "${POLYNUM[$index]}" != "0" ] ; then
	hbond="${hbond}t peg_OA | t peg_HA & r "${POLYNAME[$index]}" \n name $group
OH_"${POLYNAME[$index]}"\n"
	((group++))
		if [ "${POLYNAME[$index]}" != "MEG" ]; then
		ether="t peg_OE & r "${POLYNAME[$index]}"\n name $group
OE_"${POLYNAME[$index]}"\n"
		((group++))
		hbond="${hbond}${ether}"
		fi
	fi
((index++))
done
echo -e "${hbond}\n q" | gmx make_ndx -f "$PROD"/md.tpr -o
"$PROD"/analysis/hbond/hbond_total.ndx
#echo -e "hbond total input:\n$hbond"
```

```
#hbond
group=$start
index=0
while [ $index -lt 9 ]; do
      if [ "${POLYNUM[$index]}" != "0" ] ; then
            echo -e "$group\n$group\n" | gmx hbond\
                  -f "$PROD"/out/traj.xtc\
                  -s "$PROD"/md.tpr\
                  -n "$PROD"/analysis/hbond/hbond_total.ndx\
                  -num
"$PROD"/analysis/hbond/hbond_"${POLYNAME[$index]}"_total_OH.xvg\
                  >> "$PROD"/analysis/hbond/possible.txt
            echo -e "$group\n$(expr $group + 1)\n" | gmx hbond\
                  -f "$PROD"/out/traj.xtc\
                  -s "$PROD"/md.tpr\
                  -n "$PROD"/analysis/hbond/hbond_total.ndx\
                  -num
"$PROD"/analysis/hbond/hbond_"${POLYNAME[$index]}"_total_OE.xvg\
                  >> "$PROD"/analysis/hbond/possible.txt
            let group="$group + 2"
      fi
((index++))
done

#intramolecular
#index
group=$start
index=0
hbond=" "
offset=0
while [ $index -lt 9 ]; do
      if [ "${POLYNUM[$index]}" != "0" ]; then
            mol=1
                  if [ ${POLYNUM[$index]} -lt 10 ]; then
                        while [ $mol -le ${POLYNUM[$index]} ]; do
                        let num="$mol + $offset"
                        hbond="${hbond}t peg_OA | t peg_HA & r "${POLYNAME[$index]}"
&ri $num \n name $group OH_"${POLYNAME[$index]}"_m"$num"\n"
                        ((group++))
                              if [ "${POLYNAME[$index]}" != "MEG" ]; then
                              ether="t peg_OE & r "${POLYNAME[$index]}" &ri $num\n name
$group OE_"${POLYNAME[$index]}"_m"$num"\n"
                              ((group++))
                              hbond="${hbond}${ether}"
                              fi
                        ((mol++))
                        done
                  else
                        while [ $mol -le 10 ]; do
                        let num="$mol + $offset"
                        hbond="${hbond}t peg_OA | t peg_HA & r "${POLYNAME[$index]}"
&ri $num \n name $group OH_"${POLYNAME[$index]}"_m"$num"\n"
                        ((group++))
                              if [ "${POLYNAME[$index]}" != "MEG" ]; then
                              ether="t peg_OE & r "${POLYNAME[$index]}" &ri $num\n name
$group OE_"${POLYNAME[$index]}"_m"$num"\n"
                              ((group++))
```

```
                              hbond="${hbond}${ether}"
                              fi
                        ((mol++))
                        done
                  fi
      offset=(${offset}+"${POLYNUM[$index]}")
      fi
((index++))
done
echo -e "${hbond}\n q" | gmx make_ndx -f "$PROD"/md.tpr -o
"$PROD"/analysis/hbond/hbond_intra.ndx
#echo -e "hbond input intra:\n$hbond"

#hbond
group=$start
index=0
mkdir $PROD/analysis/hbond/intra
while [ $index -lt 9 ]; do
      if [ "${POLYNUM[$index]}" != "0" ] ; then
            mol=1
                  if [ ${POLYNUM[$index]} -lt 10 ]; then
                        while [ $mol -le ${POLYNUM[$index]} ]; do
                        echo -e "$group\n$group\n" | gmx hbond\
                              -f "$PROD"/out/traj.xtc\
                              -s "$PROD"/md.tpr\
                              -n "$PROD"/analysis/hbond/hbond_intra.ndx\
                              -num
$PROD/analysis/hbond/intra/hbond_"${POLYNAME[$index]}"_intra_OH_$mol.xvg\
                              >>
"$PROD"/analysis/hbond/intra/possible_"${POLYNAME[$index]}"_intra_OH.txt
                        echo -e "$group\n$(expr $group + 1)\n" | gmx hbond\
                              -f "$PROD"/out/traj.xtc\
                              -s "$PROD"/md.tpr\
                              -n "$PROD"/analysis/hbond/hbond_intra.ndx\
                              -num
$PROD/analysis/hbond/intra/hbond_"${POLYNAME[$index]}"_intra_OE_$mol.xvg\
                              >>
$PROD/analysis/hbond/intra/possible_"${POLYNAME[$index]}"_intra_OE.txt
                        let group="$group + 2"
                        ((mol++))
                        done
                  else
#saves the output to a seperate txt file to be averaged
                        while [ $mol -le 10 ]; do
                        echo -e "$group\n$group\n" | gmx hbond\
                              -f "$PROD"/out/traj.xtc\
                              -s "$PROD"/md.tpr\
                              -n "$PROD"/analysis/hbond/hbond_intra.ndx\
                              -num
$PROD/analysis/hbond/intra/hbond_"${POLYNAME[$index]}"_intra_OH_$mol.xvg\
                              >>
"$PROD"/analysis/hbond/intra/possible_"${POLYNAME[$index]}"_intra_OH.txt
                        echo -e "$group\n$(expr $group + 1)\n" | gmx hbond\
                              -f "$PROD"/out/traj.xtc\
                              -s "$PROD"/md.tpr\
                              -n "$PROD"/analysis/hbond/hbond_intra.ndx\
```

```
                            -num
$PROD/analysis/hbond/intra/hbond_"${POLYNAME[$index]}"_intra_OE_$mol.xvg\
                            >>
$PROD/analysis/hbond/intra/possible_"${POLYNAME[$index]}"_intra_OE.txt
                        let group="$group + 2"
                        ((mol++))
                        done
                  fi
      fi
((index++))
done

#averaging for OH
index=0
count=0
while [ $index -lt 9 ]; do
      if [ "${POLYNUM[$index]}" != "0" ] ; then
#copies the line containing the average number of H bonds to a seperate file to be
read
# line reads "Average number of hbonds per timeframe # out of # possible"
            awk '/Average/'
"$PROD/analysis/hbond/intra/possible_"${POLYNAME[$index]}"_intra_OH.txt"\
                  >
"$PROD/analysis/hbond/intra/bondline_"${POLYNAME[$index]}"_intra_OH.txt"
            sum=0
            count=0
#reads each line saving each word to the corresponding variable
            while read -r average number of hbonds per timeframe bonds out of total
possible; do
#0 is saved as undefined and then cannot be used in math. this saves 0 as 0
                  bonds=$(echo "$bonds + 0" | bc -l)
#finds the sum of the bonds, for averaging
                  sum=$(echo "$sum + $bonds" | bc -l)
#counts the number of entries, for averaging
                  ((count++))
                  all=$total
            done <
"$PROD/analysis/hbond/intra/bondline_"${POLYNAME[$index]}"_intra_OH.txt"
#takes the average and saves to a file
            ave=$(echo " $sum / $count " | bc -l)
            echo ""${POLYNAME[$index]}" intra OH: $ave out of $all" >>
"$PROD/analysis/hbond/intra_averages.txt"
      fi
((index++))
done

#averaging for OE
#same as above
index=0
count=0
while [ $index -lt 9 ]; do
      if [ "${POLYNUM[$index]}" != "0" ] ; then
            awk '/Average/'
$PROD/analysis/hbond/intra/possible_"${POLYNAME[$index]}"_intra_OE.txt >
$PROD/analysis/hbond/intra/bondline_"${POLYNAME[$index]}"_intra_OE.txt
            sum=0
            count=0
```

```
            while read -r average number of hbonds per timeframe bonds out of total
possible; do
                  bonds=$(echo "$bonds + 0" | bc -l)
                  sum=$(echo "$sum + $bonds" | bc -l)
                  ((count++))
            all=$total
            done <
"$PROD/analysis/hbond/intra/bondline_"${POLYNAME[$index]}"_intra_OE.txt"
            ave=$(echo " $sum / $count " | bc -l)
            echo ""${POLYNAME[$index]}" intra OE: $ave out of $all" >>
$PROD/analysis/hbond/intra_averages.txt
      fi
((index++))
done

#intermolecular interresidual
#index
group=$start
index=0
hbond=" "
while [ $index -lt 9 ]; do
      if [ "${POLYNUM[$index]}" != "0" ] ; then
      hbond="${hbond}t peg_OA | t peg_HA & r "${POLYNAME[$index]}" \n name $group
OH_"${POLYNAME[$index]}"\n"
      ((group++))
      hbond="${hbond}t peg_OA | t peg_HA &! r "${POLYNAME[$index]}"\n name $group
OH_!"${POLYNAME[$index]}"\n"
      ((group++))
            if [ "${POLYNAME[$index]}" != "MEG" ]; then
            ether="t peg_OE &! r "${POLYNAME[$index]}"\n name $group
OE_!"${POLYNAME[$index]}"\n"
            ((group++))
            hbond="${hbond}${ether}"
            fi
      fi
((index++))
done
#echo -e "hbond inter input:\n$hbond"
echo -e "${hbond}\n q" | gmx make_ndx -f "$PROD"/md.tpr -o
"$PROD"/analysis/hbond/hbond_inter.ndx
#hbond
group=$start
index=0
while [ $index -lt 9 ]; do
      if [ "${POLYNUM[$index]}" != "0" ] ; then
            echo -e "$group\n$(expr $group + 1)\n" | gmx hbond\
                  -f "$PROD"/out/traj.xtc\
                  -s "$PROD"/md.tpr\
                  -n "$PROD"/analysis/hbond/hbond_inter.ndx\
                  -num
"$PROD"/analysis/hbond/hbond_"${POLYNAME[$index]}"_inter_OH.xvg\
                  >> "$PROD"/analysis/hbond/possible.txt
            echo -e "$group\n$(expr $group + 2)\n" | gmx hbond\
                  -f "$PROD"/out/traj.xtc\
                  -s "$PROD"/md.tpr\
                  -n "$PROD"/analysis/hbond/hbond_inter.ndx\
```

```
                  -num
"$PROD"/analysis/hbond/hbond_"${POLYNAME[$index]}"_inter_OE.xvg\
                  >> "$PROD"/analysis/hbond/possible.txt
            if [ $WATERNMOL -gt 0 ]; then
                  echo -e "$group\n$(expr $start - 1)\n" | gmx hbond\
                        -f "$PROD"/out/traj.xtc\
                        -s "$PROD"/md.tpr\
                        -n "$PROD"/analysis/hbond/hbond_inter.ndx\
                        -num
"$PROD"/analysis/hbond/hbond_"${POLYNAME[$index]}"_OH_W.xvg\
                        >> "$PROD"/analysis/hbond/possible.txt
            fi
            let group="$group + 3"
      fi
((index++))
done

#ethers to water hbonds
if [ $WATERNMOL != 0 ];then
      group=$start
      index=0
      ether=" "
      while [ $index -lt 9 ]; do
            if [ "${POLYNUM[$index]}" != "0" ] ; then
                  if [ "${POLYNAME[$index]}" != "MEG" ]; then
                  ether="${ether}t peg_OE & r "${POLYNAME[$index]}"\n name $group
OE_"${POLYNAME[$index]}"\n"
                  ((group++))
                  fi
            fi
      ((index++))
      done
      echo -e "${ether}\n q" | gmx make_ndx -f "$PROD"/md.tpr -o
"$PROD"/analysis/hbond/hbond_water.ndx

      group=$start
      index=0
      while [ $index -lt 9 ]; do
            if [ "${POLYNUM[$index]}" != "0" ] ; then
                  echo -e "$group\n$(expr $start - 1)\n" | gmx hbond\
                        -f "$PROD"/out/traj.xtc\
                        -s "$PROD"/md.tpr\
                        -n "$PROD"/analysis/hbond/hbond_water.ndx\
                        -num
"$PROD"/analysis/hbond/hbond_"${POLYNAME[$index]}"_OE_W.xvg\
                        >> "$PROD"/analysis/hbond/possible.txt
                  ((group++))
            fi
      ((index++))
      done
fi

###END HBOND

###DIHEDRALS
mkdir -p "$PROD"/analysis/dihedrals
```

```
gmx mk_angndx -s "$PROD"/md.tpr -n "$PROD"/analysis/dihedrals/RB_dih.ndx -type
ryckaert-bellemans

dihgroup=6
index=0

while [ $index -lt 9 ]; do
      if [ "${POLYNUM[$index]}" != "0" ] ; then
            echo "$dihgroup" | gmx angle\
                  -n "$PROD"/analysis/dihedrals/RB_dih.ndx\
                  -f "$PROD"/out/traj.xtc\
                  -type ryckaert-bellemans\
                  -od "$PROD"/analysis/dihedrals/"${POLYNAME[index]}"_OaCCOe_RB.xvg
            xmgrace -nxy
"$PROD"/analysis/dihedrals/"${POLYNAME[index]}"_OaCCOe_RB.xvg\
                  -hdevice PNG -hardcopy\
                  -printfile
"$PROD"/analysis/dihedrals/"${POLYNAME[index]}"_OaCCOe_RB.png
            echo -e "${POLYNAME[index]} (0,120)\n" >> "$PROD"/analysis/dihedrals.txt
            gmx analyze\
                  -f "$PROD"/analysis/dihedrals/"${POLYNAME[index]}"_OaCCOe_RB.xvg\
                  -b 0 -e 120 -integrate >> "$PROD"/analysis/dihedrals.txt
            echo -e "${POLYNAME[index]} (-120,0)\n" >> "$PROD"/analysis/dihedrals.txt
            gmx analyze\
                  -f "$PROD"/analysis/dihedrals/"${POLYNAME[index]}"_OaCCOe_RB.xvg\
                  -b -120 -e 0 -integrate >> "$PROD"/analysis/dihedrals.txt
            if [ ${POLYNAME[$index]} = "DEG" ]; then
                  ((dihgroup += 7))
            else
                  ((dihgroup +=8))
            fi
      fi
((index++))
done
###END DIHEDRALS

###GYRATION
mkdir $PROD/analysis/gyration

echo "q" | gmx make_ndx -f $PROD/md.tpr -o $PROD/analysis/gyration/gyrate.ndx

gyr_group=2
index=0
while [ $index -lt 9 ]; do
      if [ "${POLYNUM[$index]}" != "0" ] ; then
            echo "$gyr_group" | gmx gyrate\
                  -f $PROD/out/traj.xtc\
                  -s $PROD/md.tpr\
                  -o $PROD/analysis/gyration/gyrate_"${POLYNAME[$index]}".xvg\
                  -nmol "${POLYNUM[$index]}"\
                  -n $PROD/analysis/gyration/gyrate.ndx
            echo "${POLYNAME[$index]}" >> $PROD/analysis/gyrate.txt
            gmx analyze\
                  -f $PROD/analysis/gyration/gyrate_"${POLYNAME[$index]}".xvg\
                  >> $PROD/analysis/gyrate.txt
            ((gyr_group++))
      fi
```

```
((index++))
done

#water gyration
if [ ${WATERNMOL} -gt 0 ]; then
      echo "$(expr $start - 1)" | gmx gyrate\
            -f $PROD/out/traj.xtc\
            -s $PROD/md.tpr\
            -o $PROD/analysis/gyration/gyrate_water.xvg\
            -nmol $WATERNMOL\
            -n $PROD/analysis/gyration/gyrate.ndx
      echo "water" >> $PROD/analysis/gyrate.txt
      gmx analyze\
            -f $PROD/analysis/gyration/gyrate_water.xvg\
            >> $PROD/analysis/gyrate.txt
      ((gyr_group++))
fi

###END GYRATION
```

6 Oligomer-Oligomer Radial Distribution Functions

DEG: diethylene glycol

REG: triethylene glycol

TEG: tetraethylene glycol

PEG: Pentaethylene glycol

XEG: Hexaethylene glycol

HEG: Heptaethyene glycol

6.1 RDFs obtained from unmodified OPLS Forcefield

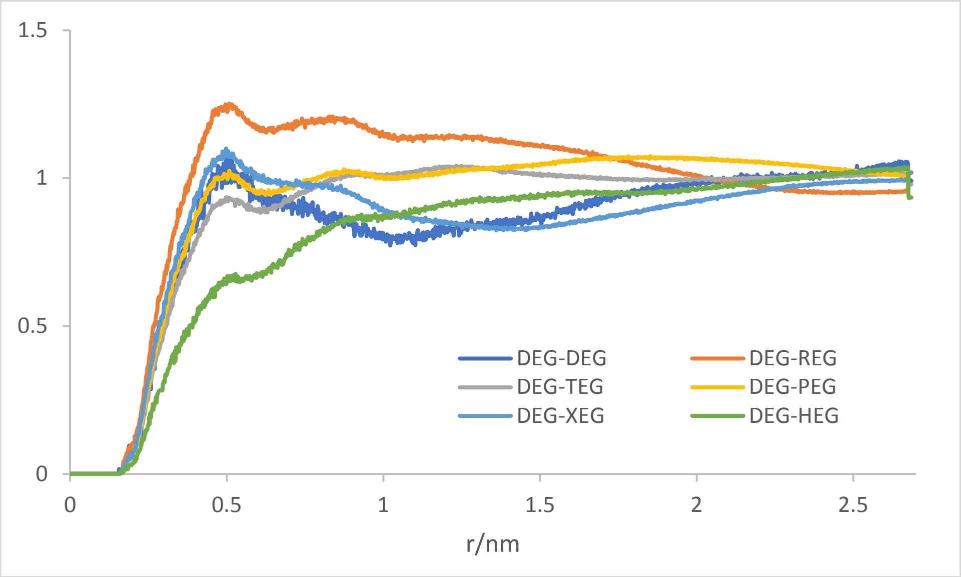

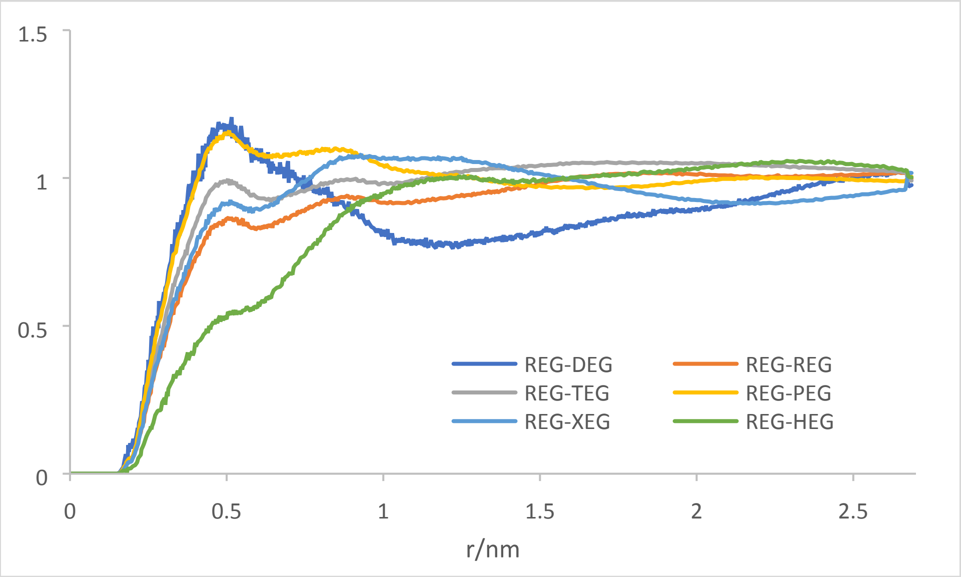
1.5
1
0.5
0
0
0.5
1
1.5
2
2.5
r/nm
REG-DEG
REG-REG
REG-TEG
REG-PEG
REG-XEG
REG-HEG

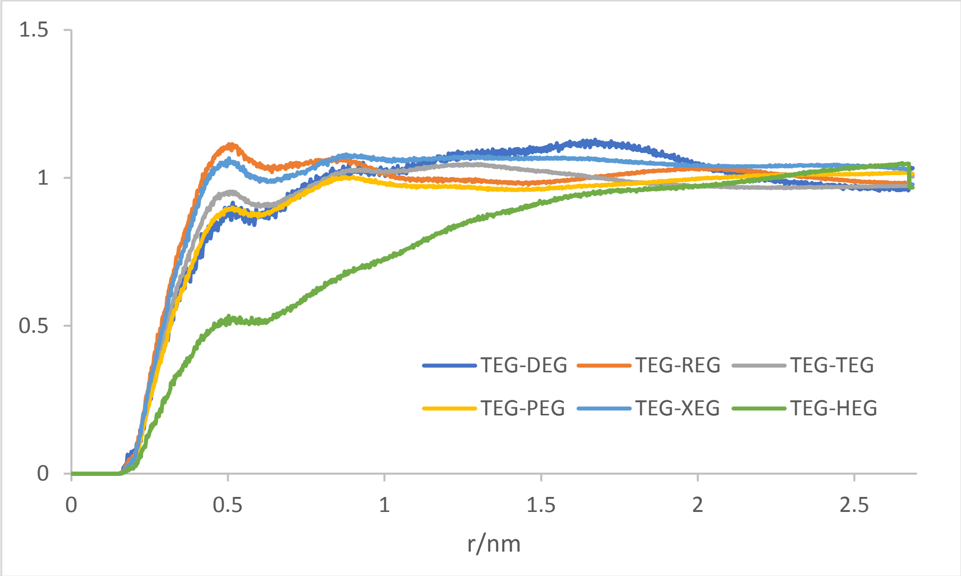
1.5
1
0.5
0
0
0.5
1
1.5
2
2.5
r/nm
TEG-DEG
TEG-REG
TEG-TEG
TEG-PEG
TEG-XEG
TEG-HEG

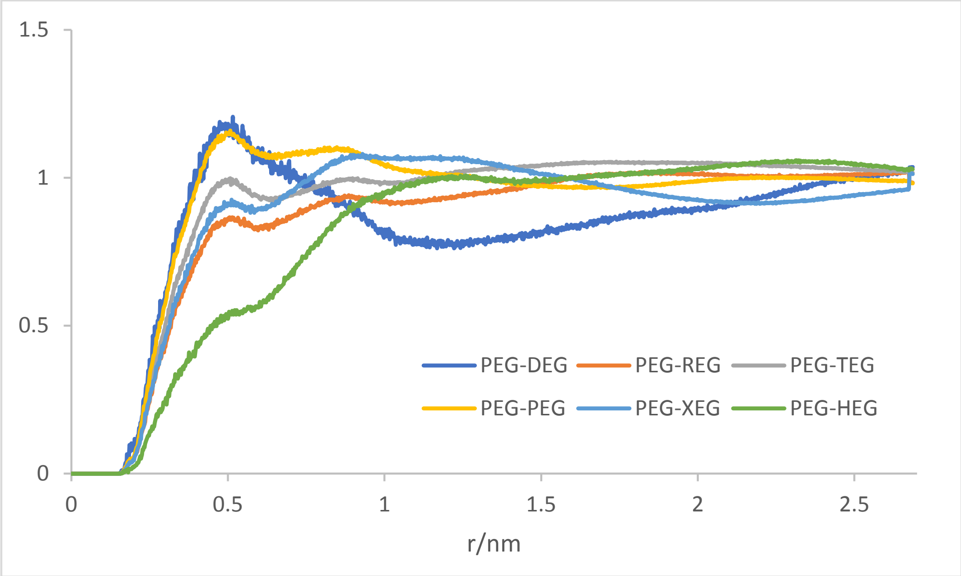
1.5
1
0.5
0
0
0.5
1
1.5
2
2.5
r/nm
PEG-DEG
PEG-REG
PEG-TEG
PEG-PEG
PEG-XEG
PEG-HEG

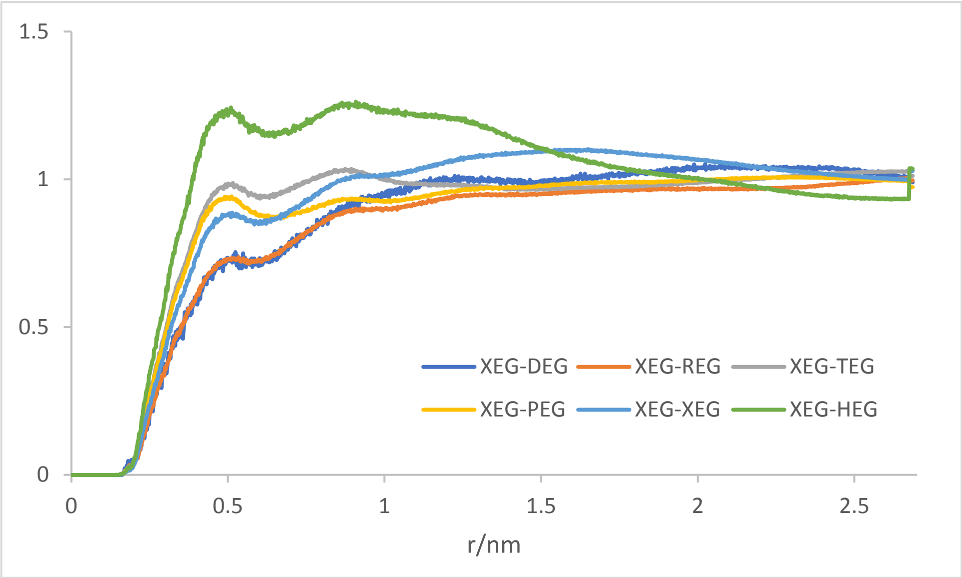
1.5
1
0.5
0
0
0.5
1
1.5
2
2.5
r/nm
XEG-DEG
XEG-REG
XEG-TEG
XEG-PEG
XEG-XEG
XEG-HEG

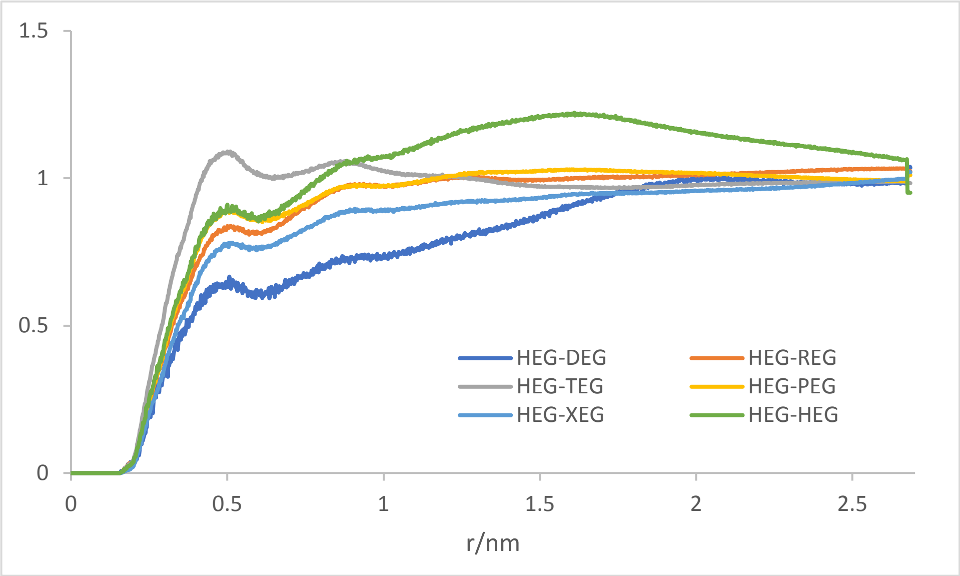
1.5
1
0.5
0
0
0.5
1
1.5
2
2.5
r/nm
HEG-DEG
HEG-REG
HEG-TEG
HEG-PEG
HEG-XEG
HEG-HEG

6.2 Modified OPLS forcefield

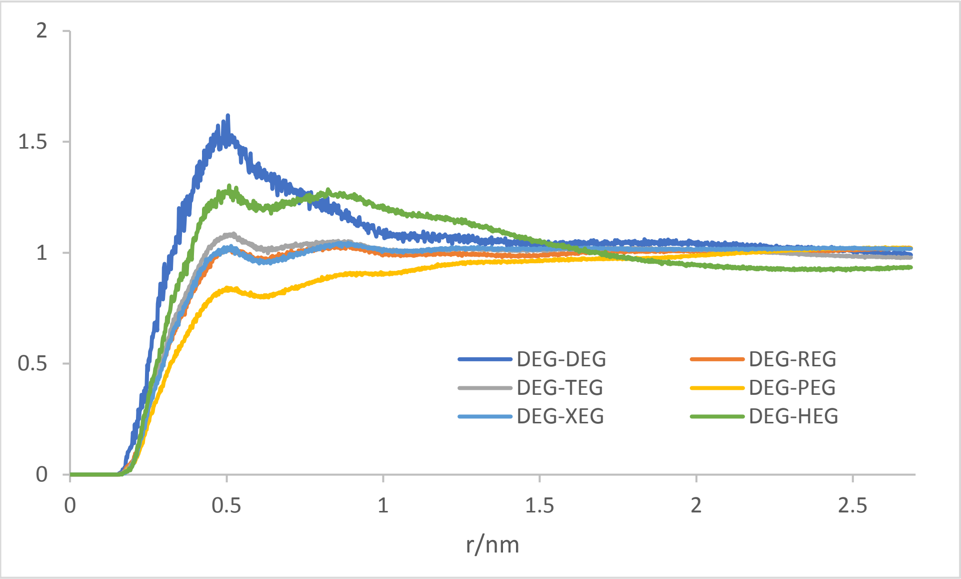

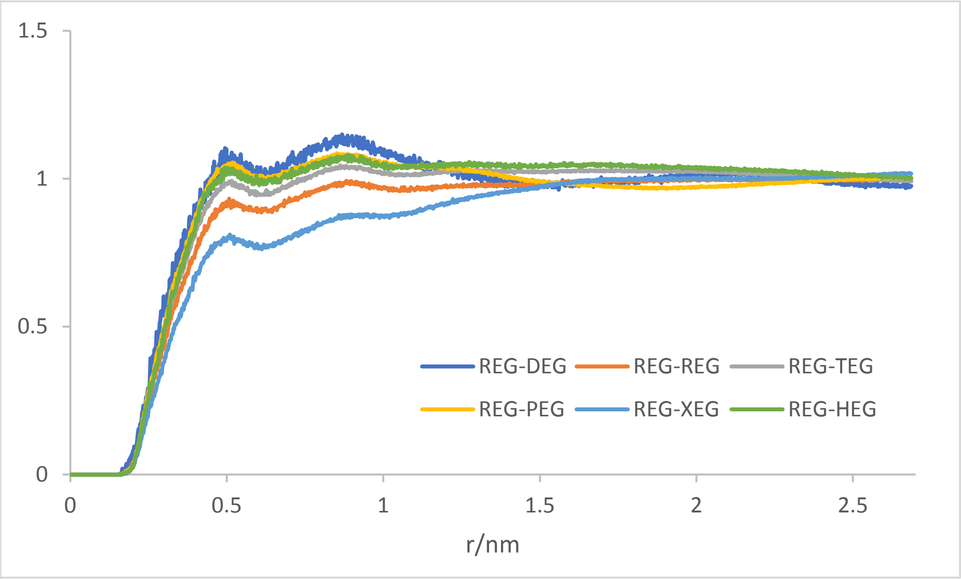

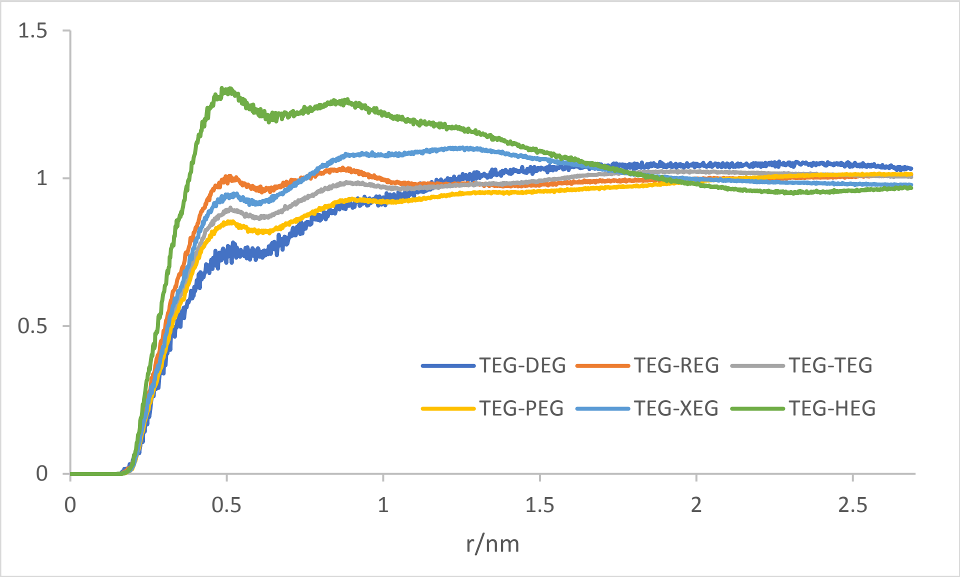
1.5
1
0.5
0
0
0.5
1
1.5
2
2.5
r/nm
TEG-DEG
TEG-REG
TEG-TEG
TEG-PEG
TEG-XEG
TEG-HEG

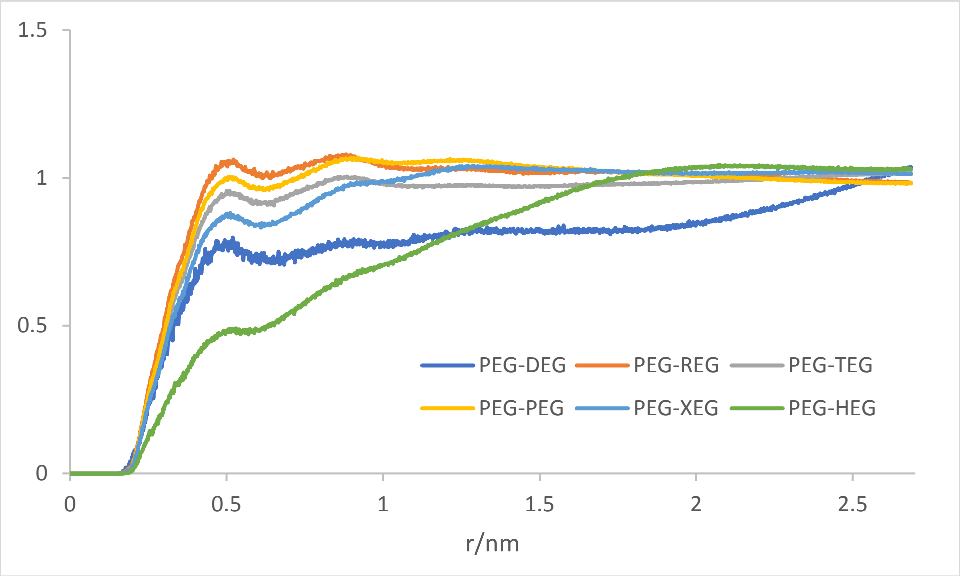
1.5
1
0.5
0
0
0.5
1
1.5
2
2.5
r/nm
PEG-DEG
PEG-REG
PEG-TEG
PEG-PEG
PEG-XEG
PEG-HEG

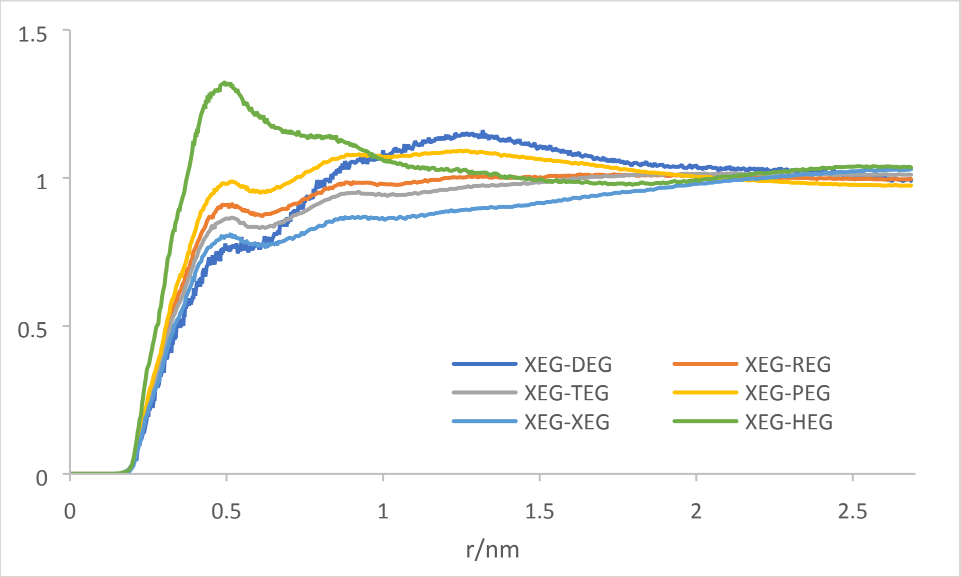
1.5
1
0.5
0
0
0.5
1
1.5
2
2.5
r/nm
XEG-DEG
XEG-REG
XEG-TEG
XEG-PEG
XEG-XEG
XEG-HEG

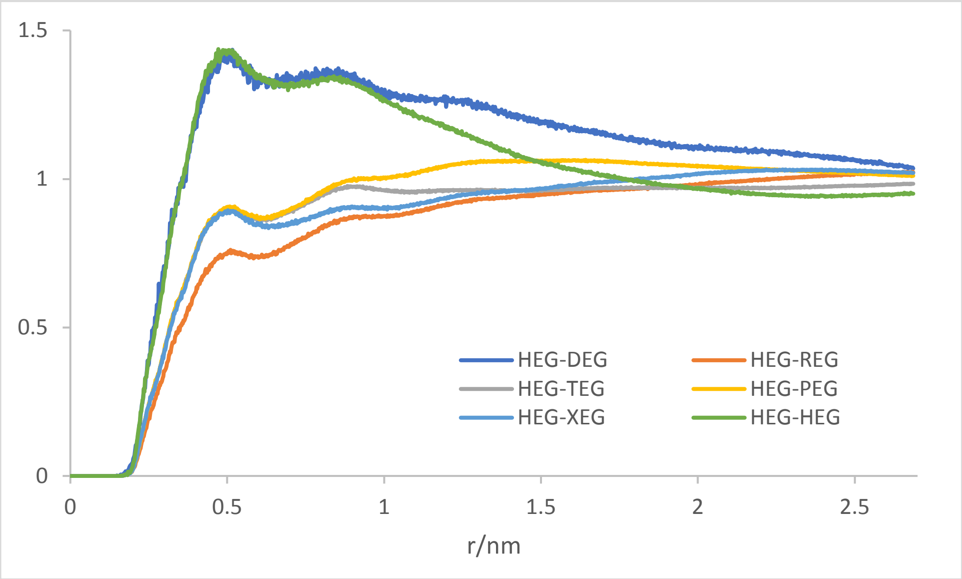

1.5
1
0.5
0
0
0.5
1
1.5
2
2.5
r/nm
HEG-DEG
HEG-REG
HEG-TEG
HEG-PEG
HEG-XEG
HEG-HEG

7 Hydrogen bonding analysis results of various PEG200 runs

Each set of tables is for one MD simulation run. Abbreviations used:

ref: specifies the to which moiety and molecule the hydrogen bonds refer to

DEG: diethylene glycol

REG: triethylene glycol

TEG: tetraethylene glycol

PEG: Pentaethylene glycol

XEG: Hexaethylene glycol

HEG: Heptaethyene glycol

OH: hydroxyl group

OE: ether oxygen

/nmol: the number of hydrogen bonds divided by the number of oligomer molecules in the PEG200 mixture

/possible: the number of hydrogen bonds divided by the number of possible hydrogen bonds.

!: “not” – for example “!DEG OH” means to all oligomer hydroxy groups but not diethylene glycol

MD simulation of PEG200 with OPLS forcefield, 500 molecules, random starting configuration

ref: DEG OH								
sel:		DEG OH			DEG OE		!DEG OH	!DEG OE
type:	total	intra	inter	total	intra	inter	inter	inter
bonds	0.50	0.15	0.35	0.54	0.49	0.05	23.21	8.83
possible	1122	34	1088	578	34	544	32844	56542
/nmol	0.02929	0.00890	0.02039	0.03176	0.02880	0.00296		
/possible	4.44E-04	4.45E-03	3.19E-04	9.34E-04	1.44E-02	9.26E-05	7.07E-04	1.56E-04
ref: REG OH								
sel:		REG OH			REG OE		!REG OH	!REG OE
type:	total	intra	inter	total	intra	inter	inter	inter
bonds	19.83	3.49	16.34	20.12	14.47	5.64	111.91	44.17
possible	49062	222	48840	49284	444	48840	172716	323676
/nmol	1.79E-01	3.14E-02	1.47E-01	1.81E-01	1.30E-01	5.08E-02		
/possible	4.04E-04	1.57E-02	3.35E-04	4.08E-04	3.26E-02	1.16E-04	6.48E-04	1.36E-04
ref: TEG OH								
sel:		TEG OH			TEG OE		!TEG OH	!TEG OE
type:	total	intra	inter	total	intra	inter	inter	Inter
bonds	40.40	9.41	31.00	44.65	26.34	18.32	136.65	41.78
possible	102080	320	101760	153600	960	152640	217600	384000
/nmol	2.53E-01	5.88E-02	1.94E-01	2.79E-01	1.65E-01	1.14E-01		
/possible	3.96E-04	2.94E-02	3.05E-04	2.91E-04	2.74E-02	1.20E-04	6.28E-04	1.09E-04
ref: PEG OH								
sel:		PEG OH			PEG OE		!PEG OH	!PEG OE
type:	total	intra	inter	total	intra	inter	inter	Inter
bonds	19.58	1.37	18.21	30.11	16.51	13.61	118.87	34.92
possible	60270	246	60024	121032	984	120048	185484	292248
/nmol	1.59E-01	1.11E-02	1.48E-01	2.45E-01	1.34E-01	1.11E-01		
/possible	3.25E-04	5.55E-03	3.03E-04	2.49E-04	1.68E-02	1.13E-04	6.41E-04	1.19E-04
ref: XEG OH								
sel:		XEG OH			XEG OE		!XEG OH	!XEG OE
type:	total	intra	inter	total	intra	inter	inter	Inter
bonds	5.81	0.64	5.18	14.32	9.10	5.22	71.93	21.01
possible	16770	130	16640	42250	650	41600	113100	176150
/nmol	8.94E-02	9.80E-03	7.96E-02	2.20E-01	1.40E-01	8.03E-02		
/possible	3.47E-04	4.90E-03	3.11E-04	3.39E-04	1.40E-02	1.25E-04	6.36E-04	1.19E-04
ref: HEG OH								
sel:		HEG OH			HEG OE		!HEG OH	!HEG OE
type:	total	intra	inter	total	intra	inter	inter	Inter
bonds	0.94	0.22	0.73	4.27	3.49	0.78	29.41	8.94
possible	2256	48	2208	6912	288	6624	45696	73728
/nmol	3.93E-02	9.00E-03	3.03E-02	1.78E-01	1.46E-01	3.24E-02		

/possible	4.18E-04	4.50E-03	3.30E-04	6.18E-04	1.21E-02	1.18E-04	6.44E-04	1.21E-04

MD simulation of PEG200 with OPLS forcefield, 1000 molecules, random starting configuration

ref: DEG OH

sel:		DEG OH			DEG OE		!DEG OH	!DEG OE
type:	total	intra	inter	total	intra	inter	inter	inter
bonds	1.36	0.34	1.02	1.18	0.96	0.22	47.67	17.52
possible	4830	70	4760	2450	70	2380	135100	232190
/nmol	0.03894	0.00970	0.02924	0.03377	0.02750	0.00627		
/possible	2.82E-04	4.85E-03	2.15E-04	4.82E-04	1.38E-02	9.22E-05	3.53E-04	7.54E-05

ref: REG OH

sel:		REG OH			REG OE		!REG OH	!REG OE
type:	total	intra	inter	total	intra	inter	inter	inter
bonds	40.17	7.23	32.95	40.31	28.32	11.99	226.46	87.29
possible	198470	446	198024	198916	892	198024	693084	1296076
/nmol	1.80E-01	3.24E-02	1.48E-01	1.81E-01	1.27E-01	5.38E-02		
/possible	2.02E-04	1.62E-02	1.66E-04	2.03E-04	3.18E-02	6.06E-05	3.27E-04	6.73E-05

ref: TEG OH

sel:		TEG OH			TEG OE		!TEG OH	!TEG OE
type:	total	intra	inter	total	intra	inter	inter	Inter
bonds	80.15	19.14	61.02	89.10	52.32	36.78	274.06	85.10
possible	408960	640	408320	614400	1920	612480	870400	1530880
/nmol	2.50E-01	5.98E-02	1.91E-01	2.78E-01	1.64E-01	1.15E-01		
/possible	1.96E-04	2.99E-02	1.49E-04	1.45E-04	2.73E-02	6.00E-05	3.15E-04	5.56E-05

ref: PEG OH

sel:		PEG OH			PEG OE		!PEG OH	!PEG OE
type:	total	intra	inter	total	intra	inter	inter	Inter
bonds	40.11	3.08	37.03	60.57	32.52	28.05	236.70	68.61
possible	241572	492	241080	484128	1968	482160	741936	1165056
/nmol	1.63E-01	1.25E-02	1.51E-01	2.46E-01	1.32E-01	1.14E-01		
/possible	1.66E-04	6.25E-03	1.54E-04	1.25E-04	1.65E-02	5.82E-05	3.19E-04	5.89E-05

ref: XEG OH

sel:		XEG OH			XEG OE		!XEG OH	!XEG OE
type:	total	intra	inter	total	intra	inter	inter	Inter
bonds	11.47	1.03	10.44	28.38	18.15	10.23	143.97	41.54
possible	66306	258	66048	166410	1290	165120	449436	698406
/nmol	8.89E-02	8.00E-03	8.09E-02	2.20E-01	1.41E-01	7.93E-02		
/possible	1.73E-04	4.00E-03	1.58E-04	1.71E-04	1.41E-02	6.19E-05	3.20E-04	5.95E-05

ref: HEG OH

sel:		HEG OH			HEG OE		!HEG OH	!HEG OE
type:	total	intra	inter	total	intra	inter	inter	Inter
bonds	1.58	0.24	1.34	8.28	6.66	1.62	56.99	17.20
possible	8742	94	8648	26508	564	25944	179164	288580

/nmol	3.37E-02	5.10E-03	2.86E-02	1.76E-01	1.42E-01	3.44E-02		
/possible	1.81E-04	2.55E-03	1.55E-04	3.12E-04	1.18E-02	6.23E-05	3.18E-04	5.96E-05

MD simulation of PEG200 with OPLS forcefield, 500 molecules, clustered starting configuration

ref: DEG OH

sel:		DEG OH			DEG OE		!DEG OH	!DEG OE
type:	total	intra	inter	total	intra	inter	inter	inter
bonds	0.52	0.15	0.37	0.52	0.49	0.03	23.47	8.64
possible	1122	34	1088	578	34	544	32844	56542
/nmol	0.03041	0.00880	0.02161	0.03041	0.02880	0.00161		
/possible	4.61E-04	4.40E-03	3.38E-04	8.94E-04	1.44E-02	5.04E-05	7.15E-04	1.53E-04

ref: REG OH

sel:		REG OH			REG OE		!REG OH	!REG OE
type:	total	intra	inter	total	intra	inter	inter	inter
bonds	22.99	3.41	19.58	21.14	14.19	6.96	109.24	42.48
possible	49062	222	48840	49284	444	48840	172716	323676
/nmol	2.07E-01	3.07E-02	1.76E-01	1.90E-01	1.28E-01	6.27E-02		
/possible	4.69E-04	1.54E-02	4.01E-04	4.29E-04	3.20E-02	1.42E-04	6.32E-04	1.31E-04

ref: TEG OH

sel:		TEG OH			TEG OE		!TEG OH	!TEG OE
type:	total	intra	inter	total	intra	inter	inter	inter
bonds	41.47	9.66	31.81	45.46	26.90	18.57	133.43	41.84
possible	102080	320	101760	153600	960	152640	217600	384000
/nmol	2.59E-01	6.04E-02	1.99E-01	2.84E-01	1.68E-01	1.16E-01		
/possible	4.06E-04	3.02E-02	3.13E-04	2.96E-04	2.80E-02	1.22E-04	6.13E-04	1.09E-04

ref: PEG OH

sel:		PEG OH			PEG OE		!PEG OH	!PEG OE
type:	total	intra	inter	total	intra	inter	inter	inter
bonds	22.17	1.33	20.84	32.16	16.72	15.45	113.42	32.86
possible	60270	246	60024	121032	984	120048	185484	292248
/nmol	1.80E-01	1.08E-02	1.69E-01	2.61E-01	1.36E-01	1.26E-01		
/possible	3.68E-04	5.40E-03	3.47E-04	2.66E-04	1.70E-02	1.29E-04	6.11E-04	1.12E-04

ref: XEG OH

sel:		XEG OH			XEG OE		!XEG OH	!XEG OE
type:	total	intra	inter	total	intra	inter	inter	inter
bonds	6.36	0.42	5.94	14.63	8.91	5.73	71.05	20.88
possible	16770	130	16640	42250	650	41600	113100	176150
/nmol	9.79E-02	6.50E-03	9.14E-02	2.25E-01	1.37E-01	8.81E-02		
/possible	3.79E-04	3.25E-03	3.57E-04	3.46E-04	1.37E-02	1.38E-04	6.28E-04	1.19E-04

ref: HEG OH

sel:		HEG OH			HEG OE		!HEG OH	!HEG OE
type:	total	intra	inter	total	intra	inter	inter	inter
bonds	1.22	0.16	1.06	4.67	3.65	1.02	28.85	8.28

possible	2256	48	2208	6912	288	6624	45696	73728
/nmol	5.08E-02	6.70E-03	4.41E-02	1.95E-01	1.52E-01	4.24E-02		
/possible	5.41E-04	3.35E-03	4.80E-04	6.75E-04	1.27E-02	1.54E-04	6.31E-04	1.12E-04

MD simulation of PEG200 with modified OPLS force field, 500 molecules, random starting configuration

ref: DEG OH

sel:		DEG OH			DEG OE		!DEG OH	!DEG OE
type:	total	intra	inter	total	intra	inter	inter	inter
bonds	0.89	0.40	0.49	1.01	0.94	0.07	13.94	4.70
possible	1122	34	1088	578	34	544	32844	56542
/nmol	0.05253	0.02370	0.02883	0.05924	0.05540	0.00384		
/possible	7.96E-04	1.19E-02	4.50E-04	1.74E-03	2.77E-02	1.20E-04	4.24E-04	8.32E-05

ref: REG OH

sel:		REG OH			REG OE		!REG OH	!REG OE
type:	total	intra	inter	total	intra	inter	inter	inter
bonds	18.27	11.16	7.11	29.82	27.43	2.39	39.56	11.74
possible	49062	222	48840	49284	444	48840	172716	323676
/nmol	1.65E-01	1.01E-01	6.41E-02	2.69E-01	2.47E-01	2.16E-02		
/possible	3.72E-04	5.03E-02	1.46E-04	6.05E-04	6.18E-02	4.90E-05	2.29E-04	3.63E-05

ref: TEG OH

sel:		TEG OH			TEG OE		!TEG OH	!TEG OE
type:	total	intra	inter	total	intra	inter	inter	inter
bonds	26.71	19.18	7.52	55.56	52.13	3.43	42.36	8.17
possible	102080	320	101760	153600	960	152640	217600	384000
/nmol	1.67E-01	1.20E-01	4.70E-02	3.47E-01	3.26E-01	2.15E-02		
/possible	2.62E-04	6.00E-02	7.39E-05	3.62E-04	5.43E-02	2.25E-05	1.95E-04	2.13E-05

ref: PEG OH

sel:		PEG OH			PEG OE		!PEG OH	!PEG OE
type:	total	intra	inter	total	intra	inter	inter	inter
bonds	7.19	1.62	5.56	36.15	33.33	2.82	38.02	7.11
possible	60270	246	60024	121032	984	120048	185484	292248
/nmol	5.84E-02	1.32E-02	4.52E-02	2.94E-01	2.71E-01	2.29E-02		
/possible	1.19E-04	6.60E-03	9.27E-05	2.99E-04	3.39E-02	2.35E-05	2.05E-04	2.43E-05

ref: XEG OH

sel:		XEG OH			XEG OE		!XEG OH	!XEG OE
type:	total	intra	inter	total	intra	inter	inter	inter
bonds	1.64	0.19	1.45	6.56	4.82	1.74	20.73	7.06
possible	16770	130	16640	42250	650	41600	113100	176150
/nmol	2.52E-02	2.90E-03	2.23E-02	1.01E-01	7.42E-02	2.68E-02		
/possible	9.77E-05	1.45E-03	8.71E-05	1.55E-04	7.42E-03	4.18E-05	1.83E-04	4.01E-05

ref: HEG OH

sel:		HEG OH			HEG OE		!HEG OH	!HEG OE
type:	total	intra	inter	total	intra	inter	inter	inter
bonds	0.23	0.05	0.18	2.06	1.85	0.20	8.38	2.97

possible	2256	48	2208	6912	288	6624	45696	73728
/nmol	9.63E-03	2.10E-03	7.53E-03	8.56E-02	7.71E-02	8.53E-03		
/possible	1.02E-04	1.05E-03	8.18E-05	2.97E-04	6.43E-03	3.09E-05	1.83E-04	4.03E-05

MD simulation of PEG200 with modified OPLS force field, 1000 molecules, random starting config.

ref: DEG OH

sel:		DEG OH			DEG OE		!DEG OH	!DEG OE
type:	total	intra	inter	total	intra	inter	inter	inter
bonds	1.76	0.85	0.91	2.05	1.92	0.13	29.06	9.78
possible	4830	70	4760	2450	70	2380	135100	232190
/nmol	0.05029	0.02430	0.02599	0.05869	0.05490	0.00379		
/possible	3.64E-04	1.22E-02	1.91E-04	8.38E-04	2.75E-02	5.57E-05	2.15E-04	4.21E-05

ref: REG OH

sel:		REG OH			REG OE		!REG OH	!REG OE
type:	total	intra	inter	total	intra	inter	inter	inter
bonds	36.44	22.55	13.90	59.89	54.86	5.03	80.65	23.65
possible	198470	446	198024	198916	892	198024	693084	1296076
/nmol	1.63E-01	1.01E-01	6.23E-02	2.69E-01	2.46E-01	2.26E-02		
/possible	1.84E-04	5.06E-02	7.02E-05	3.01E-04	6.15E-02	2.54E-05	1.16E-04	1.82E-05

ref: TEG OH

sel:		TEG OH			TEG OE		!TEG OH	!TEG OE
type:	total	intra	inter	total	intra	inter	inter	inter
bonds	53.30	37.79	15.51	110.81	106.18	4.63	85.79	16.36
possible	408960	640	408320	614400	1920	612480	870400	1530880
/nmol	1.67E-01	1.18E-01	4.85E-02	3.46E-01	3.32E-01	1.45E-02		
/possible	1.30E-04	5.91E-02	3.80E-05	1.80E-04	5.53E-02	7.56E-06	9.86E-05	1.07E-05

ref: PEG OH

sel:		PEG OH			PEG OE		!PEG OH	!PEG OE
type:	total	intra	inter	total	intra	inter	inter	inter
bonds	14.57	2.83	11.74	72.52	66.76	5.76	76.03	14.01
possible	241572	492	241080	484128	1968	482160	741936	1165056
/nmol	5.92E-02	1.15E-02	4.77E-02	2.95E-01	2.71E-01	2.34E-02		
/possible	6.03E-05	5.75E-03	4.87E-05	1.50E-04	3.39E-02	1.19E-05	1.02E-04	1.20E-05

ref: XEG OH

sel:		XEG OH			XEG OE		!XEG OH	!XEG OE
type:	total	intra	inter	total	intra	inter	inter	inter
bonds	3.24	0.32	2.92	12.98	9.38	3.60	41.31	13.92
possible	66306	258	66048	166410	1290	165120	449436	698406
/nmol	2.51E-02	2.50E-03	2.26E-02	1.01E-01	7.27E-02	2.79E-02		
/possible	4.88E-05	1.25E-03	4.41E-05	7.80E-05	7.27E-03	2.18E-05	9.19E-05	1.99E-05

ref: HEG OH

sel:		HEG OH			HEG OE		!HEG OH	!HEG OE

type:	total	intra	inter	total	intra	inter	inter	inter
bonds	0.47	0.07	0.40	3.99	3.46	0.53	16.38	5.74
possible	8742	94	8648	26508	564	25944	179164	288580
/nmol	9.91E-03	1.50E-03	8.41E-03	8.50E-02	7.36E-02	1.14E-02		
/possible	5.33E-05	7.50E-04	4.57E-05	1.51E-04	6.13E-03	2.06E-05	9.14E-05	1.99E-05

MD simulation of PEG200 with modified OPLS forcefield, 500 molecules, clustered starting configuration

ref: DEG OH

sel:		DEG OH			DEG OE		!DEG OH	!DEG OE
type:	total	intra	inter	total	intra	inter	inter	inter
bonds	0.78	0.40	0.38	0.98	0.93	0.04	14.36	4.79
possible	1122	34	1088	578	34	544	32844	56542
/nmol	0.04559	0.02330	0.02229	0.05753	0.05490	0.00263		
/possible	6.91E-04	1.17E-02	3.48E-04	1.69E-03	2.75E-02	8.22E-05	4.37E-04	8.47E-05

ref: REG OH

sel:		REG OH			REG OE		!REG OH	!REG OE
type:	total	intra	inter	total	intra	inter	inter	inter
bonds	18.09	11.32	6.76	29.70	27.46	2.24	40.14	11.63
possible	49062	222	48840	49284	444	48840	172716	323676
/nmol	1.63E-01	1.02E-01	6.09E-02	2.68E-01	2.47E-01	2.02E-02		
/possible	3.69E-04	5.10E-02	1.38E-04	6.03E-04	6.19E-02	4.59E-05	2.32E-04	3.59E-05

ref: TEG OH

sel:		TEG OH			TEG OE		!TEG OH	!TEG OE
type:	total	intra	inter	total	intra	inter	inter	inter
bonds	26.79	18.64	8.15	55.55	52.78	2.77	42.48	8.20
possible	102080	320	101760	153600	960	152640	217600	384000
/nmol	1.67E-01	1.17E-01	5.09E-02	3.47E-01	3.30E-01	1.73E-02		
/possible	2.62E-04	5.83E-02	8.01E-05	3.62E-04	5.50E-02	1.81E-05	1.95E-04	2.13E-05

ref: PEG OH

sel:		PEG OH			PEG OE		!PEG OH	!PEG OE
type:	total	intra	inter	total	intra	inter	inter	inter
bonds	7.13	1.50	5.63	36.33	33.49	2.83	38.17	7.19
possible	60270	246	60024	121032	984	120048	185484	292248
/nmol	5.80E-02	1.22E-02	4.58E-02	2.95E-01	2.72E-01	2.30E-02		
/possible	1.18E-04	6.10E-03	9.38E-05	3.00E-04	3.40E-02	2.36E-05	2.06E-04	2.46E-05

ref: XEG OH

sel:		XEG OH			XEG OE		!XEG OH	!XEG OE
type:	total	intra	inter	total	intra	inter	inter	inter
bonds	1.66	0.17	1.49	6.57	4.68	1.89	20.69	7.07
possible	16770	130	16640	42250	650	41600	113100	176150
/nmol	2.55E-02	2.60E-03	2.29E-02	1.01E-01	7.20E-02	2.91E-02		
/possible	9.87E-05	1.30E-03	8.93E-05	1.56E-04	7.20E-03	4.55E-05	1.83E-04	4.02E-05

ref: HEG OH

sel:		HEG OH			HEG OE		!HEG OH	!HEG OE
type:	total	intra	inter	total	intra	inter	inter	inter
bonds	0.27	0.05	0.22	2.11	1.80	0.31	8.31	2.94
possible	2256	48	2208	6912	288	6624	45696	73728
/nmol	1.13E-02	1.90E-03	9.35E-03	8.78E-02	7.49E-02	1.29E-02		
/possible	1.20E-04	9.50E-04	1.02E-04	3.05E-04	6.24E-03	4.67E-05	1.82E-04	3.99E-05

MD simulation of tri- and hexaethylene glycol with average molar weight of 200 g·mol^{-1} using OPLS force field, 500 molecules, random starting configuration

ref: DEG OH

sel:
type:
bonds
possible
/nmol
/possible

ref: REG OH

sel:		REG OH			REG OE		!REG OH	!REG OE
type:	total	intra	inter	total	intra	inter	inter	inter
bonds	142.25	9.42	132.83	86.24	38.75	47.49	161.65	83.71
possible	383780	620	383160	384400	1240	383160	235600	589000
/nmol	4.59E-01	3.04E-02	4.28E-01	2.78E-01	1.25E-01	1.53E-01		
/possible	3.71E-04	1.52E-02	3.47E-04	2.24E-04	3.13E-02	1.24E-04	6.86E-04	1.42E-04

ref: TEG OH

sel:
type:
bonds
possible
/nmol
/possible

ref: PEG OH

sel:
type:
bonds
possible
/nmol
/possible

ref: XEG OH

sel:		XEG OH			XEG OE		!XEG OH	!XEG OE
type:	total	intra	inter	total	intra	inter	inter	inter
bonds	48.28	1.54	46.74	71.36	25.31	46.05	161.65	26.54
possible	144020	380	143640	361000	1900	359100	235600	235600
/nmol	2.54E-01	8.10E-03	2.46E-01	3.76E-01	1.33E-01	2.42E-01		
/possible	3.35E-04	4.05E-03	3.25E-04	1.98E-04	1.33E-02	1.28E-04	6.86E-04	1.13E-04

ref: HEG OH								
sel:								
type:								
bonds								
possible								
/nmol								
/possible								

MD simulation of tri- and hexaethylene glycol with average molar weight of 200 g·mol^{-1} using modified OPLS force field, 500 molecules, random starting configuration

ref: DEG OH								
sel:								
type:								
bonds								
possible								
/nmol								
/possible								
ref: REG OH								
sel:		REG OH			REG OE		!REG OH	!REG OE
type:	total	intra	inter	total	intra	inter	inter	inter
bonds	86.84	31.62	55.22	94.32	75.58	18.74	48.17	21.33
possible	383780	620	383160	384400	1240	383160	235600	589000
/nmol	2.80E-01	1.02E-01	1.78E-01	3.04E-01	2.44E-01	6.05E-02		
/possible	2.26E-04	5.10E-02	1.44E-04	2.45E-04	6.10E-02	4.89E-05	2.04E-04	3.62E-05
ref: TEG OH								
sel:								
type:								
bonds								
possible								
/nmol								
/possible								
ref: PEG OH								
sel:								
type:								
bonds								
possible								
/nmol								
/possible								
ref: XEG OH								
sel:		XEG OH			XEG OE		!XEG OH	!XEG OE
type:	total	intra	inter	total	intra	inter	inter	inter
bonds	13.53	0.49	13.04	29.40	13.87	15.53	48.17	10.03
possible	144020	380	143640	361000	1900	359100	235600	235600

/nmol	7.12E-02	2.60E-03	6.86E-02	1.55E-01	7.30E-02	8.17E-02		
/possible	9.40E-05	1.30E-03	9.08E-05	8.14E-05	7.30E-03	4.33E-05	2.04E-04	4.26E-05
ref: HEG OH								
sel:								
type:								
bonds								
possible								
/nmol								
/possible								

8 Plots on intermolecular hydrogen bonding in PEG200

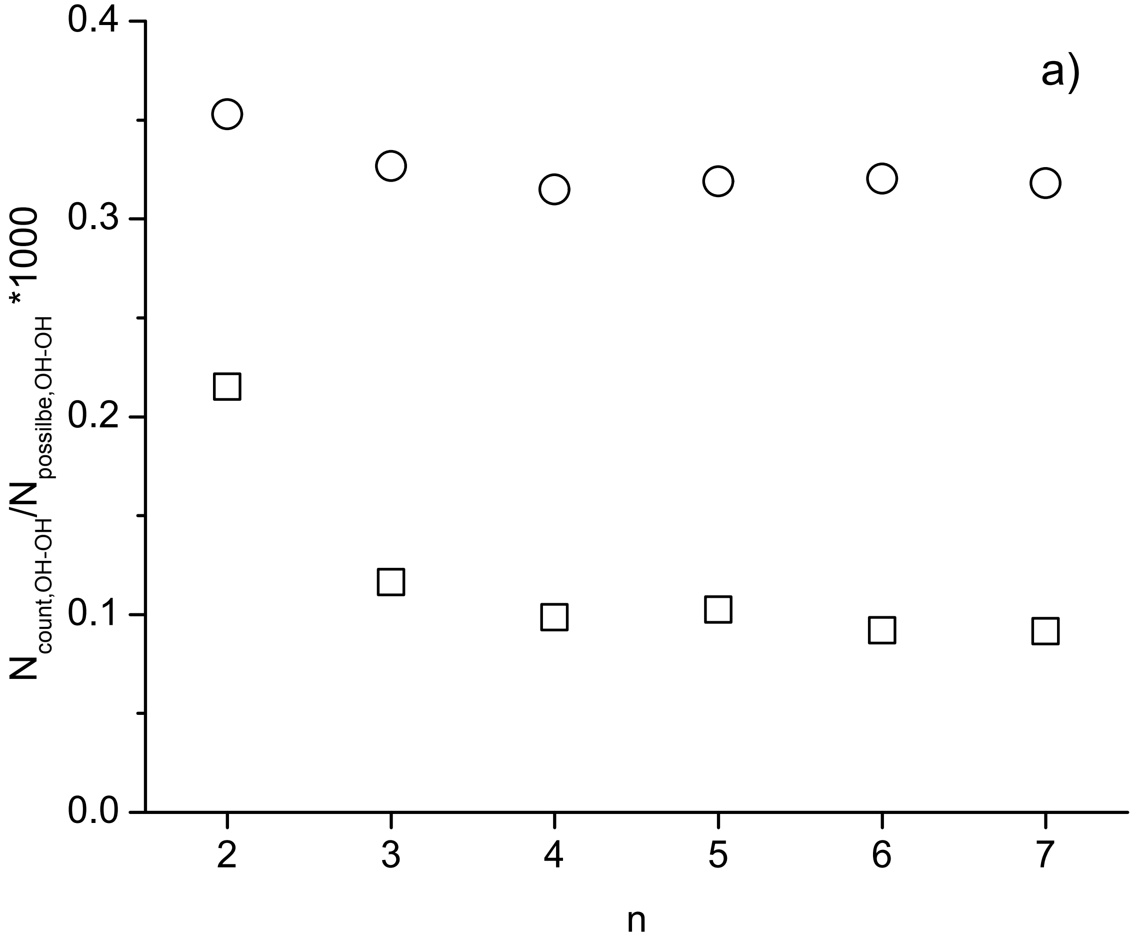

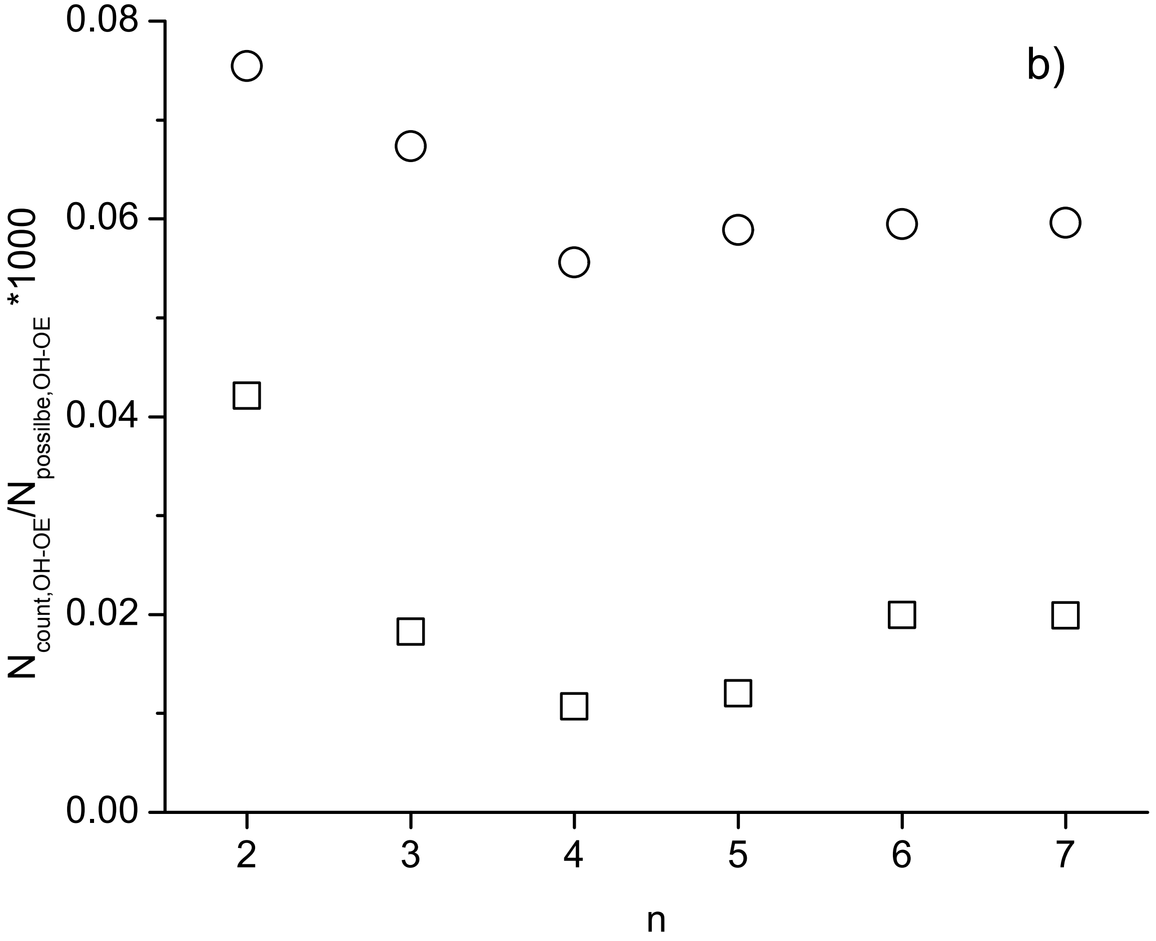

Figure S4: Intermolecular a) OH-OH and b) OH-OE hydrogen bonding of PEG200 simulated with OPLS force field (circles) and modified (see Table 4) OPLS force field (squares).
